# Panchromatic light funneling through the synergy in hexabenzocoronene–(metallo)porphyrin–fullerene assemblies to realize the separation of charges†

**DOI:** 10.1039/d0sc02028a

**Published:** 2020-06-17

**Authors:** Maximilian Wolf, Dominik Lungerich, Stefan Bauroth, Maximilian Popp, Benedikt Platzer, Timothy Clark, Harry L. Anderson, Norbert Jux, Dirk M. Guldi

**Affiliations:** Department of Chemistry and Pharmacy, Friedrich-Alexander-University Erlangen-Nuremberg, Interdisciplinary Center for Molecular Materials (ICMM) Egerlandstraße 3 91058 Erlangen Germany dirk.guldi@fau.de; Department of Chemistry and Pharmacy, Friedrich-Alexander-University Erlangen-Nuremberg, Interdisciplinary Center for Molecular Materials (ICMM) Nikolaus-Fiebiger-Str. 10 91058 Erlangen Germany; Department of Chemistry, University of Oxford, Chemistry Research Laboratory Oxford OX1 3TA UK; Department of Chemistry and Pharmacy, Computer Chemistry Centre (CCC), Friedrich-Alexander-University Germany

## Abstract

Here, we present a novel butadiyne-linked HBC-ethynyl-porphyrin dimer, which exhibits in the ground state strong absorption cross sections throughout the UV and visible ranges of the solar spectrum. In short, a unidirectional flow of excited state energy from the HBC termini to the (metallo)porphyrin focal points enables concentrating light at the latter. Control over excitonic interactions within, for example, the electron-donating porphyrin dimers was realized by complexation of bidentate ligands to set up panchromatic absorption that extends all the way into the near-infrared range. The bidentate binding motif was then exploited to create a supramolecular electron donor–acceptor assembly based on a HBC-ethynyl-porphyrin dimer and an electron accepting bis(aminoalkyl)-substituted fullerene. Of great relevance is the fact that charge separation from the photoexcited HBC-ethynyl-porphyrin dimer to the bis(aminoalkyl)-substituted fullerene is activated not only upon photoexciting the HBCs in the UV as well as the (metallo)porphyrins in the visible but also in the NIR. Implicit is the synergetic interplay of energy and charge transfer in a photosynthetic mimicking manner. The dimer and bis-HBC-ethynyl-porphyrin monomers, which serve as references, were probed by means of steady-state as well as time-resolved optical spectroscopies, including global target analyses of the time-resolved transient absorption data.

## Abstract 2

Here, we present a novel butadiyne-linked HBC-ethynyl-porphyrin dimer, which exhibits in the ground state strong absorption cross sections throughout the UV and visible ranges of the solar spectrum.

## Graphical abstract

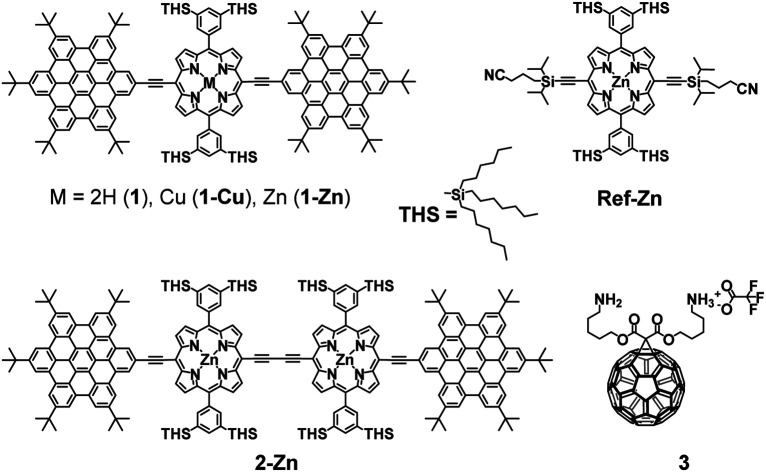

## Introduction

Exploiting the full spectrum of solar radiation for light harvesting applications remains a challenging task. In particular, architectures based on molecular chromophores are often limited to discrete absorption bands. This pitfall has been overcome in nature by means of self-assembled superstructures of chlorophylls, which made porphyrins one of the most important classes of molecular entities in photosynthesis. Nowadays, they are also important model compounds in fields as diverse as catalysis, molecular electronics, sensing, and, last but not least, electron-transfer applications.^1–19^ Their strong absorption throughout the visible part of the solar spectrum makes them useful for solar-energy applications. Much work has been devoted to refining and tuning these features. Ethynyl substitution, for example, of the porphyrin core in the *meso*- and/or β-positions has been shown to shift their absorption bands and alter the corresponding relative intensities.^20–26^

Porphyrin (homo/hetero) dimers linked by π-conjugated oligomeric bridges have been studied extensively, not only with regard to their spectroscopic properties, but also to their propensity to build supramolecular architectures and mediate charge transport between different porphyrins.^27–37^ Another way of tailoring porphyrins is to couple polycyclic aromatic hydrocarbons, PAHs, directly to the porphyrin core (*e.g.* tetraphenylporphyrins, TPPs). Likewise, the incorporation of peripheral ethynyl groups allows for tuning ground- and excited-state interactions.^25,26,38,39^ Hexa-*peri*-hexabenzo-coronenes (HBCs) have attracted a great deal of attention because of their electronic and optoelectronic properties. After a synthesis for soluble HBCs was published by Müllen and coworkers in the late 1990s,^40,41^ HBCs have recently found use as graphene model systems.^42,43^ Several reports have described the use of conjugates of HBC with perylenediimides and/or porphyrins for photochemical applications albeit with the use of rotationally flexible bonds.^44–50^ One of the detrimental consequences is a rather moderate electronic coupling between the individual building blocks and, in turn, an ineffective flow of charges across the entire conjugate in addition to structural flexibility, conformational freedom, *etc.*

**Fig. 1:**
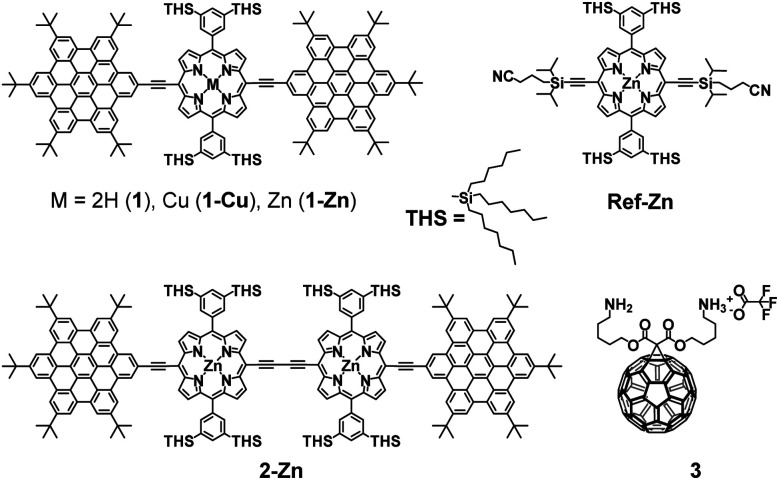
Conjugates and references synthesized and tested in the present work.

In this work, we present the design of a new family of covalently-linked, ethynyl-bridged (metallo)porphyrin-HBC conjugates in their free-base (**1**) and/or metallated with Cu (**1-Cu**) or Zn (**1-Zn**) forms, which were complemented by an HBC-terminated, butadiyne-bridged zinc-porphyrin (ZnP) dimer **2-Zn**. Of great importance for our molecular design is the choice of ethynyl bridges as they foster the panchromatic absorption throughout the UV and visible regions of the solar spectrum all the way into the near-infrared region. Going beyond this aspect, ethynyl bridges enable control over the electronic couplings between the HBCs and the (metallo)porphyrins, on one hand, and the flow of energy/charges, on the other hand. An incentive for placing the HBCs at the termini and the (metallo)porphyrins at the focal points is to set up a gradient along which a unidirectional flow of energy supports the pooling of light. Ultimately, the efficient utilization of the concentrated light is realized by coordinating, for example, an electron-accepting C_60_ to the (metallo)porphyrin dimer. As such, the synergy of the aforementioned characteristics enables the perfect blueprint of mimicking photosynthesis, namely the basic principles of light-harvesting arrays and reaction centers. The absorptive and emissive properties of all conjugates in their ground and excited states were characterized thoroughly by an arsenal of steady-state and time-resolved optical spectroscopies. The insight gathered for **1**, **1-Cu**, and **1-Zn** helped interpret the excited-state reactivity of **2-Zn** coordinated to **3**: the sequence of a unidirectional flow of energy followed by that of charges. Experimental results are complemented by molecular modeling to address the coordination-induced changes in the excited-state characteristics (Fig. 1).

For details of the synthetic procedures and characterization of intermediate products, please refer to the ESI.†

### Steady-state spectroscopy

**Fig. 2:**
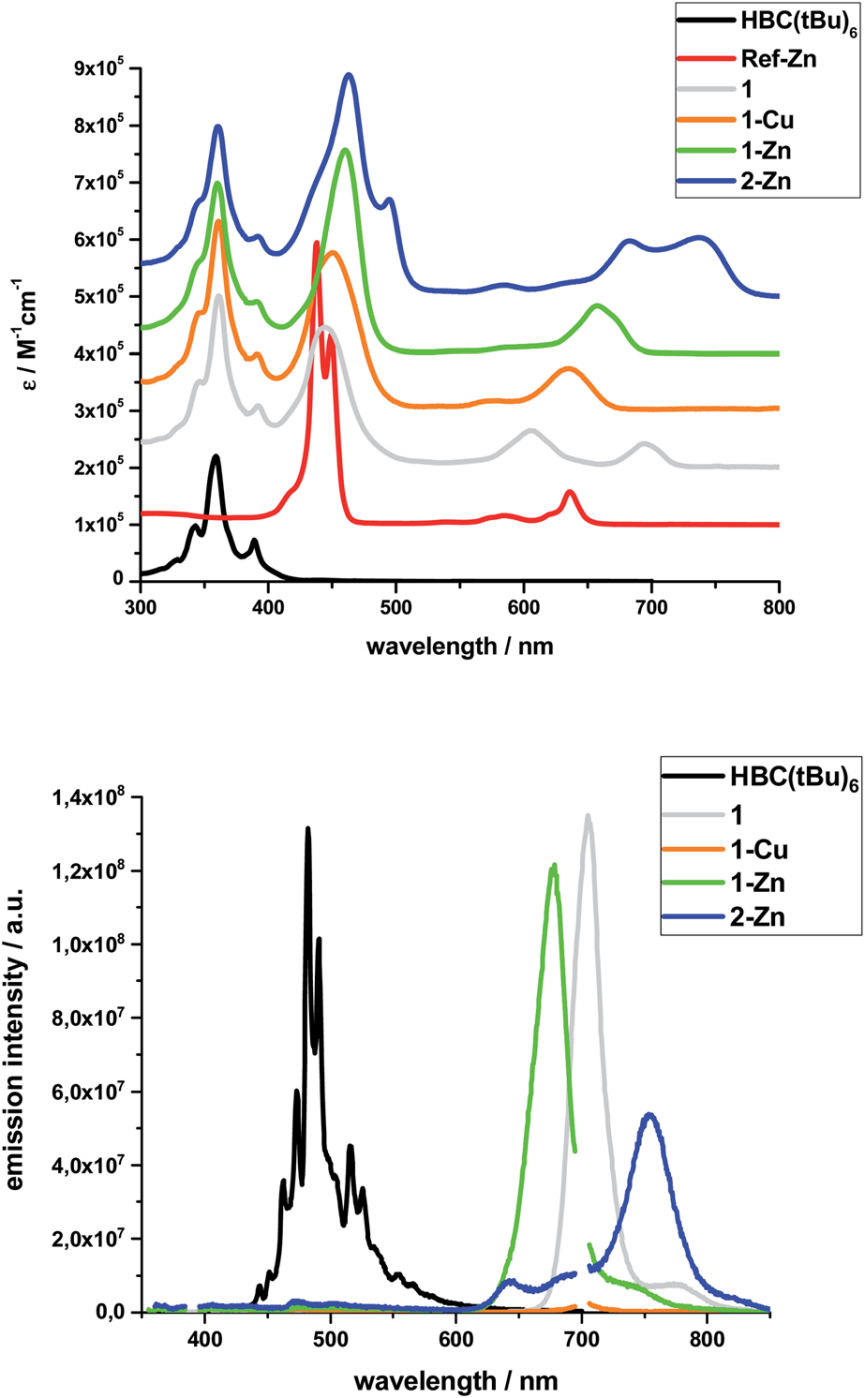
Top: Extinction coefficients of the investigated systems and references in THF (Note: individual spectra set off by 10^5^ M^−1^ cm^−1^ for clarity); Bottom: Fluorescence spectra of the investigated systems and reference **HBC(tBu)6** in THF normalized to the respective emission maximum; excitation at 350 nm for **1-Cu**, **1-Zn**, **2-Zn**, **HBC(tBu)6** (second order diffraction peak at 700 nm), 360 nm for **1**.

Steady-state absorption spectra of all HBC–porphyrin conjugates (**1**, **1-Cu**, **1-Zn**, and **2-Zn**) were measured in THF – Fig. 2, top. Table 1 summarizes the absorption features in terms of maxima and absorption coefficients.

**Table 1:** Peak positions with corresponding absorbances in parentheses for the HBC–porphyrin compounds and references (recorded in THF)^a^

Sample	Peak positions/nm, (*ε*/10^5^ M^−1^ cm^−1^)								
**1**	346 (1.52)	361 (3.00)	392 (1.08)	445 (2.46)	606 (0.65)	695 (0.42)			
**1-Cu**	346 sh (1.71)	361 (3.32)	391 (0.99)	450 (2.77)	577 sh (0.18)	634 (0.74)			
**1-Zn**	346 sh (1.62)	360 (2.99)	391 (0.91)	461 (3.57)	585 sh (0.11)	657 (0.84)			
**2-Zn**	346 sh (1.68)	361 (2.98)	392 (1.06)	463 (3.88)	495 (1.71)	586 (0.19)	635 sh (0.24)	683 (0.98)	737 (1.04)
**HBC(tBu)6**	343 (0.97)	359 (2.2)	389 (0.73)						
**Ref-Zn**				417 sh (0.59)	438 (4.94)	449 (3.32)	584 (0.16)	620 sh (0.19)	636 (0.58)

a sh: shoulder.

Inspection of the spectra reveals two main contributions, the HBC-centered absorption features with a major peak at 360/361 nm and two minor peaks at 346 and 391/392 nm, and the (metallo)porphyrin-centered absorption features between 400 and 800 nm. Significant differences are found relative to, for example, well-known zinc tetraphenylporphyrin (ZnTPP). **Ref-Zn** exhibits a split Soret-band absorption that is red-shifted relative to **ZnTPP** in THF. The most intense Q-band absorption is found at 636 nm with two smaller bands/shoulders at 584 and 620 nm, whereas **ZnTPP** displays two distinct Q-band absorptions at 550 and 595 nm.

Both the Soret- and Q-band absorptions in **1-Zn** are further red-shifted as well as broadened and result in an asymmetric Soret-band with a 461 nm maximum and an asymmetric Q-band with a 675 nm maximum. Similar changes are discernable for **1-Cu** and **1**, that is, red-shifted absorptions and lower number of broadened Q-bands. A major contribution to these effects stems from the presence of ethynyl groups in the *meso*-positions of the porphyrins. On the one hand, the extended π-system results in smaller band gaps and, on the other hand, the symmetry is lowered from *D*_4h_ to *D*_2h_ for all metalloporphyrins.

Additional interactions with the HBC substituents induce further red-shifts, as well as broadening and changing intensities of the spectral features. Previous work on similar di- and tetra-substituted porphyrins and porphyrin-dimers, bearing aryl-ethynyl substituents, revealed similar effects.^22,25,51–54^

The absorption spectrum of dimeric **2-Zn** also is influenced by the factors discussed above. In addition, both the Soret-band (463 and 495 nm) and the Q-band (683 and 737 nm) absorptions are split into two, suggesting electronic coupling of the two porphyrin moieties: placing two or more porphyrins in close proximity and defined geometry, as in **2-Zn**, is known to cause excitonic splitting. However, for butadiyne-linked porphyrin dimers such as **2-Zn**, the spectral changes have also been described in terms of an extended conjugated π-system across the two porphyrins and the bridge that depends on the dihedral angle between the porphyrin subunits.^36,52,53,55–59^

The same trends, *i.e.*, red-shifted (relative to **ZnTPP**) and single fluorescence maxima, were found for the (metallo)porphyrin-centered features upon 450 nm photoexcitation. Only **1-Cu** does not fluoresce because of the open-shell character of the central metal ion. HBC photoexcitation at 350 nm (Fig. 2, bottom) shows that the HBC-centered fluorescence is quenched and replaced by the (metallo)porphyrin-centered fluorescence. The underlying energy transfer occurs with quantum yields close to unity, as also found previously in similar HBC–porphyrin systems.^46–49^ Independent confirmation of the postulated energy transfer came from excitation spectra of the (metallo)porphyrin-centered fluorescence. A perfect match to the absorption spectra includes the HBC and (metallo)porphyrin fingerprints. The fluorescence quantum yields of the fluorescent **1**, **1-Zn** and **2-Zn** were evaluated by analogously using **ZnTPP** (*Φ*_Fl_ = 0.04) as fluorescence standard – Table 2.

**Table 2:** Steady state emission properties of the investigated compounds in THF^a^

Sample	Peak positions/nm							Δ*E*_opt_^b^/eV	*Φ*_Fl_
**1**	468 tr	488 tr	498 tr	705	777 sh			1.77	0.14
**1-Cu**	475 tr	506 tr	705^c^	777^c^				≈1.96^d^	—
**1-Zn**	475 tr	502 tr	677	743 sh				1.86	0.13
**2-Zn**	473 tr	504 tr	536 sh	642	688 sh	754	820 sh	1.66	0.08
**HBC(tBu)6**	463	472	482	491	515	525		2.92	0.04

a tr: trace; sh: shoulder.

b Optical band gap determined as Δ*E*_opt_ = [*E*(*λ*_max,Abs_) + *E*(*λ*_min,Em_)]/2.

c Trace amounts of a free base impurity cause fluorescence identical to **1**.

d Due to lack of fluorescence, value based on the long-wave absorption maximum at 634 nm only.

**2-Zn** fluorescence reveals, like its absorption, two maxima, at 640 and 750 nm upon either 350 nm HBC or 450 nm (metallo)porphyrin photoexcitation. Both exhibit excitation-dependent intensities and, consequently, distinctly different excitation spectra.

**Fig. 3:**
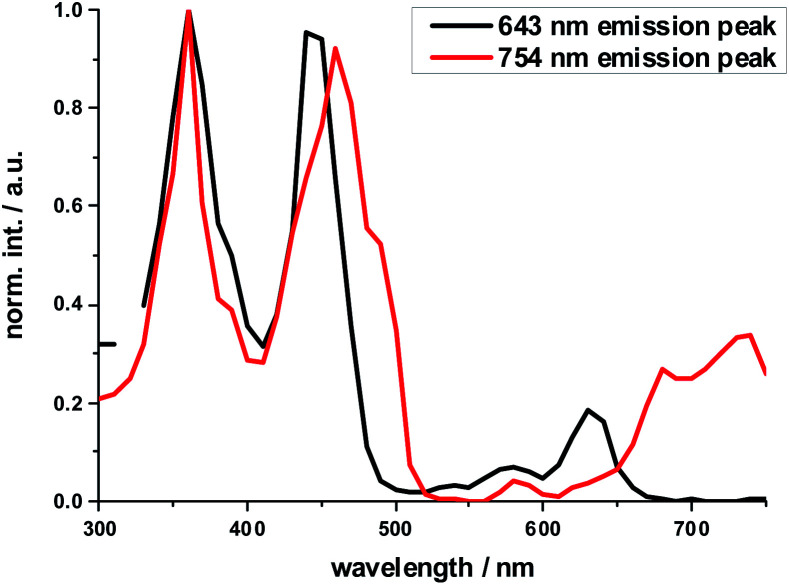
Normalized excitation spectra of the fluorescence of **2-Zn** taken at the global fluorescence maximum at 754 nm and at the local maximum at 643 nm with 10 nm increments.

The excitation spectrum taken at the 640 nm maximum is strikingly similar to the absorption spectrum of **Ref-Zn**, while that of the 750 nm maximum agrees closely with that of **2-Zn**. We rationalize these findings on the basis of two different molecular transitions, the first localized on the individual (metallo)porphyrins and the second, which is orthogonal to the first, delocalized across the porphyrin–butadiyne–porphyrin system. These distinct states correspond to the dihedral angle between the two porphyrin, that is, monomer-like states at around 90°, and delocalized states at around 0°. This is in sound agreement with the literature on similar butadiyne-linked ZnP dimer systems.^59,60^ Both fluorescent states are also populated *via* energy transfer from HBC, as inferred from contributions in the 300 to 400 nm range in both excitation spectra (Fig. 3).

**Fig. 4:**
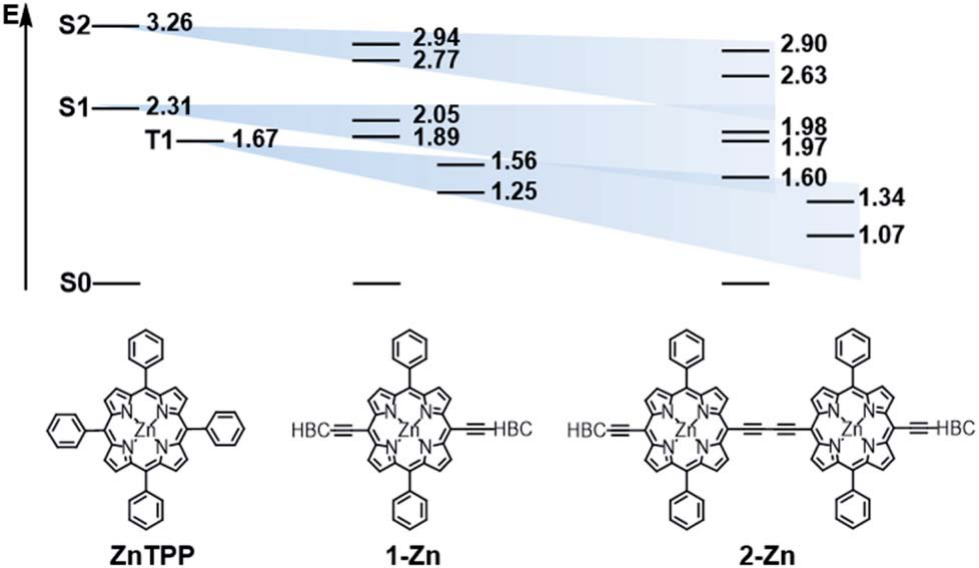
Schematic illustration of the stabilization and splitting of the porphyrin electronic states due to the introduction of ethynyl-HBC substituents in the 5,15 *meso*-positions. Numbers represent orbital energy values in eV, obtained by TD-DFT B3LYP/def2-TZVP gas phase calculations.

In order to investigate the impact of symmetry, functionalization, dimerization and coplanarity on the absorption and emission properties, we turned to molecular modeling. A series of porphyrins of different degrees of functionalization was optimized with B3LYP and CAM-B3LYP density functionals utilizing the def2-TZVP basis set. Time-dependent density functional theory (TD-DFT) was used to investigate the nature of the excited states. Fig. S38 and S39† show the S_0_ to S_1_/S_2_ Q-band vertical excitation energies and oscillator strengths thus calculated for a series of *D*_4h_ and *D*_2h_ functionalized porphyrin monomers. Functionalization in the *meso*-position leads to stabilization of the excited states, with ethynyl substituents having a larger impact than phenyl. While *D*_4h_ symmetrical **ZnTPP** shows degeneracy in the excited singlet and triplet states, the *D*_2h_ symmetrical compounds, such as **ZnDPP** (5,15-diphenyl-Zn^II^) and **ZnDPP(C2H)2** (5,15-diethinyl-10,20-diphenylporphyrinato-ZnII), which are for symmetry reasons a good comparison to **1-Zn**, show an increased splitting in the order **ZnDPP** < **ZnDPP(C2H)2** < **1-Zn**. These significant changes in excited-state energy levels are accompanied by a significant rise of the *Q*_*x*_ (S_1_) oscillator strength, while *Q*_*y*_ (S_1_′) remains almost unchanged. The predicted S_0_–S_1_ energies are close to the experimentally observed *E*_00_ transitions with 1.89–1.97 eV for **1-Zn** (experiment 1.88 eV) and 1.60–1.77 for **2-Zn** (experiment 1.69). Soret band transitions are affected in a similar manner, decreasing in energy with extension of the π-system. However, the changes in oscillator strength are not as pronounced as in the Q-band transition (Fig. S40 and S41†). The two lowest triplet-excited states are stabilized similarly to the lowest singlet excited state under loss of degeneracy. These trends are continued for the porphyrin dimer **2-Zn** and corresponding model compounds **2-ZnDPP** and **2-ZnDPP(C2H)2** (Fig. S42–S45†). In contrast to the monomer species, additional excited states are found between Soret- and Q-band (Tables S7–S9 and Fig. S47–S51†). The excited states are stabilized by 0.1–0.2 eV because of functionalization. Coplanarity of the porphyrin subunits affects the singlet excited state (Fig. S46†) most strongly.^49^ Coplanarity has several effects: the S_1_ state is stabilized by 0.7 eV and the S_0_–S_1_ oscillator strength increases; the two Soret band transitions are further split and transitions with significant oscillator strength evolve between the Soret- and Q-band regimes. The results obtained are summarized in simplified form in Fig. 4, which demonstrates the effects of symmetry loss and stabilization, respectively, due to functionalization.

### Dimer coordination experiments

As the exciton coupling strength depends on the dihedral angle between the two porphyrins in **2-Zn**, maximum electronic communication is enabled in the coplanar conformation.^57,59–61^ In various ZnP–ZnP dimers, bidentate coordination with diamines has been shown to enhance the exciton splitting.^62^

Therefore, we probed **2-Zn** with the following diamines of varying chain length; 1,7-diaminoheptane (**1,7-DA**), 1,10-diaminodecane (**1,10-DA**), and 1,12-diaminododecane (**1,12-DA**). In addition, we used a monoamine as reference: heptylamine (**HA**) (see ESI, Fig. S23 and S24† for steady state absorption and emission spectra).

**Fig. 5:**
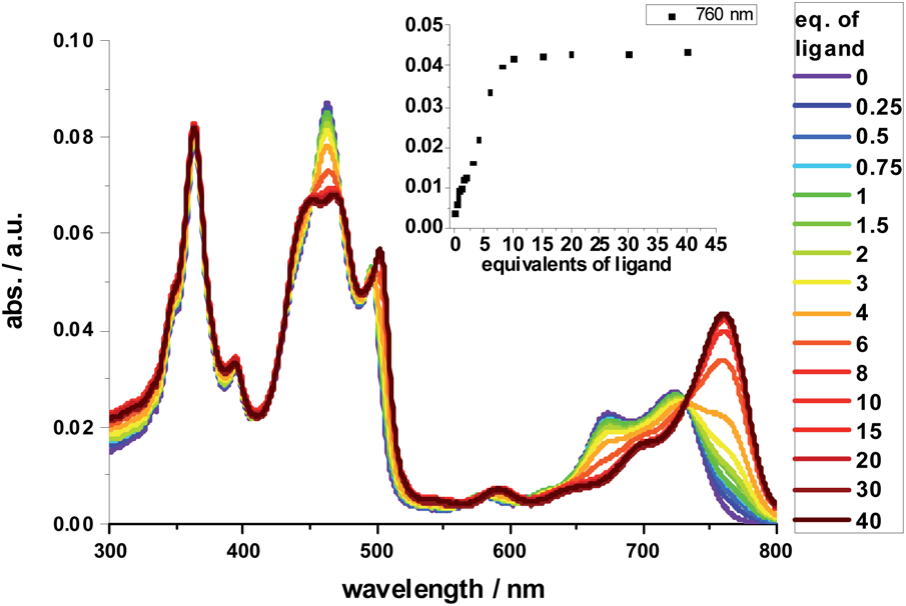
Spectral changes upon addition of increasing amounts of **1,10-DA** (0–1.0 × 10^−5^ M) to **2-Zn** (2.5 × 10^−7^ M) in toluene. Inset: evolution of the 760 nm absorption band, indicating the **2-Zn·1,10-DA** complex formation – please note that the *y*-axis labeling is identical in both presentations.

**Fig. 6:**
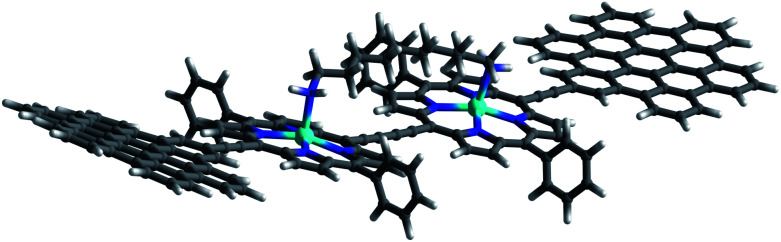
B3LYP/def2-TZVP optimized lowest energy conformer of **2-Zn·1,10-DA**.

Complexation with **1,10-DA** yields the most pronounced spectral changes in the **2-Zn** titration experiments – Fig. 5. In particular, in the range of the Q-band absorptions, that is, between 550 and 800 nm, the initial maxima at 580, 632 (shoulder), 674, and 724 nm, shift to 591, 645 (shoulder), 700, and 761 nm, respectively. Hereby, the intensity of the long-wavelength maximum increases strongly, while those at 674 and 724 nm decrease in intensity. In the 400 to 550 nm Soret-band range, new transitions evolve at 452 and 502 nm. Isosbestic points develop at 451, 473, 492, 497, and 731 nm. Importantly, the HBC-centered absorptions do not change throughout the titration assays. Our observations are consistent with excitonic splitting and indicate a coplanar or nearly coplanar arrangement between the two ZnPs in **2-Zn·1,10DA**. B3LYP/def2-TZVP optimization of **2-Zn·1,10DA** yields the same results – Fig. 6. Interestingly, for **2-Zn·1,7DA** even at 30 equivalents the equilibrium is not reached. In contrast, the equilibrium is completed for **1,12DA** at 2 equivalents (Fig. S23†). An immediate consequence is a higher binding energy for **1,12DA** and, in turn, a higher association constant.

Red-shifts were also found when **HA** or pyridine was added to either **1-Zn** or **2-Zn**, affording binding constants of 4 × 10^5^ M^−1^ for **1-Zn·HA** and 7.9 × 10^5^ M^−1^ for **2-Zn·HA** – Fig. S23.† The Q-band absorptions shift in any of these cases by less than 25 nm and without any major changes in intensity. No appreciable changes are found in the Soret-band absorptions. Titrations with **1,7-DA** or **1,12-DA** are accompanied by similar, although less pronounced, changes in the Soret- and Q-band range. Based on the concentration dependence and the 760 nm intensity in combination with the aforementioned we conclude that **1,7-DA** is too short and **1,12-DA** is too long to induce any coplanar conformation. This conclusion is in sound agreement with the modeling. Due to chelating effects and the best flexibility, **1,12-DA** features the highest binding constant of 1.3 × 10^8^ M^−1^, which renders it ideal for supramolecular assays – *vide infra*.

Fluorescence spectra mirror the changes seen in the absorption spectra (ESI, Fig. S24†). In particular, the short-wavelength fluorescence maxima of **2-Zn** shift upon their excitation along with the longest-wavelength absorption features without, however, affecting the Stokes shifts. Isosbestic points develop around 760 nm for **1,7-DA**, **1,10-DA**, and **1,12-DA**, while reference titrations with **HA** lacked clear isosbestic points. Coordination in reference experiments with **HA** and **1-Zn** results in red shifts as large as 20 nm. Notably, addition of **1,10-DA** to **2-Zn** red-shifts the maximum relative to **2-Zn·HA** by another 15 nm to a total of 35 nm due to coplanarization of the two ZnPs and excitonic splitting. Overall, the optical band-gap is decreased by as much as 80 meV when comparing **2-Zn** in the absence and in the presence of **1,10-DA** – Table 3. Fitting the underlying binding isotherm of the fluorescence experiments affords a binding constant of 1.7 × 10^6^ M^−1^ for **2-Zn·1,10DA** – Fig. S24.†

**Table 3:** Influence of various coordinating amines in toluene on Stokes shift and optical bandgap

Sample	Stokes shift/eV	*E*_0–0_/eV
**1-Zn**	0.057	1.88
**1-Zn·HA**	0.054	1.83
**2-Zn**	0.030	1.69
**2-Zn·1,7-DA**	0.011	1.64
**2-Zn·HA**	0.032	1.63
**2-Zn·1,12-DA**	0.028	1.62
**2-Zn·1,10-DA**	0.027	1.62

**Fig. 7:**
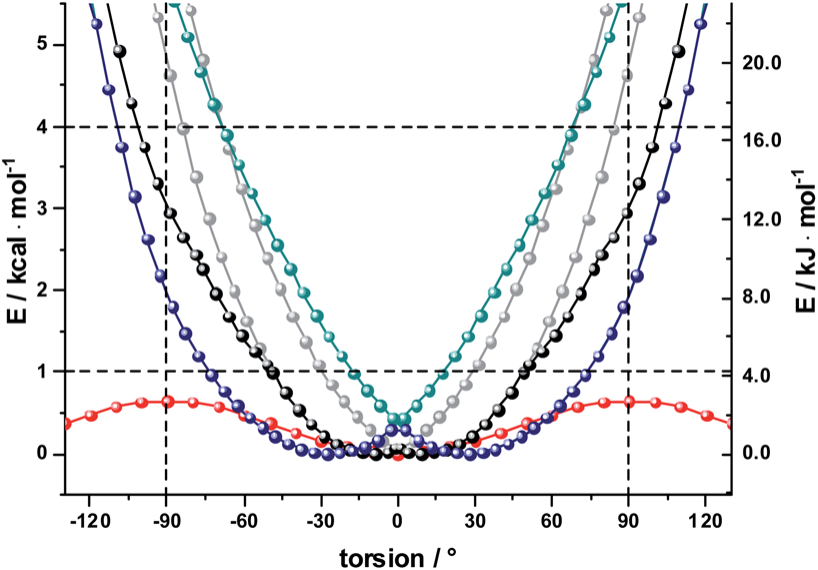
B3LYP/def2-SVP calculations gas phase relaxed potential energy scans for **2-ZnPDPP** (red), complexed with **1,10 DA** (grey – two global minima conformers, black – lowest energy structure, allowing rotation around C–C bonds in DA) and **1,12 DA** (cyan – two global minima conformers, blue – lowest energy structure, allowing rotation around C–C bonds in DA).

As a complement, the influence of complexation with the different mono- and diamines was investigated by molecular modeling. Relaxed torsion scans, in which the angle between the two porphyrin planes was varied, yield rotation barriers of 0.6 kcal mol^−1^ (2.5 kJ mol^−1^) independent of further functionalization on the outer periphery (Fig. S29†), in good agreement with existing literature.^59,61^ We next studied axial amine coordination to the Zn center with a reference system (ZnP·methylamine) in the gas phase (Fig. S30 & Table S1†). The binding energy and the length of the Zn–N bond depend strongly on the size of the basis set, suggesting significant basis set superposition error (BSSE) for def2-SVP, while the triple ζ def2-TZVP gives results within 1 kcal mol^−1^ of the largest basis set used. Furthermore, including empirical dispersion corrections and using range-separated functionals increases the binding energy significantly. Our estimate of the binding energy for a monodentate coordination lies in the range of 10–18 kcal mol^−1^. This was confirmed when studying the force-field annealed and DFT-optimized structures of **2-ZnDPP** with all three diamines (Fig. S31, Tables S2 and S3†). The binding energies for bidentate complex formation are always less than twice the monodentate binding energy because of reorganization effects within the diamines and porphyrin dimer. However, the chelating nature enables binding energies higher than 16 kcal mol^−1^ for **1,10-DA** and **1,12-DA** in the gas phase, which is 6 kcal mol^−1^ better stabilized than the monodentate coordination of **1,7-DA**. Including solvation in toluene and THF with the PCM model reduces the binding energy to 13 and 10 kcal mol^−1^, respectively (Fig. S32†). As this is 14 times higher than the torsional barrier observed for **2-Zn**, the hybrid geometry is determined by the binding motif and the length of the DA aliphatic chain. **2-Zn·1,10-DA** exhibits both the conformation with the smallest dihedral angle between the ZnP subunits (8° B3LYP/TZVP, 0° with dispersion correction) and the largest rotation barrier. The minimum-energy conformation for **2-Zn·1,12-DA** lies close to 28° (72° with dispersion correction included) ZnP–ZnP torsional angle because the alkyl chain is longer than the Zn–Zn distance (Fig. 7, S34–S36 and Table S5†). Torsional barriers rise steeply on bidentate complexation with the diamines; from 0.6 kcal mol^−1^ for **2-Zn** to 2.0 or 4.8 kcal mol^−1^ for complexes with **1,12-DA** and **1,10-DA**, respectively. **1,7-DA** cannot coordinate to **2-Zn** in a bidentate fashion without severe bending of the ZnP–butadiyne–ZnP scaffold (45° out of plane) and is, therefore, likely to bind to only one of the two ZnPs.

The pronounced geometrical changes on inclusion of dispersion corrections have two main reasons; the low torsional barrier of 0.6 kcal mol^−1^ of the **2-Zn** dimer and the attractive dispersive interaction of the aliphatic backbone of the DA with the butadiyne bridge. Benchmark calculations (Fig. S36 and Table S5†) show that all methods without dispersion correction underestimate this interaction significantly, while empirical dispersion corrections overestimate it. Secondly, the σ–π interaction of −0.66 kcal mol^−1^ for the two possible binding motifs (CCSD(T) reference calculation) is only 0.06 kcal mol^−1^ higher than the torsional barrier, so that slight deviations from this reference value lead to large geometrical changes.

Finally, we used AM1 semiempirical direct molecular-dynamics simulations to study the movement around the Zn–Zn bond for **2-Zn** and its complexes with **1,10-DA** and **1,12-DA** (Fig. S37†). Both diamines block the rotation efficiently, restricting the oscillations to the range of −90° to 90° at 150 K and 300 K after 100 ps equilibration time. The harmonic oscillator model gives a 50% increased stiffness for **2-Zn·1,10-DA** compared to **2-Zn·1,12-DA** (Table S6†). This is further corroborated by the increased width of the Gaussian fit in the statistical analysis.

The results let us conclude that **1,10-DA** is best suited for stabilizing **2-Zn** in a planar arrangement, while the backbone of **1,7-DA** is too short to chelate **2-Zn**. In contrast, **1,12-DA** is too long for an efficient planarization, although it leads to the best stabilization – in line with the titration experiments (ESI, Fig. S23 and S24†).

**Fig. 8:**
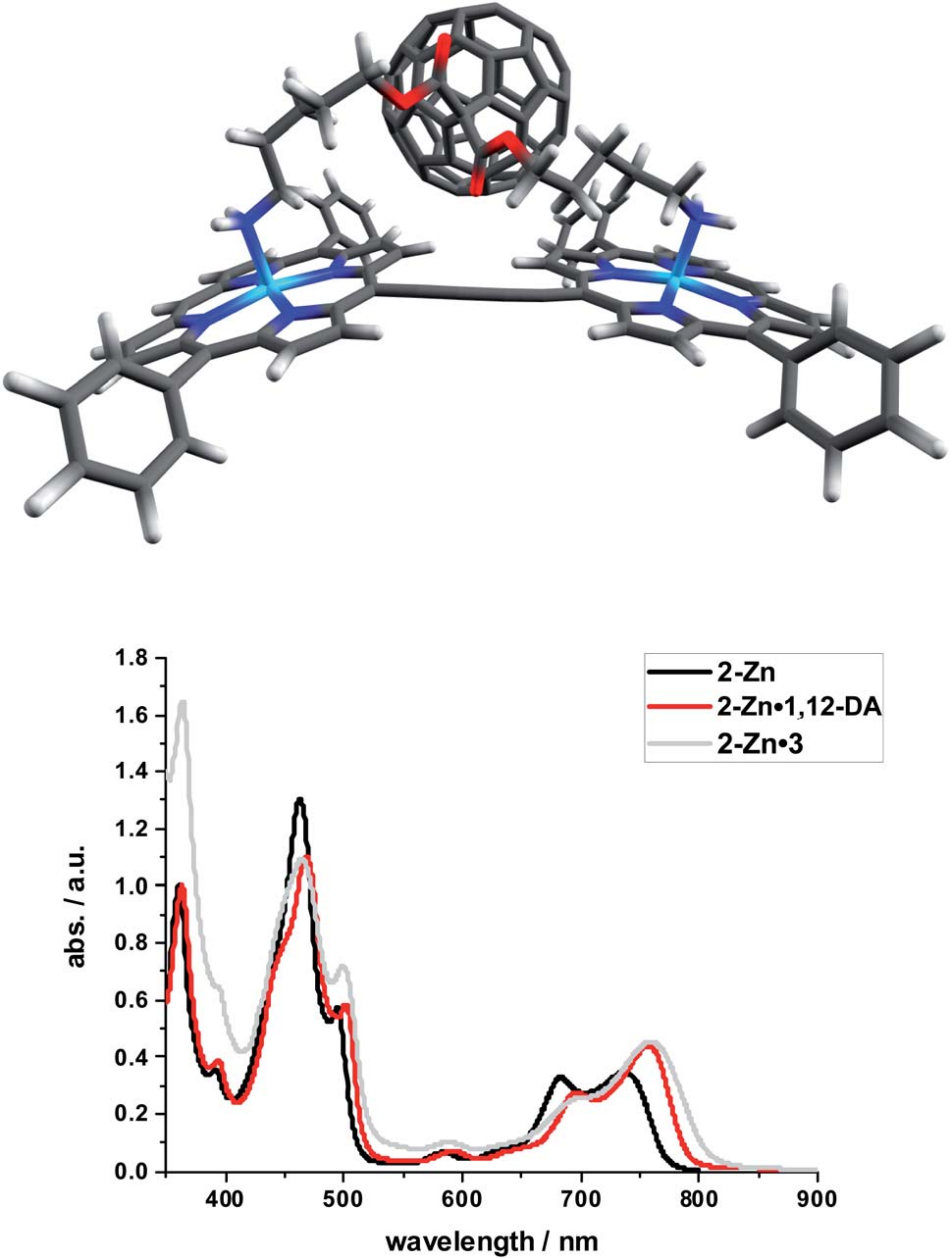
Top: Optimized structure of the **2-Zn**·**3** complex (HBC and silyl substituents omitted); Bottom: Comparison of the spectral changes induced by complexation of **2-Zn** with **1,12-DA** and **3**.

Binding constants on the order of 10^8^ M^−1^ encouraged us to test C_60_-derivative **3** bearing two pentylamine side chains to coordinate to **2-Zn** and to afford electron donor–acceptor **2-Zn**·**3** with near panchromatic absorptions from the UV to the NIR. We opted for the strongest binding rather than the highest degree of coplanarity. The rather poor solubility of **3** necessitated partial protonation by adding 0.5 eq. of trifluoroacetic acid.^63^ Importantly, the absorption spectrum of **2-Zn**·**3** is a good match of that found for **2-Zn·1,12-DA** – Fig. 8. Next, **2-Zn**·**3** was probed by time-resolved transient absorption spectroscopy.

### Time-resolved transient absorption spectroscopy

The excited state properties of the HBC–(metallo)porphyrins **1**, **1-Zn**, **1-Cu**, and **2-Zn** were probed in pump-probe experiments with ultrashort 387 or 450 nm laser pulses in the absence of molecular oxygen. Additional experiments were performed with 676 nm excitation for **1-Zn**, with 505, 676, and 775 nm excitations for **2-Zn**, and 775 nm excitation for **2-Zn**·**3**.

**Fig. 9:**
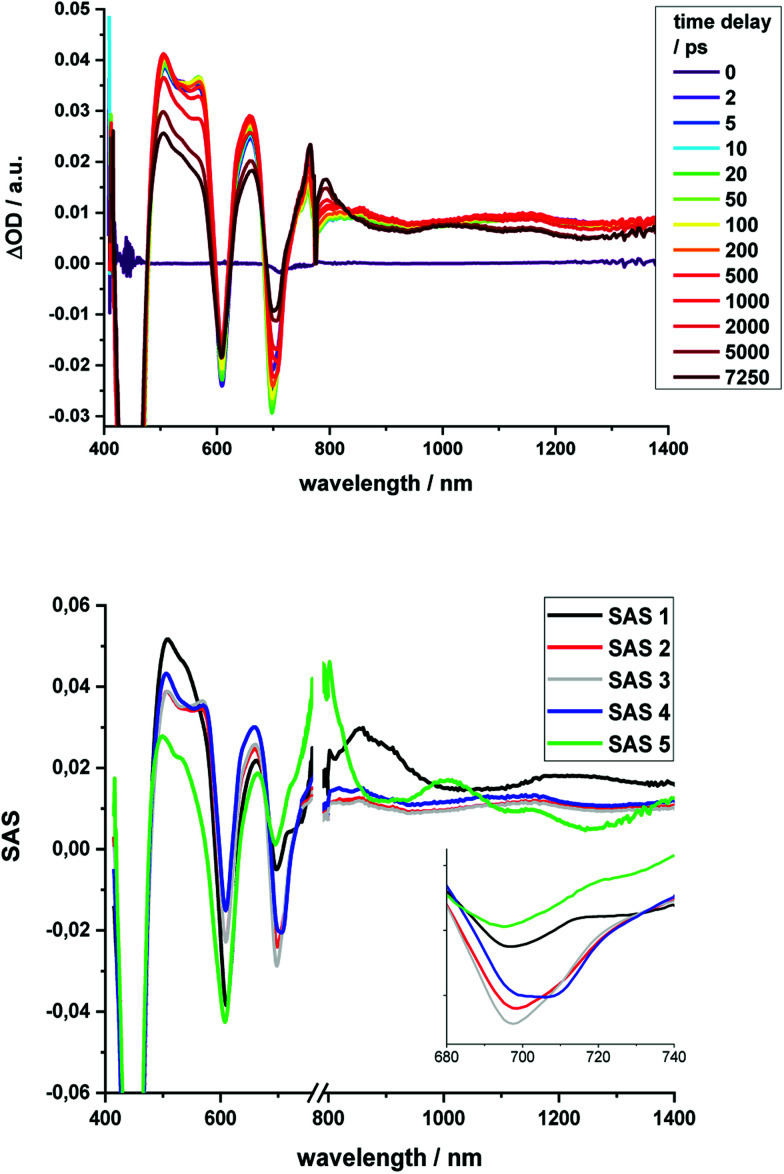
Top: Differential absorption spectra (visible and NIR) of **1** obtained upon excitation with 387 nm pulses with time delays from 0 ps to 7.25 ns after excitation in THF; Bottom: Deconvoluted species associated spectra derived from target analysis *via* GloTarAn with lifetimes of 0.35 ps (SAS1) 12 ps (SAS2), 254 ps (SAS3), 7.7 ns (SAS4), and >100μs (SAS5).

The excited state deactivation of all HBC–porphyrin-conjugates presented is extremely complex in comparison to (metallated) TPP derivatives. The splitting of states induced by the lowered symmetry and additional conjugation with the ethynyl-HBC-substituents, as discussed above, introduces additional energy levels/states (Fig. 4), which are transiently populated en route to the ground state. Detailed descriptions of the time constants of these states and their differential absorption features can be found in the ESI, Fig. S1–S21.† For the monomeric HBC–porphyrin-conjugates **1**, **1-Cu**, and **1-Zn**, Soret-band excitation gives rise to deactivation through five states, which we assign as four singlet excited states and the respective lowest triplet excited state. An example of transient spectra and the species associated spectra of the states involved in the excited state deactivation is given for **1** in Fig. 9. **2-Zn** deactivates *via* five singlet excited states and its lowest triplet excited state – compare Fig. 4. Choosing a significantly lower excitation energy leads to the population of fewer states in the excited state deactivation (ESI, Fig. S14–S21†).

**Fig. 10:**
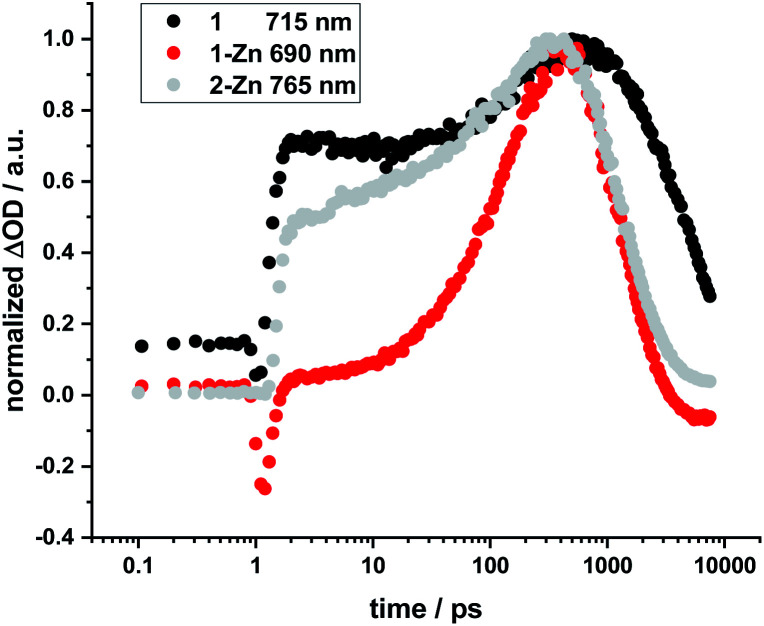
Normalized time profiles taken from transient absorption measurements of **1**, **1-Zn**, and **2-Zn**, showing the evolution of the respective fluorescence signals as a function of time.

No HBC-centered features, namely singlet excited-state maxima at 560 and 620 nm as well as triplet excited-state maxima at 500 and 540 nm, were found for any of the conjugates on either 387 nm HBC or 450 nm (metallo)porphyrin excitation (compare ESI, Fig. S22†). We infer a unidirectional HBC to (metallo)porphyrin energy transfer faster than the time resolution of our experimental set-up from the steady-state fluorescence experiments, giving a lower rate limit of >10^12^ s^−1^. Interestingly, in the free-base compound **1**, the Zn-monomer **1-Zn** and the Zn-dimer **2-Zn** systems we observe a marked delay of the stimulated fluorescence features (Fig. 10). The fluorescence signals reach their maximum intensity after 600 ps for **1**, and approximately 400 ps for **1-Zn** and **2-Zn**. Transient maxima in the NIR region that exhibit the same formation kinetics are found in all cases (also for **1-Cu**). These findings suggest a cascade of deactivation through the split excited states identified by molecular modeling (see above), before populating the lowest, emissive singlet excited state.

For butadiyne-linked ZnP dimers, whose structures resemble **2-Zn**, it has been demonstrated that intramolecular rotation of the porphyrins relative to the butadiyne axis occurs at room temperature (Δ*G*_298 K_ ≈ 2 kJ mol^−1^),^61^ so that a broad distribution of porphyrin–porphyrin dihedral angles is present. Overall, the room-temperature rotation has been linked both to a rise in fluorescence with a time constant of approximately 100 ps and to changes in transient absorption with approximately 200 ps.^60^ Variations in temperature,^59–61^ solvent viscosity,^60^ and excitation wavelength^60^ affect the distribution of the dimer conformations (perpendicular or coplanar). Not only the **2-Zn** dimer displays delayed formation of fluorescence and related transient absorption features, but also **1**, **1-Cu**, and **1-Zn** monomers. For example, marked rise times in fluorescence and shifts in the transient features are linked to excitation dependence. Changes in **1**, **1-Cu**, and **1-Zn** monomers are, however, attributed to the electronic structure of the ethynyl-extended porphyrin cores and their reduced symmetry/increased number of energetically non-degenerate states.^22,64^

**Fig. 11:**
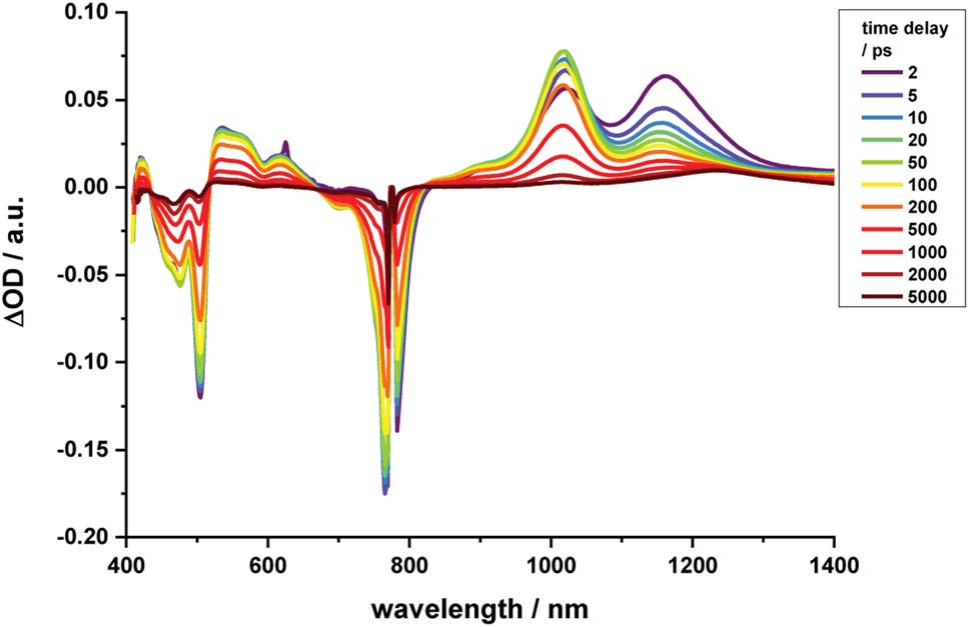
Time-resolved transient absorption spectra of **2-Zn**·**3** in chlorobenzene with several time delays between 0 and 5000 ps; excitation with 775 nm pulses.

Regarding **2-Zn**·**3**, 775 nm pulses give rise to unambiguous evidence for the **2-Zn**-centered excited-state formation, from which charge separation and charge recombination affords the intermediate **2-Zn˙+**·**3˙−** charge-separated state – Fig. 11. For example, short laser pulses generate the excited state of **2-Zn** in the form of ground state bleaching of the Soret- and Q-band absorptions in the 400 to 500 nm and 700 to 800 nm ranges, respectively. The latter are accompanied by a maximum in the range between 1100 to 1300 nm. A number of differences are discernable relative to pristine **2-Zn**: first, in the 400 to 500 nm range, the most intense bleaching evolves at 505 nm, accompanied by minor features at 475 and 455 nm, rather than at 465 nm, which is followed by a weaker 500 nm minimum. Second, a set of two similarly strong minima at 685 and 740 nm is replaced by a single minimum at 765 nm. Third, the 1145 nm maximum is red-shifted to 1160 nm. The presence of C_60_ in **2-Zn**·**3** induces a charge separation from the excited state of **2-Zn** rather than the intrinsic decay seen in the experiments without C_60_ – Fig. S15–S21.† Evidence for the charge separation stems from the NIR fingerprint absorptions of the one-electron reduced form of C_60_ and the one electron-oxidized form of **2-Zn**. One-electron oxidized butadiyne-bridged ZnP-dimers such as **2-Zn** are known to exhibit intense NIR absorption bands,^65^ with an intense maximum around 1000 nm, whereas the one-electron reduced form of C_60_ exhibits a characteristic absorption around 1020 nm.^66^ The newly developing feature around 1000 nm is a superimposition of contributions from **2-Zn˙+** and C_60_˙^−^. It is important that, within the context of charge separation, the growth at 1020 nm occurs simultaneously with the decay at, for example, 1160 nm (Fig. S25†). Important is the lack of charge separation in reference experiments with **2-Zn·1,10-DA** and **2-Zn·1,12-DA** – Fig. S26† – due to the absence of electron accepting fullerenes. On a longer timescale, the triplet excited state of **2-Zn** persists. Most notable is the 1240 nm maximum, which indicates a significant red-shift relative to the 1210 nm maximum found for **2-Zn** in the absence of C_60_. The triplet excited state of C_60_ is, however, not populated en-route towards ground state recovery. We hypothesize that the red-shifts seen in the absorption spectra of **2-Zn** relative to **Ref-Zn** places its triplet excited state energy below that of C_60_-derivative **3**. Such an inactive participation seems reasonable, considering that the involvement of either C_60_- or porphyrin centered triplet excited states in charge recombination processes depends on their energy relative to the charge separated state.^67–69^

**Fig. 12:**
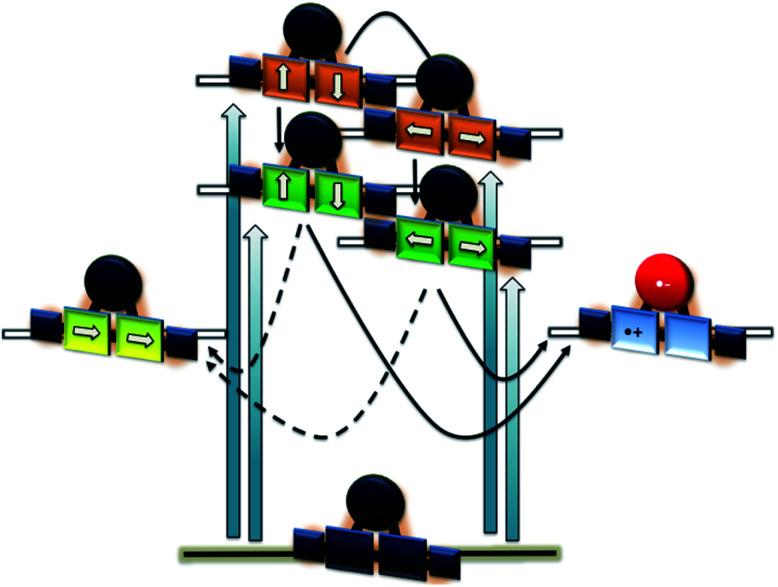
Energy diagram to illustrate the deactivation of **2-Zn**·**3** upon 450 and 775 nm photoexcitation on the left and right hand side, respectively. Orange, green, and yellow reflect the second singlet excited state, the first singlet excited state, and the triplet excited state, while blue/red the charge-separated state.

Similar to Fig. 12, a global fit of the transient absorption data of **2-Zn**·**3** in chlorobenzene yields good results using five species (ESI, Fig. S25†). In accordance with the spectral changes described above, the first and second species, with lifetimes of 0.4 and 8 ps, represent the split first singlet excited state of **2-Zn** – *vide supra*. Both undergo charge separation to afford the one-electron reduced form of C_60_ and the one-electron oxidized form of **2-Zn**. The latter, however, is seen as the third and fourth species. Notably, the flexibility of the linkers that connect **2-Zn** with C_60_ is likely to lead to a distribution of center-to-center distances between the electron donor and acceptor. We interpret the corresponding lifetimes as upper and lower limits for the distribution of lifetimes with values of 380 and 950 ps. The fifth species is the triplet excited state of **2-Zn**. It is populated from the second species and can be considered the intrinsic intersystem crossing, which competes with the charge separation. Its overall quantum yield is only about 10%.

This constitutes an intriguing electron donor–acceptor system, which exhibits absorption exploitable to drive a charge separation from the UV (∼350 nm) to the NIR (∼800 nm).^70–72^ The charge-separated state formed accordingly is generated with high efficiency and lives up to 1 ns before relaxing to the ground state.

## Conclusion

In a series of novel ethynyl-bridged HBC–porphyrin conjugates, we have chemically linked HBCs to porphyrins and have investigated their photophysical properties by means of steady-state and time-resolved spectroscopic methods. In interplay with molecular modeling, a firm basis for the interpretation of the ground- and excited-state properties of these systems is found. Particular focus was placed on the tuning of the excitonic couplings. Starting with the porphyrin core, adding just bare ethynyl groups leads to appreciable changes in the absorption and fluorescence across the visible range. Next, linking HBCs enables an expansion of the resulting absorption to the UV and, in turn, is the basis for a unidirectional and unit efficient HBC-to-porphyrin energy transfer. Additional control over the absorptive and emissive features is realized by complexing bidentate ligands including a bis(aminoalkyl)-substituted fullerene to the porphyrin dimer with binding constants in the range from 10^6^ to 10^8^ M^−1^. An immediate consequence is a panchromatic absorption reaching from around 350 nm in the UV to 800 nm in the near infrared and beyond. A suitable ligand length facilitates both the locking of the dihedral angle between the two porphyrins at close to 0° and a photosynthetic sequence of HBC-to-porphyrin energy transfer and porphyrin-to-C_60_ charge transfer.

## Conflicts of interest

There are no conflicts to declare.

## Supplementary Material

SC-011-D0SC02028A-s001
