# Supplementary material for: Panchromatic light funneling through the synergy in hexabenzocoronene–(metallo)porphyrin–fullerene assemblies to realize the separation of charges
Source: Chem Sci. 2020 Jun 17;11(27):7123–32. doi: 10.1039/d0sc02028a (PMC8159381; doi:10.1039/d0sc02028a)
Supplement: SC-011-D0SC02028A-s001 [file SC-011-D0SC02028A-s001.pdf]

Electronic Supplementary Material (ESI) for Chemical Science.
This journal is © The Royal Society of Chemistry 2020

Panchromatic Absorption through Chemically and Excitonically Coupled Hexabenzocoronene–(Metallo)porphyrin–Fullerene Assemblies

Maximilian Wolf, Dominik Lungerich, Stefan Bauroth, Maximilian Popp, Benedikt Platzer, Timothy Clark, Harry L. Anderson, Norbert Jux, and Dirk M. Guldi

Table of contents

1. Synthetic section ... 4

Scheme S1 ... 5

Dipyrromethane **S1** ... 6

3,5-Bis(trihexylsilyl)benzaldehyde **S2** ... 7

5-15-Bis(3,5-di(trihexylsilyl)phenyl)porphyrin **S3** ... 8

5-15-Bis(3,5-di(trihexylsilyl)phenyl)porphyrinato-ZnII **S4** ... 9

5-15-Dibromo-10,20-bis(3,5-di(trihexylsilyl)phenyl)porphyrinato-ZnII **S5** ... 10

5-15-Dibromo-10,20-bis(3,5-di(trihexylsilyl)phenyl)porphyrin **S6** ... 11

5-15-Dibromo-10,20-bis(3,5-di(trihexylsilyl)phenyl)porphyrinato-CuII **S7** ... 12

Bis-(HBC-ethynyl)porphyrin **1** ... 13

Bis-(HBC-ethynyl)porphyrinato-Zn^{II} **1-Zn** ... 14

Bis-(HBC-ethynyl)porphyrinato-Cu^{II} **1-Cu** ... 15

Mono-(HBC-ethynyl)porphyrinato-ZnII **S11** ... 16

Bis-HBC-porphyrin dimer **2-Zn** ... 17

2,3,4,5-Tetrakis(4-*tert*-butylphenyl)cyclopenta-2,4-diene-1-one **S13** ... 18

4-Bromo-4'-*t*-butyltolan **S14** ... 19

4-Bromo-4',4'',4''',4'''',4'''''-penta(*tert*-butyl)hexaphenylbenzene **S15** ... 20

2-Bromo-5,8,11,14,17-penta(*tert*-butyl)hexa-peri-hexabenzocoronene **S16** ... 21

2-(trihexylsilylethynyl)-5,8,11,14,17-penta(*tert*-butyl)hexa-peri-hexabenzocoronene **S17** ... 22

2-(Ethynyl)-5,8,11,14,17-penta(*tert*-butyl)hexa-peri-hexabenzocoronene **S18** ... 23

5-((*tert*-Butoxycarbonyl)amino)pentyl methyl malonate **S19** ... 24

5-((*tert*-Butoxycarbonyl)amino)pentyl methyl malonyl –[1,0]–mono–1,2,–dihydro[60]–fullerene **S20** ... 25

5-Aminopentyl methyl malonyl –[1,0]–mono–1,2,–dihydro[60]–fullerene **S21** ... 26

Bis(5-((*tert*-butoxycarbonyl)amino)pentyl) malonate **S22** ... 27

Bis(5-((*tert*-butoxycarbonyl)amino)pentyl) malonyl –[1,0]–mono–1,2,–dihydro[60]–fullerene **S23** ... 28

Bis(5-aminopentyl) malonyl –[1,0]–mono–1,2,–dihydro[60]–fullerene **3** ... 29

2. Spectroscopic supplementary information ... 30

Figure S1: fsTAS analysis of **1** upon 387 nm excitation ... 31

Figure S2: nsTAS analysis of **1** upon 387 nm excitation....32
Figure S3: fsTAS analysis of **1** upon 450 nm excitation....33
Figure S4: nsTAS analysis of **1** upon 450 nm excitation....34
Figure S5: fsTAS analysis of **1-Zn** upon 387 nm excitation....35
Figure S6: nsTAS analysis of **1-Zn** upon 387 nm excitation....36
Figure S7: fsTAS analysis of **1-Zn** upon 450 nm excitation....37
Figure S8: nsTAS analysis of **1-Zn** upon 450 nm excitation....38
Figure S9: fsTAS analysis of **1-Zn** upon 676 nm excitation....39
Figure S10: fsTAS analysis of **1-Cu** upon 387 nm excitation....40
Figure S11: nsTAS analysis of **1-Cu** upon 387 nm excitation....41
Figure S12: fsTAS analysis of **1-Cu** upon 450 nm excitation....42
Figure S13: nsTAS analysis of **1-Cu** upon 450 nm excitation....43
Figure S14: fsTAS analysis of **2-Zn** upon 387 nm excitation....44
Figure S15: nsTAS analysis of **2-Zn** upon 387 nm excitation....45
Figure S16: fsTAS analysis of **2-Zn** upon 450 nm excitation....46
Figure S17: nsTAS analysis of **2-Zn** upon 450 nm excitation....47
Figure S18: fsTAS analysis of **2-Zn** upon 505 nm excitation....48
Figure S19: nsTAS analysis of **2-Zn** upon 505 nm excitation....49
Figure S20: fsTAS analysis of **2-Zn** upon 676 nm excitation....50
Figure S21: fsTAS analysis of **2-Zn** upon 775 nm excitation....51
Figure S22: Comparison of selected time traces....52
Figure S23: Steady state absorption spectra of supramolecular titration experiments....53
Figure S24: Steady state emission spectra of supramolecular titration experiments....54
Figure S25: fsTAS analysis of **2-Zn** + **3** upon 775 nm excitation....55
Figure S26: Reference fs-TAS measurements....56
3. Molecular Modelling....57
Figure S27: B3LYP/def2-TZVP optimized geometries of **2-Zn** and **1-Zn** in the gas phase....58
Figure S28: B3LYP/def2-TZVP optimized geometries of porphyrin monomers and dimers used for potential energy scans and TD-DFT calculations....59
Figure S29: Relaxed potential energy scan of **2-ZnDPP**....60
Figure S30 and Table S1: Benchmark of Zn-N interaction energy of a reference structure (B)....61
Figure S31: B3LYP/TZVP Optimized geometries of **2-ZnDPP(C_2H)$_2$** upon complexation....62
Table S2: Binding energy for complex formed of **2-ZnDPP** with **1,12** – **1,10** and **1,7 – DA**....63
Table S3: Binding energy derived by different levels of theory....64
Figure S32: Solvent dependence of the binding energy for complexation of **ZnDPP**....65
Figure S33: Relaxed potential energy scan of **2-ZnDPP**....66

Table S4: Width and activation barrier upon Zn-Zn torsion for **2-Zn** + **1,12 –DA** and **1,10 –DA**. 66
Figure S34: top: Relaxed potential energy scan of **1,10 DA** 67
Figure S35: Relaxed potential energy scan of **2-ZnDPP** complexated with **1,12 DA** 68
Figure S36 and Table S5: Benchmark of Dispersion interaction. 69
Figure S37: Zn-Zn torsion evolution 70
Table S6: Oscillation frequency 71
Figure S38: B3LYP/def2-TZVP calculated vertical excitation energies and oscillator strengths of monomers. 72
Figure S39: CAM-B3LYP/def2-TZVP calculated vertical excitation energies and oscillator strengths of monomers. 73
Figure S40: B3LYP calculated vertical excitation energies and oscillator strengths of monomers....74
Figure S41: CAM-B3LYP calculated vertical excitation energies and oscillator strengths of monomers. 75
Figure S42: B3LYP calculated vertical excitation energies and oscillator strengths of dimers. 76
Figure S43: B3LYP calculated vertical excitation energies and oscillator strengths of dimers. 77
Figure S44: CAM-B3LYP calculated vertical excitation energies and oscillator strengths of dimers .78
Figure S45: CAM-B3LYP calculated vertical excitation energies and oscillator strengths of dimers. 79
Figure S46: B3LYP/def2-TZVP predicted vertical excitations for various conformations of **2-ZnDPP(C_2H)$_2$** 80
Table S7: Vertical excitations and oscillator strengths calculated with B3LYP/def2-TZVP in the gas phase. 81
Table S8: Vertical excitations and oscillator strengths calculated with CAM-B3LYP/def2-TZVP in the gas phase 82
Table S9: Vertical excitations and oscillator strengths calculated with AM1 in the gas phase. 83
Figure S47: Natural transition orbitals for the Q- and Soret band transition of **ZnP** 84
Figure S48: Natural transition orbitals for the Q- and Soret band transition of **ZnDPP** 85
Figure S49: Natural transition orbitals for the Q- and Soret band transition of **ZnDPP(C_2H)$_2$** 86
Figure S50: Natural transition orbitals for the Q- band transition of **1-Zn** 87
Figure S51: Natural transition orbitals for the Soret- band transition of **1-Zn** 89
Figure S52: Vertical energies predicted with B3LYP/TZVP//B3LYP/TZVP of **1-Zn**, **2-Zn**, and **ZnTPP**. 90
4. Spectral Appendix (NMR, MS) 92-145

1. Synthetic section

General experimental information

All chemicals were purchased from Sigma-Aldrich and used without any further purification. HPLC-grade solvents were purchased from Sigma-Aldrich. Reactions under inert conditions were carried out using standard Schlenk techniques. Unless otherwise stated, solvents and reaction mixtures were degassed by three cycles of vacuum (1 min)/argon purge under sonication. RBF stands for round bottom flask. Thin layer chromatography was performed on Merck silica gel 60 F254, detected by UV-light (254 nm, 366 nm). Column chromatography (LC) and flash column chromatography were performed on Macherey–Nagel silica gel 60 M (230–400 mesh, 0.04–0.063 mm). Size exclusion chromatography (SEC) was carried out on SX1 biobeeds. MALDI mass spectrometry was carried out on Waters MALDI Micro MX, with DCTB, (*trans*-2-[3-(4-*tert*-butylphenyl)-2-methyl-2-propenylidene]-malononitrile, as matrix. NMR spectroscopy was performed on Bruker AVII400 and AVII500 with cryoprobe. Chemical shifts are referenced to residual protic impurities in the solvent ($CDCl_3$) (^1H; 7.24 ppm) or the deuterated solvent itself (^{13}C; 77.0 ppm). The resonance multiplicities are indicated as, s (singlet), bs (broad singlet), d (doublet), t (triplet), q (quartet) and m (multiplet). For Uv/vis spectra a Perkin Elmer Lambda 1050 was used with 1 cm path length in quartz glass Suprasil (Hellma) cuvettes (scan rate 140 nm/min). In all cases the background solvent was subtracted.

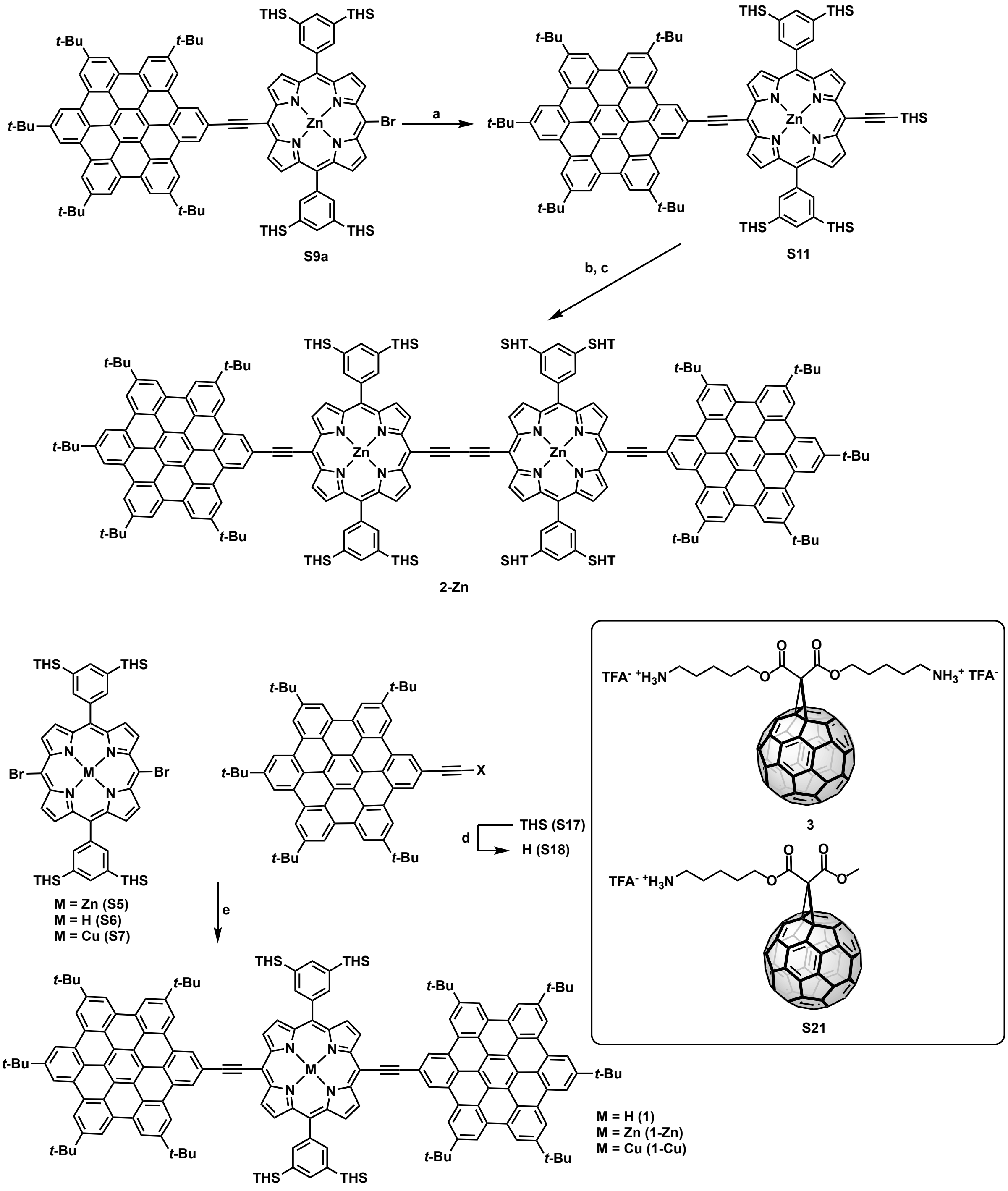

Scheme S1. Bottom: Synthesis of HBC end-capped porphyrin dimer **2-Zn**; a) THF, DIPA, 25 % $Pd(PPh_3)_2Cl_2$, 50 % CuI, THS-acetylene, 60 °C, 19 h; b) THF, TBAF, rt, 30 min; c) THF, DIPA, 10 % $Pd(PPh_3)_2Cl_2$, 75 % CuI, 3 equiv. *p*-benzoquinone, rt, 19 h (67 % over all steps). Bottom: Synthesis of bis-alkynyl-bridged HBC-porphyrin conjugates **1**, **1-Cu**, **1-Zn**; d) THF, TBAF, rt, 30 min (93 %); e) DIPA/THF, 5 % $Pd(PPh_3)_2Cl_2$, 5 % CuI, 60 °C, 15 h (**1-Zn** 57 %, **1-Cu** 89 %); for **1**: NEt_3/THF, 10 % $Pd(OAc)_2$, 4 equiv. $AsPh_3$, 60 °C, 48 h (17 %). Box shows fullerene Bingel adducts (**3** and **S21**) for complexation experiments

Dipyrromethane; S1

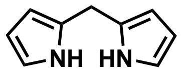

$C_9H_{10}N_2$
M = 146,19

Synthesized according to previously published protocol: Littler, B. J.; Miller, M. A.; Hung, C.-H.; Wagner, R. W.; O'Shea, D. F.; Boyle, P. D.; Lindsey, J. S. J. Org. Chem. 1999, 64, 1391–1396

^{1}H NMR (400 MHz; $CDCl_3$; rt): δ [ppm] = 7.76 (bs, 2H), 6.62 (d, J = 1.20 Hz), 6.14 (m, 2H), 6.02 (m, 2H), 3.94 (s, 2H).

^{13}C NMR (100 MHz; $CDCl_3$; rt): δ [ppm] = 129.1, 117.4, 108.4, 108.3, 106.5, 26.4.

3,5-Bis(trihexylsilyl)benzaldehyde; S2

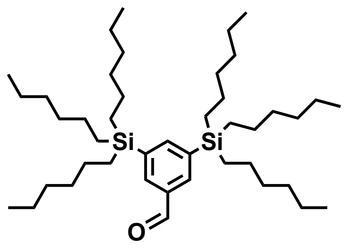

$C_{43}H_{82}OSi_2$
M = 671,30

Synthesized according to previously published protocol: Grozema, F. C.; Houarner-Rassin, C.; Prins, P.; Siebbeles, L. D. A.; Anderson, H. L. J. Am. Chem. Soc. 2007, 129, 13370–13371

R_f (SiO_2): 0.10 (petrol ether 40-60 °C).

^{1}H NMR (400 MHz; $C_2D_2Cl_4$; rt): *δ* [ppm] = 10.01 (s, 1H), 7.00 (s, 2H), 7.82 (s, 1H)

1.29–0.78 (m, 78H).

^{13}C NMR (100 MHz; $CDCl_3$; rt): *δ* [ppm] = 193.6, 146.1, 138.3, 135.9, 134.6, 33.4, 31.5, 23.7, 22.6, 14.1, 12.3.

5-15-Bis(3,5-di(trihexylsilyl)phenyl)porphyrin; S3

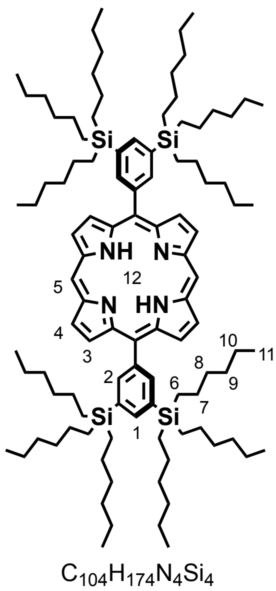

$C_{104}H_{174}N_4Si_4$
M = 1592,90

Synthesized according to previously published protocol: Grozema, F. C.; Houarner-Rassin, C.; Prins, P.; Siebbeles, L. D. A.; Anderson, H. L. J. Am. Chem. Soc. 2007, 129, 13370–13371

R_f (SiO_2): 0.81 (petrol ether 40-60 °C).

^1H NMR (400 MHz; $CDCl_3$; rt): δ [ppm] = 10.32 (s, 2H, 5), 9.39 (d, J = 4.40 Hz, 4H), 9.08 (d, J = 4.40 Hz, 4H), 8.35 (s, 4H, 2), 8.00 (s, 2H, 1), 1.52–0.85 (m; 156H, 6–11), -3.05 (s, 2H, 12).

^{13}C NMR (125 MHz; $CDCl_3$; rt): δ [ppm] = 147.4, 145.1, 141.1, 139.5, 139.2, 135.3, 131.4, 131.1, 120.3, 105.1, 33.6, 31.6, 24.1, 22.7, 14.2, 12.7.

UV/Vis (THF; rt): λ [nm] (rel. Int. [%]) = 407 (100), 503 (5), 537 (3), 576 (2), 632(1).

5-15-Bis(3,5-di(trihexylsilyl)phenyl)porphyrinato-ZnII; S4

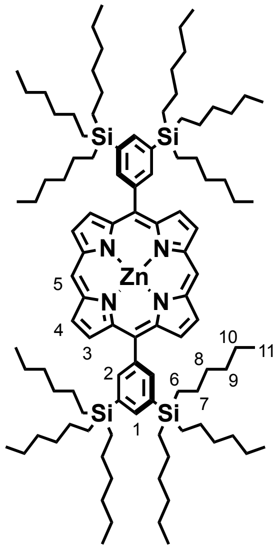

$C_{104}H_{172}N_4Si_4Zn$
M = 1656,27

Synthesized according to previously published protocol: Grozema, F. C.; Houarner-Rassin, C.; Prins, P.; Siebbeles, L. D. A.; Anderson, H. L. J. Am. Chem. Soc. 2007, 129, 13370–13371

R_f (SiO_2): 0.43 (petrol ether 40-60°C).

^{1}H NMR (400 MHz; $CDCl_3$; rt): δ [ppm] = 10.34 (s, 2H, 5), 9.43 (d, J = 4.40 Hz, 4H), 9.13 (d, J = 4.40 Hz, 4H), 8.33, (s, 4H, 2), 7.99 (s, 2H, 1), 1.51–0.83 (m, 156H, 6–11).

^{13}C NMR (125 MHz; $CDCl_3$; rt): δ [ppm] = 150.4, 149.4, 140.8, 140.6, 139.0, 134.9, 132.7, 131.6, 121.3, 106.2, 33.5, 31.6, 24.0, 22.6, 14.1, 12.7.

UV/Vis (THF; rt): λ [nm] (rel. Int. [%]) = 415 (100), 545 (4), 581 (1).

5-15-Dibromo-10,20-bis(3,5-di(trihexylsilyl)phenyl)porphyrinato-ZnII; S5

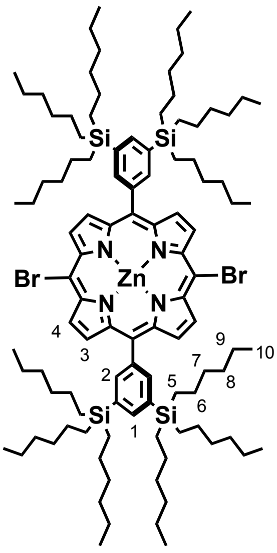

$C_{104}H_{170}Br_2N_4Si_4Zn$
M = 1814,06

Synthesized according to previously published protocol: Grozema, F. C.; Houarner-Rassin, C.; Prins, P.; Siebbeles, L. D. A.; Anderson, H. L. J. Am. Chem. Soc. 2007, 129, 13370–13371

R_f (SiO_2): 0.51 (95:5 petrol ether (40-60 °C/ CH_2Cl_2).

^{1}H NMR (400 MHz; $CDCl_3$; rt): δ [ppm] = 9.74 (d, J = 4.80 Hz, 4H), 8.94 (d, J = 4.80, 4H), 8.23 (s, 4H, 2), 8.00 (s, 2H, 1), 1.48–0.84 (m, 156H, 5–10).

^{13}C NMR (125 MHz; $CDCl_3$; rt): δ [ppm] = 151.2, 150.2, 140.6, 140.2, 139.3, 135.1, 133.8, 133.2, 123.7, 105.3, 33.6, 31.7, 24.1, 22.7, 14.2, 12.7.

MS (LDI): *m/z* (*rel. int.*) = 1814.53 $[M]^+$ (*100*).

UV/Vis (THF; rt): λ [nm] (rel. Int. [%]) = 426 (85), 432 (100), 566 (4), 607 (3).

5-15-Dibromo-10,20-bis(3,5-di(trihexylsilyl)phenyl)porphyrin; S6

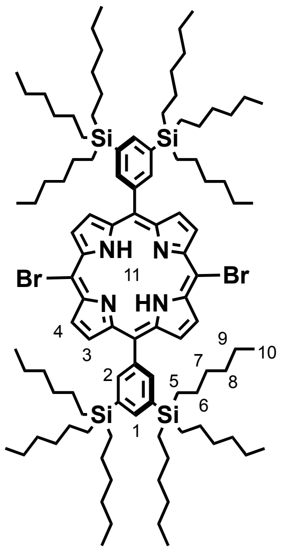

$C_{104}H_{172}Br_2N_4Si_4$
M = 1750,70

A 150 mL RBF equipped with a magnetic stir bar was charged with 5-15-dibromo-10,20-bis(3,5-di(trihexylsilyl)phenyl)-porphyrinato-Zn^{II} **S5** (200 mg, 110 µmol) and dissolved in $CHCl_3$ (100 mL). TFA (1 mL) was added in 0.1 mL steps and the mixture was stirred at rt for 7 minutes. The mixture was quenched with pyridine (4 mL) and subsequently purified by plug filtration (SiO_2, 12x6 cm; petrol ether 40-60 °C), yielding the product as purple oil in 99 % (190 mg, 109 µmol).

R_f (SiO_2): 0.85 (petrol ether 40-60°C).

^{1}H NMR (400 MHz; $CDCl_3$; rt): δ [ppm] = 9.62 (d, *J* = 4.80 Hz, 4H), 8.83 (d, *J* = 4.80 Hz, 4H), 8.24 (s, 4H, 2), 8.01 (s, 2H, 1), 1.52–0.86 (m, 156H, 5–10), -2.66 (s, 2H, 11).

^{13}C NMR (125 MHz; $CDCl_3$; rt): δ [ppm] = 140.7, 139.6, 139.5, 135.3, 132.6, 122.7, 103.6, 33.5, 31.6, 24.0, 22.7, 14.2, 12.6.

MS (LDI): *m/z* (*rel. int.*) = 1748.52 [M]$^+$ (*100*).

UV/Vis (THF; rt): λ [nm] (rel. Int. [%]) = 421 (99), 425 (100), 522 (5), 556 (4), 602 (3), 661 (3).

5-15-Dibromo-10,20-bis(3,5-di(trihexylsilyl)phenyl)porphyrinato-CuII; S7

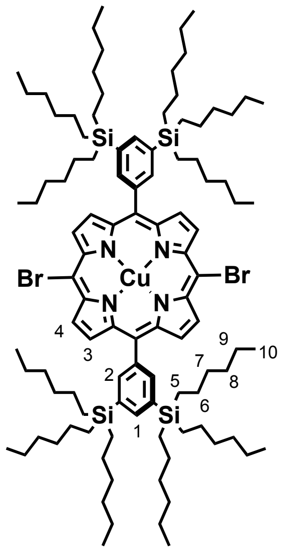

$C_{104}H_{170}Br_2CuN_4Si_4$
M = 1812,23

A 50 mL RBF equipped with a magnetic stir bar was loaded with 5-15-dibromo-10,20-bis(3,5-di(trihexylsilyl)phenyl)-porphyrin **S6** (190 mg, 109 µmol) and dissolved in $CHCl_3$ (10 mL). A solution of $Cu(OAc)_2 \cdot H_2O$ (65 mg, 327 µmol) in MeOH (5 mL) was added and the mixture was stirred at rt for 3 h. The mixture was purified by plug filtration (SiO_2, 10x6 cm; petrol ether 40-60 °C) yielding the product as dark red oil in 95 % (186 mg, 103 µmol).

R_f (SiO_2): 0.96 (petrol ether 40-60 °C).

^{1}H NMR (400 MHz; $CDCl_3$; rt): δ [ppm] = 9.65 (bs), 8.63 (bs), 8.27 (s), 8.04 (bs), 7.90 (bs), 7.73 (bs), 7.55 (s), 1.33 (s), 0.91 (s).

MS (LDI): *m/z* (*rel. int.*) = 1813.36 $[M+H]^+$ (*100*).

UV/Vis (THF; rt): λ [nm] (ε [$M^{-1}cm^{-1}$]) = 422 (532000), 549 (26200), 590 (7280).

Bis-(HBC-ethynyl)porphyrin; 1

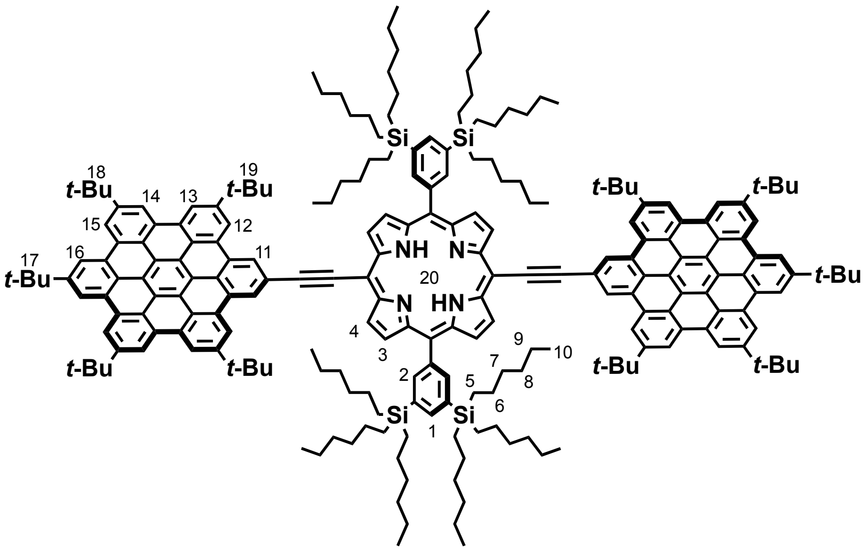

$C_{232}H_{286}N_4Si_4$
M = 3243,21

A 100 mL Schlenk tube equipped with a magnetic stir bar, was charged with 5-15-dibromo-10,20-bis(3,5-di(trihexylsilyl)-phenyl)-porphyrin **S6** (75 mg, 43 µmol), $Pd(OAc)_2$ (1 mg, 4.3 µmol), $AsPPh_3$ (5.2 mg, 17.1 µmol) and dissolved in dry NEt_3 (6 mL). The mixture was degassed and put under argon. The mixture was stirred for 20 min. A degassed solution of HBC-acetylene **S18** (100 mg, 89 µmol) in dry THF (12 mL) was added via syringe at rt. The mixture was heated to 60 °C for 48 h. The mixture was concentrated and purified by plug filtration (SiO_2, 3x6 cm; CH_2Cl_2). Further purification was carried out by size exclusion chromatography (SX1, 2x100 cm; THF), column chromatography (SiO_2 15x6 cm; 9:1 petrol ether (40-60 °C)/ CH_2Cl_2) and crystalization from CH_2Cl_2 and MeOH, yielding the product as green solid in 17 % (24 mg, 7.4 µmol).

R_f (SiO_2): 0.39 (9:1 petrol ether (40-60°C)/CH_2Cl_2).

^{1}H NMR (400 MHz; $CDCl_3$; rt): δ [ppm] = 10.04 (d, J = 4.40 Hz, 4H), 9.84 (s, 4H), 9.48 (s, 4H), 9.39 (s, 4H), 9.34 (s, 12H) 9.01 (d, J = 4.40 Hz, 4H), 8.39 (s, 4H, 2), 8.04 (s, 2H, 1), 1.86 (s, 36H), 1.84 (s, 54H), 1.42–0.85 (m, 156H, 5–10), -1.69 (s, 2H, 20).

^{13}C NMR (125 MHz; $CDCl_3$; rt): δ [ppm] = 149.5, 149.2, 140.8, 135.4, 131.4, 130.7, 130.54, 130.45, 129.7, 124.9, 124.1, 123.9, 121.0, 120.7, 119.5, 119.1, 96.1, 35.9, 35.8, 33.5, 32.1, 32.0, 31.6, 24.1, 22.7, 14.2, 12.7.

MS (LDI): *m/z* (*rel. int.*) = 3243.26 $[M]^+$ (*100*).

Bis-(HBC-ethynyl)porphyrinato-Zn^{II}; 1-Zn

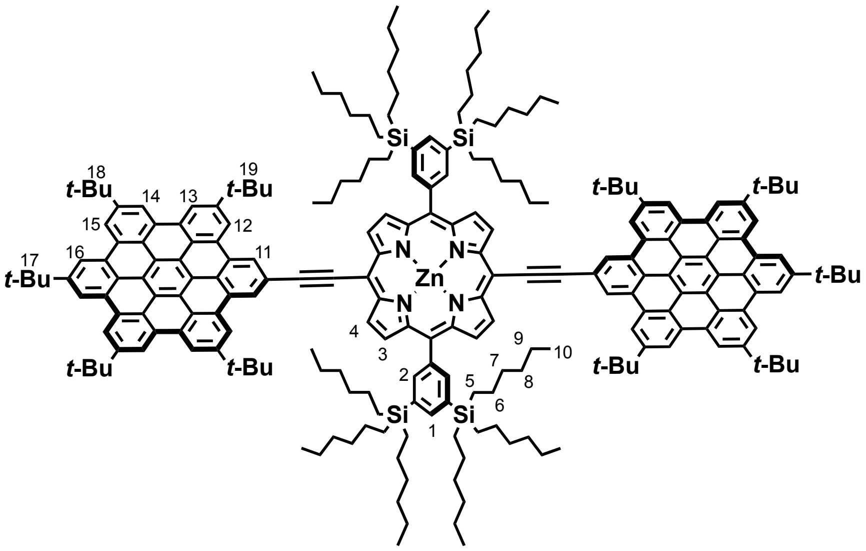

$C_{232}H_{284}N_4Si_4Zn$
M = 3306,57

A 100 mL Schlenk tube equipped with a magnetic stir bar was charged with 5-15-dibromo-10,20-bis(3,5-di(trihexylsilyl)phenyl)-porphyrinato-Zn^{II} **S5** (100 mg, 55 µmol), $Pd(PPh_3)_2Cl_2$ (8 mg, 11 µmol), CuI (2 mg, 11 µmol) and dissolved in DIPA (5 mL). The mixture was degassed and put under argon. The mixture was stirred for 20 min. A degassed solution of HBC-acetylene **S18** (100 mg, 121 µmol) in dry THF (10 mL) was added via syringe at rt. The mixture was heated to 60 °C for 15 h. The mixture was concentrated and purified by plug filtration (SiO_2, 12x6 cm; 8:2 petrol ether (40-60 °C)/ CH_2Cl_2). Further purification was carried out by size exclusion chromatography (SX1, 2x100 cm; THF), column chromatography (SiO_2, 20x6 cm; 9:1 petrol ether (40-60 °C)/ CH_2Cl_2) and crystallization from CH_2Cl_2 and MeOH, yielding the product as green solid in 57 % (104 mg, 32 µmol).

R_f (SiO_2): 0.43 (8:2 petrol ether (40-60°C)/CH_2Cl_2).

^{1}H NMR (400 MHz; $CDCl_3$; rt): *δ* [ppm] = 10.22 (d, *J* = 4.60 Hz, 4H), 9.73 (s, 4H,), 9.38 (s, 4H), 9.32–9.28 (m, 16H), 9.17 (d, *J* = 4.60 Hz, 4H), 8.46 (s, 4H, 2), 8.09 (s, 2H, 1), 1.89 (s, 36H), 1.88 (s, 18H, 17), 1.87 (s, 36H), 1.58–0.99 (m, 120H, 5–9), 0.87 (t, *J* = 4.80 Hz, 36 H, 10).

^{13}C NMR (125 MHz; $CDCl_3$; rt): *δ* [ppm] = 152.4, 150.7, 149.14, 140.06, 148.9, 140.7, 140.4, 139.4, 135.2, 133.2, 131.4, 131.1, 130.6, 130.4, 130.3, 129.6, 125.8, 124.8, 124.4, 123.8, 123.8, 121.8, 121.0, 120.8, 120.5, 120.0, 119.4, 119.3, 118.9, 102.0, 98.1, 93.0, 35.83, 35.79, 35.8, 33.6, 32.12, 32.10, 31.6, 24.1, 22.7, 14.2, 12.7.

MS (LDI): *m/z* (*rel. int.*) = 3306.10 [M]$^+$ (*100*).

Bis-(HBC-ethynyl)porphyrinato-Cu^{II}; 1-Cu

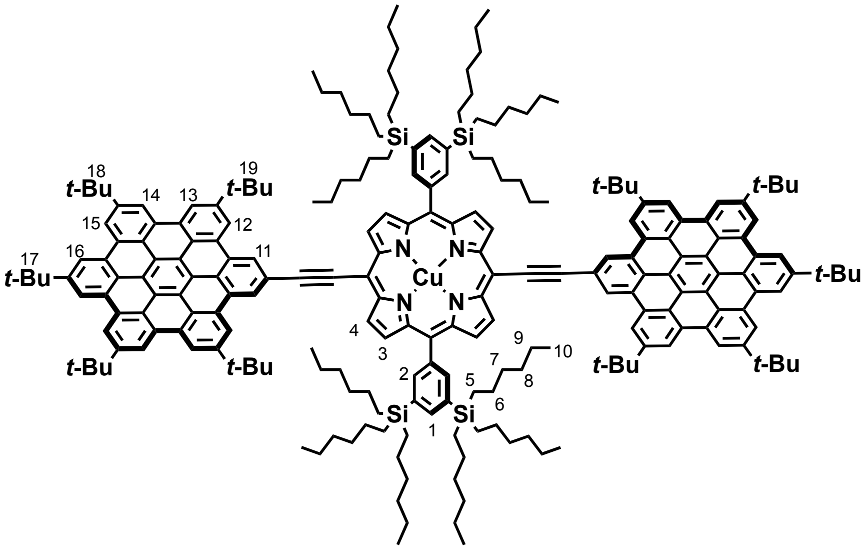

$C_{232}H_{284}CuN_4Si_4$
M = 3304,74

A 100 mL Schlenk tube equipped with a magnetic stir bar was charged with 5-15-dibromo-10,20-bis(3,5-di(trihexylsilyl)phenyl)-porphyrinato-Cu^{II} **S7** (50 mg, 28 µmol), $Pd(PPh_3)_2Cl_2$ (2 mg, 2.8 µmol), CuI (1 mg, 5.6 µmol) and dissolved in dry DIPA (4 mL). The mixture was degassed and put under argon. The mixture was stirred for 20 min. A degassed solution of HBC-acetylene **S18** (57 mg, 69 µmol) in dry THF (8 mL) was added via syringe at rt. The mixture was heated to 60 °C for 15 h. The mixture was concentrated, purified by plug filtration (SiO_2, 3x6 cm; 9:1 petrol ether (40-60 °C)/ CH_2Cl_2). Further purification was carried out by size exclusion chromatography (SX1, 2x100 cm; THF) and crystalization from CH_2Cl_2 and MeOH, yielding the product as green solid in 89 % (83 mg, 25 µmol).

R_f (SiO_2): 0.34 (9:1 petrol ether (40-60°C)/CH_2Cl_2).

^{1}H NMR (400 MHz; $CDCl_3$; rt): *δ* [ppm] = 9.16 (bs), 8.04 (bs), 1.91 (bs), 1.37 (bs), 0.92 (bs).

(LDI): *m/z* (*rel. int.*) = 3304.75 [M]$^+$ (*100*).

Mono-(HBC-ethynyl)porphyrinato-ZnII; S11

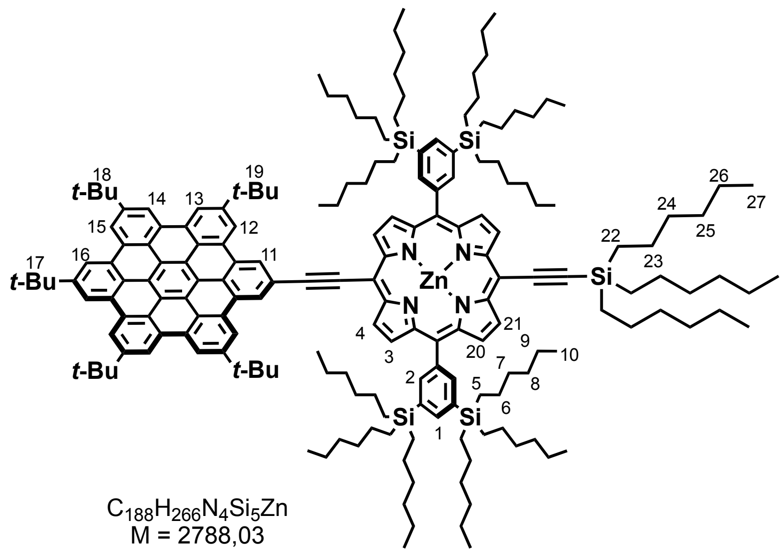

A 100 mL Schlenk tube equipped with a magnetic stir bar was charged with monobromo-mono-HBC-porphyrinato-Zn^{II} **S9a** (11 mg, 4.3 µmol), $Pd(PPh_3)_2Cl_2$ (1 mg, 1.3 µmol), CuI (0.5 mg, 2.6 µmol) and dissolved in THF (5 mL) and DIPA (2 mL). The mixture was degassed and stirred at rt for 20 min, then THS-acetylene (7.3 µL, 20 µmol) was added and the mixture was heated to 60 °C under argon for 19 h. The mixture was concentrated and purified by plug filtration (SiO_2, 20x6 cm; 9:1 petrol ether (40-60 °C)/ CH_2Cl_2). The obtained product was used in the next step without further purification or characterization.

(Monobromo-mono-HBC-porphyrinato-Zn^{II} **S9a** *was isolated as side-product in the reaction to bis-(HBC-ethynyl)porphyrinato-Zn^{II}* **S9** *by size exclusion chromatography as second fraction. It contained partially debrominated mono-adduct, which could not be separated from* **S9a.***)*

R_f (SiO_2): 0.15 (petrol ether 40-60 °C).

Bis-HBC-porphyrin dimer; 2-Zn

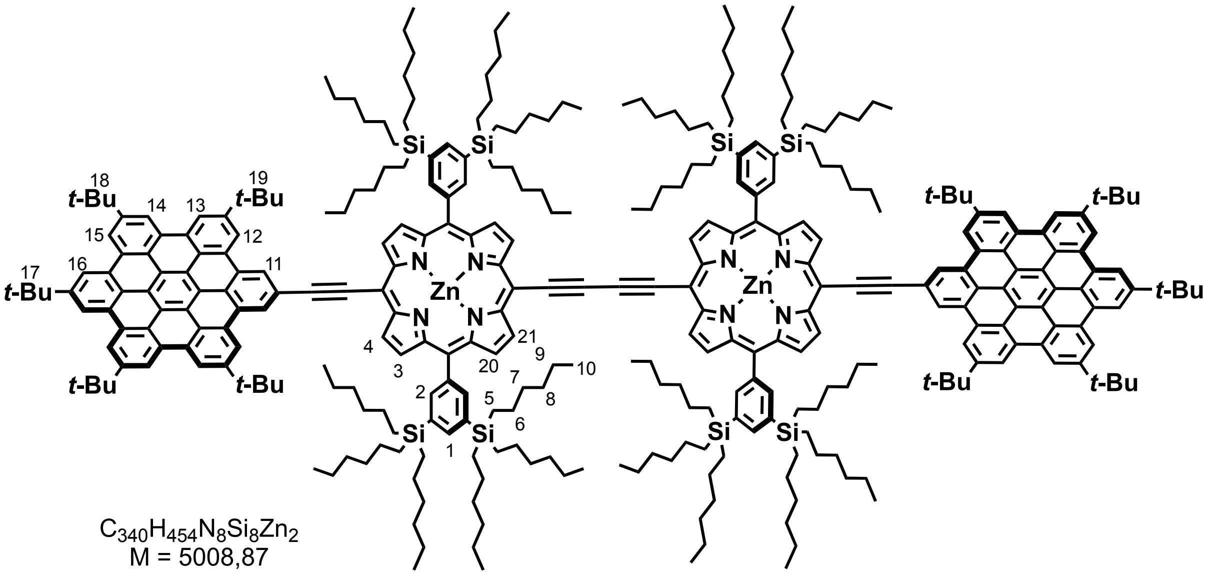

A 50 mL RBF equipped with a magnetic stir bar was charged with mono-(HBC-ethynyl)porphyrinato-Zn^{II} **S11** (10 mg, 3.6 µmol) and dissolved in THF (5 mL). TBAF (1M solution in THF) (0.1 mL, 100 µmol) was added and the mixture was stirred at rt for 30 min. The mixture was purified by plug filtration (SiO_2, 3x6 cm; CH_2Cl_2) and concentrated. The green solid was transferred to a 5 mL RBF and dissolved in THF (1 mL) and DIPA (1 mL). $Pd(PPh_3)_2Cl_2$ (0.2 mg, 0.27 µmol) CuI (0.5 mg, 2.7 µmol) and 1,4-benzoquinone (1.2 mg, 10.8 µmol) were added as solid and the mixture was stirred at rt for 19 h. The mixture was pre-purified by plug filtration (SiO_2, 2x3 cm; CH_2Cl_2), concentrated and purified by size exclusion chromatography (SX1, 100x2 cm; THF) and crystallization from CH_2Cl_2 and MeOH, yielding the product as dark green solid in 67 % (6 mg, 1.2 µmol).

R_f (SiO_2): 0.39 (9:1 petrol ether (40-60°C)/CH_2Cl_2).

^{1}H NMR (400 MHz; $CDCl_3$; rt): δ [ppm] = 10.11 (d, *J* = 4.80 Hz, 4H), 9.98 (d, *J* = 4.80 Hz, 4H), 9.81 (s, 4H), 9.46 (s, 4H), 9.36 (s, 4H), 9.33 (s, 12H), 9.07 (d, *J* = 4.80 Hz, 4H), 9.05 (d, *J* = 4.80 Hz, 4H), 8.37 (s, 8H, 2) 8.04 (s, 4H, 1) 1.86 (s, 36H), 1.85 (s, 54H), 1.55–0.86 (m, 312H, 5–10).

^{13}C NMR (125 MHz; $CDCl_3$; rt): δ [ppm] = 152.2, 150.8, 150.4, 149.4, 149.3, 149.2, 143.7, 140.7, 140.4, 139.3, 135.1, 133.4, 133.1, 131.3, 130.9, 130.7, 130.52, 130.50, 130.43, 129.7, 126.0, 124.9, 124.6, 124.0, 123.9, 122.5, 122.1, 121.2, 121.0, 120.7, 120.2, 119.54, 119.46, 119.02, 118.99, 102.3, 100.0, 98.0, 35.9, 35.8, 33.6, 32.1, 32.0, 31.7, 24.1, 22.7, 14.2, 12.7.

(LDI): *m/z* (*rel. int.*) = 5001.25 [M]$^+$ (*100*).

2,3,4,5-Tetrakis(4-*tert*-butylphenyl)cyclopenta-2,4-diene-1-one; S13

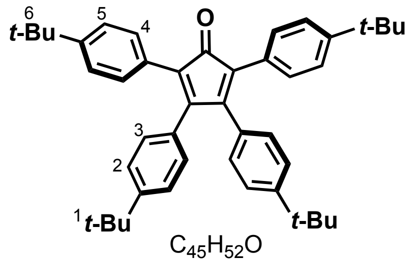

$C_{45}H_{52}O$
M = 608,91

The preparation of **S13** and the necessary precursors is described in:

D. Lungerich, J. F. Hitzenberger, M. Marcia, F. Hampel, T. Drewello, N. Jux, *Angew. Chem. Int. Ed.* **2014**, *53*, 12231–12235.

4-Bromo-4'-*t*-butyltolan; S14

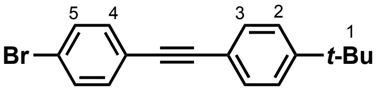

A 250 mL Schlenk-RBF equipped with a magnetic stir bar and a rubber septum, was charged with 1-bromo-4-iodobenzene (3.0 g,10.6 mmol), $Pd(PPh_3)_2Cl_2$ (150 mg, 0.21 mmol), CuI (20 mg, 0.10 mmol) and dissolved in DIPA (125 mL). The mixture was degassed. Then, 4-*t*-butylphenylacetylene (1.69 g, 10.7 mmol) was added neat via syringe and the mixture was stirred at rt for 20 h. The mixture was concentrated and purified by liquid chromatography (SiO_2, 12x8 cm; hexanes), yielding a white solid in 99 % (3.29 g; 10.5 mmol).

R_f (SiO_2): 0.71 (7:3 hexanes/EtOAc).

^{1}H NMR ($CDCl_3$, 400 MHz, rt): δ [ppm] = 7.45 (m, 4H, ar-*H*), 7.36 (m, 4H, ar-*H*), 1.31 (s, 9H, 1).

^{13}C NMR ($CDCl_3$, 100 MHz, rt): δ [ppm] = 151.8, 133.0, 131.6, 131.3, 125.4, 122.5, 122.2, 119.8, 90.7, 87.7, 34.8, 31.2.

4-Bromo-4′,4′′,4′′′,4′′′′,4′′′′′-penta(*tert*-butyl)hexaphenylbenzene; S15

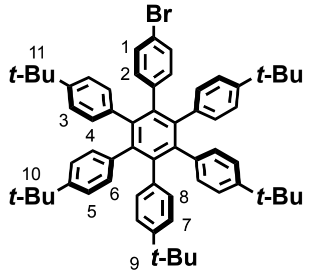

$C_{62}H_{69}Br$
M = 894,14

A 100 mL pressure flask equipped with a magnetic stir bar, was charged with 2,3,4,5-tetrakis(4-(*t*-butyl)phenyl)cyclopentadienone **S13** (500 mg, 0.82 mmol), 1-bromo-4’-*t*-butyltolan **S14** (257 mg, 0.82 mmol) and dissolved in toluene (10 mL). The flask was purged with argon, closed and heated to 240 °C for 18 h. After cooling to rt, MeOH (90 mL) was added. The off-white precipitate was filtered, washed with MeOH and dried in vacuo, yielding a white solid in 75 % (550 mg, 615 µmol).

R_f (SiO_2): 0.57 (20:1 hexanes/EtOAc).

^{1}H NMR ($CDCl_3$, 400 MHz, rt): δ [ppm] = 6.95 (d, J = 8.0 Hz, 2H, ar-*H*), 6.83 (d, J = 8.40 Hz, 4H, ar-*H*), 6.80–6.77 (m, 6H), 6.70 (d, J = 8.40 Hz, 2H, ar-*H*), 6.66–6.62 (m, 10H, ar-*H*), 1.11 (s, 18H, 11), 1.08 (s, 27H, 9, 10).

^{13}C NMR ($CDCl_3$, 100 MHz, rt): δ [ppm] = 147.8, 147.5, 147.4, 140.8, 140.7, 140.14, 140.05, 138.4, 137.73, 137.70, 137.5, 133.3, 131.02, 130.97, 130.96, 129.5, 123.4, 123.1, 123.0, 119.0, 34.1, 34.04, 34.03, 31.19, 31.17;

MS (LDI): *m/z* (*rel. int.*) = 894 [M^+] (100).

2-Bromo-5,8,11,14,17-penta(*tert*-butyl)hexa-peri-hexabenzocoronene; S16

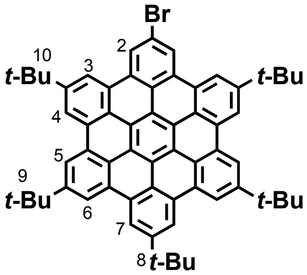

$C_{62}H_{57}Br$
M = 882,04

A 500 Schlenk-RBF equipped with a magnetic stir bar, a rubber septum and a glass tube was charged with 4-Bromo-4′,4′′,4′′′,4′′′′,4′′′′′-penta(*tert*-butyl)hexaphenylbenzene **S15** (550 mg, 615 µmol), 4 Å molecular sieves (5 g) and dissolved in CH_2Cl_2 (250 mL). The mixture was cooled to 0 °C and degassed by bubbling N_2 through the solution for 30 min. A solution of $FeCl_3$ (1.6 g, 9.80 mmol) in $MeNO_2$ (5.3 mL) was added slowly while bubbling vigorously N_2 into the reaction mixture at 0 °C. After 60 min, the mixture was stirred for another 45 min at 0 °C without N_2 bubbling. The reaction was quenched by addition of 100 mL MeOH. The mixture was concentrated to a volume of approximately 60 mL. The yellow precipitate was filtered, washed with MeOH and dried in vacuo, yielding a bright yellow solid in 97 % (530 mg; 601µmol).

R_f (SiO_2): 0.56 (20:1 hexanes/EtOAc).

^{1}H NMR (400 MHz; $CDCl_3$; rt): δ [ppm] = 9.17 (s, 2H, HBC-*H*), 9.12 (s, 2H, HBC-*H*), 9.04 (s, 2H, HBC-*H*), 8.34 (s, 2H, HBC-*H*), 8.67 (s, 2H, HBC-*H*), 8.65 (s, 2H, HBC-*H*), 1.90 (s, 9H, 8), 1.86 (s, 18H, 9), 1.78 (s, 18H, 10).

^{13}C NMR (100 MHz; $CDCl_3$; rt): δ [ppm] = 148.3, 148.1, 147.9, 131.6, 130.1, 129.8, 129.4, 128.3, 123.4, 123.2, 123.1, 123.0, 122.9, 121.0, 119.8, 119.6, 119.3, 119.0, 118.53, 118.48, 118.3, 35.8, 35.7, 35.6, 32.22, 32.18, 32.10;

MS (LDI): *m/z* (*rel. int.*) = 882 [M$^+$] (100 %).

HRMS (APPI; MeCN, CH_2Cl_2, toluene): *m/z* calc. for $C_{62}H_{57}Br$ [M$^+$] 882.362255, found: 882.364700.

2-(trihexylsilylethynyl)-5,8,11,14,17-penta(*tert*-butyl)hexa-peri-hexabenzocoronene; S17

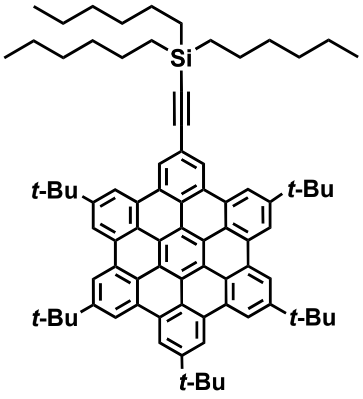

$C_{82}H_{96}Si$
M = 1109,76

A 100 mL Schlenk tube equipped with a magnetic stir bar was charged with 2-bromo-5,8,11,14,17-penta(*tert*-butyl)hexa-peri-hexabenzocoronene **S16** (300 mg, 340 µmol), $Pd(PPh_3)_2Cl_2$ (24 mg, 34.0 µmol) and CuI (13 mg, 68.0 µmol). The solids were dissolved in THF (30 mL) and DIPA (7.5 mL). The mixture was degassed and put under argon. The mixture was stirred at rt for 20 min followed by addition of neat trihexylsilylacetylene (188 µL, 510 µmol). The mixture was heated to 60 °C under argon for 15 h. The reaction mixture was concentrated to dryness and purified by column chromatography (SiO_2, 12x6 cm; 9:1 petrol ether 40-60 °C/ CH_2Cl_2). The bright yellow fraction was collected, concentrated and precipitated from CH_2Cl_2/MeOH. The solid was filtered, washed with MeOH and dried in vacuo, yielding a bright yellow solid in 99% (280 mg, 338 µmol).

R_f (SiO_2): 0.49 (9:1 petrol ether 40-60 °C/ CH_2Cl_2).

^{1}H NMR (400 MHz; $CDCl_3$; rt): δ [ppm] = 9.10 (s, 2H, HBC-*H*), 9.05 (s, 2H, HBC-*H*), 9.01 (s, 2H, HBC-*H*), 8.93 (s, 2H, HBC-*H*), 8.78 (s, 2H, HBC-*H*), 8.73 (s, 2H, HBC-*H*), 1.91 (s, 9H), 1.88 (s, 18H), 1.82 (s, 18H), 1.75–166 (m, 6H), 1.56–152 (m, 6H), 1.49–139 (m, 12H), 0.98–0.94 (m, 15H).

^{13}C NMR (125 MHz; $CDCl_3$; rt): δ [ppm] = 148.3, 148.1, 130.1, 129.9, 129.6, 129.0, 124.8, 124.7, 123.22, 123.15, 123.0, 120.4, 120.2, 120.0, 119.7, 119.0, 118.6, 118.5, 118.4, 108.1, 93.2, 35.8, 35.7, 35.6, 33.5, 32.22, 32.19, 32.15, 31.8, 24.3, 22.8, 14.2, 13.7.

MS (LDI): *m/z* (*rel. int.*) = 1121.26 [M^++C] (100 %).

UV/Vis (THF; rt): λ [nm] (ε [$M^{-1}cm^{-1}$]) = 331 (60700), 345 (161000), 363 (385000), 373 (s, 138000), 393 (116000), 403 (s, 46400), 410 (s, 34500).

2-(Ethynyl)-5,8,11,14,17-penta(*tert*-butyl)hexa-peri-hexabenzocoronene; S18

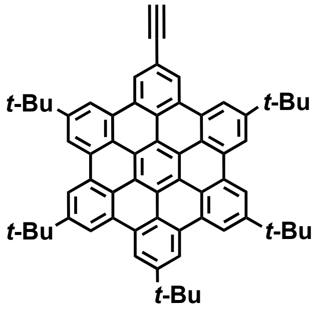

$C_{64}H_{58}$
M = 827,17

A 50 mL Schlenk-RBF equipped with a magnetic stir bar was charged with 2-(trihexylsilylethynyl)-5,8,11,14,17-penta(*tert*-butyl)hexa-peri-hexabenzocoronene **S17** (150 mg, 135 µmol) and dissolved in THF (15 mL) under argon. 1 M TBAF/THF solution (1 mL) was added at rt and the mixture was stirred for 30 min. The mixture was quenched by addition of MeOH (15 mL), concentrated and purified by plug filtration (SiO_2, 2x2 cm; CH_2Cl_2). The yellow fraction was concentrated and precipitated from CH_2Cl_2/MeOH. The yellow solid was filtered, washed with MeOH and dried in vacuo, yielding a bright yellow solid in 93 % (104 mg, 126 µmol).

R_f (SiO_2): 0.32 (9:1 petrol ether 40-60 °C/ CH_2Cl_2).

^{1}H NMR (400 MHz; $CDCl_3$; rt): δ [ppm] = 9.20 (s, 2H, HBC-*H*), 9.150 (s, 2H, HBC-*H*), 9.09 (s, 2H, HBC-*H*), 9.01 (s, 2H), HBC-*H*, 8.87 (s, 2H, HBC-*H*), 8.85 (s, 2H, HBC-*H*), 3.53 (s, 1H, CC*H*), 1.88 (s, 9H, *t*-Bu), 1.85 (18H, *t*-Bu), 1.78 (18H, *t*-Bu).

^{13}C NMR (125 MHz; $CDCl_3$; rt): δ [ppm] = 148.7, 148.51, 148.50, 130.4, 130.2, 130.1, 130.0, 129.8, 129.0, 125.5, 124.7, 123.41, 123.37, 123.3, 120.5, 120.2, 119.9, 119.3, 119.2, 119.1, 118.8, 118.70, 118.66, 118.6, 85.6, 77.8, 35.8, 35.70, 35.66, 32.12, 32.09, 32.06.

MS (LDI): *m/z* (*rel. int.*) = 827.60 [M^+] (100 %).

UV/Vis (THF; rt): λ [nm] (ε [$M^{-1}cm^{-1}$]) = 330 (63400), 345 (172000), 362 (409000), 371 (s, 145000), 392 (131000), 402 (s, 45600), 409 (s, 30100).

5-((*tert*-Butoxycarbonyl)amino)pentyl methyl malonate; S19

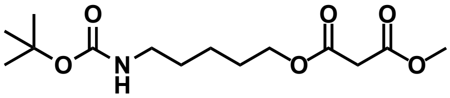

$C_{14}H_{25}NO_6$
M = 303,36

A 100 mL Schlenk-RBF equipped with a magnetic stir bar and 25 mL dropping funnel was charged with *tert*-butyl (5-hydroxypentyl)carbamate (2.00 g; 9.85 mmol), pyridine (1.00 mL; 12.3 mmol) and dissolved in CH_2Cl_2 (50.0 mL) under N_2. Via dropping funnel methyl malonyl chloride (1.06 mL, 9.85 mmol) dissolved in CH_2Cl_2 (5.00 mL) was dropped to the mixture over a period of 15 min and the mixture was stirred at room temperature overnight. The mixture was washed with brine (3*50 mL) dried over $NaSO_4$, filtered and concentrated. Purification by column chromatography (SiO_2; CH_2Cl_2/EtOAc 7:3) yielded the product as yellow oil in 71 % (2.12 g; 6.99 mmol) yield.

R_f (SiO_2): 0.49 (CH_2Cl_2/EtOAc 9:1).

^{1}H NMR (400 MHz; $CDCl_3$; rt): δ [ppm] = 1.28 - 1.49 (m, 13 H, $COOCH_2CH_2CH_2$, NCH_2CH_2, CCH_3), 1.58 –1.65 (m, 2H, $COOCH_2CH_2$), 3.06 (dd, ^3J = 12.6, 6.5 Hz, 2H, NCH_2), 3.34 (s, 2H, $OOCCH_2COO$), 3.70 (s, 3H, OCH_3), 4.09 (t, ^3J = 6.5 Hz, 2H, $COOCH_2$), 4.58 (br, 1H, NH).

^{13}C NMR (100 MHz; $CDCl_3$; rt): δ [ppm] = 23.0, 28.0, 28.3, 29.5, 40.2, 41.3, 52.4, 65.3, 79.0, 156.0, 166.5, 167.0.

MS (MALDI, dctb): *m/z* = 304 $[M+H]^+$, 326 $[M+H+Na]^+$, 342 $[M+H+Ka]^+$.

IR(ATR; rt): $\tilde{\nu}$[cm^{-1}] = 525, 658, 690, 787, 864, 1011, 1260, 1503, 2963.

5-((*tert*-Butoxycarbonyl)amino)pentyl methyl malonyl –[1,0]–mono–1,2,–dihydro[60]–fullerene; S20

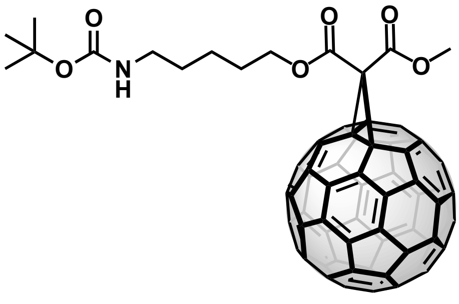

$C_{74}H_{32}NO_6$
M = 1031,07

A 2 L Schlenk-RBF equipped with a magnetic stir bar was charged with C_{60} (1.09 g; 1.51 mmol), 5-((*tert*-Butoxycarbonyl)amino)pentyl methyl malonate **S19** (500 mg; 1.51 mmol) and iodine (288 mg; 2.27 mmol). Toluene (1.00 L) was added and the mixture degassed under the exclusion of light. DBU (560 µL; 3.78 mmol) was added slowly and the mixture was stirred at room temperature for 1 h under nitrogen atmosphere. The crude product was plug filtrated (CH_2Cl_2/EtOAc 1:1) and concentrated. After column chromatography (SiO_2; CH_2Cl_2/EtOAc 8:2) the product was obtained as dark brown solid in 46 % (716 mg; 0.700 mmol) yield.

R_f (SiO_2): 0.68 (CH_2Cl_2/EtOAc 9:1).

^{1}H NMR (400 MHz; $CDCl_3$; rt): δ [ppm] = 1.23 (t, ^3J = 7.2 Hz, 2H, $COOCH_2CH_2CH_2$), 1.41 –1.54 (m, 13 H, $COOCH_2CH_2$, NCH_2CH_2,, CCH_3), 3.12 (dd, ^3J = 12.6, 6.5 Hz, 2H, NCH_2), 4.07 (s, 3H, OCH_3), 4.47 (t, ^3J = 6.5 Hz, 2H, $COOCH_2$), 4.55 (br, 1H, N*H*).

^{13}C NMR (100 MHz; $CDCl_3$; rt): δ [ppm] = 23.2, 28.2, 28.4, 29.7, 40.4, 52.0, 54.0, 67.2, 71.4, 79.2, 139.4, 139.6, 141.5, 142.4, 142.4, 142.7, 143.5, 143.5, 143.6, 144.4, 145.2, 145.2, 145.4, 145.6, 145.7, 145 7, 145.7, 145.8, 145.8, 155.9, 163.5, 164.1.

MS (MALDI): *m/z* = 720 $[C_{60}]^+$, 1044 $[M+Na]^+$.

HRMS (MALDI-TOF): *m/z* calc. for $C_{74}H_{23}NO_6$: 1021.1520 $[M]^+$, $C_{74}H_{23}NNaO_6$: 1044.1418 $[M+Na]^+$, found: 1021.1503, 1044.1417.

IR(ATR; rt): $\tilde{\nu}$[cm^{-1}] = 527, 583, 702, 737, 1167, 1234, 1366, 1392, 1427, 1457, 1510, 168, 1698, 1747, 2858, 2925, 3953.

UV/Vis (; rt): λ [nm] (ε [M^{-1}cm^{-1}]) = 226 (37000), 258 (34000)), 325 (10000), 426 (2000).

5-Aminopentyl methyl malonyl –[1,0]–mono–1,2,–dihydro[60]–fullerene; S21

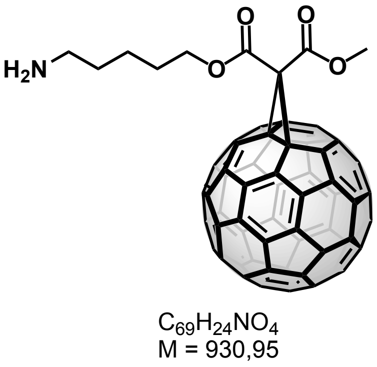

A 10 mL RBF was charged with 5-((*tert*-butoxycarbonyl)amino)pentyl methyl malonate fullerene monoadduct **S20** (664 mg; 0.650 mmol) and dissolved in TFA (5 mL) under the exclusion of light. After stirring overnight, excess TFA was removed in vacuo and co-evaporated with methanol several times to give **S21** as dark brown solid in 96 % (599 mg; 0.650 mmol) yield.

^{1}H NMR (400 MHz; CDCl$_3$; rt): δ [ppm] = 0.97 – 1.03 (m, 2H, COOCH$_2$CH$_2$C*H*$_2$), 1.10 –1.16 (m, 2 H, COOCH$_2$C*H*$_2$), 1.10 –1.16 (m, 2 H, NCH$_2$C*H*$_{2,}$) 2.33 (dd, ^3J = 12.6, 6.5 Hz, 2H, NC*H*$_2$), 3.25 (br, 1H, N*H*$_2$), 3.60 (s, 3H, OC*H*$_3$), 4.03 (t, ^3J = 6.5 Hz, 2H, COOC*H*$_2$).

^{13}C NMR (100 MHz; CDCl$_3$; rt): δ [ppm] = 23.1, 27.3, 28.3, 40.4, 53.1, 55.3, 67.9, 72.2, 139.2, 139.4, 141.4 (2x), 142.2, 142.3, 142.6, 142.6, 143.4 (2x), 143.4, 143.4, 143.5, 144.2 (2x), 145.0, 145.0, 145.1, 145.1 (2x), 145.2, 145.5, 145.5, 145.5, 145.6, 145.6 (2x), 145.9, 146.1, 163.5, 164.1.

MS (MALDI, dctb): *m/z* = 720 [C$_{60}$]$^+$, 922 [M+H]$^+$.

HRMS (MALDI, dctb): *m/z* calc. for C$_{69}$H$_{16}$NO$_4$: 922.1074 [M+H]$^+$, found: 922.1700.

IR(ATR; rt): $\tilde{\nu}$[cm^{-1}] = 527, 581, 699, 736, 1060, 1095, 1115, 1187, 1233, 1258, 1430, 1579, 1747, 2358, 2848, 2921.

UV/Vis (; rt):λ [nm] (ε [M^{-1}cm^{-1}]) = 226 (7300), 256 (6500), 325 (2200), 426 (400).

Bis(5-((*tert*-butoxycarbonyl)amino)pentyl) malonate; S22

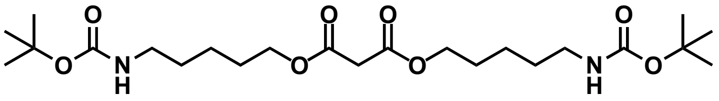

$C_{23}H_{42}N_2O_8$
M = 474,60

A 250 mL Schlenk-RBF equipped with a magnetic stir bar and 50 mL dropping funnel was charged with *tert*-butyl (5-hydroxypentyl)carbamate (8.00 g; 39.4 mmol), methyl malonic acid (2.05 g; 19.7 mmol) and dissolved in dry MeCN (50.0 mL) under N_2. Via dropping funnel DCC (8.13 g; 39.4 mmol) dissolved in dry MeCN (50.0 mL) was dropped to the mixture over a period of 20 min and the mixture was stirred at room temperature overnight. The resulting white precipitate was filtered and washed with CH_2Cl_2 (3*50 mL). The organics were concentrated. Purification by column chromatography (SiO_2; CH_2Cl_2/EtOAc 8:2) yielded the product as yellow oil in 78 % (7.30 g; 15.4 mmol) yield.

R_f (SiO_2): 0.73 (CH_2Cl_2/EtOAc 9:1).

^{1}H NMR (400 MHz; $CDCl_3$; rt): δ [ppm] = 1.28 - 1.49 (m, 26 H, $COOCH_2CH_2CH_2$, NCH_2CH_2, CCH_3), 1.58 –1.65 (m, 4H, $COOCH_2CH_2$), 3.06 (dd, ^3J = 12.6, 6.5 Hz, 4H, NCH_2), 3.32 (s, 2H, $OOCCH_2COO$), 4.09 (t, ^3J = 6.5 Hz, 4H, $COOCH_2$), 4.62 (br, 2H, N*H*).

^{13}C NMR (100 MHz; $CDCL_3$; rt): δ [ppm] = 23.0, 28.0, 28.3, 29.6, 40.3, 41.5, 65.3, 79.0, 155.9, 166.6.

MS (MALDI): *m/z* = 497 $[M+H+Na]^+$.

HRMS (ESI-TOF): *m/z* calc. for $C_{23}H_{42}N_2NaO_8$: 497.283337$[M]^+$, found:497.283559.

IR(ATR; rt): $\tilde{\nu}$[cm^{-1}] (ε [$M^{-1}cm^{-1}$]) = 520, 660, 684, 790, 866, 1013, 1089, 1146, 1164, 1256, 1365, 1457, 1520, 1692, 2850, 2923, 2960.

Bis(5-((*tert*-butoxycarbonyl)amino)pentyl) malonyl –[1,0]–mono–1,2,–dihydro[60]–fullerene; S23

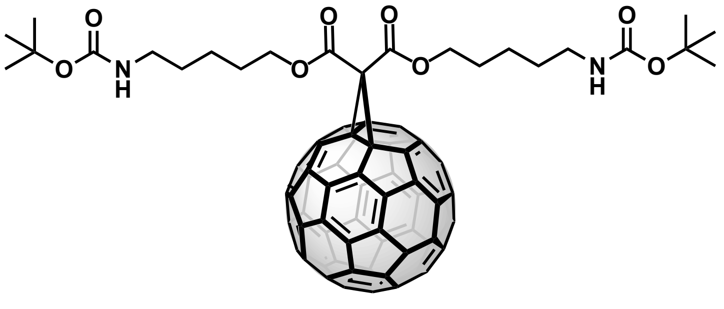

$C_{83}H_{49}N_2O_8$
M = 1202,31

A 2 L Schlenk-RBF equipped with a magnetic stir bar was charged with C_{60} (756 mg; 1.05 mmol), Bis(5-((*tert*-butoxycarbonyl)amino)pentyl) malonate **S22** (500 mg; 1.05 mmol) and iodine (199 mg; 1.57 mmol). Toluene (700 mL) was added and the mixture degassed under the exclusion of light. DBU (400 µL; 2.63 mmol) was added slowly and the mixture was stirred at room temperature for 1 h under nitrogen atmosphere. The crude product was plug filtrated (SiO_2; CH_2Cl_2/EtOAc 1:1) and concentrated. After column chromatography (SiO_2; CH_2Cl_2/EtOAc 8:2) the product was obtained as dark brown solid in 37 % (461 mg; 0.390 mmol) yield.

R_f (SiO_2): 0.27 (hexane/EtOAc 8:2).

^{1}H NMR (400 MHz; $CDCl_3$; rt): δ [ppm] = 1.41 –1.57 (m, 26 H, $COOCH_2CH_2$, NCH_2CH_2,, CCH_3), 1.81 –1.87 (m, 4H, $COOCH_2CH_2$), 3.11 (dd, ^3J = 12.6, 6.3 Hz, 4H, NCH_2), 4.47 (t, ^3J = 6.5 Hz, 4H, $COOCH_2$), 4.63 (br, 2H, N*H*).

^{13}C NMR (100 MHz; $CDCL_3$; rt): δ [ppm] = 23.0, 28.2, 28.4, 29.7, 40.4, 52.2, 67.2, 71.4, 79.1, 139.5, 141.5, 142.4, 142.8, 143.5, 143.5, 143.6, 143.8, 144.4, 145.2, 145.2, 145.4, 145.7, 145.7, 145.8, 156.0, 163.6.

MS (MALDI, dctb): *m/z* = 720 $[C_{60}]^+$, 1192 $[M]^+$.

HRMS (APPI; CH_2Cl_2): *m/z* calc. for $C_{83}H_{40}N_2NaO_8$ 1215.2677 $[M]^+$, found: 1215.2674.

IR(ATR; rt): $\tilde{\nu}$[cm^{-1}] = 527, 735, 866, 1164, 1234, 1267, 1361, 1392, 1425, 1455, 1510, 1694, 1744, 2853, 2928.

UV/Vis (; rt):λ [nm] (ε [$M^{-1}cm^{-1}$]) = 226 (28000), 257 (28000), 325 (9000), 426 (1500).

Bis(5-aminopentyl) malonyl –[1,0]–mono–1,2,–dihydro[60]–fullerene; 3

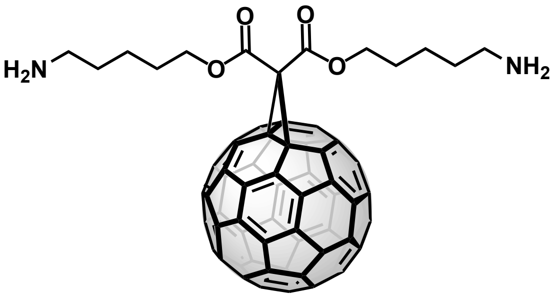

$C_{73}H_{33}N_2O_4$
M = 1002,08

A 10 mL RBF was charged with Bis(5-((*tert*-butoxycarbonyl)amino)pentyl) malonate fullerene monoadduct **S23** (305 mg;0.260 mmol) and dissolved in TFA (5 mL) under the exclusion of light. After stirring overnight, excess TFA was removed in vacuo and co-evaporated with methanol several times to give **3** as dark brown solid in 94 % (258 mg; 0.260 mmol) yield.

^{1}H NMR (400 MHz; CDCl$_3$; rt): δ [ppm] = 1.41 –1.90 (m, 12 H, COOCH$_2$CH_2, NCH$_2$CH_2,), 1.81 –1.87 (m, 4H, COOCH$_2$CH_2), 2.94 (m, 4H, NCH$_2$), 3.31 (t, ^3J = 6.5 Hz, 4H, COOCH_2), 4.59 (br, 4H, NH_2).

^{13}C NMR (100 MHz; CDCL$_3$; rt): δ [ppm] = 23.1, 28.4, 29.5, 40.7, 55.9, 68.4, 73.3, 138.9, 140.9, 141.8, 142.2, 142.2, 143.0, 143.1, 143.3, 143.9, 144.7, 144.9, 145.2, 145.2, 164.7.

MS (MALDI, dctb): *m/z* = 993 [M]$^+$.

IR(ATR; rt): $\tilde{\nu}$[cm^{-1}] = 527, 656, 744, 798, 1023, 1092, 1260, 1374, 1455, 1681, 1730, 2329, 2387, 2851, 2921, 2959.

UV/Vis (; rt):λ [nm] (ε [M^{-1}cm^{-1}]) = 226 (24000), 254 (15000), 318 (5000).

2. Spectroscopic supplementary information

Experimental. Femtosecond time-resolved transient absorption spectroscopy (fsTAS): laser sources were a Clark MXR CPA2110 and a CPA2101 Ti:Sapphire amplifier with a pulsed output of 775 nm at 1 kHz and pulse width of 150 fs. Time resolved transient absorption spectra with 150 fs resolution and time delays from 0 to 7500 ps were acquired using Ultrafast Systems HELIOS Femtosecond Transient Absorption Spectrometer. Visible white light (~400-770 nm) was generated by focusing a fraction of the fundamental 775 nm output onto a 2 mm sapphire disk; for the (near) IR (780-1500 nm), a 1 cm sapphire was used. Excitation pulses of 430 nm wavelength were generated by a NOPA with subsequent frequency doubling; a bandpass filter with ±5 nm was used to ensure low spectral width and to exclude 775 and 387 nm photons.

Ultrafast Systems EOS Sub-Nanosecond Transient Absorption Spectrometer was employed to measure transient absorption spectra with time delays of ~1 ns to 400 µs with 1 ns time resolution (nsTAS). White light (~370 to >1600 nm) was generated by a built-in photonic crystal fiber supercontinuum laser source with a fundamental of 1064 nm at 2 kHz output frequency and pulse width of approximately 1 ns.

Notes regarding on how the laser scatter was treated and how the plots were generated. In the fsTAS experiments, the pump and probe pulses are both generated from the same 775 nm light source. As a matter of fact, this leads to a strong scatter at this wavelength. Notably, the probe pulses are generated separately for the visible and the near-infrared regions. Therefore, the 775 nm fundamental scatter is a natural break in between the two probes. The scatter overloads the detectors and results in several “Not a number” / “0” data points in the range around 775 nm. In turn, target analysis fails to viable results in this particular wavelength range, which let us to introduce a break on the x-axis in the corresponding graphs. In stark contrast, for the nsTAS experiments, the probe spectrum is generated by a fiber laser with a 1064 nm fundamental and a continuous white light output from <400 to >1600 nm. Consequently, the 775 nm scatter, which dominates in the fsTAS experiments is absent in the resulting datasets. Therefore, the full range up to ~900 nm is displayed in the datasets, together with the range from 900 to ~>1500 nm. Due to inevitably imperfect focusing of the pump and probe pulses, the junction between the displays of the visible and the near-infrared regions includes some discrepancy in signal intensity. The aforementioned leads to a discontinuity at the crossover between detection ranges at ~900 nm. The only laser scatter in these datasets is, however, located around 1064 nm. Again this causes an oversaturating of the detector and a readings of ~0 in the 1030 to 1100 nm range.

All fsTAS and nsTAS experiments were corrected for the chirp and instrumental responses (IRF) with the built-in feature of glotaran software package.[§] Please note that in the nsTAS experiments the chirp is far shorter than the instrumental response time, which is caused by the 1ns pulse width of the white light laser.

Steady state UV-vis absorption and emission spectroscopy: Absorption spectra between 300 and 900 nm were measured with a Perkin-Elmer Lambda2 dual beam absorption spectrometer with a scan rate of 600 nm/min and a resolution of 0.5 nm.

Emission spectra between 400 and 850 nm were recorded with a Horiba Fluoromax with a resolution of 0.5 nm and excitation / detection spectral bandwidth of 2 nm.

Sample solutions with increasing concentrations between 10^{-8} and 10^{-6} M were titrated into 1 x 1 cm quartz glass cuvettes. For determination of fluorescence quantum yields by the comparative method, the OD at the wavelength of excitation and beyond was kept below 0.1.

[§] Glotaran software wiki webpage: http://www.timpgui.org/wiki.html by Joris Snellenburg and Sergey Laptenok under supervision of Ivo van Stokkum.

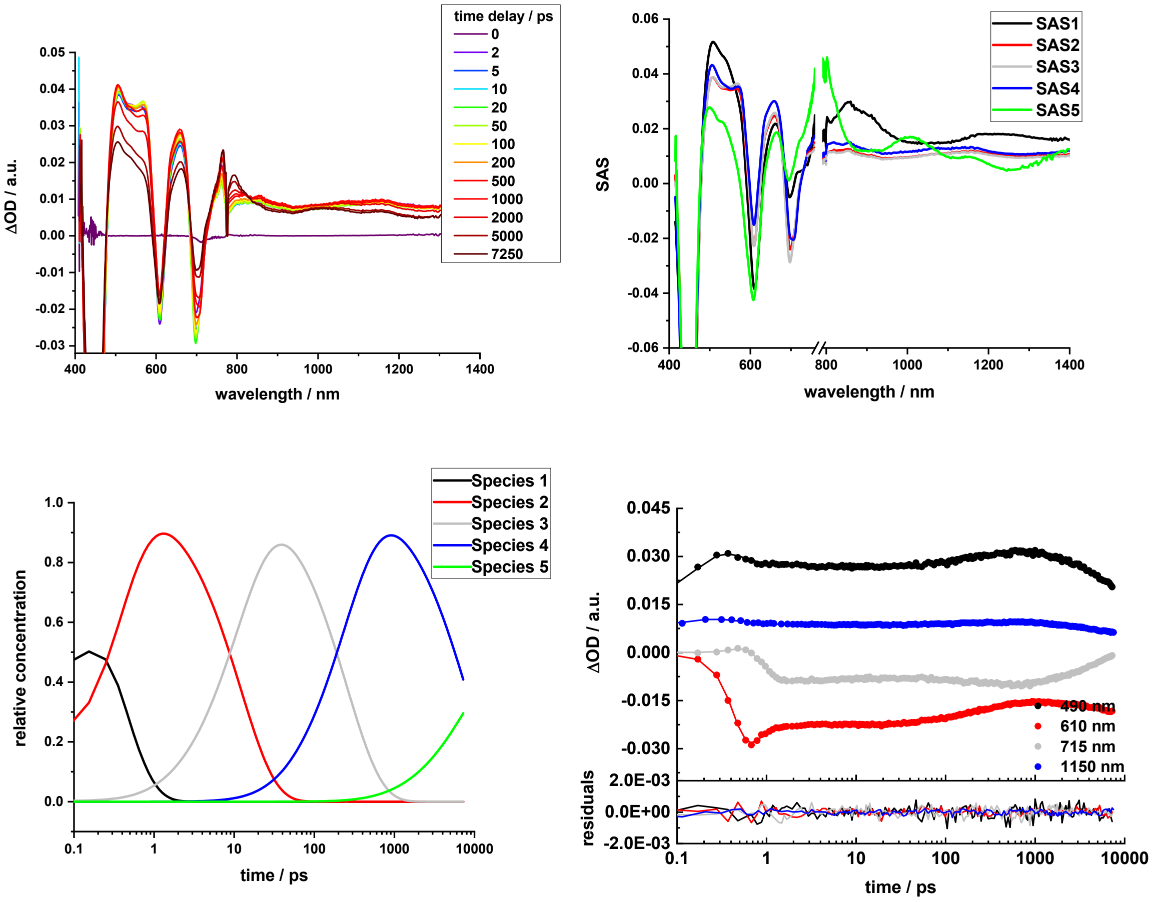

Figure S1: fsTAS analysis of **1** upon 387 nm excitation. Top left: Differential absorption changes (visible and near-infrared) obtained upon pump probe experiments; top right: deconvoluted species associated spectra derived from target analysis via GloTarAn with lifetimes of 0.35 ps (SAS1) 12.02 ps (SAS2), 254.49 ps (SAS3), 7789.81 ps (SAS4), and 1e9 ps (SAS5); bottom left: relative population of the transient species derived from target analysis; bottom right: selected time traces with fits and fit residuals resulting from target analysis. ~150 fs time resolution, solvent THF.

The differential absorption spectra of **1** are characterized by newly developing maxima at 505, 572, 660, 742, and 805 nm, a broad, featureless absorption between 1000 and 1200 nm, as well as ground state bleaching at 445, 605, and 695 nm. The 695 nm minimum coincides hereby with the bleaching stemming from stimulated fluorescence at 705 and 780 nm. Global target analysis of the differential spectra requires five species on the sub-ps to microsecond time scale to achieve negligible fit residuals (SI, Fig. S1-S4), in accordance with the theoretical results on orbital splitting. Lifetimes of the five species are 0.35, 12, 254, and 7700 ps, and >100 µs.

The resulting species-associated spectra feature the transient peaks described above and ground-state bleaching to varying degrees. The 705 nm bleaching, which corresponds to the fluorescence, is only present in the 7700 ps species, indicating that this component is the only emissive species (Figure 9, bottom). The earlier species are subject to internal conversion on a sub-nanosecond time scale and populate the lowest, fluorescent singlet excited state of **1**. The lifetime of the latter is 7700 ps and reflects the intersystem crossing to afford the corresponding triplet excited state.

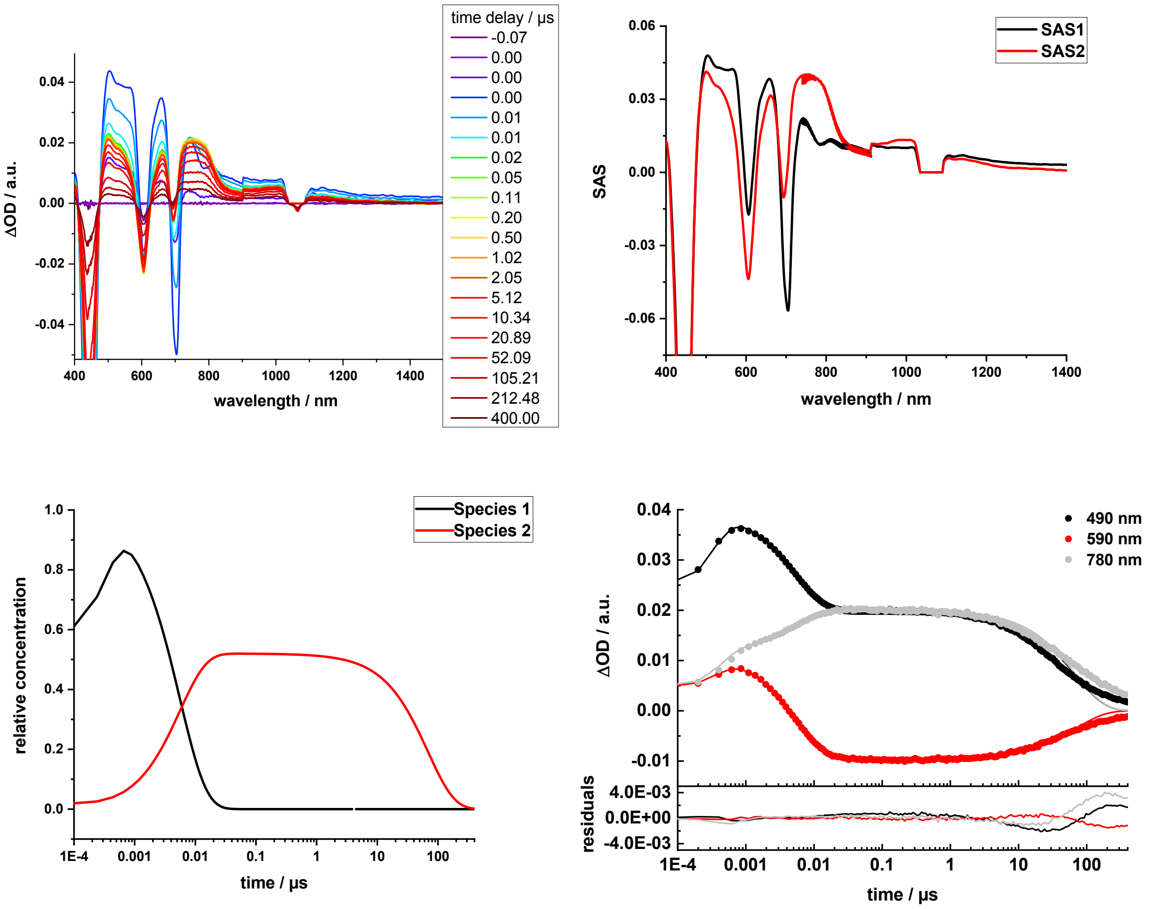

Figure S2: nsTAS analysis of **1** upon 387 nm excitation. Top left: Differential absorption changes (visible and near-infrared) obtained upon pump probe experiments; top right: deconvoluted species associated spectra derived from target analysis via GloTarAn with lifetimes of 7.7 ns (SAS1) and 68.64 µs (SAS2); bottom left: relative population of the transient species derived from target analysis; bottom right: selected time traces with fits and fit residuals resulting from target analysis. <1 ns time resolution, solvent THF.

The triplet-excited state persists for nearly hundreds of microseconds. Its lifetime is limited by diffusion-controlled triplet-triplet annihilation with either **1** or residual molecular oxygen.

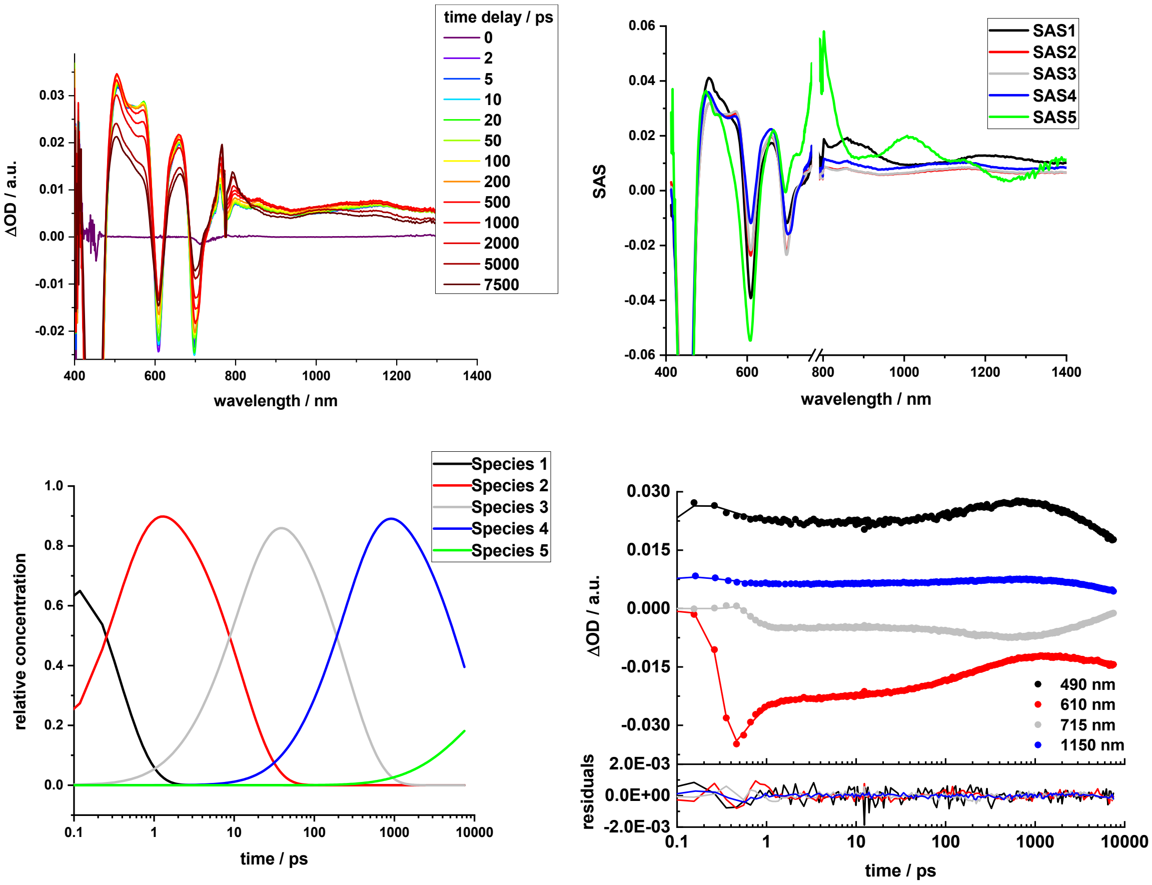

Figure S3: fsTAS analysis of **1** upon 450 nm excitation. Top left: Differential absorption changes (visible and near-infrared) obtained upon pump probe experiments; top right: deconvoluted species associated spectra derived from target analysis via GloTarAn with lifetimes of 0.35 ps (SAS1), 12.02 ps (SAS2), 254.49 ps (SAS3), 7789.81 ps (SAS4), and >>10 ns (SAS5); bottom left: relative population of the transient species derived from target analysis; bottom right: selected time traces with fits and fit residuals resulting from target analysis. ~150 fs time resolution, solvent THF.

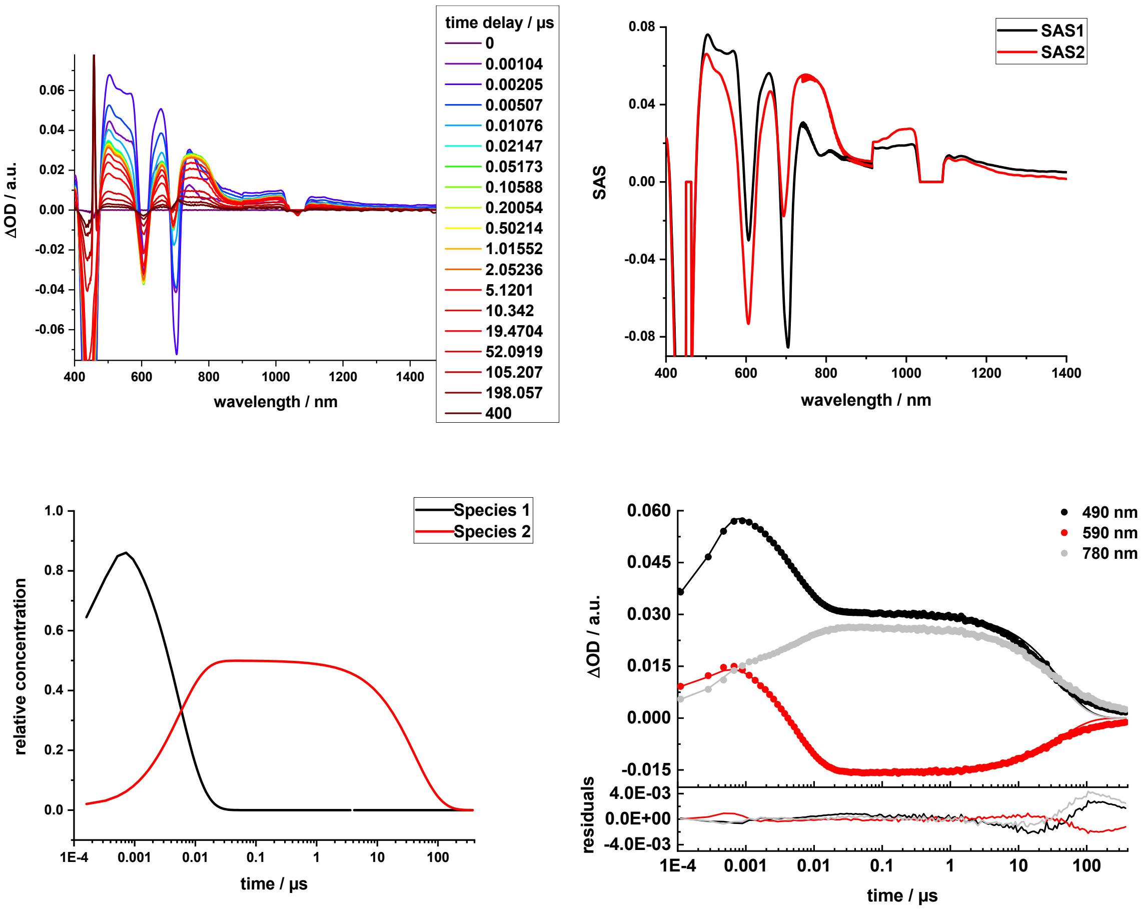

Figure S4: nsTAS analysis of **1** upon 450 nm excitation. Top left: Differential absorption changes (visible and near-infrared) obtained upon pump probe experiments; top right: deconvoluted species associated spectra derived from target analysis via GloTarAn with lifetimes of 7.7 ns (SAS1) and 41.68 µs (SAS2); bottom left: relative population of the transient species derived from target analysis; bottom right: selected time traces with fits and fit residuals resulting from target analysis. <1 ns time resolution, solvent THF.

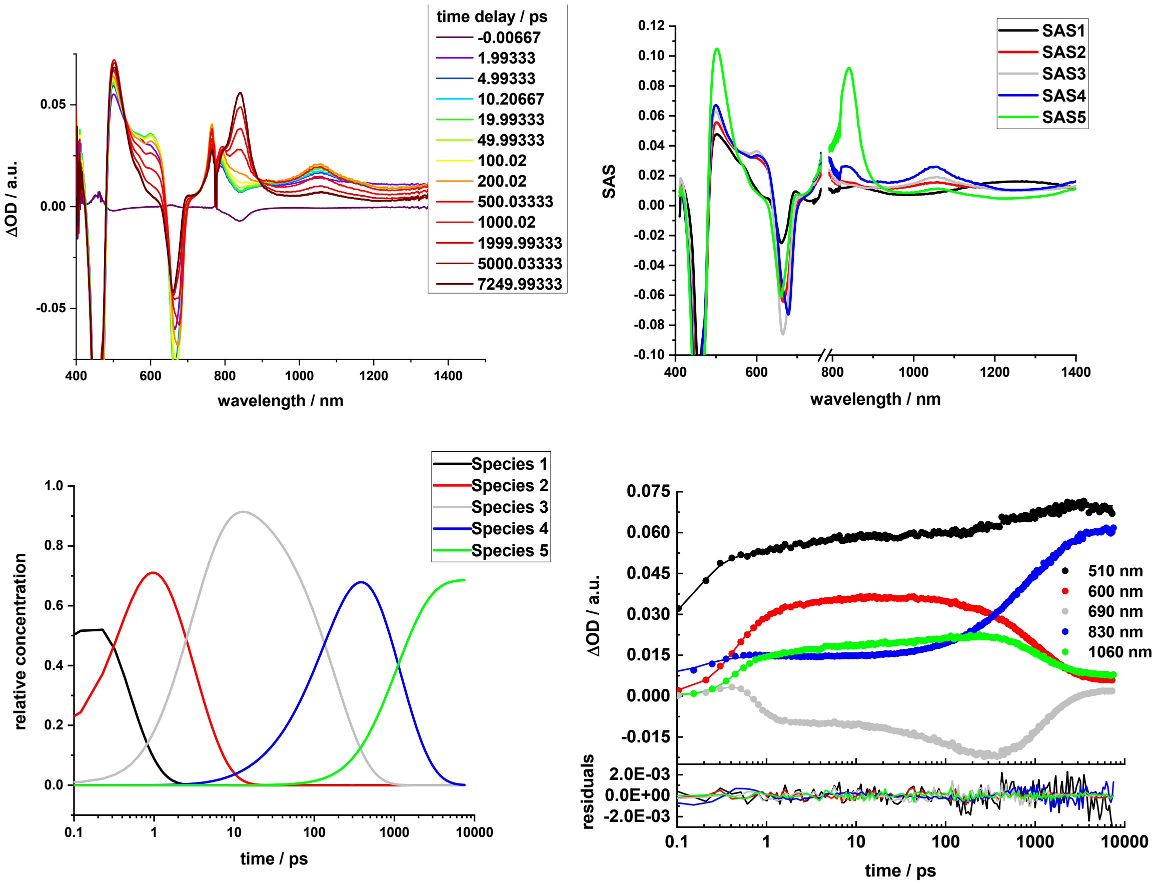

Figure S5: fsTAS analysis of **1-Zn** upon 387 nm excitation. Top left: Differential absorption changes (visible and near-infrared) obtained upon pump probe experiments; top right: deconvoluted species associated spectra derived from target analysis via GloTarAn with lifetimes of 0.40 ps (SAS1), 3.00 ps (SAS2), 179.66 ps (SAS3), 1038.01 ps (SAS4), and >>10 ns (SAS5); bottom left: relative population of the transient species derived from target analysis; bottom right: selected time traces with fits and fit residuals resulting from target analysis. ~150 fs time resolution, solvent THF.

For **1-Zn**, the differential absorption spectra (SI, Figs. S5-S9) give rise to 495, 563, 605, 768, and 1055 nm maxima. They go hand-in-hand with ground-state bleaching and stimulated emission at 460, 660, 675, and 730 nm, respectively. As for **1**, the global target analysis on the sub-ps to microsecond time scale required a five-species model with lifetimes of 0.7, 10, 170, 1000 ps and ~200µs. The transient minimum related to fluorescence is discernible in the 1000 ps species.

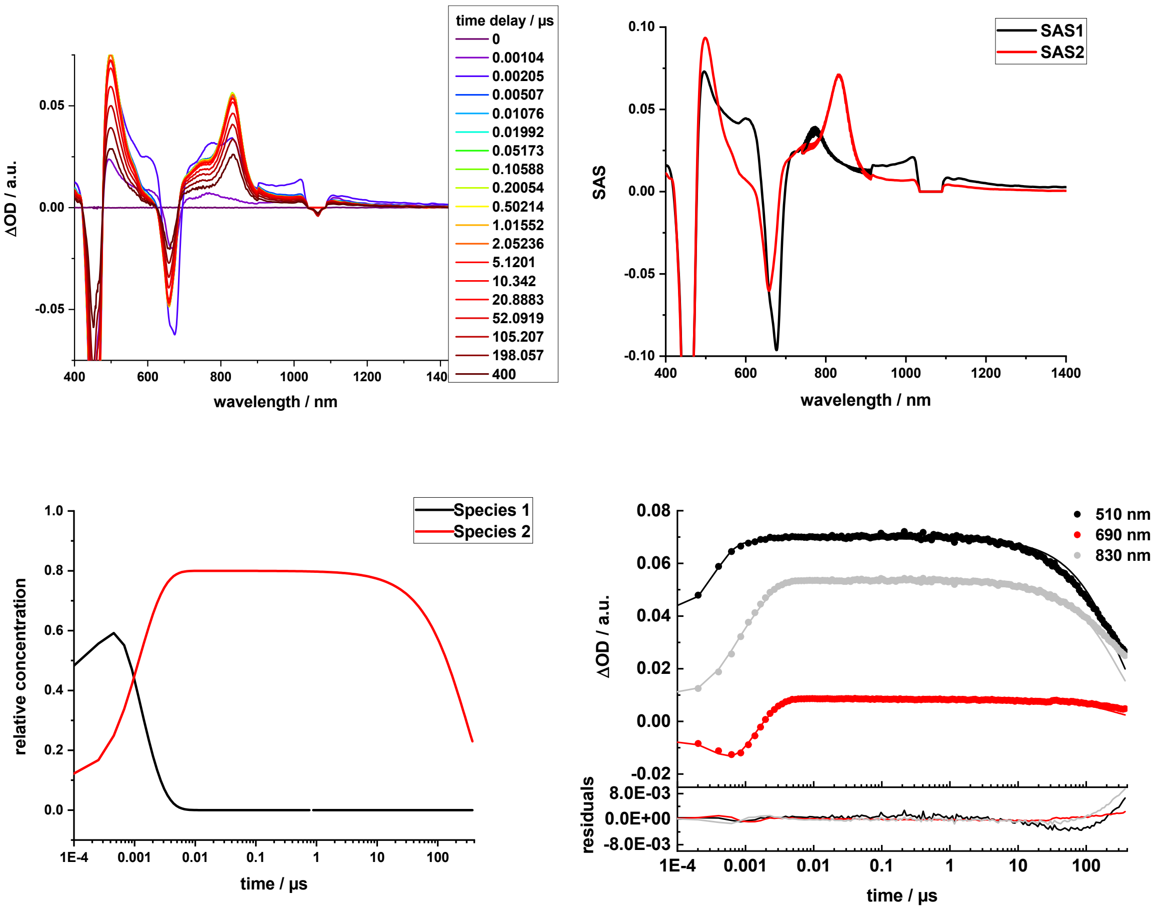

Figure S6: nsTAS analysis of **1-Zn** upon 387 nm excitation. Top left: Differential absorption changes (visible and near-infrared) obtained upon pump probe experiments; top right: deconvoluted species associated spectra derived from target analysis via GloTarAn with lifetimes of 1.14 ns (SAS1) and 298.96 µs (SAS2); bottom left: relative population of the transient species derived from target analysis; bottom right: selected time traces with fits and fit residuals resulting from target analysis. <1 ns time resolution, solvent THF.

The triplet excited-state features comprise maxima at 500 and 833 nm, a shoulder at 750 nm, and a broad band around 1060 nm. The triplet decays to the ground state with a lifetime of about 200 µs, again limited by diffusion-controlled triplet-triplet annihilation with **1-Zn** or oxygen molecules.

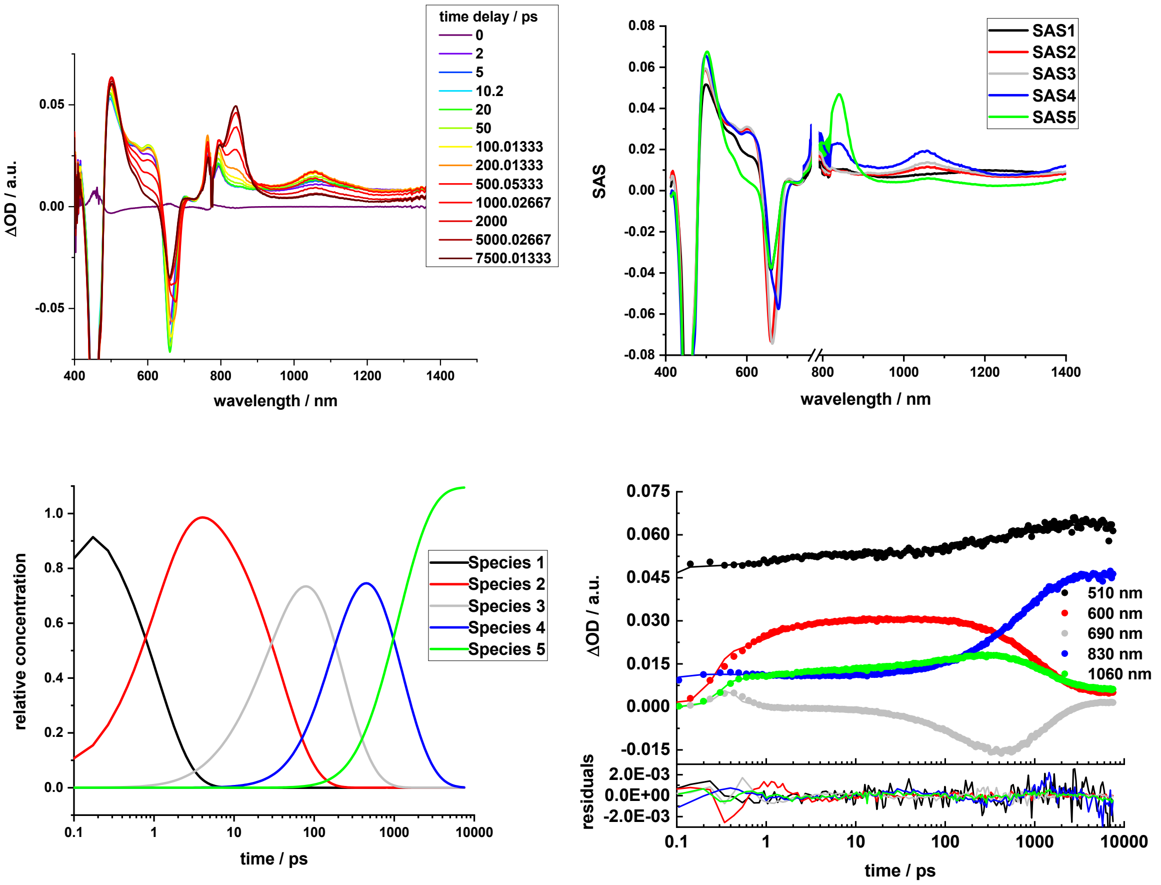

Figure S7: fsTAS analysis of **1-Zn** upon 450 nm excitation. Top left: Differential absorption changes (visible and near-infrared) obtained upon pump probe experiments; top right: deconvoluted species associated spectra derived from target analysis via GloTarAn with lifetimes of 0.72 ps (SAS1), 13.70 ps (SAS2), 172.41 ps (SAS3), 1063.31 ps (SAS4), and >>10 ns (SAS5); bottom left: relative population of the transient species derived from target analysis; bottom right: selected time traces with fits and fit residuals resulting from target analysis. ~150 fs time resolution, solvent THF.

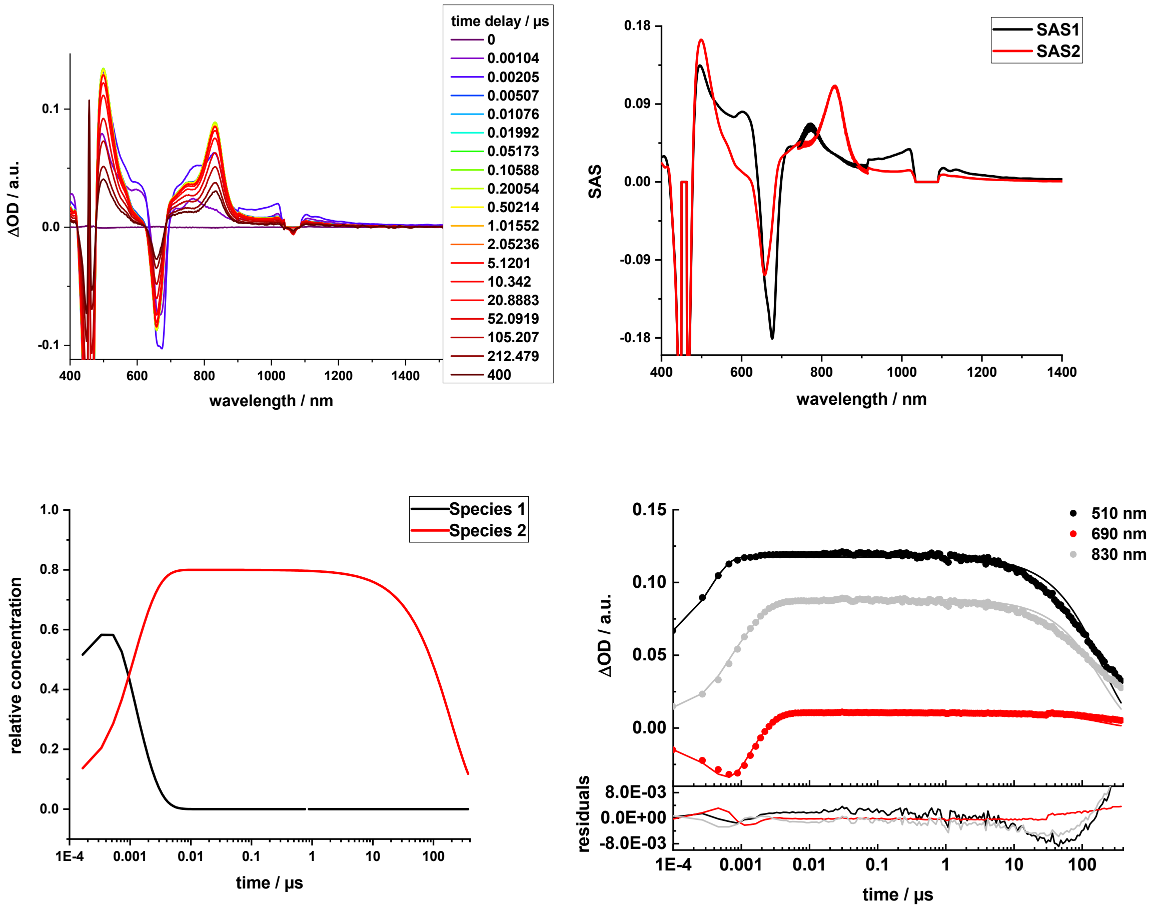

Figure S8: nsTAS analysis of **1-Zn** upon 450 nm excitation. Top left: Differential absorption changes (visible and near-infrared) obtained upon pump probe experiments; top right: deconvoluted species associated spectra derived from target analysis via GloTarAn with lifetimes of 1.11 ns (SAS1) and 194.69 µs (SAS2); bottom left: relative population of the transient species derived from target analysis; bottom right: selected time traces with fits and fit residuals resulting from target analysis. <1 ns time resolution, solvent THF.

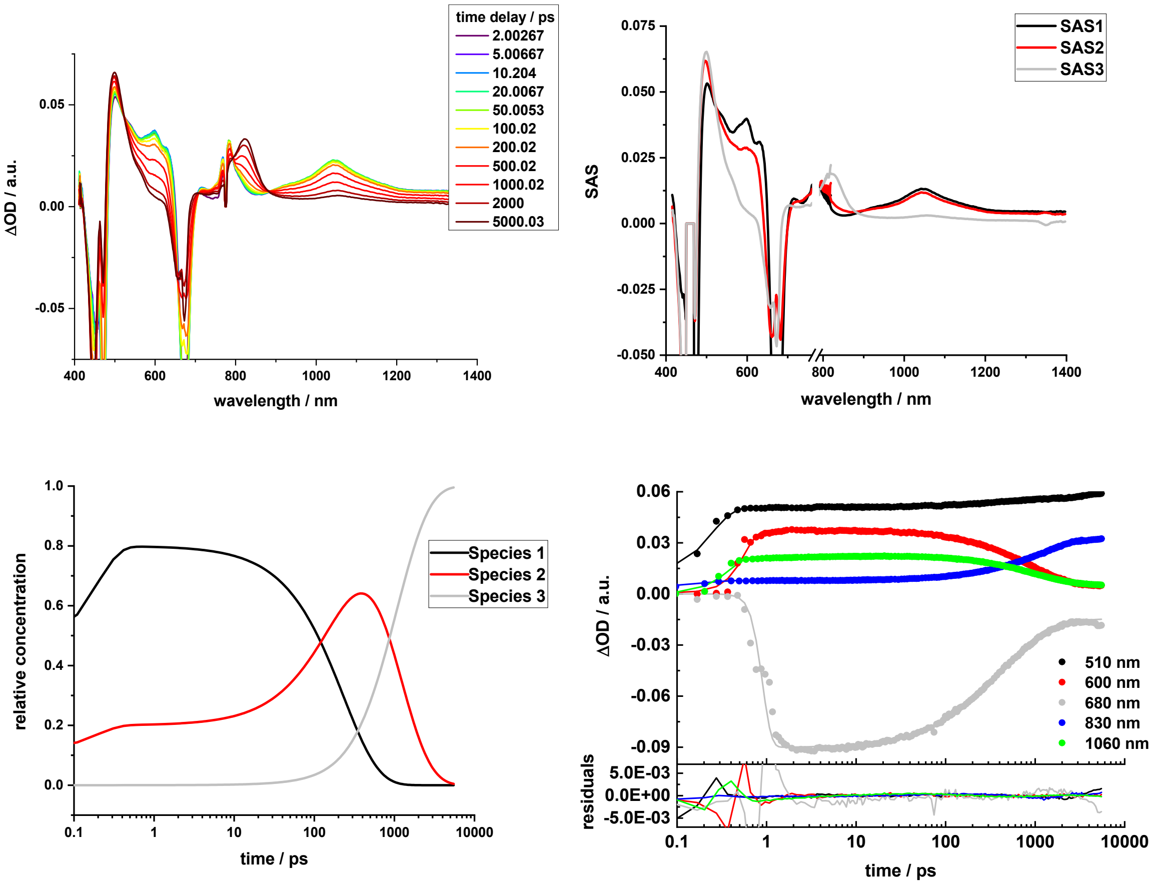

Figure S9: fsTAS analysis of **1-Zn** upon 676 nm excitation. Top left: Differential absorption changes (visible and near-infrared) obtained upon pump probe experiments; top right: deconvoluted species associated spectra derived from target analysis via GloTarAn with lifetimes of 233.86 ps (SAS1), 976.08 ps (SAS2), and >>10 ns (SAS3); bottom left: relative population of the transient species derived from target analysis; bottom right: selected time traces with fits and fit residuals resulting from target analysis. ~150 fs time resolution, solvent THF.

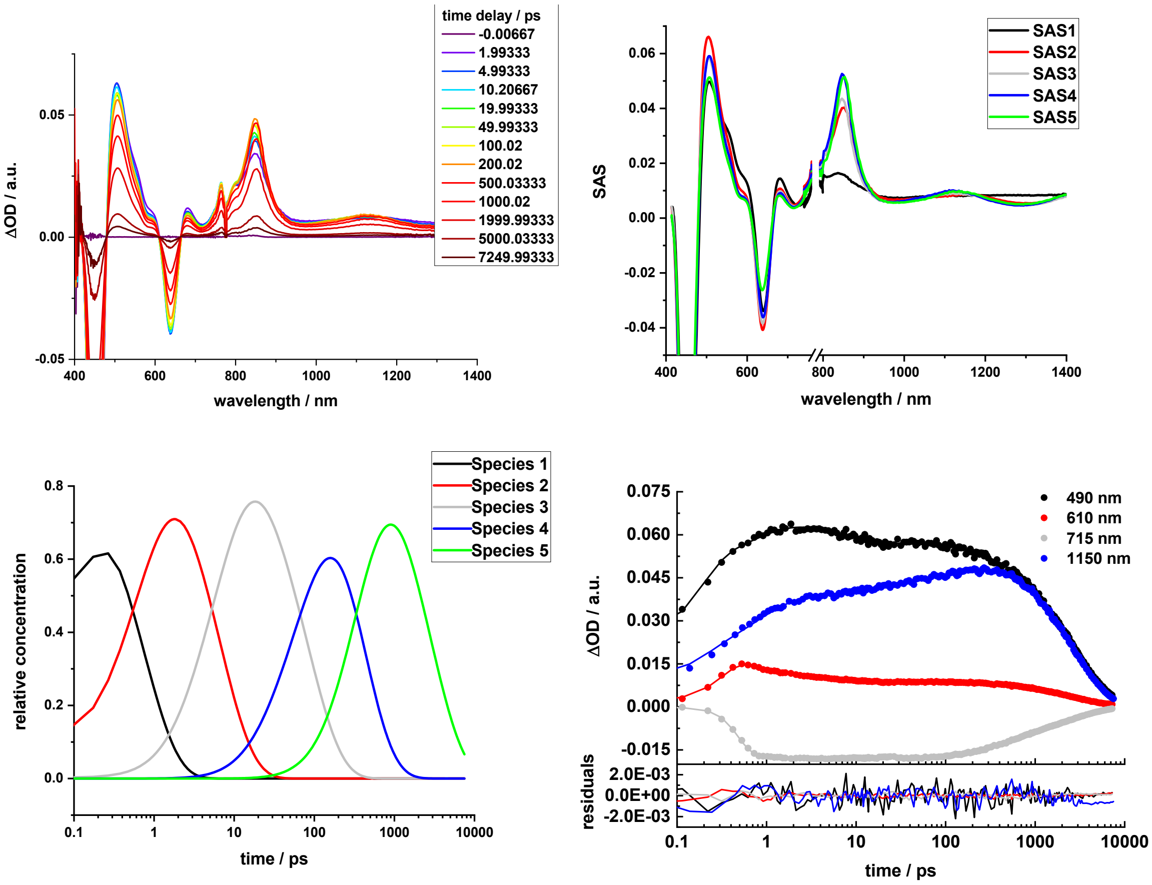

Figure S10: fsTAS analysis of **1-Cu** upon 387 nm excitation. Top left: Differential absorption changes (visible and near-infrared) obtained upon pump probe experiments; top right: deconvoluted species associated spectra derived from target analysis via GloTarAn with lifetimes of 0.73 ps (SAS1) 6.35 ps (SAS2), 80.01 ps (SAS3), 339.46 ps (SAS4), and 2656.16 ps (SAS5); bottom left: relative population of the transient species derived from target analysis; bottom right: selected time traces with fits and fit residuals resulting from target analysis. ~150 fs time resolution, solvent THF.

The differential absorption spectrum of **1-Cu** (SI, Figs S10-S13) indicates much faster deactivation of its excited states due both to the open-shell character of the central metal and its heavy-atom effect. Both enhance the intersystem crossing by spin-orbit coupling. The system therefore reverts to the ground state orders of magnitude faster than **1** and **1-Zn**. Nevertheless, the sub-nanosecond dynamics require the use of five species for reasonable fitting results. The lifetimes derived from the global target analysis are 0.5, 5, 80, 300, and 2600 ps. The transients closely resemble those of **1-Zn**, with maxima at 505 and 842 nm, complemented by a shoulder at 765 and a broad maximum around 1130 nm. Minima caused by ground-state bleaching are discernable at 450 and 638 nm.

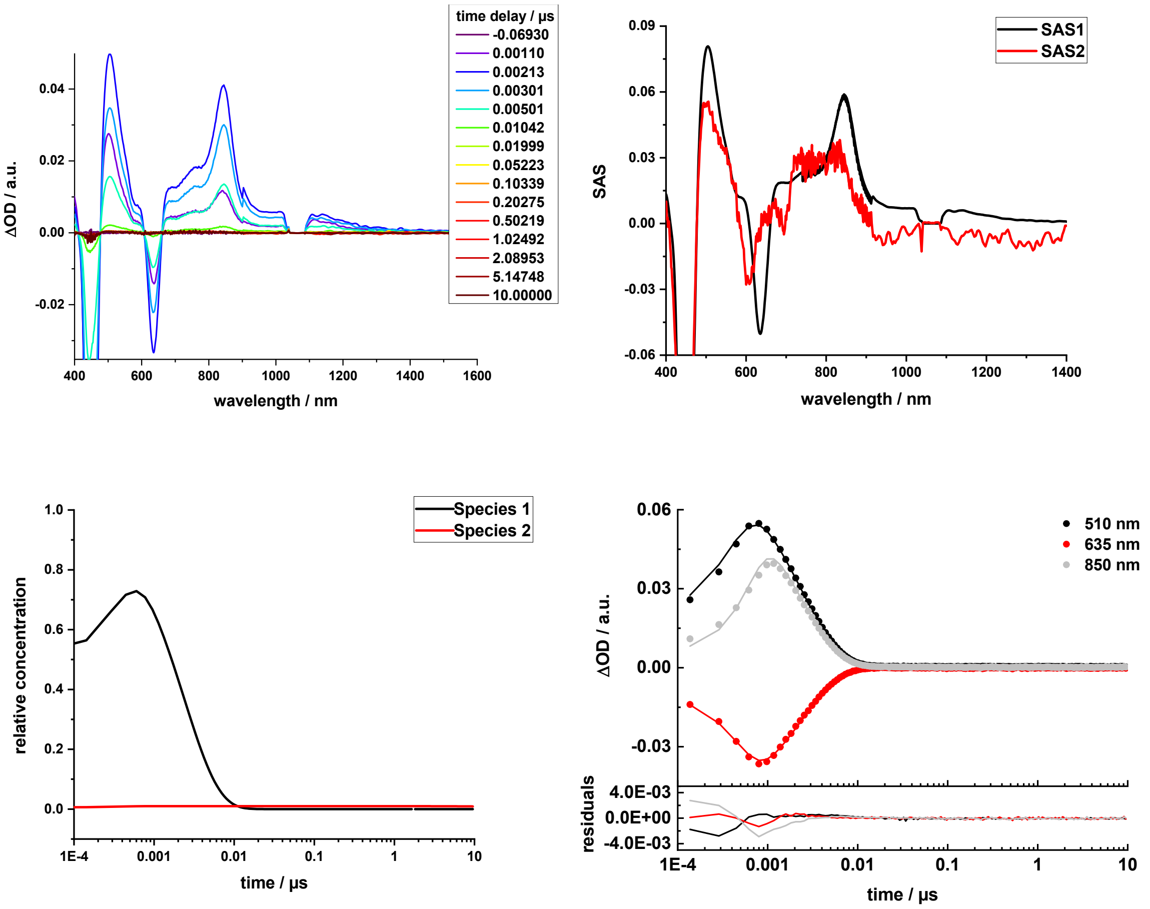

Figure S11: nsTAS analysis of **1-Cu** upon 387 nm excitation. Top left: Differential absorption changes (visible and near-infrared) obtained upon pump probe experiments; top right: deconvoluted species associated spectra derived from target analysis via GloTarAn with lifetimes of 2.38 ns (SAS1) and 100 µs (SAS2); bottom left: relative population of the transient species derived from target analysis; bottom right: selected time traces with fits and fit residuals resulting from target analysis. <1 ns time resolution, solvent THF.

Notably, a very weak and long-lived transient is visible on the sub-ns to µs time scale following the complete decay of the Cu-porphyrin triplet excited state. Its species-associated spectrum matches the features of **1** triplet excited state and is thus attributed to the presence of a very minor impurity of non-metallated porphyrin.

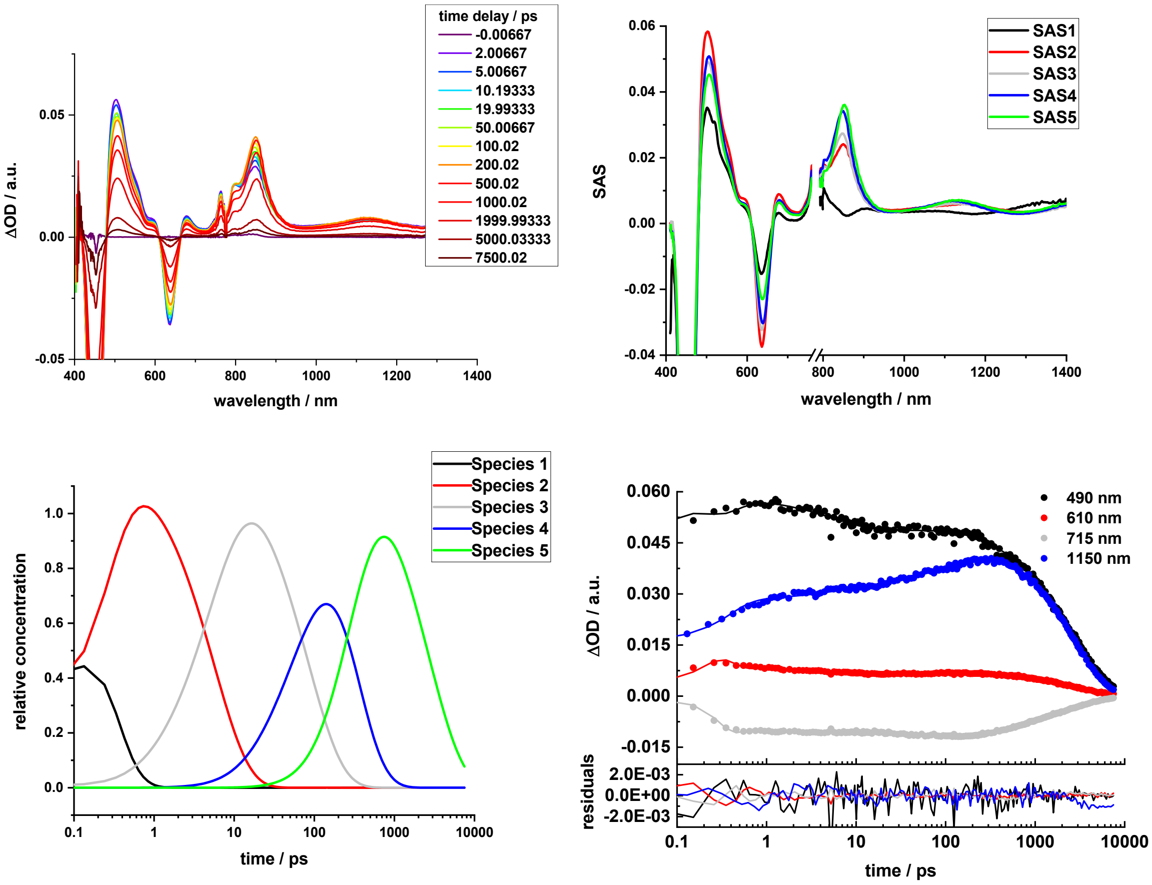

Figure S12: fsTAS analysis of **1-Cu** upon 450 nm excitation. Top left: Differential absorption changes (visible and near-infrared) obtained upon pump probe experiments; top right: deconvoluted species associated spectra derived from target analysis via GloTarAn with lifetimes of 0.2 ps (SAS1) 5.63 ps (SAS2), 82.94 ps (SAS3), 241.18 ps (SAS4), and 2631.88 ps (SAS5); bottom left: relative population of the transient species derived from target analysis; bottom right: selected time traces with fits and fit residuals resulting from target analysis. ~150 fs time resolution, solvent THF.

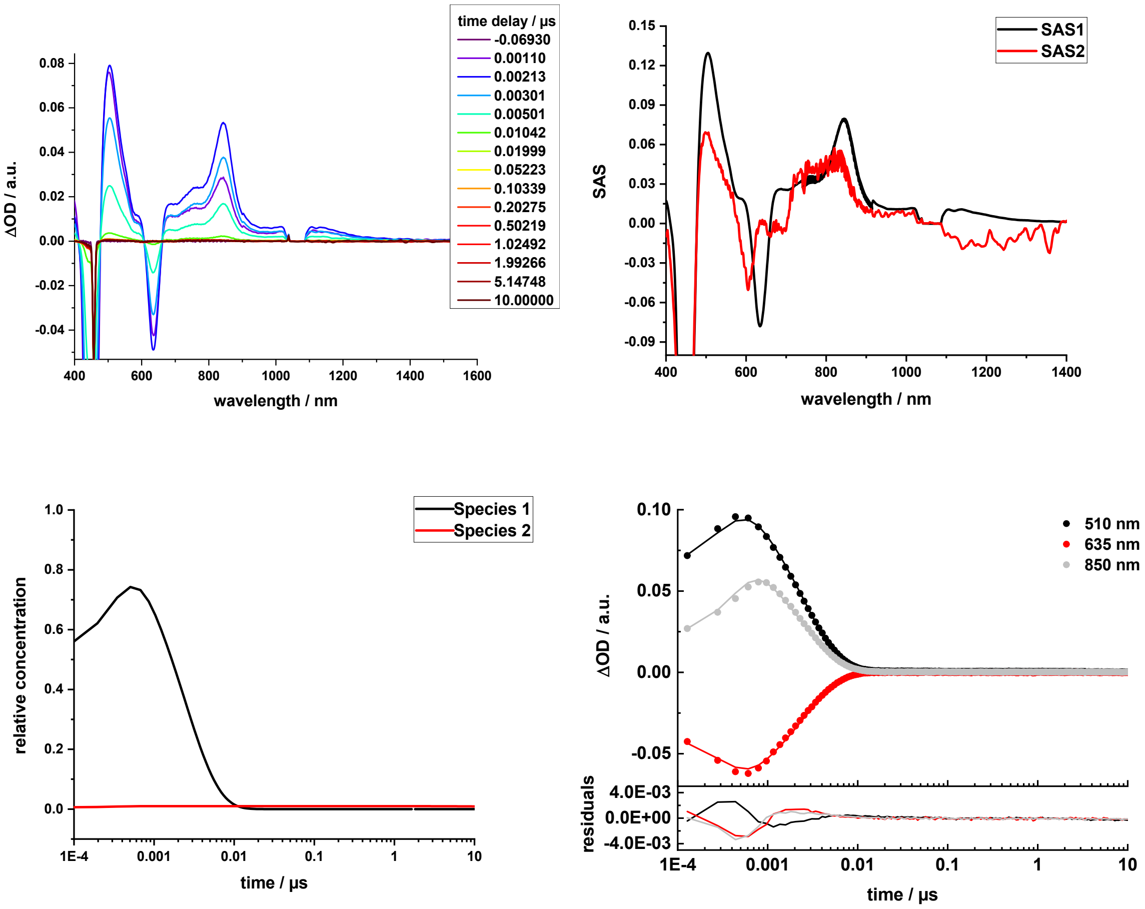

Figure S13: nsTAS analysis of **1-Cu** upon 450 nm excitation. Top left: Differential absorption changes (visible and near-infrared) obtained upon pump probe experiments; top right: deconvoluted species associated spectra derived from target analysis via GloTarAn with lifetimes of 2.40 ns (SAS1) and 100 µs (SAS2); bottom left: relative population of the transient species derived from target analysis; bottom right: selected time traces with fits and fit residuals resulting from target analysis. <1 ns time resolution, solvent THF.

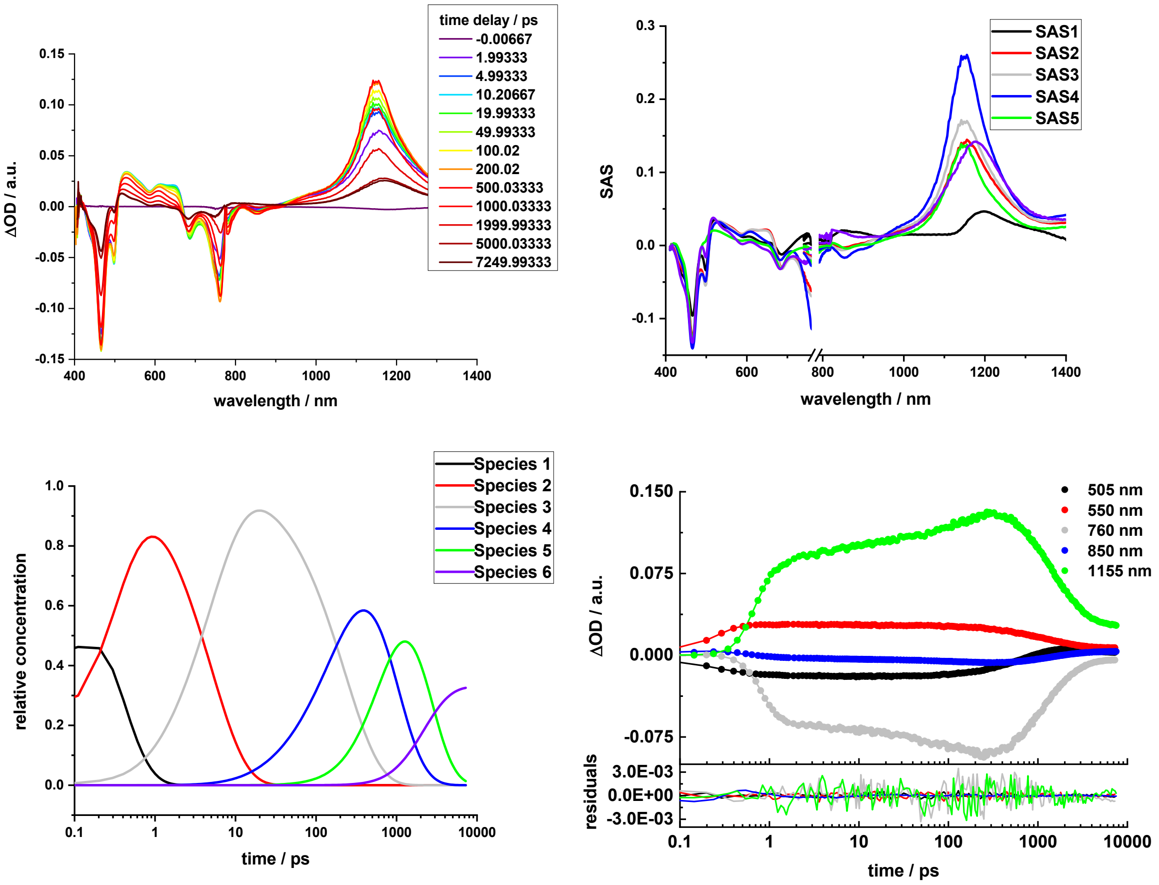

Figure S14: fsTAS analysis of **2-Zn** upon 387 nm excitation. Top left: Differential absorption changes (visible and near-infrared) obtained upon pump probe experiments; top right: deconvoluted species associated spectra derived from target analysis via GloTarAn with lifetimes of 0.30 ps (SAS1), 5.01 ps (SAS2), 228.78 ps (SAS3), 712.48 ps (SAS4), 1400.52 ps (SAS5), and >>10 ns (SAS6); bottom left: relative population of the transient species derived from target analysis; bottom right: selected time traces with fits and fit residuals resulting from target analysis. ~150 fs time resolution, solvent THF.

The differential absorption spectrum of **2-Zn** exhibits new signals starting at 400 nm and ranging all the way to 1600 nm (SI, Figs. S14-S21). Intense ground-state bleaching and stimulated fluorescence are found at 464, 495, 682, 757, and 850 nm. Maxima at 408, 522, 605, and 1142 nm are fingerprints of the singlet-excited state and transform to the longer-lived triplet excited state within about 1.5 ns. Here, the maxima are shifted to 515, 605, 795, and 1160 nm. The triplet lifetime is approximately 200 µs. The number of species needed to model the excited-state dynamics of **2-Zn** on the pico- to nanoseconds time scale depends on the excitation wavelength: Six species with lifetimes of 0.3, 5, 250, 650, 1400 ps and ~200 µs upon photoexcitation at 387 or 450 nm; five species with lifetimes of 5, 250, 650, 1400 ps and ~200 µs upon photoexcitation at 505; four species with lifetimes of 47, 350, 1200 ps, and ~200 µs after photoexcitation at 676 nm; three species with lifetimes of 250, 1200 ps and ~200 µs upon photoexcitation at 775 nm.

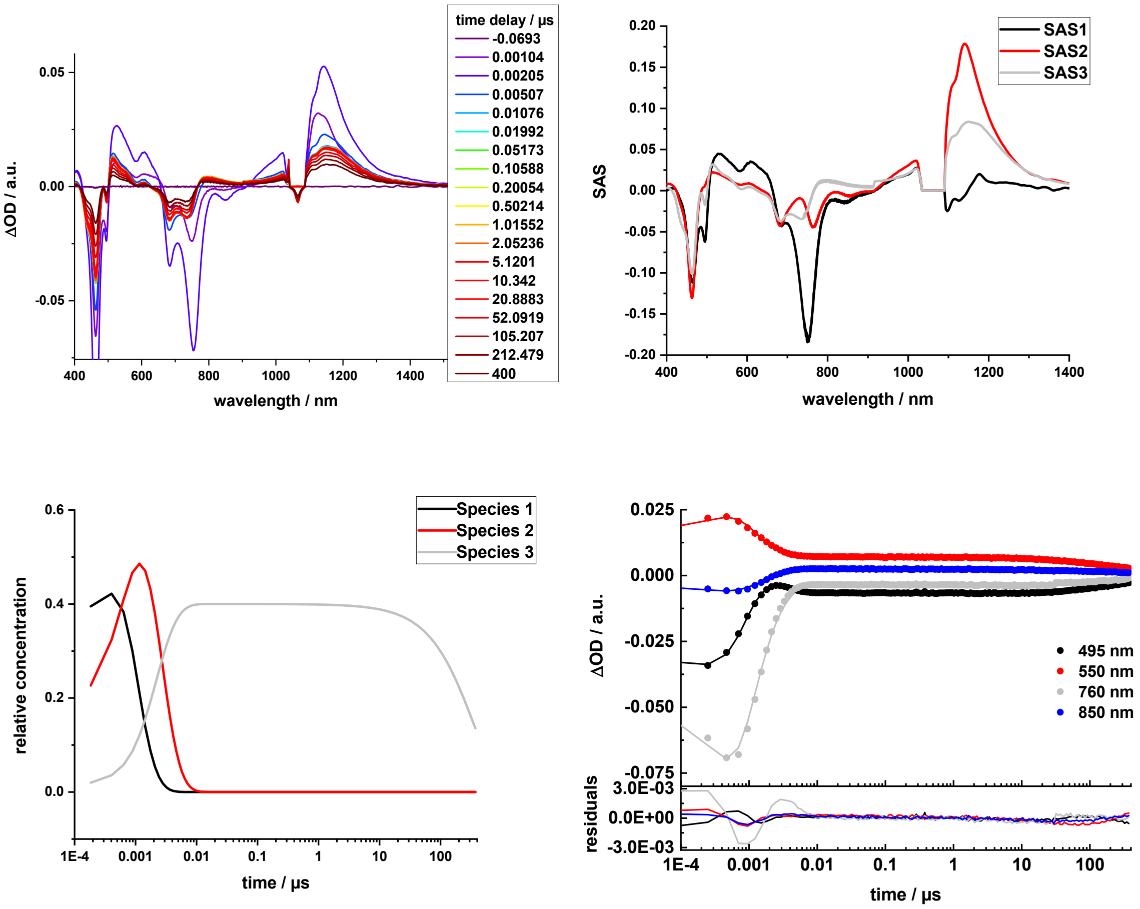

Figure S15: nsTAS analysis of **2-Zn** upon 387 nm excitation. Top left: Differential absorption changes (visible and near-infrared) obtained upon pump probe experiments; top right: deconvoluted species associated spectra derived from target analysis via GloTarAn with lifetimes of 0.67 ns (SAS1), 1.54 ns (SAS2), and 344 µs (SAS3); bottom left: relative population of the transient species derived from target analysis; bottom right: selected time traces with fits and fit residuals resulting from target analysis. <1 ns time resolution, solvent THF.

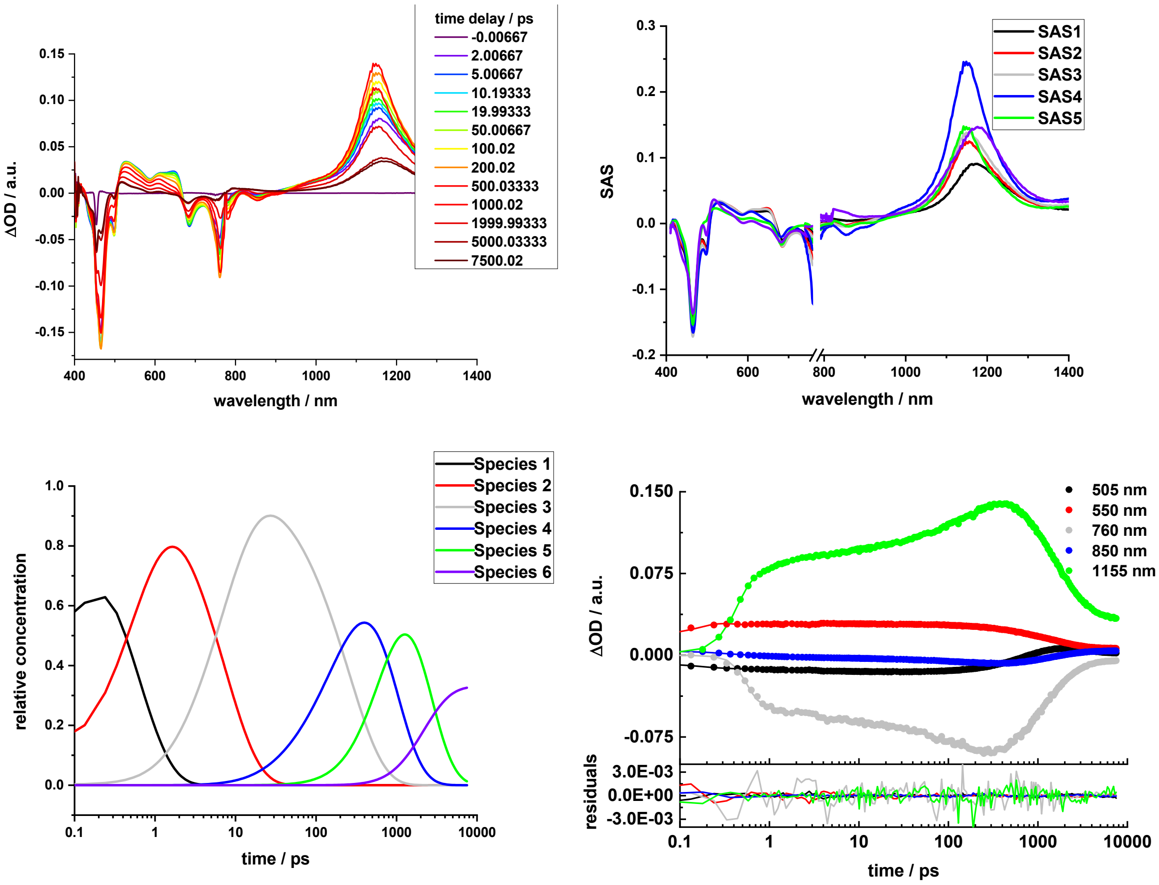

Figure S16: fsTAS analysis of **2-Zn** upon 450 nm excitation. Top left: Differential absorption changes (visible and near-infrared) obtained upon pump probe experiments; top right: deconvoluted species associated spectra derived from target analysis via GloTarAn with lifetimes of 0.60 ps (SAS1), 7.23 ps (SAS2), 254.55 ps (SAS3), 638.34 ps (SAS4), 1465.52 ps (SAS5), and >>10 ns (SAS6); bottom left: relative population of the transient species derived from target analysis; bottom right: selected time traces with fits and fit residuals resulting from target analysis. ~150 fs time resolution, solvent THF.

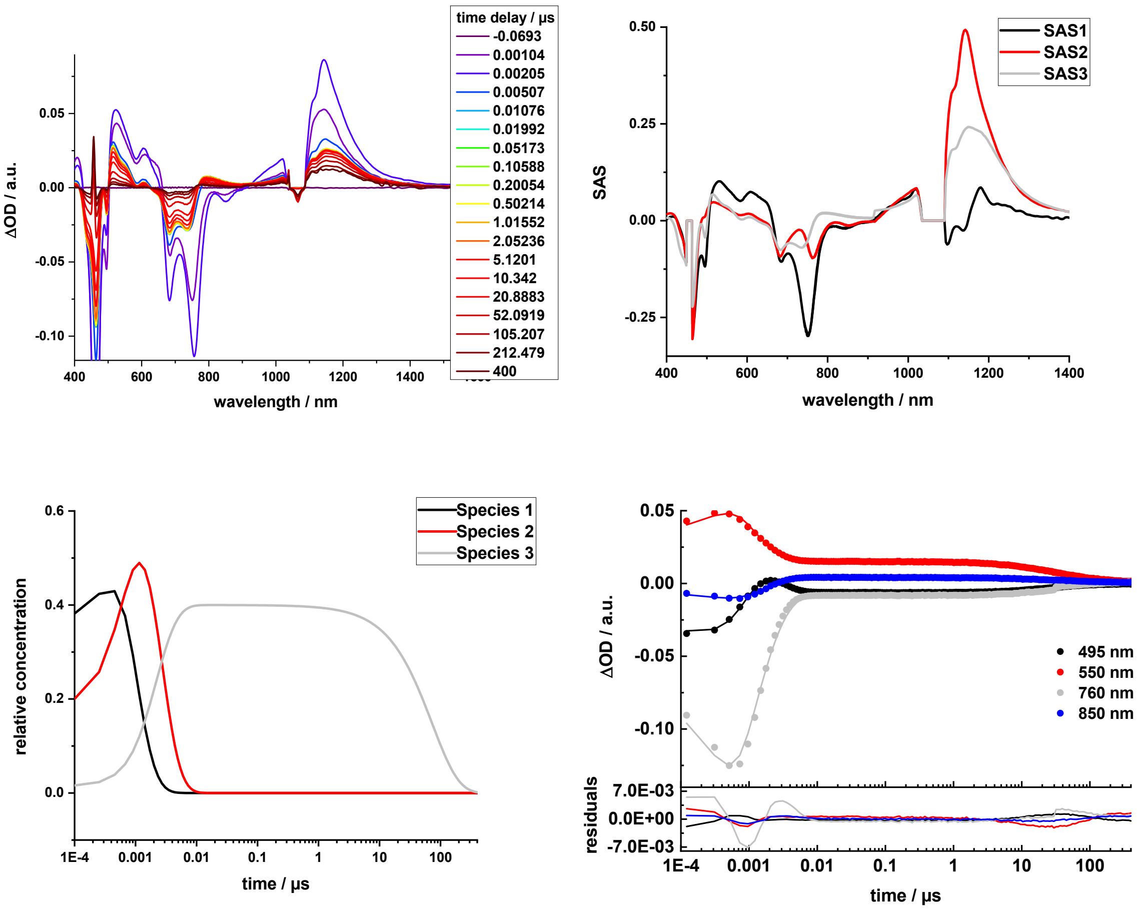

Figure S17: nsTAS analysis of **2-Zn** upon 450 nm excitation. Top left: Differential absorption changes (visible and near-infrared) obtained upon pump probe experiments; top right: deconvoluted species associated spectra derived from target analysis via GloTarAn with lifetimes of 0.67 ns (SAS1), 1.54 ns (SAS2), and 73.32 µs (SAS3); bottom left: relative population of the transient species derived from target analysis; bottom right: selected time traces with fits and fit residuals resulting from target analysis. <1 ns time resolution, solvent THF.

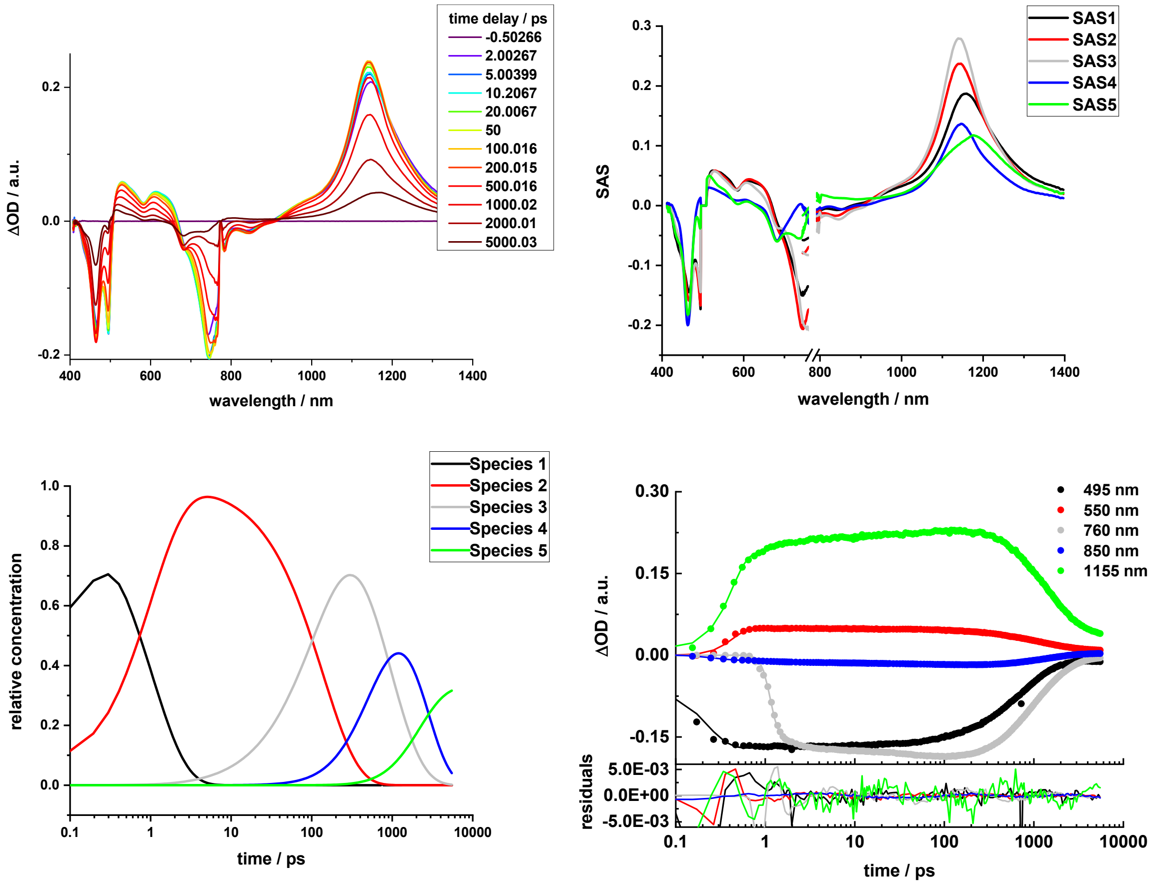

Figure S18: fsTAS analysis of **2-Zn** upon 505 nm excitation. Top left: Differential absorption changes (visible and near-infrared) obtained upon pump probe experiments; top right: deconvoluted species associated spectra derived from target analysis via GloTarAn with lifetimes of 1.03 ps (SAS1), 138.56 ps (SAS2), 850.82 ps (SAS3), 1283.93 ps (SAS4), and >>10 ns (SAS5); bottom left: relative population of the transient species derived from target analysis; bottom right: selected time traces with fits and fit residuals resulting from target analysis. ~150 fs time resolution, solvent THF.

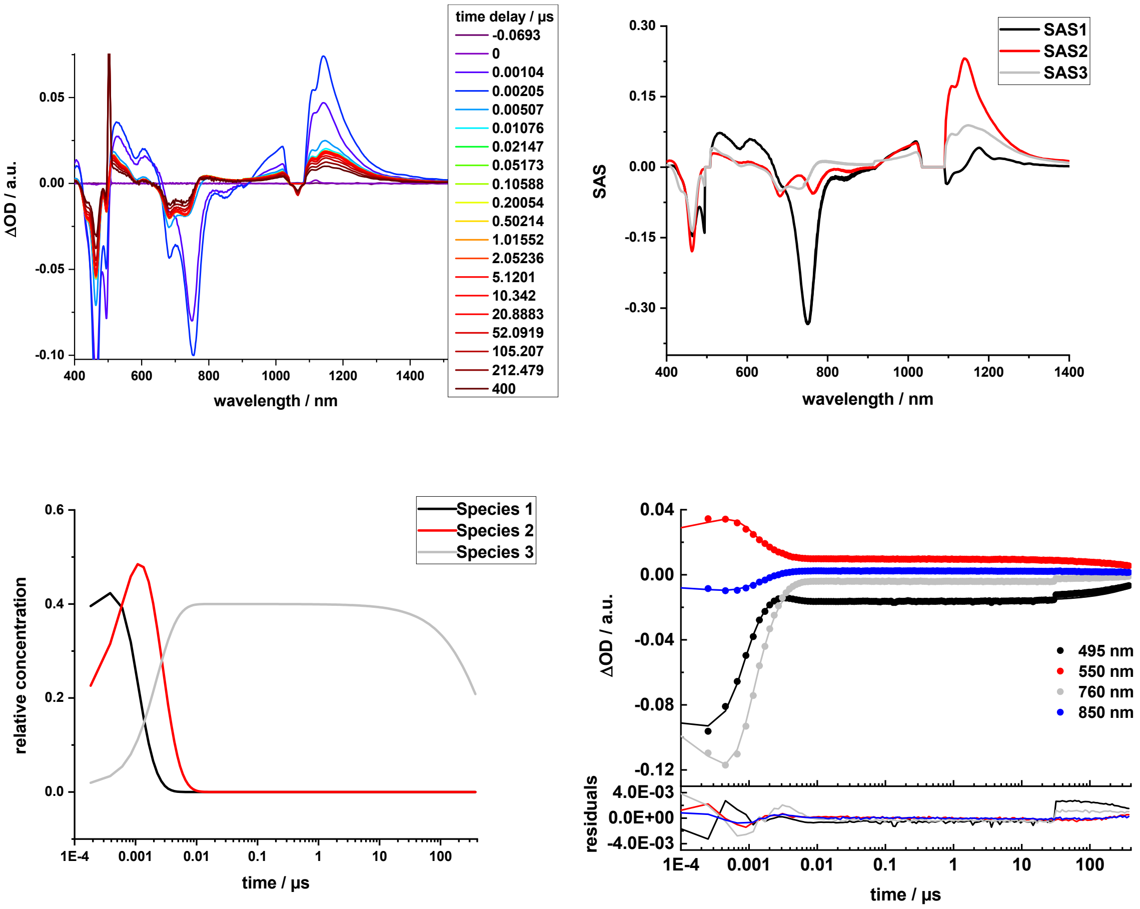

Figure S19: nsTAS analysis of **2-Zn** upon 505 nm excitation. Top left: Differential absorption changes (visible and near-infrared) obtained upon pump probe experiments; top right: deconvoluted species associated spectra derived from target analysis via GloTarAn with lifetimes of 0.67 ns (SAS1), 1.54 ns (SAS2), and 572.18 µs (SAS3); bottom left: relative population of the transient species derived from target analysis; bottom right: selected time traces with fits and fit residuals resulting from target analysis. <1 ns time resolution, solvent THF.

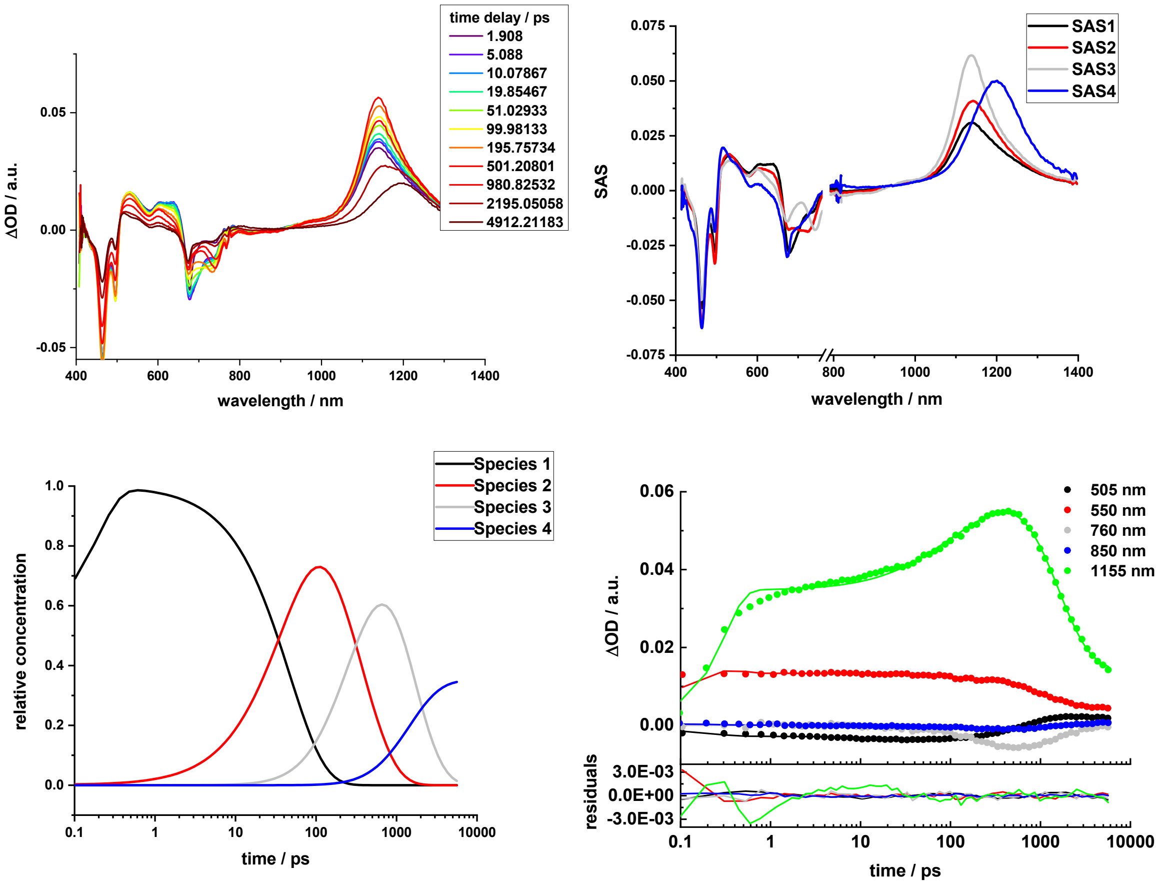

Figure S20: fsTAS analysis of **2-Zn** upon 676 nm excitation. Top left: Differential absorption changes (visible and near-infrared) obtained upon pump probe experiments; top right: deconvoluted species associated spectra derived from target analysis via GloTarAn with lifetimes of 47.37 ps (SAS1), 347.15 ps (SAS2), 1215.32 ps (SAS3), and >>10 ns (SAS4); bottom left: relative population of the transient species derived from target analysis; bottom right: selected time traces with fits and fit residuals resulting from target analysis. ~150 fs time resolution, solvent THF.

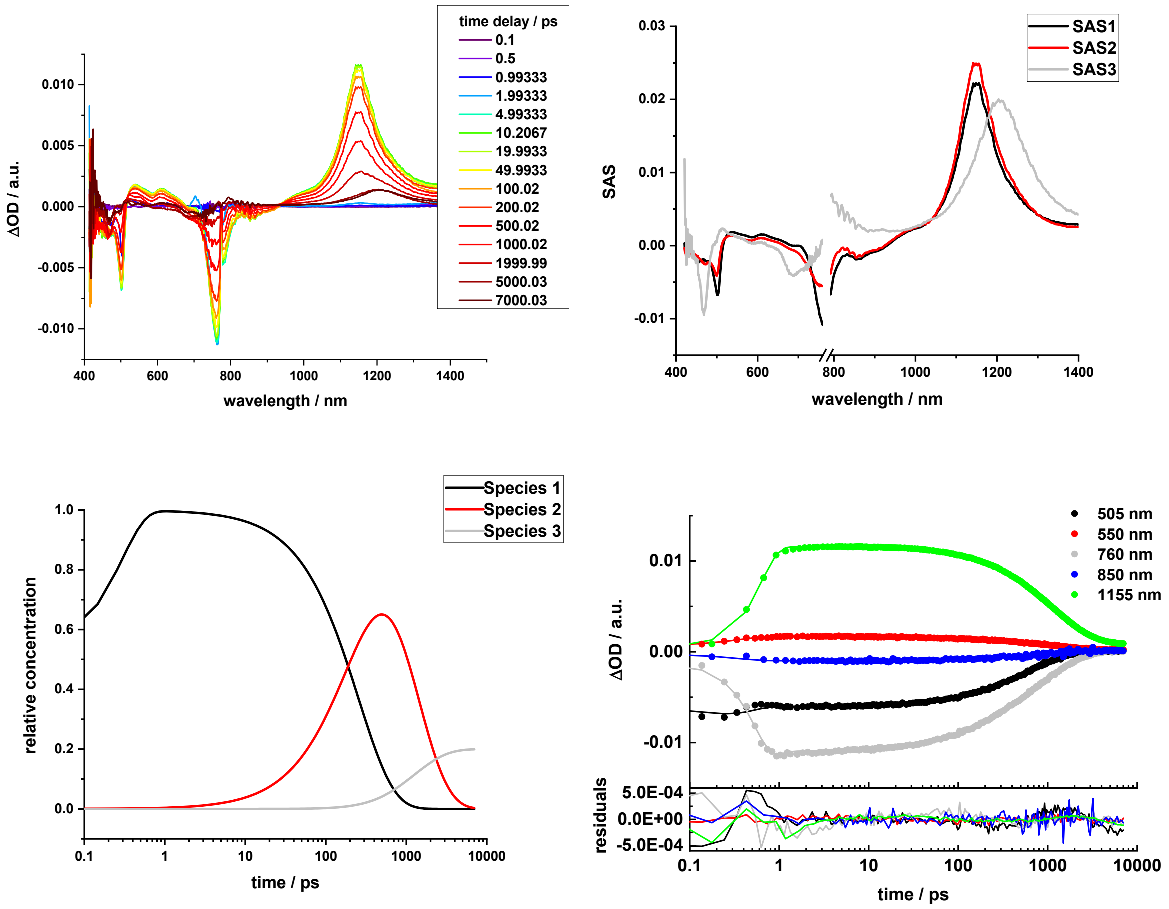

Figure S21: fsTAS analysis of **2-Zn** upon 775 nm excitation. Top left: Differential absorption changes (visible and near-infrared) obtained upon pump probe experiments; top right: deconvoluted species associated spectra derived from target analysis via GloTarAn with lifetimes of 256.04 ps (SAS1), 1150.99 ps (SAS2), and >>10 ns (SAS3); bottom left: relative population of the transient species derived from target analysis; bottom right: selected time traces with fits and fit residuals resulting from target analysis. ~150 fs time resolution, solvent THF.

Interestingly, upon excitation at 775 nm, the ground-state bleaching in the transient absorption spectrum differs from the ground-state absorption spectra. Rather than registering minima around 460 and 730 nm as for, for example, upon excitation < 500 nm, the minima evolve at 500 and 760 nm. Instead, the bleaching fits very well with the absorption spectrum of a fully planarized **2-Zn** - vide supra. In other words, only the coplanar conformer is excited resonantly at 775 nm. Such a wavelength-selective excitation of different conformations is consistent with the phenomena described in the steady-state emission spectroscopy, the results of our calculations, and with the cited literature. The long-lived transient absorption features that persist after a few nanoseconds, which are assigned to the triplet excited state of **2-Zn**, are the same irrespective of excitation wavelength.

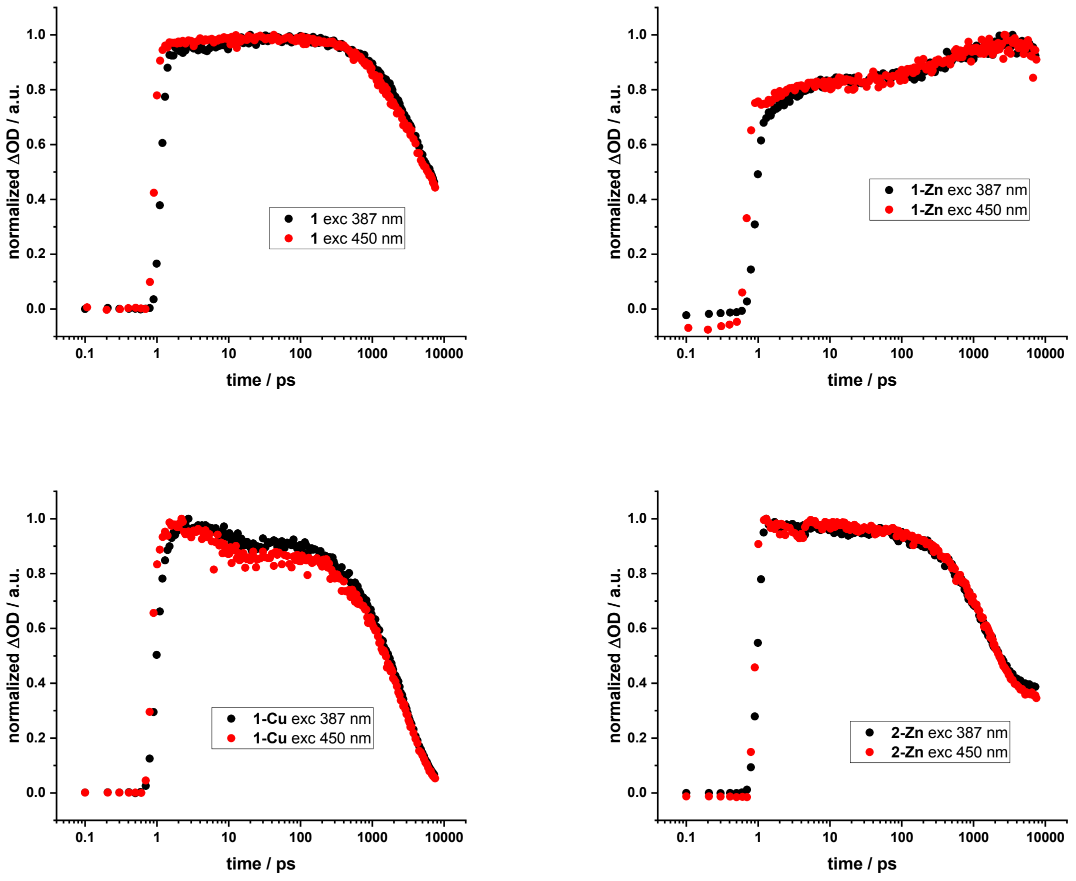

Figure S22: Comparison of selected time traces from time resolved transient absorption analysis of the first 7.5 ns with fs resolution at 387 nm or 450 nm excitation.

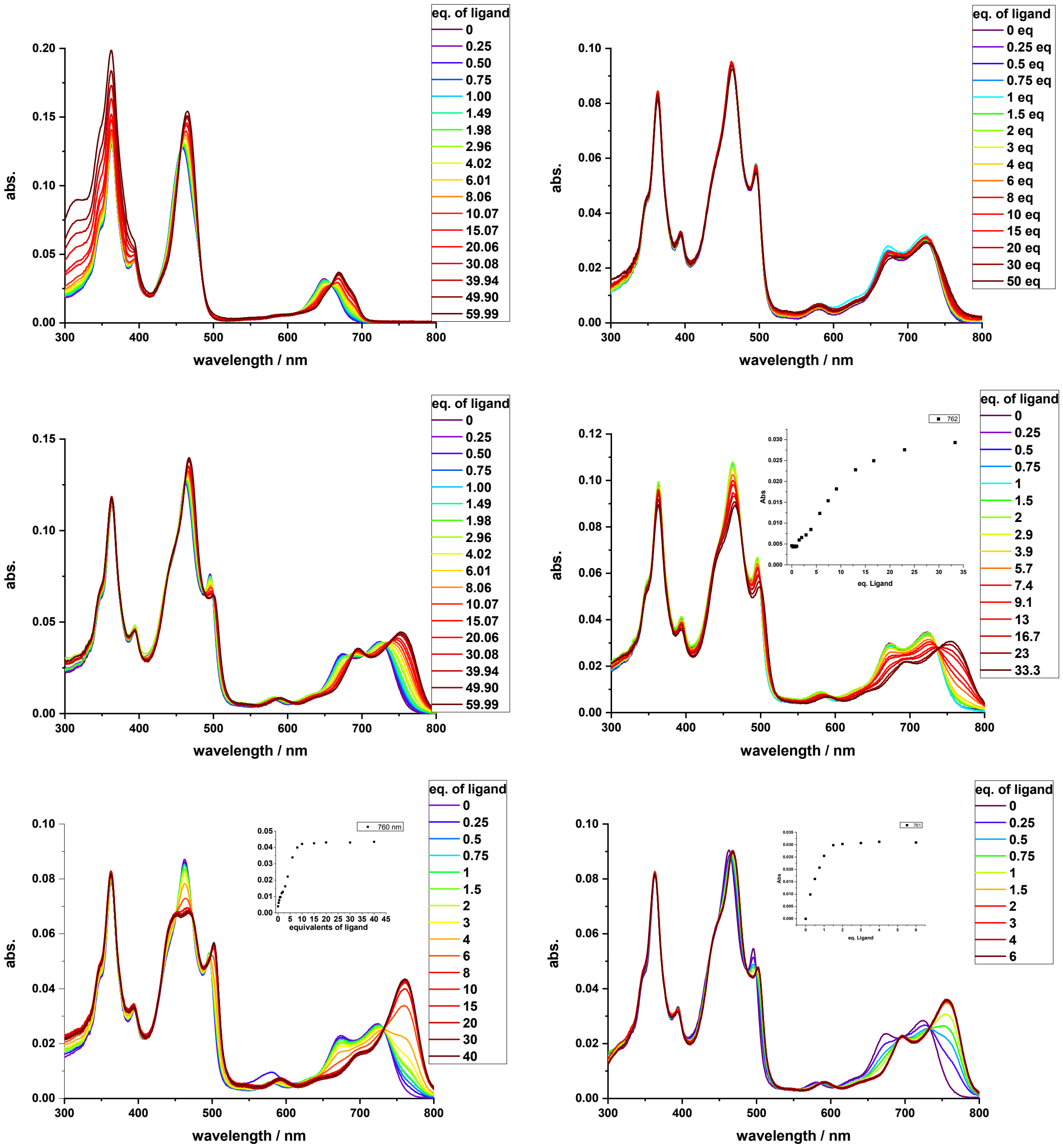

Figure S23: Steady state absorption spectra of supramolecular titration experiments. Top left: **1-Zn** + **heptylamine**; top right: **2-Zn** + **pyridine**; middle left: **2-Zn** + **heptylamine**; middle right: **2-Zn** + **1,7-diaminoheptane**; bottom left: **2-Zn** + **1,10-diaminodecane**; bottom right: **2-Zn** + **1,12-diaminododecane**.

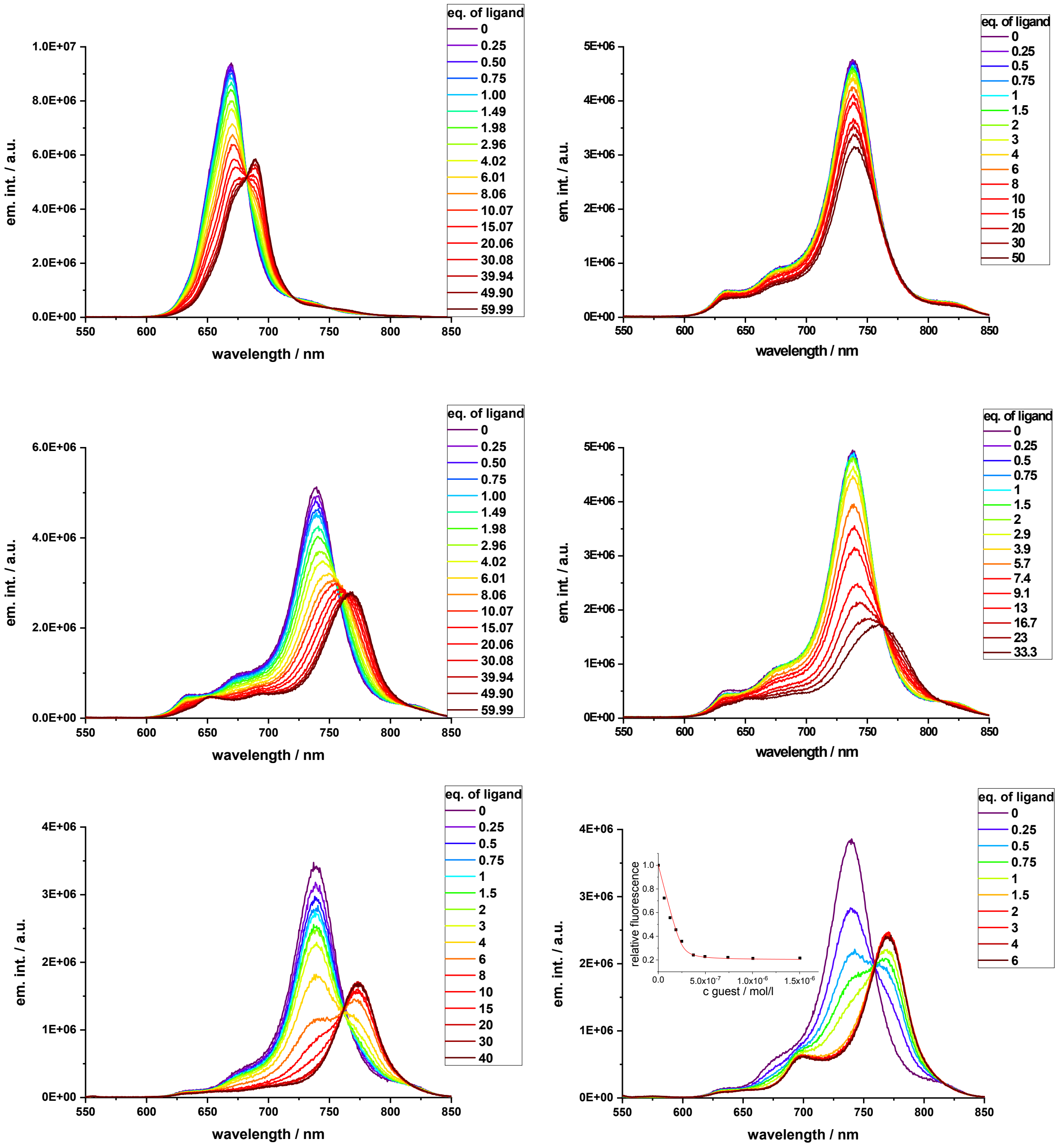

Figure S24: Steady state emission spectra of supramolecular titration experiments. Top left: **1-Zn** + **heptylamine**; top right: **2-Zn** + **pyridine**; middle left: **2-Zn** + **heptylamine**; middle right: **2-Zn** + **1,7-diaminoheptane**; bottom left: **2-Zn** + **1,10-diaminodecane**; bottom right: **2-Zn** + **1,12-diaminododecane**; inset: fit of the relative quenching of the fluorescence maximum to obtain the binding constant.

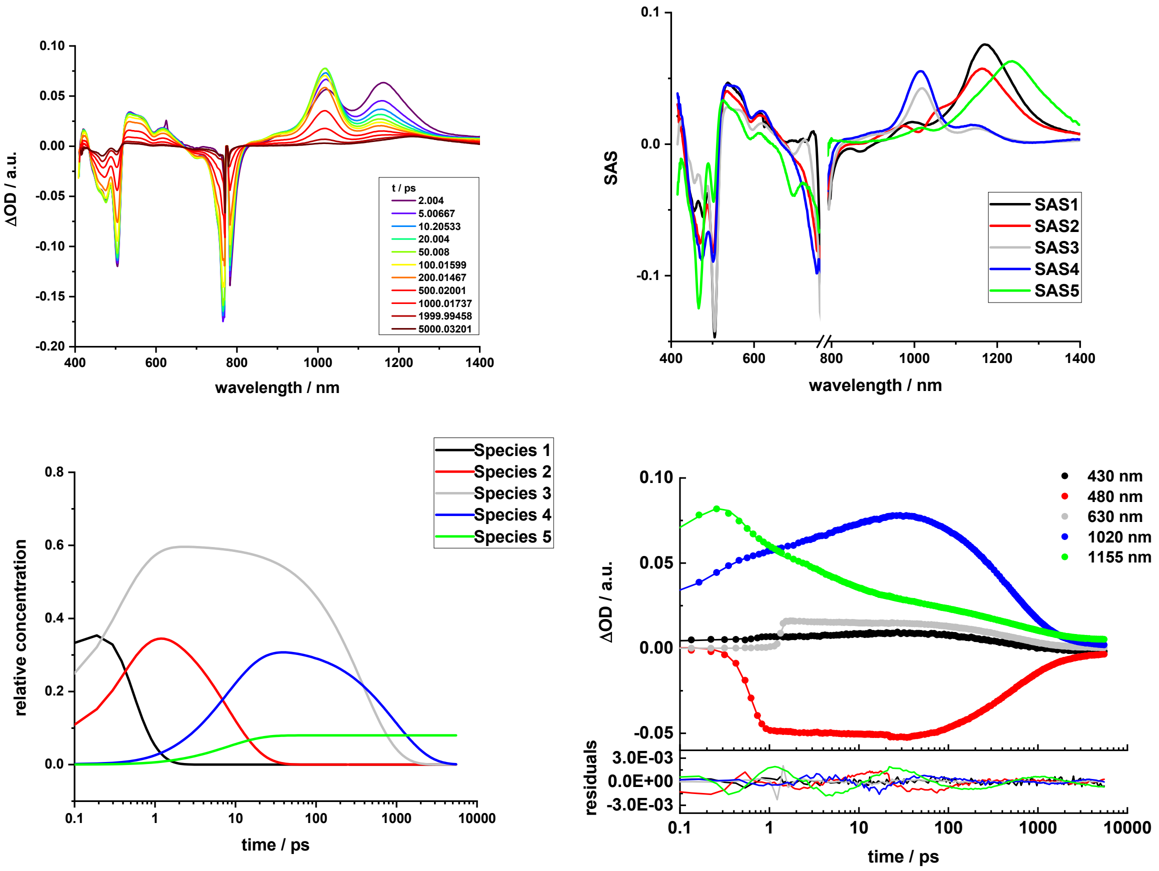

Figure S25: fsTAS analysis of **2-Zn** + **3** upon 775 nm excitation. Top left: Differential absorption changes (visible and near-infrared) obtained upon pump probe experiments; top right: species associated spectra derived from target analysis of the TA data; bottom left: relative population of the transient species derived from target analysis; bottom right: selected time traces with fits and fit residuals resulting from target analysis. ~150 fs time resolution, solvent chlorobenzene.

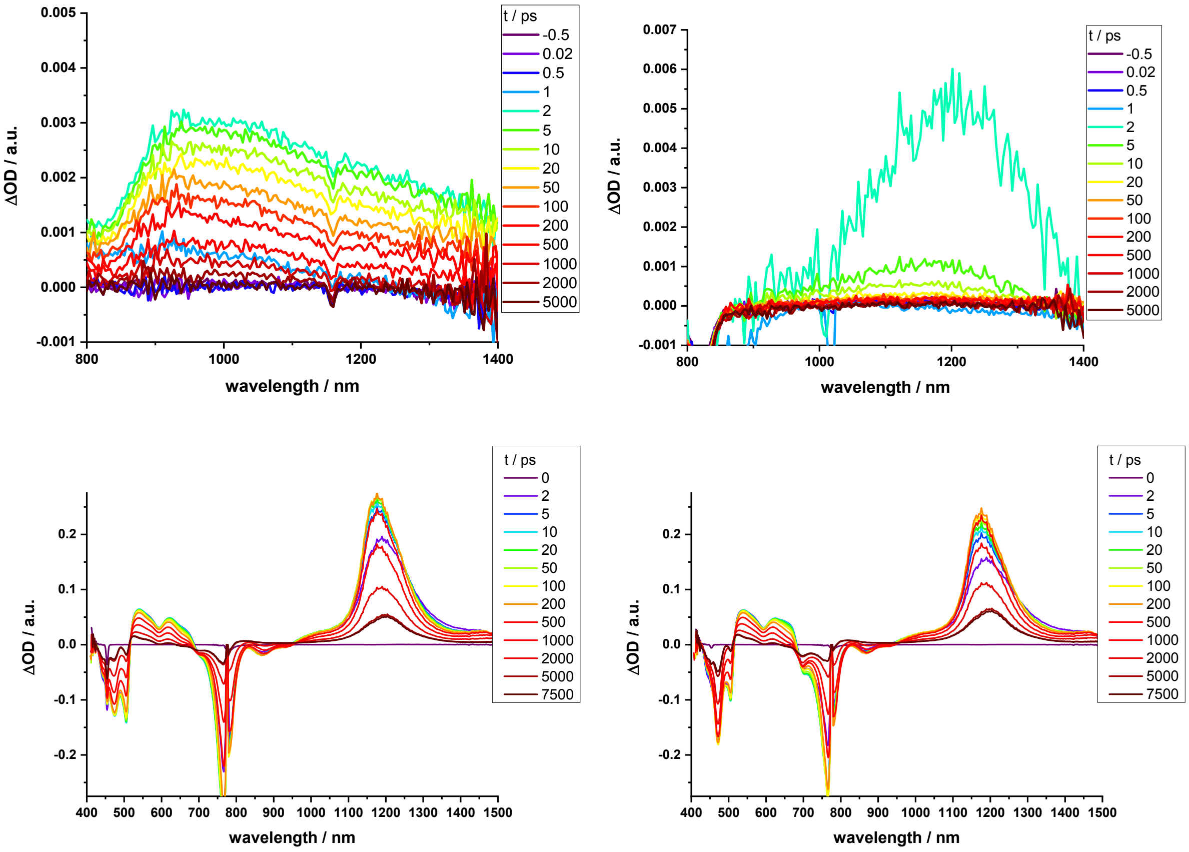

Figure S26: Reference fs-TAS measurements. Top left: excitation of **3** at 387 nm in the C_{60} UV absorption. Top right: excitation of **3** at 775 nm, where **3** does not exhibit absorption. Note: the observed extremely short-lived transient is a solvent response. Bottom left: excitation of **2Zn + 1,10DA** at 450 nm. Bottom right: excitation of **2Zn + 1,12DA** at 450 nm.

3. Molecular Modelling

All geometries used in this study are pre-optimized with a modified Dreiding forcefield. Simulated Annealing, including 2000 geometries, yields low energy conformations, that were further optimized with DFT using B3LYP/def2-SVP in gasphase. All potential energy scans are also performed on this level of theory. The resulting structures were finally optimized with B3LYP/def2-TZVP in gasphase. The same is done for certain steps along the potential energy scans. Finally, single point calculations, including solvation in toluene and THF, as well as empirical dispersion correction are used to verify the obtained gas phase results under conditions close to the experimental ones. Estimations for the binding strength of the amines with the ZnP are calculated with:

B3LYP/def2-SVP//B3LYP/def2-SVP

B3LYP/def2-TZVP//B3LYP/def2-SVP

B3LYP/def2-TZVP//B3LYP/def2-TZVP

Cam-B3LYP/def2-TZVP//B3LYP/def2-TZVP

B3LYP/def2-TZVP//B3LYP/def2-TZVP dispersion correction GD3

B3LYP/def2-TZVP//B3LYP/def2-TZVP dispersion correction GD3, IEFPCM solvation THF and toluene

B3LYP/def2-TZVP//B3LYP/def2-TZVP IEFPCM solvation THF and toluene

TD-DFT single point calculations including the 20 lowest singlet and 20 lowest triplet excited states were carried out with B3LYP/def2-TZVP//B3LYP/def2-TZVP in gasphase and solvation in toluene and THF. Furthermore, the range separated Cam-B3LYP is used with the same basis set.

For most calculations, the structures are reduced to the ZnP-center with neighboring substituents, excluding the two HBC moieties, however the full structures were also optimized and treated with the same routine as mentioned above.

We limited the used geometries to the porphyrins and HBCs by replacing the trihexylsilyl (THS) and tert-butyl substituents with terminating hydrogens. This was done, to reduce the degree of freedom in the system and, thus, to increase the comparability between the calculated dimers.

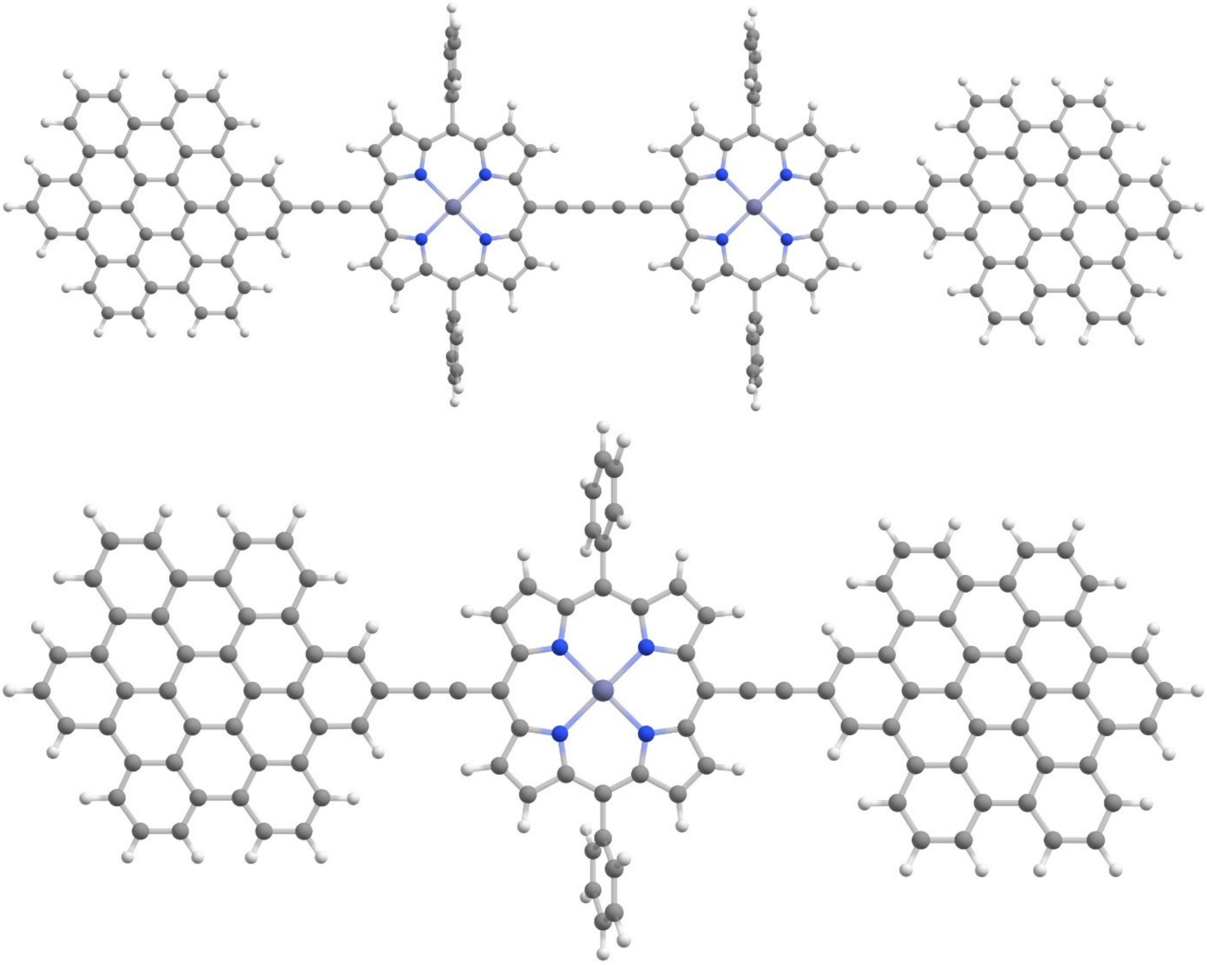

Figure S27: B3LYP/def2-TZVP optimized geometries of **2-Zn** (top) and **1-Zn** (bottom) in the gas phase.

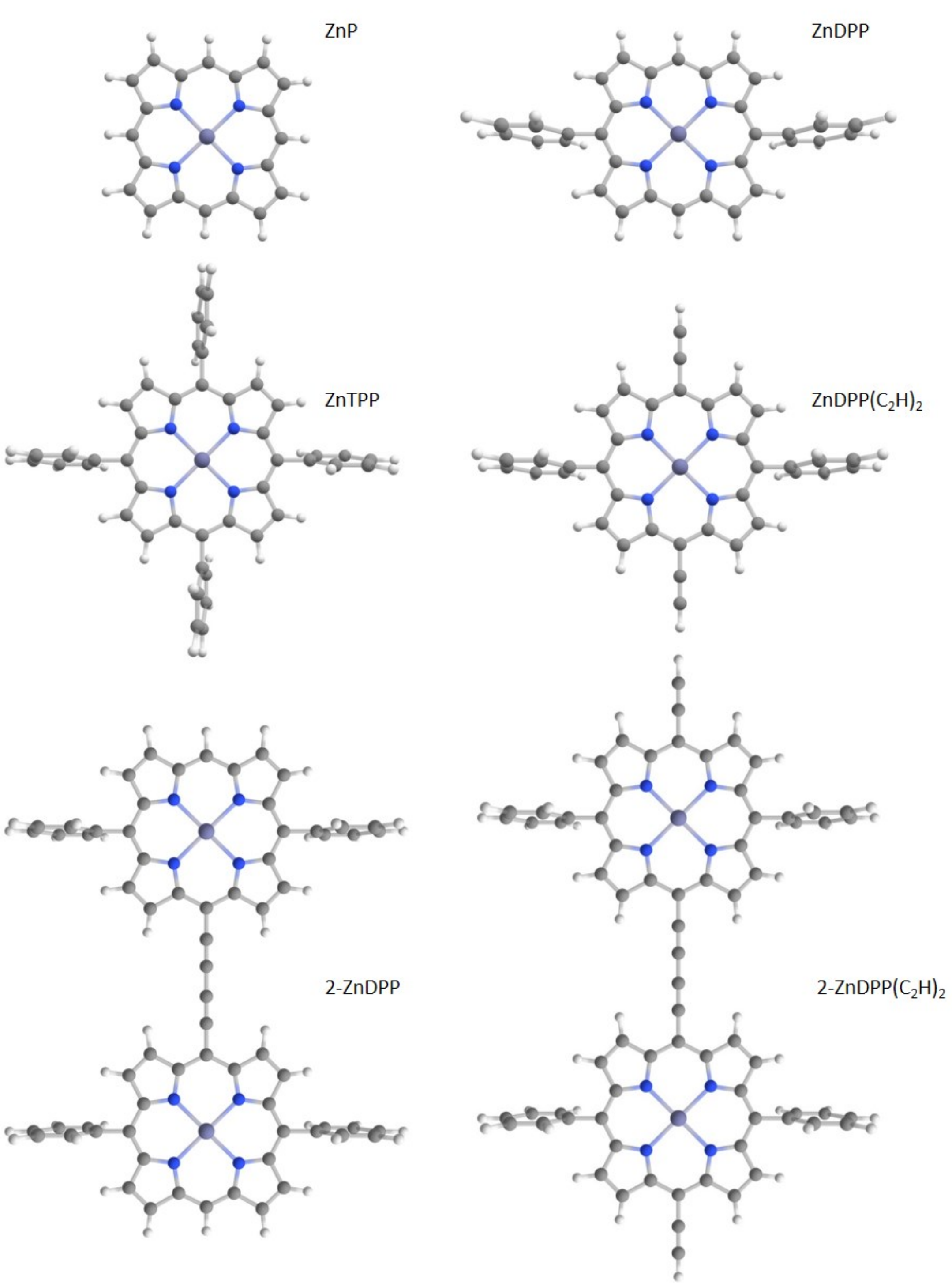

Figure S28: B3LYP/def2-TZVP optimized geometries of porphyrin monomers (top) and dimers (bottom) used for potential energy scans and TD-DFT calculations.

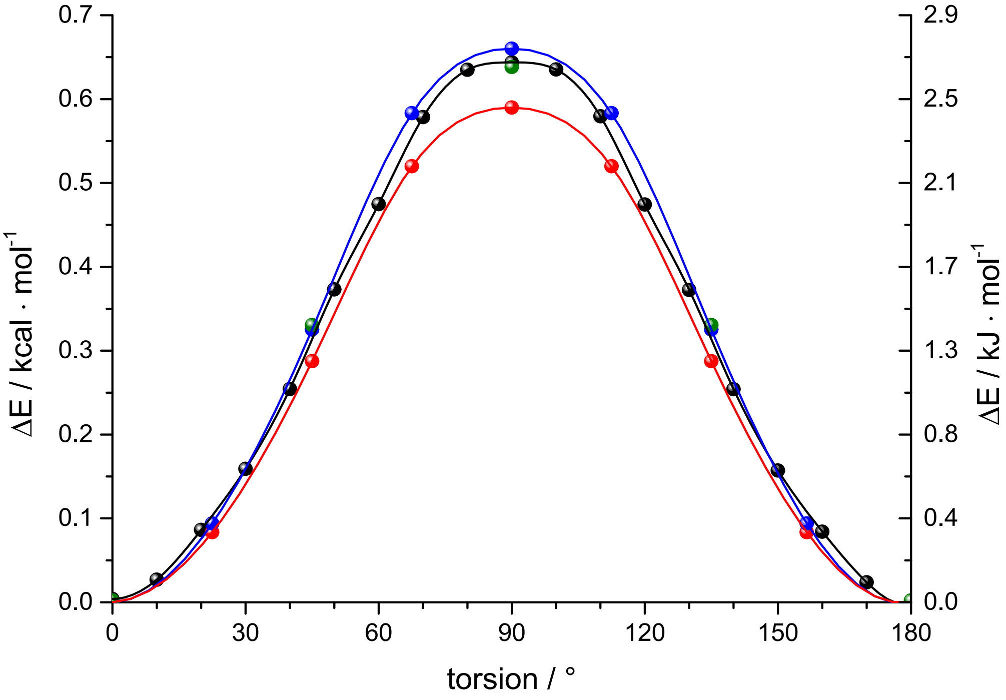

Figure S29: Relaxed potential energy scan of **2-ZnDPP**. (red – B3LYP/def2-SVP, black – B3LYP/def2-TZVP), **2-ZnDPP(C_2H)$_2$** (blue – B3LYP/def2-TZVP) and **2-Zn** (green – B3LYP/def2-TZVP) – lines are based on a cubic spline.

		$E_{complex}$ Ha	E_{amine} Ha	E_{ZnP} Ha	E_D kcal · mol^{-1}	Zn-N pm
MP2	TZVP	-2860.35116	-95.64342	-2764.67190	-22.47	215.79
CBS-APNO		-2860.91343	-95.71923	-2765.18462	-6.01	224.44
AM1* (kcal/mol)		179.50	-7.37	198.47	-11.60	237.22
B3LYP	SVP	-2862.74638	-95.78490	-2766.93413	-17.15	220.42
	TZVP	-2864.15823	-95.90122	-2768.23995	-10.70	224.42
	QZVP	-2864.27279	-95.91162	-2768.34548	-9.84	224.44
	QZVPP	-2864.27296	-95.91162	-2768.34574	-9.78	224.44
B3LYP GD3	SVP	-2862.80446	-2766.97650	-95.78619	-26.19	218.01
	TZVP	-2864.21611	-2768.28234	-95.90250	-19.61	221.20
	QZVP	-2864.33068	-2768.38787	-95.91290	-18.75	221.16
	QZVPP	-2864.33085	-2768.38813	-95.91290	-18.70	221.16
Cam-B3LYP	SVP	-2862.21861	-95.72840	-2766.45849	-19.88	217.23
	TZVP	-2863.63341	-95.84461	-2767.76780	-13.17	220.64
	QZVP	-2863.74871	-95.85507	-2767.87419	-12.19	220.66
	QZVPP	-2863.74889	-95.85507	-2767.87447	-12.13	220.66

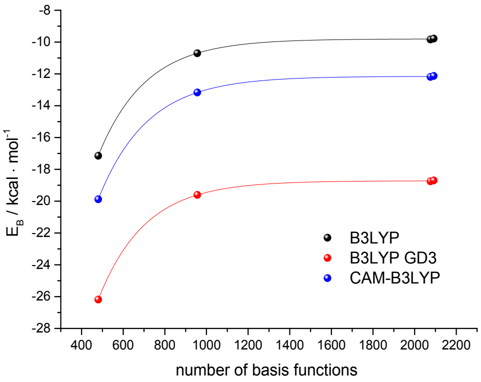

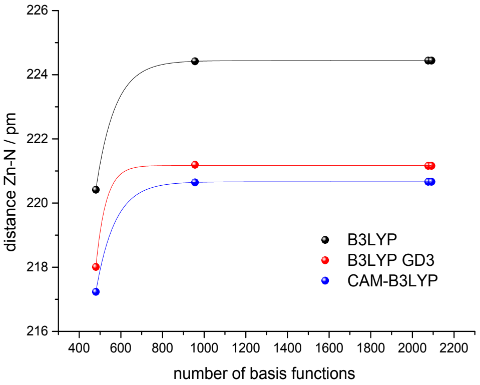

Figure S30 and Table S1: Benchmark of Zn-N interaction energy of a reference structure (B). B3LYP with and without empirical dispersion correction, Cam-B3LYP and wB97xd, all with def2-SVP, def2-TZVP, def2-QZVP and def2-QZVPP basis were compared to MP2, CBS-APNO and AM1* results.

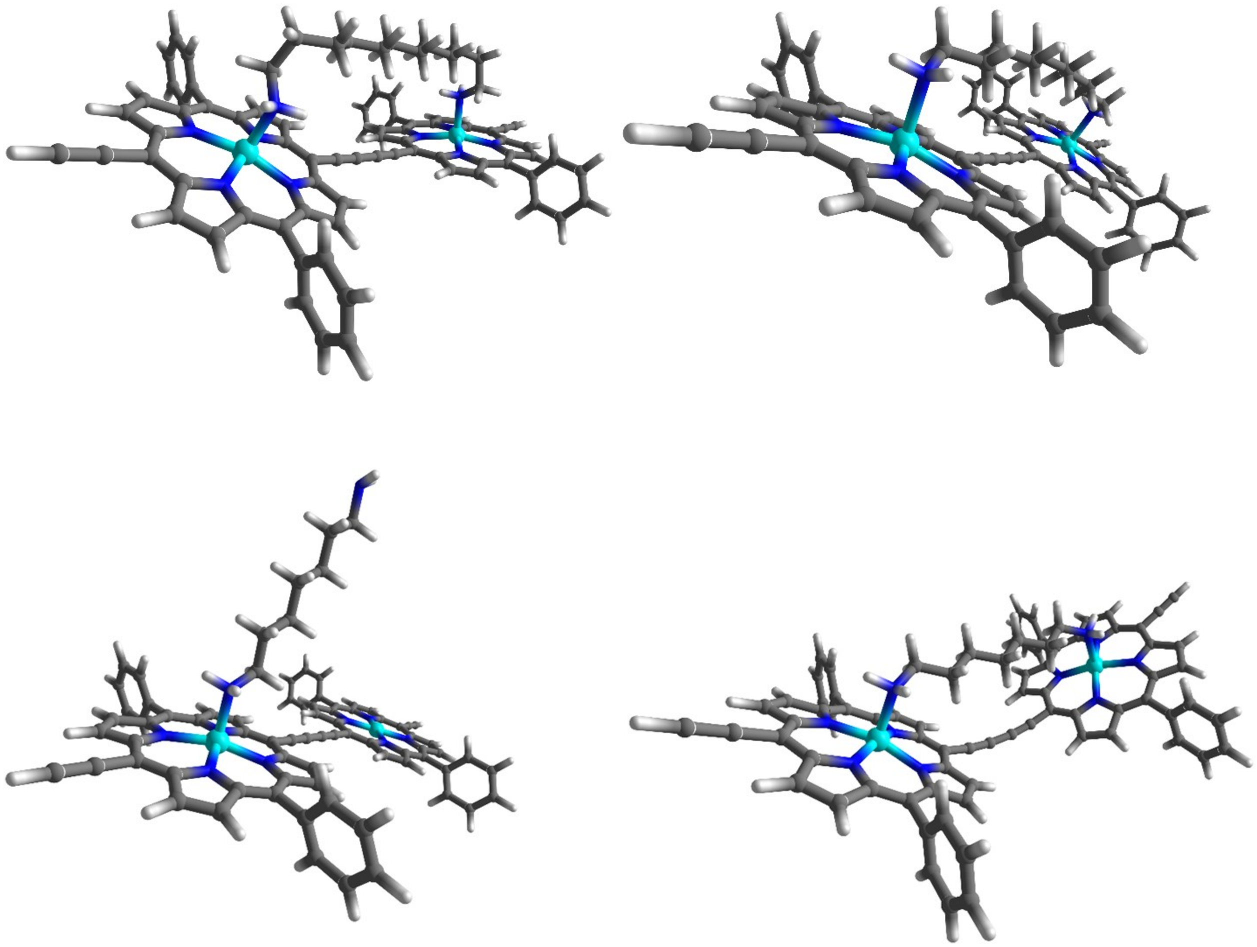

Figure S31: B3LYP/TZVP Optimized geometries of **2-ZnDPP(C_2H)$_2$** upon complexation with **1,12-DA** (top left), **1,10-DA** (top, right), **1,7-DA** (bottom left and right).

B3LYP/SVP GP<br>B3LYP/SVP GP	E_B / kcal mol^{-1}<br>adiabiabatic	E_B / kcal mol^{-1}<br>non adiabatic	E_D / kcal mol^{-1}<br>ZnP	E_D / kcal mol^{-1}<br>bridge	a / °	b / °
ZnDPP 1,12-DA	-32.1	-44.0	9.2	2.6	28	171
ZnDPP 1,10-DA	-31.9	-44.7	10.2	2.5	9	172
ZnDPP 1,7-DA (bi)	-26.2	-43.6	14.6	2.7	13	135
ZnDPP 1,7-DA (mono)	-17.4	-22.0	4.4	0.2	0	180
$ZnDPP_2$					0	180

B3LYP/TZVP GP<br>B3LYP/SVP GP	E_B / kcal mol^{-1}<br>adiabiabatic	E_B / kcal mol^{-1}<br>non adiabatic	E_D / kcal mol^{-1}<br>ZnP	E_D / kcal mol^{-1}<br>bridge	a / °	b / °
ZnDPP 1,12-DA	-18.3	-31.8	10.6	2.9	28	171
ZnDPP 1,10-DA	-17.9	-31.2	11.6	1.7	9	172
ZnDPP 1,7-DA (bi)	-13.6	-31.1	15.7	1.9	13	135
ZnDPP 1,7-DA (mono)	-10.9	-16.1	5.1	0.0	0	180
$ZnDPP_2$					0	180

B3LYP/TZVP GP<br>B3LYP/TZVP GP	E_B / kcal mol^{-1}<br>adiabiabatic	E_B / kcal mol^{-1}<br>non adiabatic	E_D / kcal mol^{-1}<br>ZnP	E_D / kcal mol^{-1}<br>bridge	a / °	b / °
ZnDPP 1,12-DA	-18.4	-30.9	9.4	3.2	21	170
ZnDPP 1,10-DA	-18.0	-30.4	10.0	2.3	8	172
ZnDPP 1,7-DA (bi)	-14.6	-30.4	13.9	1.8	0	134
ZnDPP 1,7-DA (mono)	-10.9	-15.6	4.6	0.2	3	180
$ZnDPP_2$					0	180

B3LYP/TZVP GP D3<br>B3LYP/TZVP GP D3	E_B / kcal mol^{-1}<br>adiabiabatic	E_B / kcal mol^{-1}<br>non adiabatic	E_D / kcal mol^{-1}<br>ZnP	E_D / kcal mol^{-1}<br>bridge	a / °	b / °
ZnDPP 1,12-DA	-47.1	-58.1	8.3	2.7	72	174
ZnDPP 1,10-DA	-48.9	-60.0	8.9	2.2	0	176
ZnDPP 1,7-DA (bi)	-41.3	-56.2	12.5	2.4	0	135
ZnDPP 1,7-DA (mono)	-21.5	-25.4	3.8	0.2	3	178
$ZnDPP_2$					0	180

B3LYP/TZVP THF<br>B3LYP/TZVP THF	E_B / kcal mol^{-1}<br>adiabiabatic	E_B / kcal mol^{-1}<br>non adiabatic	E_D / kcal mol^{-1}<br>ZnP	E_D / kcal mol^{-1}<br>bridge	a / °	b / °
ZnDPP 1,12-DA	-11.0	-22.2	8.0	3.2	19	169
ZnDPP 1,10-DA	-10.2	-21.4	8.7	2.4	7	172
ZnDPP 1,7-DA (bi)	-7.3	-21.7	12.8	1.6	0	134
ZnDPP 1,7-DA (mono)	-7.3	-11.6	4.1	0.1		
$ZnDPP_2$					2	171

B3LYP/TZVP THF D3<br>B3LYP/TZVP THF D3	E_B / kcal mol^{-1}<br>adiabiabatic	E_B / kcal mol^{-1}<br>non adiabatic	E_D / kcal mol^{-1}<br>ZnP	E_D / kcal mol^{-1}<br>bridge	a / °	b / °
ZnDPP 1,12-DA	-39.0	-48.6	6.8	2.8	69	173
ZnDPP 1,10-DA	-40.8	-50.7	7.6	2.3	3	176
ZnDPP 1,7-DA (bi)	-33.6	-47.4	11.5	2.3	0	135
ZnDPP 1,7-DA (mono)	-17.8	-21.2	3.3	0.1		
$ZnDPP_2$					2	171

Table S2: Binding energy, torsion angle between porphyrin planes (a) and the bend angle along the bridge (b) for complex formed of 2-ZnDPP with 1,12 – 1,10 and 1,7 – DA.

enviroment	GP	GP	GP	GP	GP	THF opt	THF opt	THF	THF	THF	THF	toluene	toluene	toluene	toluene
Method	B3LYP	B3LYP	B3LYP	B3LYP	B3LYP GD3	B3LYP	B3LYP GD3	B3LYP	B3LYP GD3	CAM-B3LYP	wB97xd	B3LYP	B3LYP D3	CAM-B3LYP	wB97xd
Basis set	SVP//SVP	TZVP//SVP	TZVP//TZVP	QZVPP//TZVP	TZVP//TZVP	TZVP//TZVP	TZVP//TZVP	TZVP//TZVP	TZVP//TZVP	TZVP//TZVP	TZVP//TZVP	TZVP//TZVP	TZVP//TZVP	TZVP//TZVP	TZVP//TZVP
1,12 DA	-32.1	-18.3	-18.4	-16.6	-47.1	-11.0	-39.0	-11.1	-34.0	-33.0	-33.4	-14.6	-35.4	-36.5	-37.0
1,10 DA	-31.9	-17.9	-18.0	-16.1	-48.9	-10.2	-40.8	-10.3	-36.6	-34.9	-35.9	-13.9	-38.2	-38.6	-39.6
1,7 DA bidentate	-41.3	-13.6	-14.6	-13.1	-41.3	-7.3	-33.6	-7.4	-31.2	-29.4	-30.2	-10.9	-32.8	-33.0	-33.8
1.7 DA monodentate	-17.4	-10.9	-10.9	-10.1	-21.5	-7.3	-17.8	-7.4	-17.1	-16.7	-16.7	-9.1	-16.9	-18.4	-18.4

Table S3: Binding energy derived by different levels of theory.

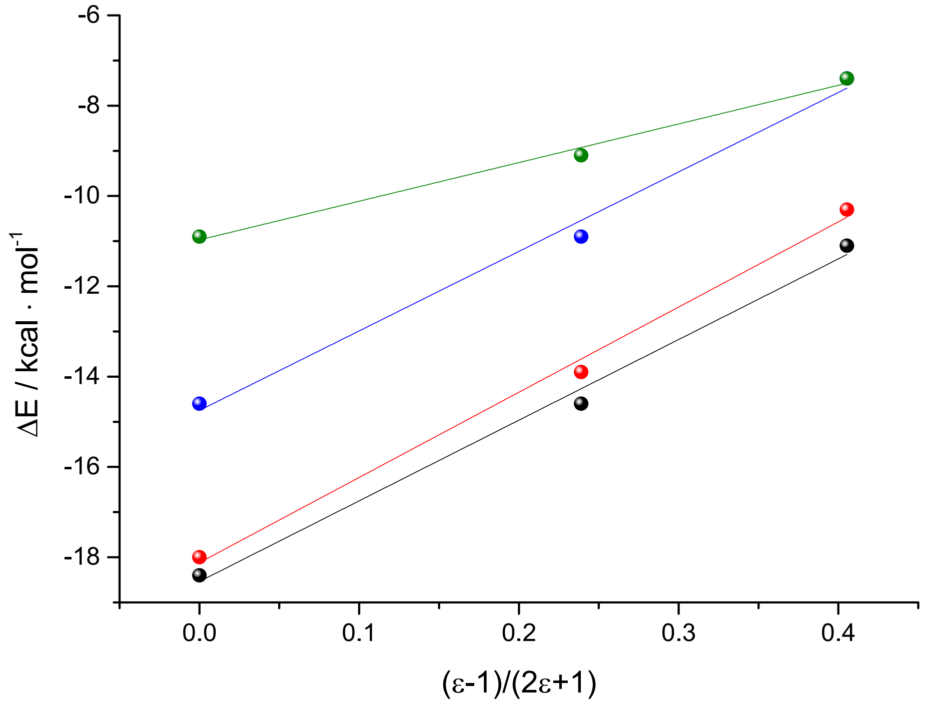

Figure S32: Solvent dependence of the binding energy for complexation of **ZnDPP** with **1,12 –DA** (black), **1,10 – DA** (red), **1,7 –DA** (blue=bidentate, green=monodentate).

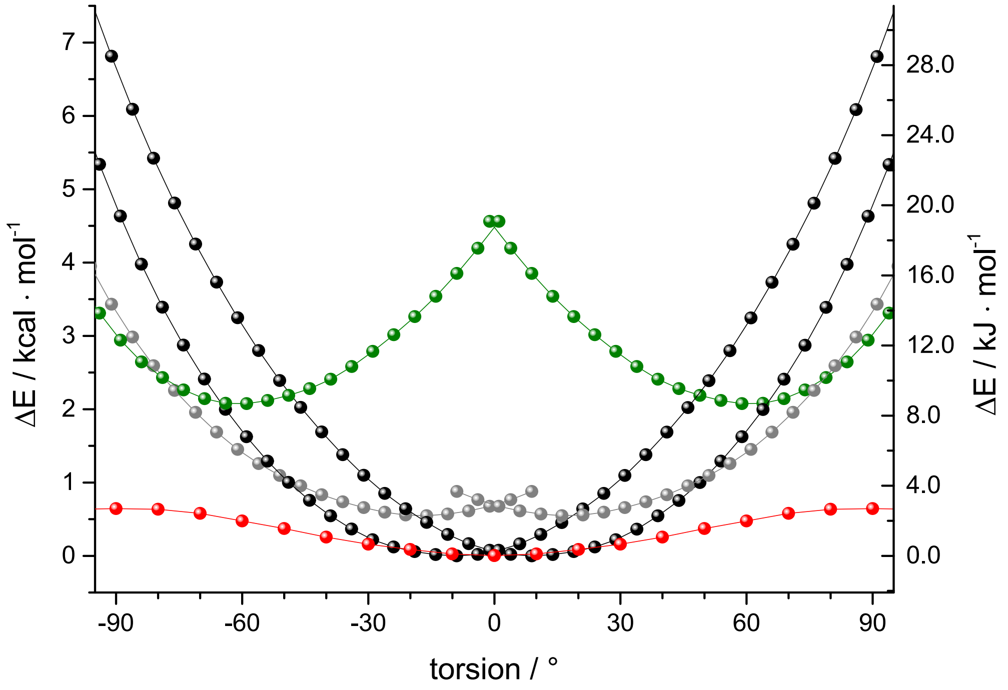

Figure S33: Relaxed potential energy scan of 2-ZnPDPP (red – B3LYP/def2-SVP) and **2-ZnDPP**, complexed with **1,10 DA** with different conformations along the bridge (black, grey, green – all B3LYP/def2-SVP).

			w (@ 1kcal mol^{-1}) °	w (@ 4 kcal mol^{-1}) °	EA (@ 90°) kcal · mol^{-1}
2-ZnPDPP	1,12 DA	non adiabatic	92	179	2.0
		adiabatic	148	220	2.0
2-ZnPDPP	1,10 DA	non adiabatic	77	153	4.8
		adiabatic	96	198	3.0

		center °	center °
2-ZnPDPP	1,12 DA	-28	28
2-ZnPDPP	1,10 DA	-8	8

Table S4: Width and activation barrier upon Zn-Zn torsion for **2-Zn** + **1,12 –DA** and **1,10 –DA**.

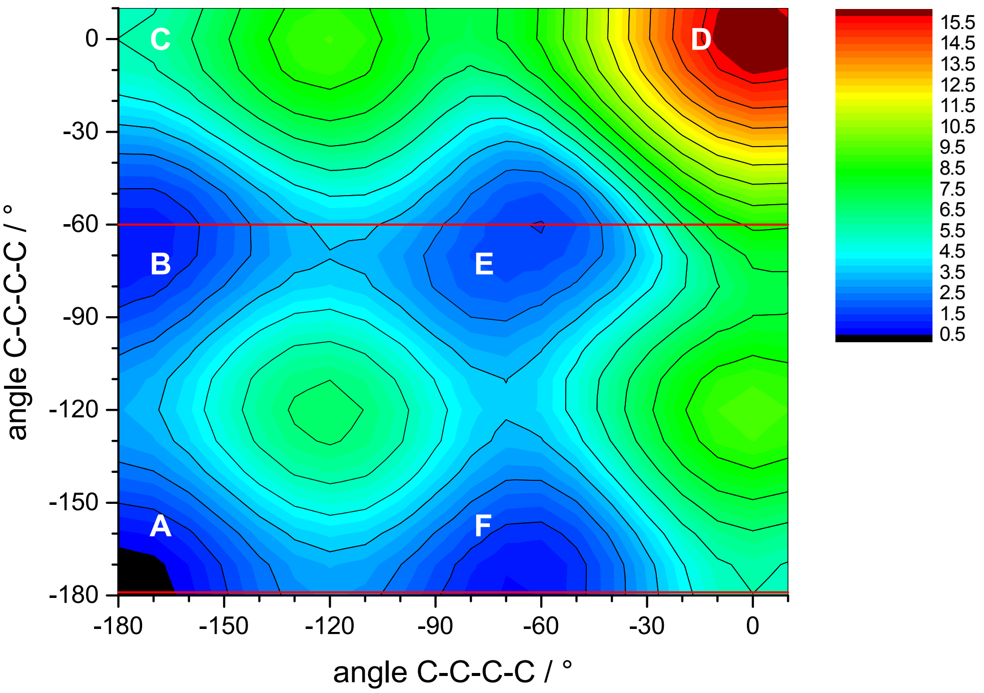

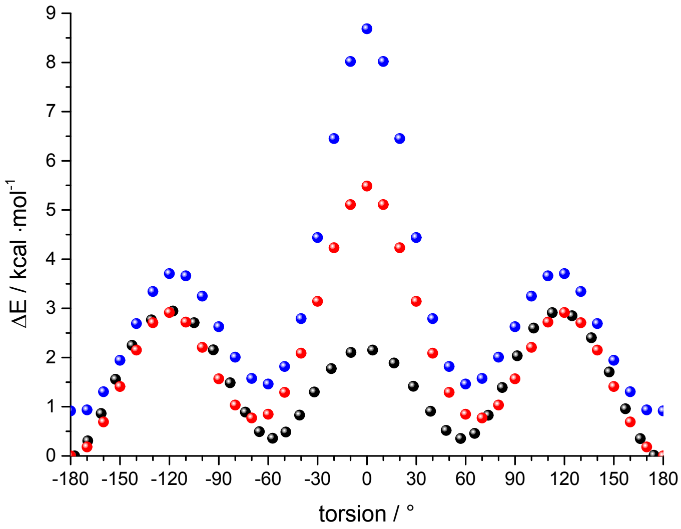

Figure S34: top: Relaxed potential energy scan of **1,10 DA.** Two neighboring C–C bonds were rotated, yielding the following conformation: anti-anti (A), anti-gauche (B), anti-eclipsed (C), eclipsed-eclipsed (D), gauche-gauche (E) and gauche-anti (F). Energies are given in kcal·mol^{-1}. Bottom: Relaxed potential torsion scan around C–NH_2 bond (black) and around C–C bond with neighboring C–C bond in anti-conformation (red) or gauche conformation (blue).

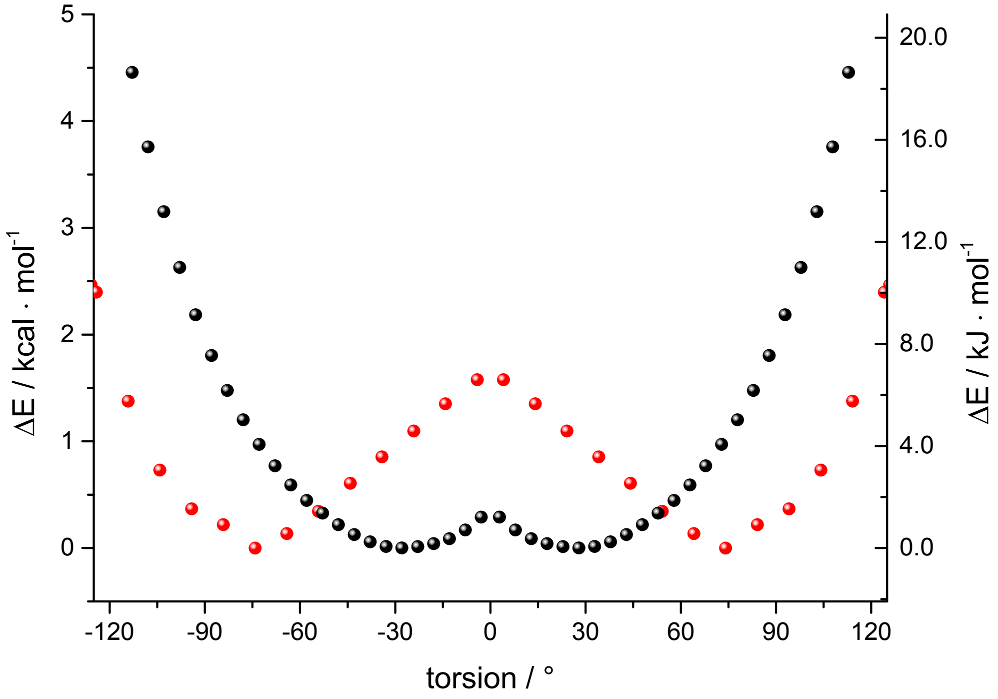

Figure S35: Relaxed potential energy scan of **2-ZnPDPP** complexated with **1,12 DA** with (red) and without dispersion correction (black) calculated with B3LYP/def2-SVP.

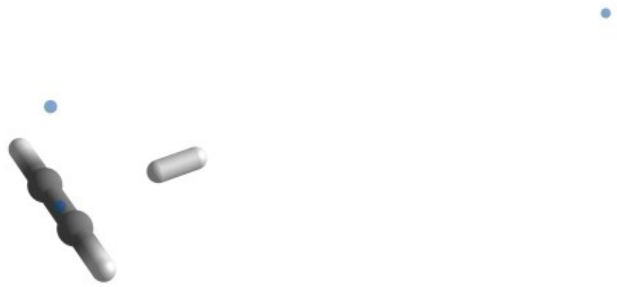

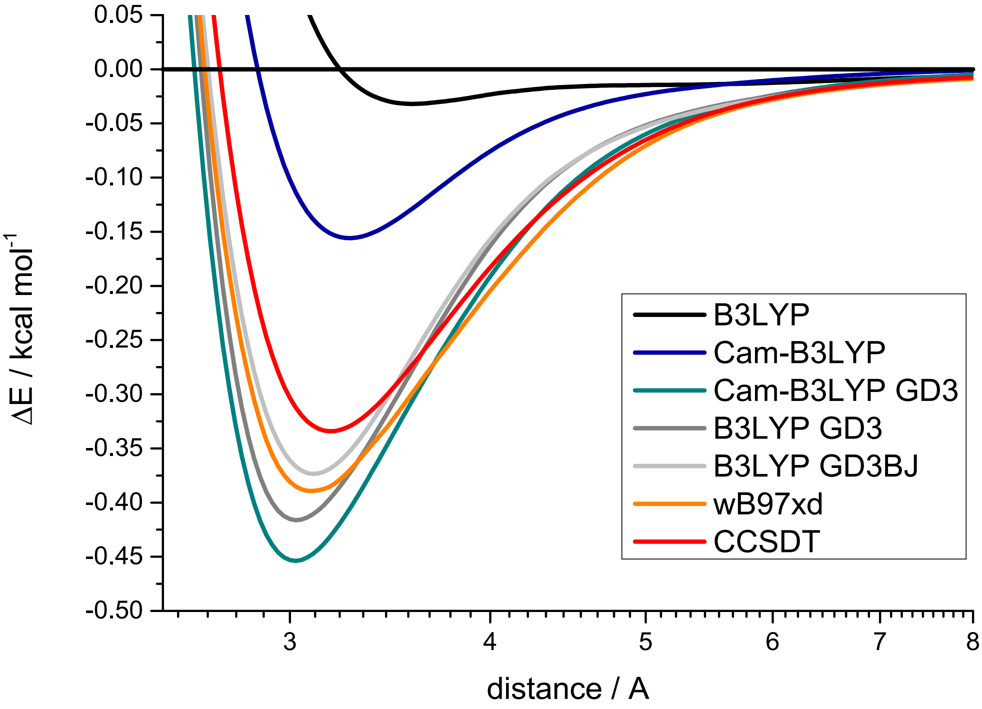

		B3LYP def2-QZVPP	Cam-B3LYP def2-QZVPP	Cam-B3LYP GD3 def2-QZVPP	B3LYP GD3 def2-QZVPP	B3LYP GD3BJ def2-QZVPP	wB97xd def2-QZVPP	CCSD(T) def2-QZVPP
ΔE_{DISP}	kcal mol^{-1}	-0.032	-0.156	-0.454	-0.416	-0.373	-0.389	-0.334
$\Delta E_{DISP} - \Delta E_{DISP}$ (CCSD(T))	kcal mol^{-1}	0.302	0.178	-0.120	-0.082	-0.039	-0.055	0.000
R	pm	357.5	327.5	302.5	302.5	310	307.5	317.5
ΔR = R - R (CCSD(T))	pm	40	10	-15	-15	-7.5	-10	0

Figure S36 and Table S5: Benchmark of Dispersion interaction.

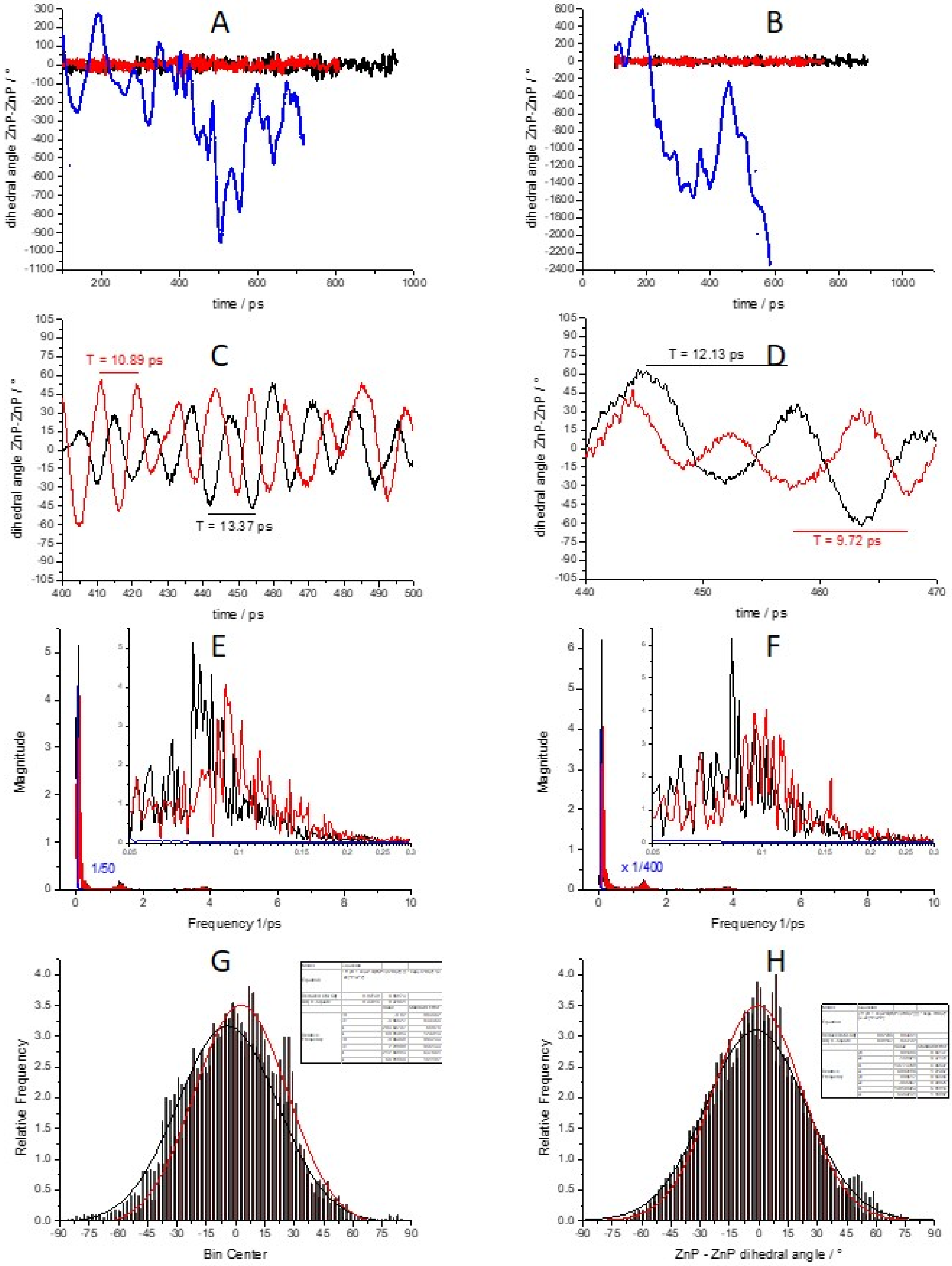

Figure S37: Zn-Zn torsion evolution of **1,12 DA** bridged **2-Zn** (black), **1,10-DA** bridged **2-Zn** (red) and **2-Zn** (blue) during AM1 molecular dynamic simulation at 150 K (A and C) and 300 K (B and D). The horizontal bars in graph C and D are scaled for the oscillation period given by the reciprocal of the main frequency as determined by Fourier Transformation (E and F). Graph G and H show the statistical analysis of the Zn-Zn torsion angles.

T	ν(1,12-DA)	ν(1,10-DA)	T (1,12-DA)	T (1,10-DA)	$\frac{k(1,12\text{-}DA)}{k(1,10\text{-}DA)}$	$\frac{k(1,10\text{-}DA)}{k(1,12\text{-}DA)}$
K	ps^{-1}	ps^{-1}	ps	ps		
150	0.07482	0.0918	13.37	10.89	0.66	1.51
300	0.08241	0.10285	12.13	9.72	0.64	1.56

Table S6: Oscillation frequency derived by Fourier transformation and the resulting oscillation period T. Last column shows the ratio of oscillation constant, assuming a harmonic oscillator were the mass is equal for both cases.

TD-DFT B3LYP/TZVP in GP

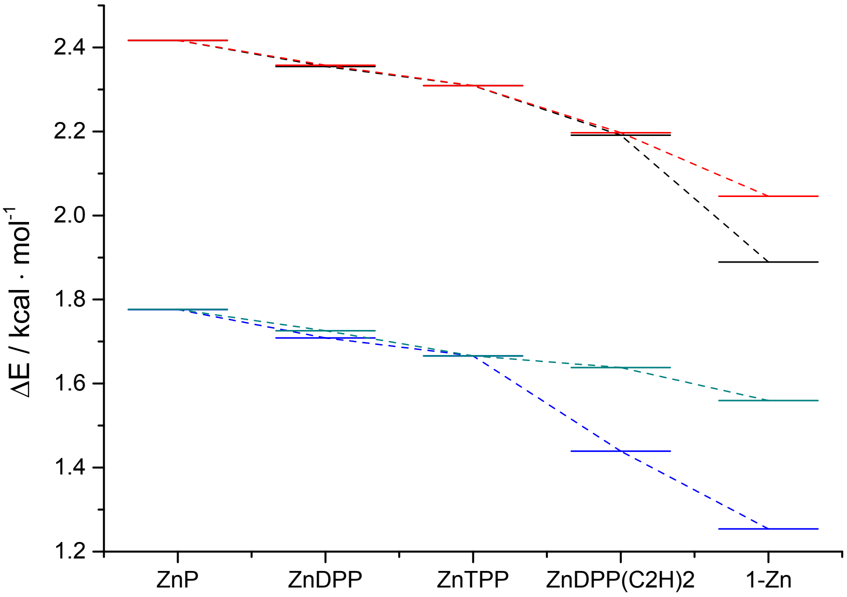

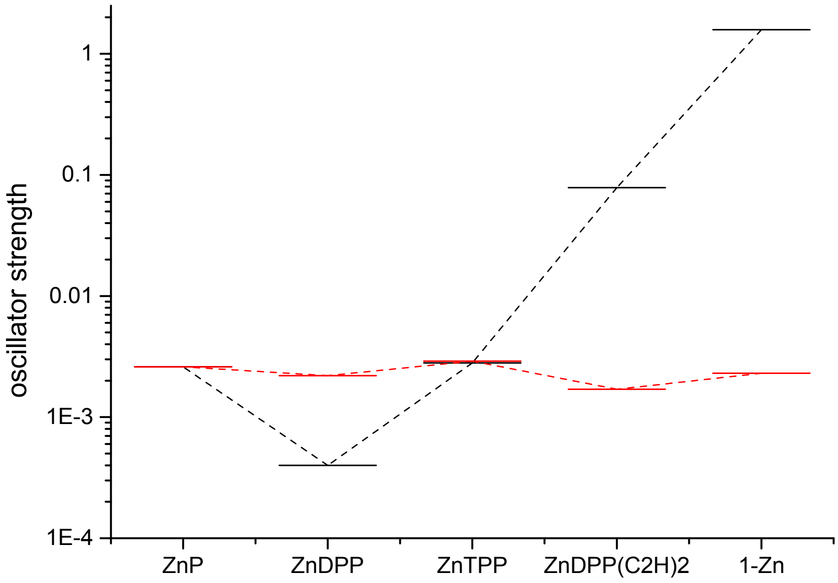

Figure S38: B3LYP/def2-TZVP calculated vertical excitation energies and oscillator strengths of monomers. Top: S_0 to S_1 / S_2 Q-band transition (black,red) and first two excited triplet states T_1 and T_2 (blue,cyan). Bottom: Oscillator strengths for S_0 to S_1 and S_2 transition (black, red).

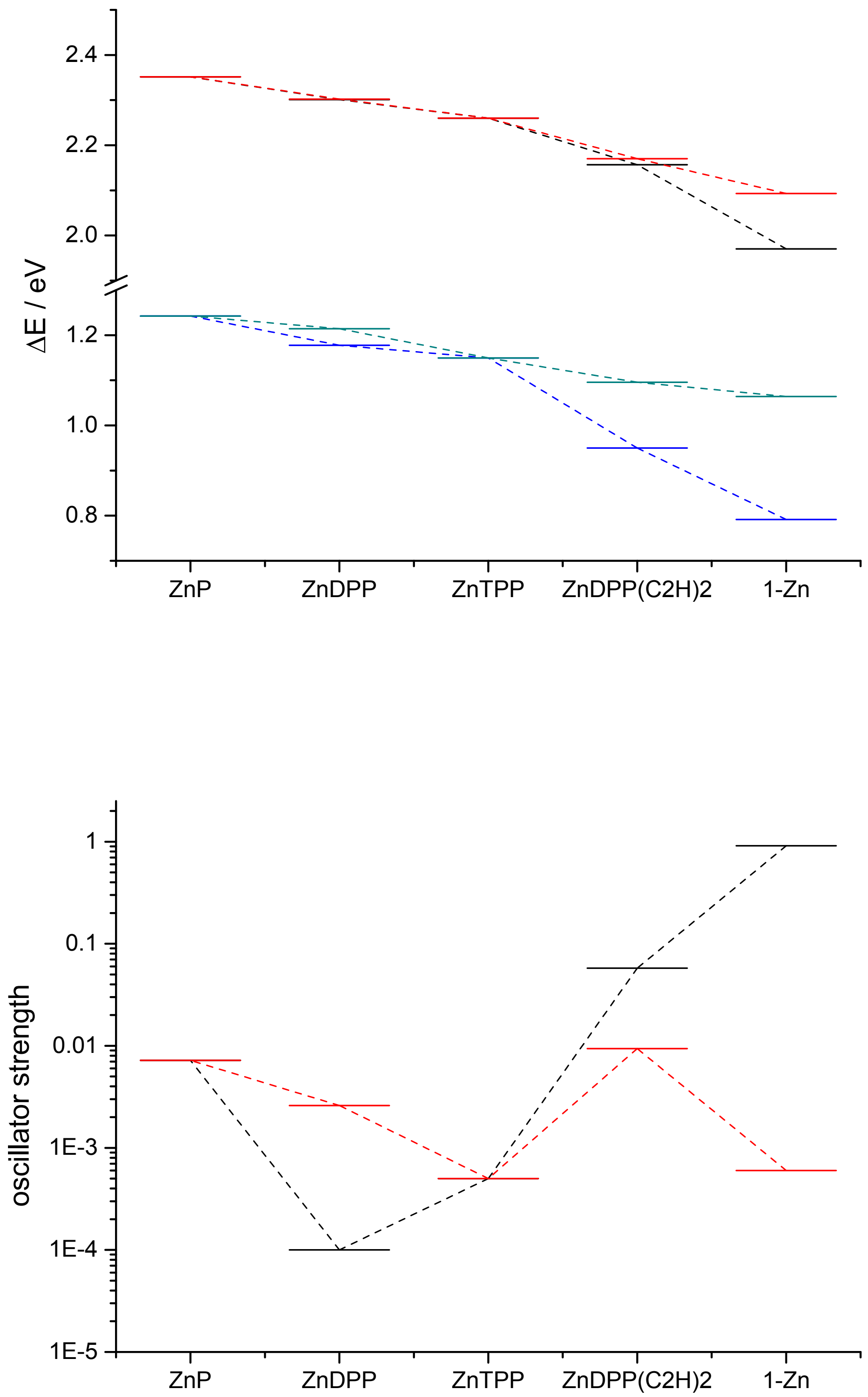

Figure S39: CAM-B3LYP/def2-TZVP calculated vertical excitation energies and oscillator strengths of monomers. Top: S_0 to S_1 / S_2 Q-band transition (black,red) and first two excited triplet states T_1 and T_2 (blue,cyan). Bottom: Oscillator strengths for S_0 to S_1 and S_2 transition (black, red).

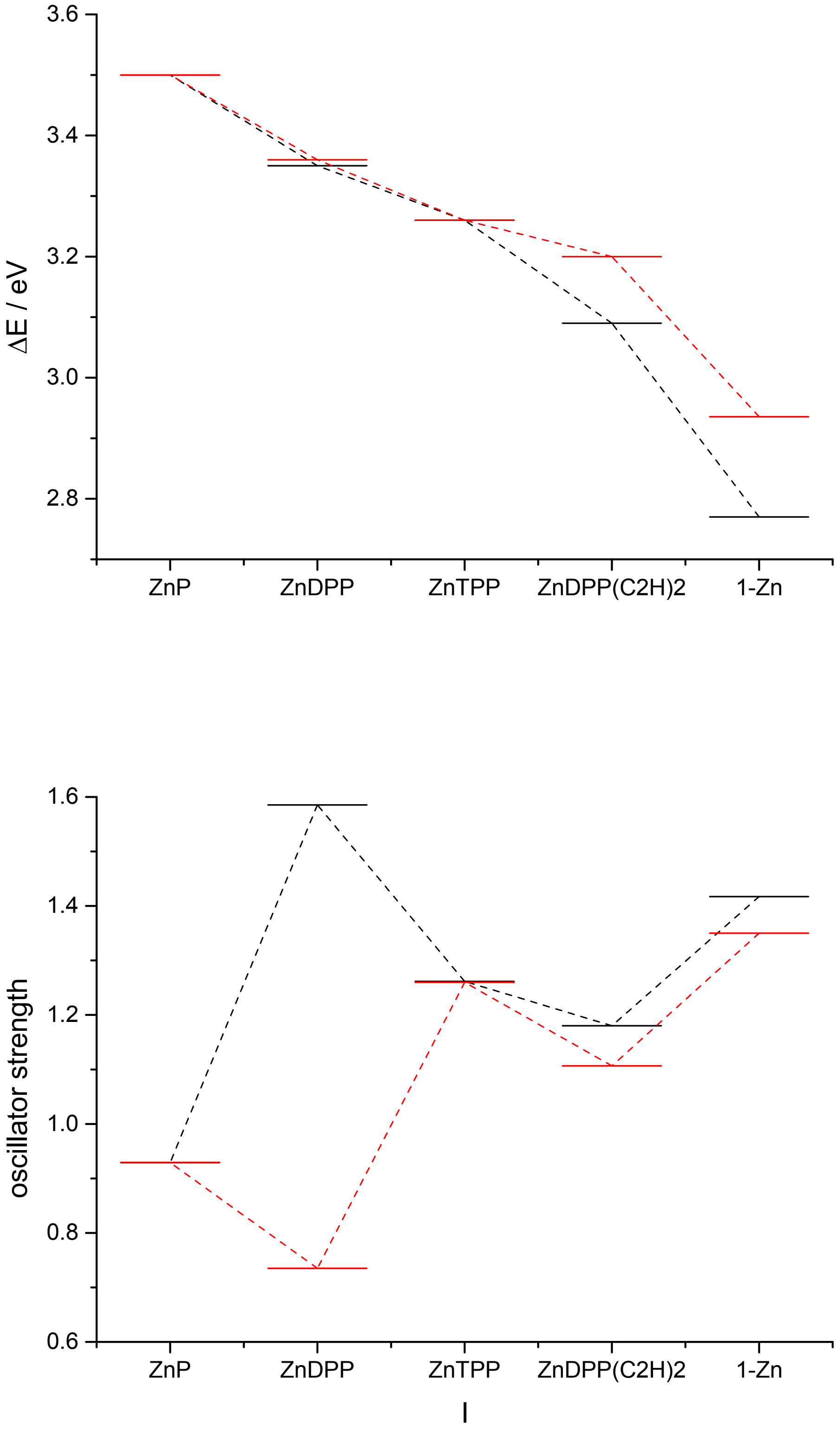

Figure S40: B3LYP calculated vertical excitation energies and oscillator strengths of monomers. Top: Soret-band transitions (black,red). Bottom: Oscillator strengths for Soret band transition (black, red).

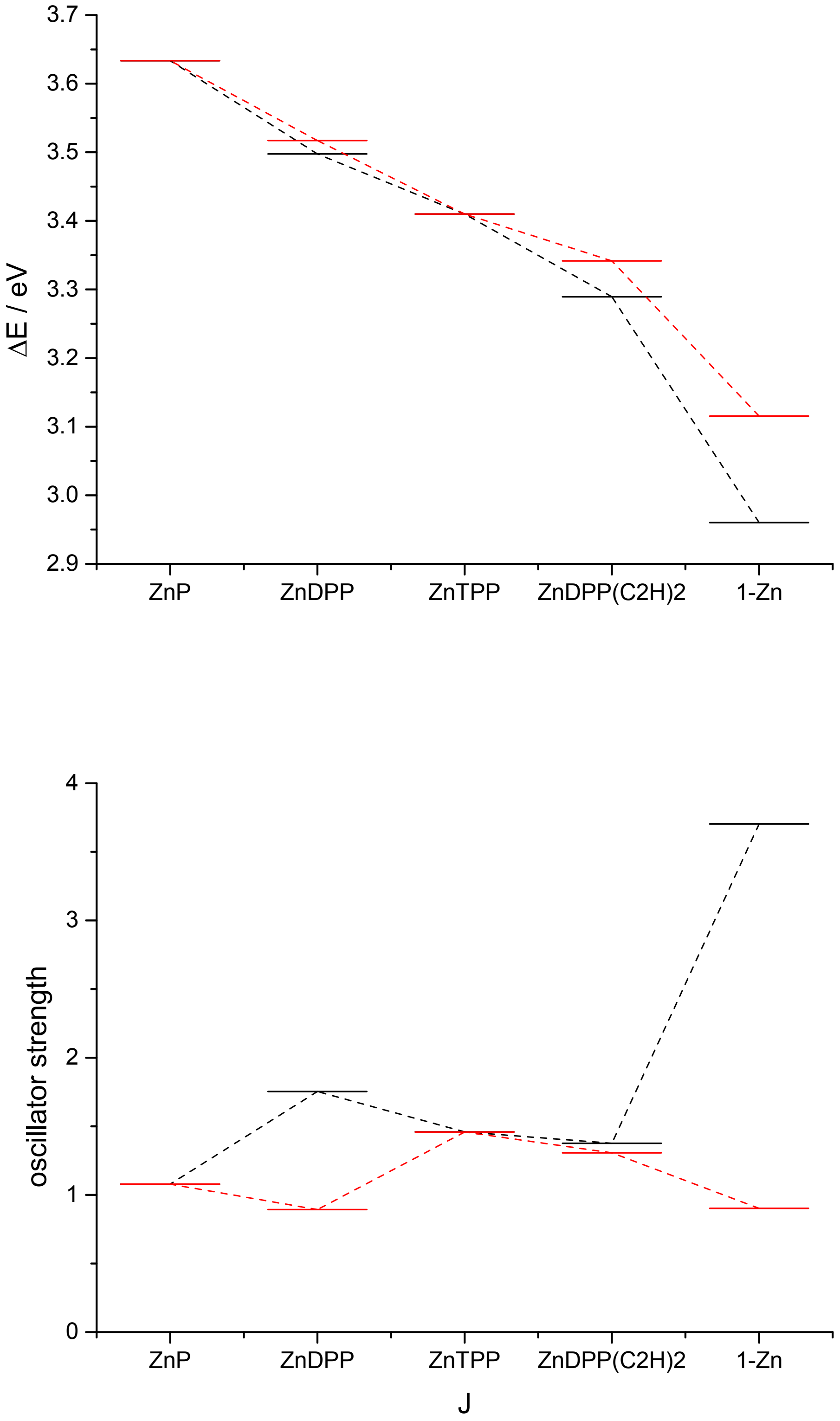

Figure S41: CAM-B3LYP calculated vertical excitation energies and oscillator strengths of monomers. Top: Soret-band transition (black,red). Bottom: Oscillator strengths for Soret band transition (black, red).

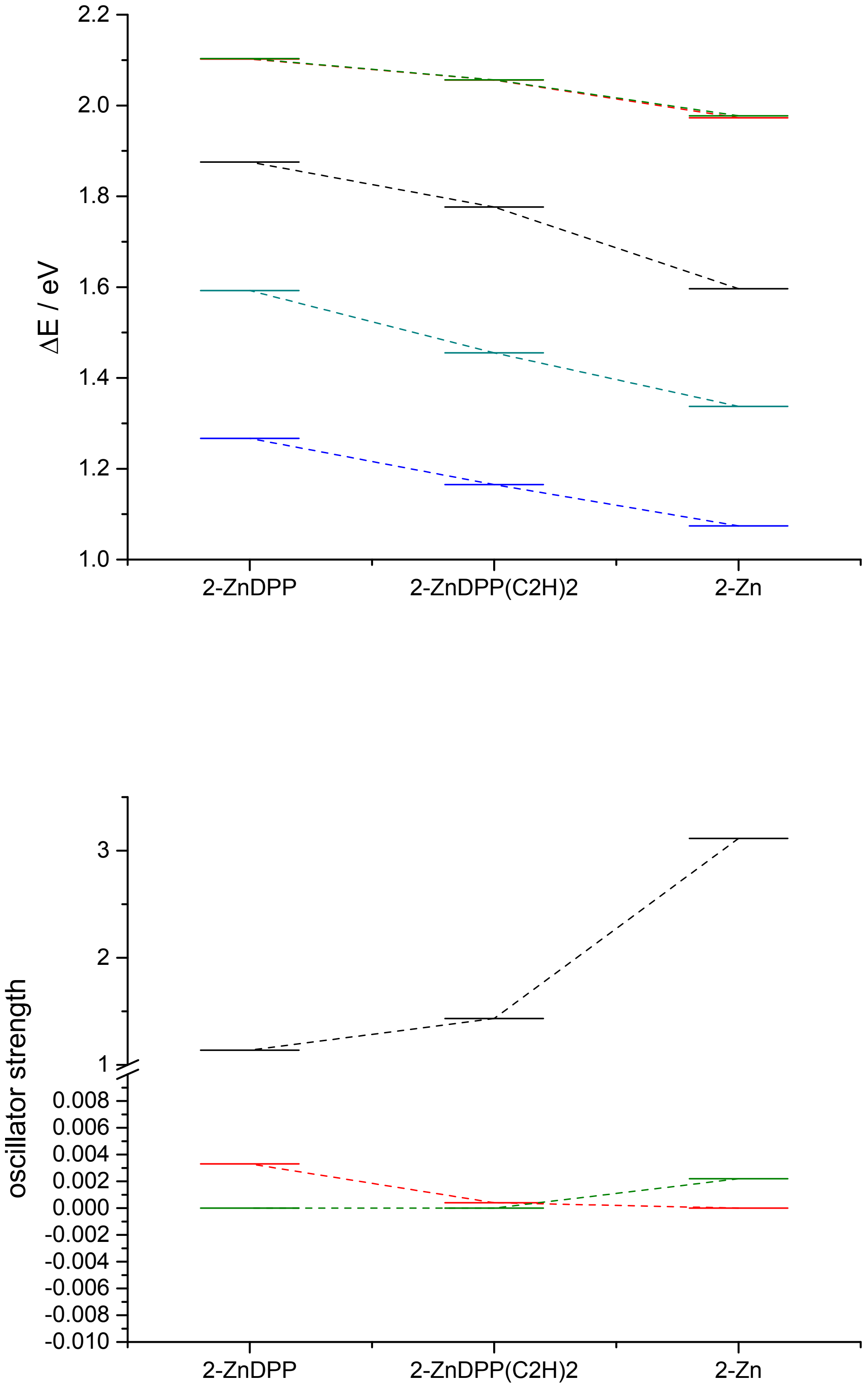

Figure S42: B3LYP calculated vertical excitation energies and oscillator strengths of dimers. Top: S_0 to S_1 / S_2 / S_3 transition (black,red,green) and S_0 to T_1 and T_2 (blue,cyan). Bottom: Oscillator strengths for S_0 to S_1 / S_2 / S_3 transition (black, red, green).

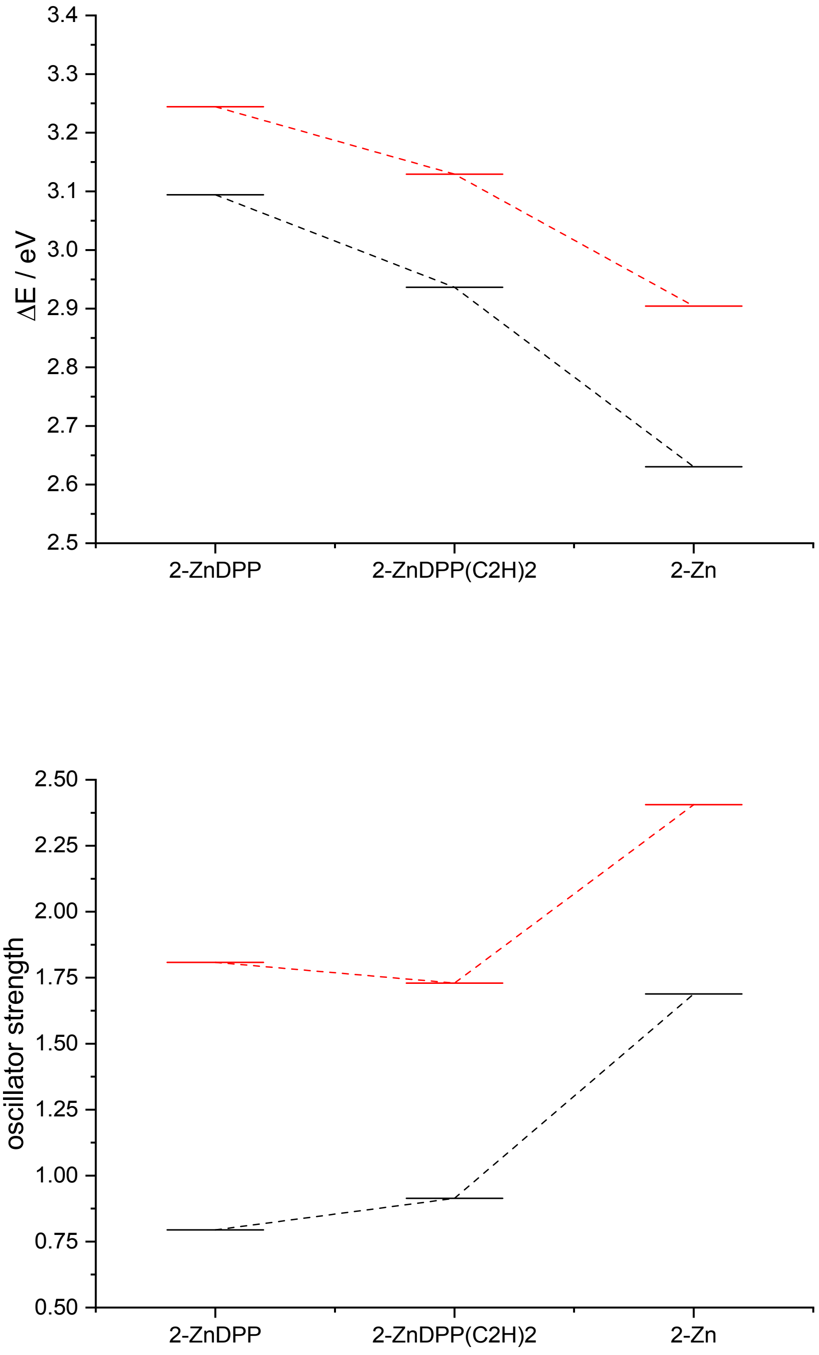

Figure S43: B3LYP calculated vertical excitation energies and oscillator strengths of dimers. Top: Soret-band transition (black, red). Bottom: Oscillator strengths for Soret-band transition (black, red).

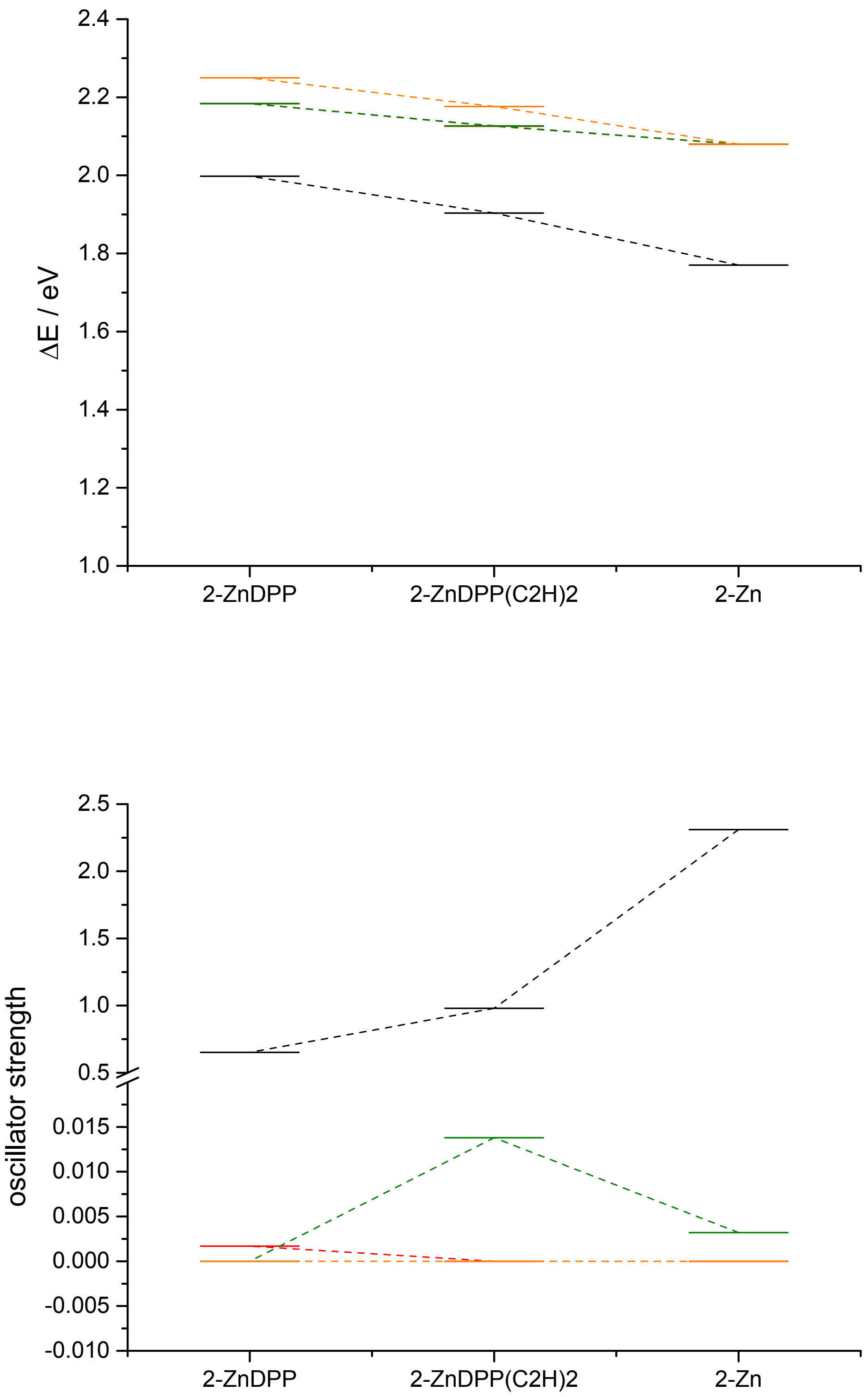

Figure S44: CAM-B3LYP calculated vertical excitation energies and oscillator strengths of dimers. Top: S_0 to S_1 / S_2 / S_3 / S_4 transition (black, red, green, yellow) and S_0 to T_1 and T_2 (blue, cyan). Bottom: Oscillator strengths for S_0 to S_1 / S_2 / S_3 / S_4 transition (black, red, green, yellow).

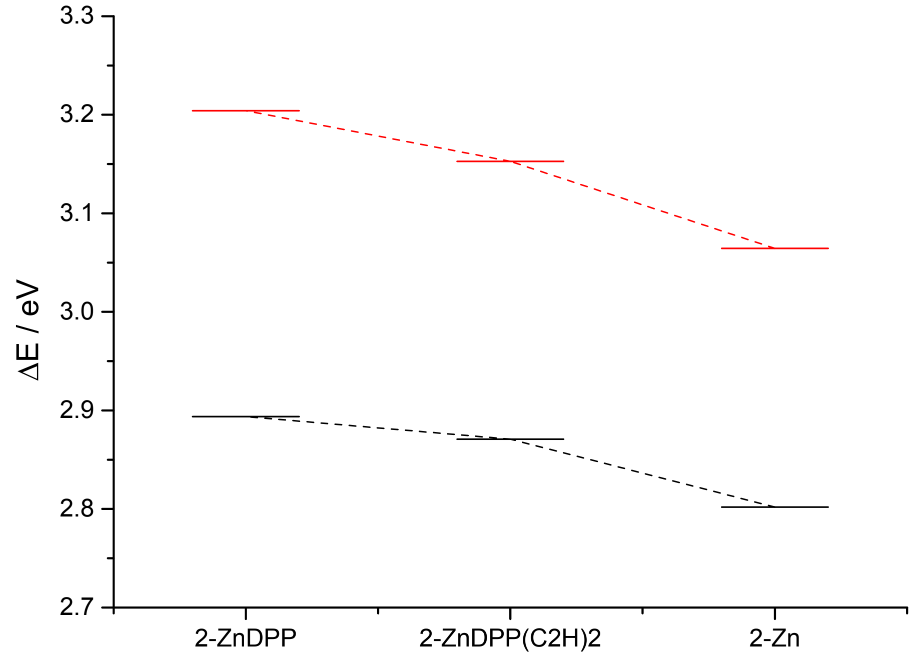

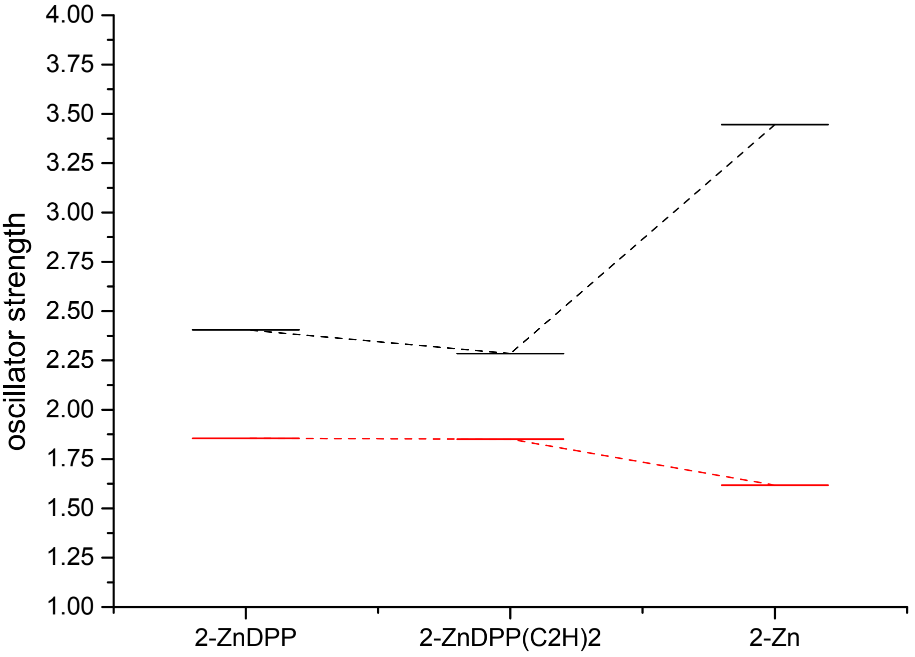

Figure S45: CAM-B3LYP calculated vertical excitation energies and oscillator strengths of dimers. Top: for Soret-band transition (black, red). Bottom: Oscillator strengths for Soret-band transition (black, red).

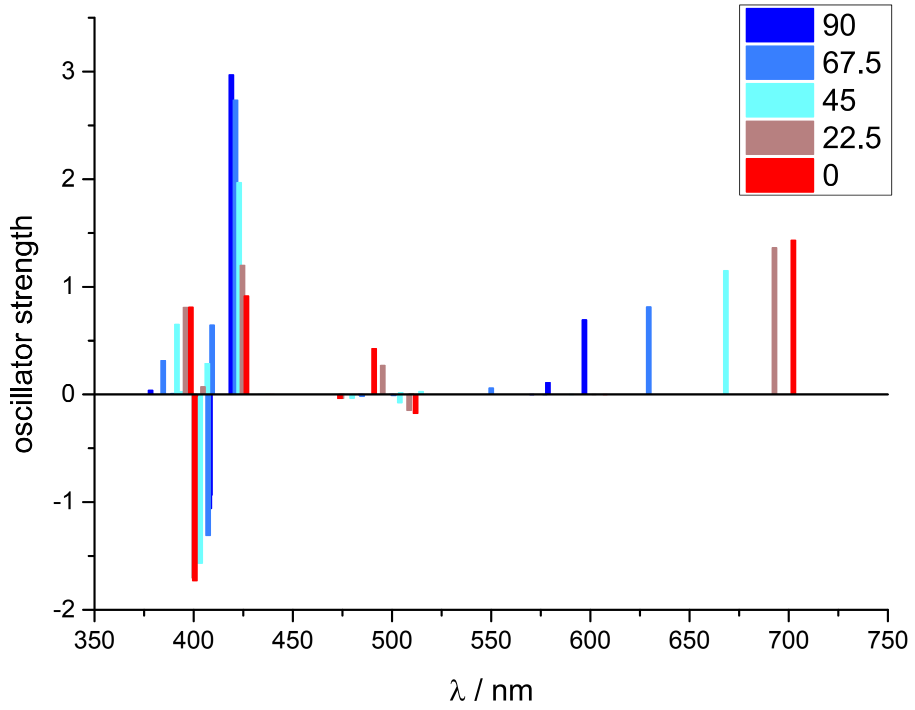

Figure S46: B3LYP/def2-TZVP predicted vertical excitations for various conformations of **2-ZnDPP(C_2H)$_2$**, from planar alignment (red) towards the perpendicular conformation (blue). Excitations with a transition dipole moment orientated along the Zn-Zn axis (x-axis) are plotted with positive oscillator strength, transitions along the y-axis are plotted with negative oscillator strength.

	ZnP				ZnDPP				ZnDPP(C$_2$H)$_2$				ZnTPP				1-Zn				2-ZnDPP				2-ZnDPP(C$_2$H)$_2$				2-Zn			
	Nr	eV	nm	OSC	Nr	eV	nm	OSC	Nr	eV	nm	OSC	Nr	eV	nm	OSC	Nr	eV	nm	OSC	Nr	eV	nm	OSC	Nr	eV	nm	OSC	Nr	eV	nm	OSC
Singlets	5	2.42	513	0.0026	5	2.35	527	0.0004	5	2.19	566	0.0785	5	2.31	537	0.0028	4	1.89	656	1.5806	7	1.88	661	1.1363	5	1.78	698	1.4323	5	1.60	777	3.1137
	6	2.42	513	0.0026	6	2.36	526	0.0022	6	2.20	564	0.0017	6	2.31	537	0.0029	8	2.05	606	0.0023	10	2.10	590	0.0033	10	2.06	603	0.0004	10	1.97	628	0
	15	3.42	362	0	15	3.35	370	1.5855	11	3.09	401	1.1803	12	3.26	381	1.2616	12	2.32	534	0	11	2.10	589	0	11	2.06	603	0	11	1.98	627	0.0022
	16	3.42	362	0	16	3.36	369	0.7351	15	3.20	388	1.1066	13	3.26	381	1.2598	13	2.37	523	0.0002	12	2.26	549	0	13	2.16	574	0	12	1.98	627	0
	18	3.50	354	0.9291	18	3.39	365	0	16	3.20	387	0	18	3.36	369	0	14	2.37	523	0.0078	14	2.36	526	0	14	2.22	559	0	15	2.02	613	0
	19	3.50	354	0.9291	19	3.40	365	0	19	3.39	366	0	19	3.36	369	0.0001	16	2.42	512	0.2038	16	2.50	495	0	16	2.44	509	0	21	2.22	558	0.0293
	21	3.74	331	0	21	3.68	337	0	20	3.43	361	0	21	3.63	342	0	27	2.68	463	0.2233	17	2.51	494	0.2061	17	2.44	508	0.1739	22	2.23	556	0.0005
	22	3.78	328	0	24	3.72	333	0	21	3.44	361	0.0318	22	3.67	338	0	28	2.68	462	0	20	2.63	472	0.8785	20	2.55	487	0.4247	23	2.23	556	0.0046
	25	3.82	325	0.0385	26	3.75	330	0.0309	23	3.45	359	0	23	3.69	336	0.0498	29	2.72	456	0.0002	23	2.73	454	0	23	2.64	470	0	25	2.31	538	0
	26	3.82	325	0.0386	28	3.76	330	0.0346	26	3.64	341	0	24	3.69	336	0.0502	30	2.72	456	0.0105	24	2.73	454	0.0043	24	2.64	469	0.0359	26	2.35	527	0.0002
	27	3.89	319	0	29	3.83	324	0	29	3.70	335	0	31	3.77	329	0	31	2.75	451	0	26	2.83	438	0	27	2.85	435	0.0002	27	2.36	526	0.1424
	28	3.97	312	0	30	3.90	318	0	31	3.72	334	0.0366	32	3.85	322	0	32	2.76	449	0.0090	28	2.83	438	0	29	2.85	435	0	28	2.36	526	0.0217
	30	4.19	296	0	33	4.01	310	0	33	3.74	331	0	33	3.98	312	0	33	2.76	449	0.0004	33	3.09	401	0.7942	32	2.94	422	0.9141	33	2.49	497	0
	31	4.26	291	0	34	4.05	306	0	34	3.78	328	0.0091	34	3.99	311	0	34	2.77	448	1.4172	34	3.19	389	0	34	3.09	401	0	34	2.50	497	0.0062
	32	4.27	290	0.1655	35	4.06	305	0.0007	35	3.82	325	0	35	4.00	310	0.0001	35	2.78	447	0	35	3.21	387	0	35	3.11	399	0	35	2.58	480	0
	33	4.27	290	0.1655	36	4.07	305	0	36	3.87	320	0	36	4.00	310	0.0001	36	2.78	447	0.0595	36	3.21	386	0.0026	36	3.13	397	0.0132	36	2.58	480	0
	37	4.67	265	0	37	4.13	300	0.0007	37	3.91	317	0.1502	37	4.01	309	0	37	2.89	430	0	37	3.22	384	0	37	3.13	396	1.7294	37	2.62	474	0
	38	4.83	257	0	38	4.17	297	0	38	3.94	315	0.0004	38	4.04	307	0.0009	38	2.89	430	0.0005	38	3.24	382	1.8079	38	3.15	394	0.8111	38	2.63	471	1.6882
	39	5.06	245	0	39	4.19	296	0.0134	39	3.94	314	0	39	4.06	305	0	39	2.94	422	1.3502	39	3.35	370	0.3316	39	3.15	394	0	39	2.64	470	0.0002
	40	5.11	243	0	40	4.21	294	0	40	3.96	313	0	40	4.10	302	0.0014	40	2.98	416	0.4235	40	3.40	365	0	40	3.23	383	0	40	2.64	470	0.0013

	ZnP			ZnDPP			ZnDPP(C$_2$H)$_2$			ZnTPP			1-Zn			2-ZnDPP			2-ZnDPP(C$_2$H)$_2$			2-Zn		
	Nr	eV	nm	Nr	eV	nm	Nr	eV	nm	Nr	eV	nm	Nr	eV	nm	Nr	eV	nm	Nr	eV	nm	Nr	eV	nm
Triplets	1	1.78	698	1	1.71	726	1	1.44	862	1	1.67	744	1	1.25	989	1	1.27	979	1	1.17	1064	1	1.07	1154
	2	1.78	698	2	1.73	718	2	1.64	757	2	1.67	744	2	1.56	795	2	1.59	779	2	1.46	852	2	1.34	927
	3	2.07	600	3	2.04	609	3	1.88	659	3	2.02	614	3	1.78	696	3	1.62	764	3	1.59	780	3	1.54	805
	4	2.07	600	4	2.04	607	4	2.03	612	4	2.02	614	5	1.98	627	4	1.62	764	4	1.59	779	4	1.54	804
	7	3.20	387	7	3.17	392	7	2.79	445	7	3.13	396	6	1.99	623	5	1.84	673	6	1.79	692	6	1.73	715
	8	3.24	382	8	3.21	386	8	3.02	411	8	3.17	391	7	2.04	608	6	1.84	673	7	1.79	692	7	1.73	715
	9	3.27	380	9	3.23	384	9	3.02	411	9	3.19	389	9	2.23	556	8	2.01	616	8	2.02	615	8	1.90	652
	10	3.27	380	10	3.23	383	10	3.04	407	10	3.19	389	10	2.30	538	9	2.03	612	9	2.03	612	9	1.95	637
	11	3.32	374	11	3.27	379	12	3.12	398	11	3.24	383	11	2.30	538	13	2.27	546	12	2.11	587	13	1.98	626
	12	3.32	374	12	3.29	377	13	3.15	394	14	3.26	381	15	2.37	523	15	2.45	506	15	2.26	549	14	1.99	622
	13	3.35	370	13	3.30	376	14	3.18	390	15	3.26	380	17	2.46	504	18	2.61	474	18	2.52	492	16	2.03	612
	14	3.42	362	14	3.34	371	17	3.21	387	16	3.26	380	18	2.46	504	19	2.61	474	19	2.52	492	17	2.12	584
	17	3.45	360	17	3.39	366	18	3.35	370	17	3.34	371	19	2.47	501	21	2.65	468	21	2.58	481	18	2.19	566
	20	3.66	338	20	3.62	342	22	3.45	360	20	3.59	345	20	2.50	497	22	2.65	468	22	2.58	481	19	2.19	566
	23	3.79	327	22	3.71	334	24	3.56	349	25	3.70	335	21	2.58	480	25	2.83	438	25	2.67	464	20	2.20	564
	24	3.79	327	23	3.72	334	25	3.56	349	26	3.70	335	22	2.59	479	27	2.83	438	26	2.84	437	24	2.26	548
	29	4.12	301	25	3.75	331	27	3.66	339	27	3.72	333	23	2.59	479	29	2.88	430	28	2.85	435	29	2.39	518
	34	4.29	289	27	3.75	330	28	3.67	337	28	3.74	331	24	2.62	474	30	3.02	411	30	2.85	435	30	2.40	518
	35	4.41	281	31	3.93	315	30	3.71	334	29	3.75	331	25	2.62	473	31	3.02	410	31	2.90	428	31	2.46	504
	36	4.53	274	32	3.96	313	32	3.72	333	30	3.75	331	26	2.65	468	32	3.02	410	33	2.96	419	32	2.46	504

Table S7: Vertical excitations and oscillator strengths calculated with B3LYP/def2-TZVP in the gas phase.

	ZnP				ZnDPP				$ZnDPP(C_2H)_2$				ZnTPP				1-Zn				2-ZnDPP				$2\text{-}ZnDPP(C_2H)_2$				2-Zn			
	Nr	eV	nm	OSC	Nr	eV	nm	OSC	Nr	eV	nm	OSC	Nr	eV	nm	OSC	Nr	eV	nm	OSC	Nr	eV	nm	OSC	Nr	eV	nm	OSC	Nr	eV	nm	OSC
Singlets	5	2.35	527	0.0072	5	2.30	539	0.0001	5	2.16	575	0.0577	5	2.26	549	0.0005	1	1.97	629	0.9112	8	2.00	621	0.6518	5	1.90	651	0.9802	1	1.77	700	2.3107
	6	2.35	527	0.0072	6	2.30	539	0.0026	6	2.17	571	0.0094	6	2.26	549	0.0005	2	2.09	592	0.0006	10	2.18	568	0.0017	10	2.13	583	0	2	2.08	597	0
	11	3.63	341	1.0779	11	3.50	354	1.7537	10	3.29	377	1.3756	9	3.41	364	1.4592	3	2.96	419	3.7039	11	2.18	568	0	11	2.13	583	0.0138	3	2.08	597	0.0032
	12	3.63	341	1.0779	13	3.52	353	0.8926	12	3.34	371	1.3067	10	3.41	364	1.4577	4	3.12	398	0.9018	12	2.25	551	0	12	2.18	570	0	4	2.08	597	0
	18	3.94	315	0	20	3.91	317	0	19	3.73	332	0	22	3.89	319	0	5	3.12	397	0	14	2.89	428	2.4056	15	2.87	432	2.2844	5	2.80	442	3.4459
	19	3.94	315	0	21	3.93	316	0	21	3.91	317	0	23	3.89	319	0	6	3.13	397	0.0168	16	3.16	392	0	16	3.10	399	0	6	2.93	423	0
	21	4.21	295	0	23	4.15	298	0	22	3.94	315	0	24	4.10	302	0	7	3.15	394	0	17	3.20	387	1.8548	17	3.11	399	0	7	3.03	410	0.0014
	22	4.27	290	0	24	4.21	294	0	23	3.99	310	0	26	4.16	298	0	8	3.32	373	0.1879	19	3.25	382	0	19	3.15	393	1.8513	8	3.06	406	0.0002
	25	4.38	283	0.0509	27	4.32	287	0.0703	25	4.02	308	0.0602	29	4.27	291	0.0829	9	3.41	364	0	29	3.49	355	0.0001	29	3.41	364	0	9	3.06	405	1.3663
	26	4.38	283	0.0509	28	4.32	287	0.0521	29	4.24	292	0.0868	30	4.27	291	0.0827	10	3.57	347	0.0021	30	3.73	333	0	30	3.55	349	0.8266	10	3.07	404	1.6174
	27	4.53	274	0	29	4.47	277	0	31	4.28	289	0	31	4.41	281	0	11	3.57	347	0.1071	31	3.73	333	0.0098	31	3.60	345	0	11	3.12	398	0.0006
	29	4.72	262	0	32	4.63	268	0	32	4.40	282	0	32	4.58	270	0	12	3.64	340	0.0004	32	3.73	332	0	32	3.60	344	0.1854	12	3.12	398	0.0034
	31	4.92	252	0	33	4.80	258	0	33	4.49	276	0	33	4.71	263	0	13	3.70	335	0.0029	33	3.75	331	0.5009	33	3.66	339	0	13	3.33	372	0.0001
	32	4.94	251	0	34	4.85	255	0	34	4.63	268	0	34	4.80	258	0	14	3.70	335	0.1519	34	3.79	327	0	34	3.71	334	0.0004	14	3.35	370	0.4747
	33	5.03	247	0.1772	35	4.88	254	0	35	4.69	264	0	35	4.85	256	0.0001	15	3.71	334	0	35	3.80	326	0	35	3.72	333	0	15	3.50	355	0.3596
	34	5.03	247	0.1772	36	4.91	253	0.0634	36	4.69	264	0	36	4.87	255	0.0485	16	3.77	329	0	36	3.83	324	0	36	3.75	331	0	16	3.50	354	0.0026
	37	5.25	236	0	37	4.96	250	0.1322	37	4.70	264	0.0893	37	4.87	255	0.0460	17	3.79	327	0.0005	37	3.84	323	1.0121	37	3.75	330	0.4361	17	3.50	354	0.0624
	38	5.41	229	0	38	4.98	249	0	38	4.82	257	0	38	4.87	254	0.0001	18	3.81	325	0	38	3.92	317	0	38	3.86	322	0.0045	18	3.54	351	0.0001
	39	5.56	223	0	39	4.99	248	0.1596	39	4.83	257	0.0323	39	4.93	252	0.1345	19	3.81	325	0.0158	39	3.97	312	0	39	3.91	317	0	19	3.54	350	0.1279
	40	5.58	222	0	40	5.13	242	0.0026	40	4.84	256	0.0624	40	4.93	252	0.1327	20	3.88	319	0	40	3.97	312	0	40	3.91	317	0.0002	20	3.54	350	0

	ZnP			ZnDPP			$ZnDPP(C_2H)_2$			ZnTPP			1-Zn			2-ZnDPP			$2\text{-}ZnDPP(C_2H)_2$			2-Zn		
	Nr	eV	nm	Nr	eV	nm	Nr	eV	nm	Nr	eV	nm	Nr	eV	nm	Nr	eV	nm	Nr	eV	nm	Nr	eV	nm
Triplets	1	1.24	998	1	1.18	1053	1	0.95	1305	1	1.15	1079	1	0.79	1566	1	0.86	1446	1	0.72	1731	1	1.07	1154
	2	1.24	998	2	1.21	1021	2	1.10	1132	2	1.15	1079	2	1.06	1165	2	1.14	1088	2	1.02	1212	2	1.34	927
	3	2.12	584	3	2.08	595	3	1.98	626	3	2.06	602	3	1.77	702	3	1.14	1087	3	1.09	1135	3	1.54	805
	4	2.12	584	4	2.09	593	4	2.01	616	4	2.06	602	4	1.79	694	4	1.16	1070	4	1.09	1133	4	1.54	804
	7	3.38	366	7	3.36	369	7	3.01	411	7	3.33	373	5	1.90	652	5	1.97	629	6	1.94	639	6	1.73	715
	8	3.44	361	8	3.42	363	8	3.22	385	8	3.39	366	6	1.96	631	6	1.97	629	7	1.94	639	7	1.73	715
	9	3.50	355	9	3.47	358	9	3.28	378	11	3.44	360	7	2.42	513	7	1.98	625	8	1.98	627	8	1.90	652
	10	3.50	355	10	3.47	357	11	3.31	375	12	3.44	360	8	2.52	492	9	2.03	610	9	2.01	618	9	1.95	637
	13	3.65	340	12	3.50	354	13	3.36	369	13	3.49	355	9	2.54	489	13	2.66	465	13	2.49	499	13	1.98	626
	14	3.66	338	14	3.55	350	14	3.38	367	14	3.53	351	10	2.54	489	15	3.03	409	14	2.86	434	14	1.99	622
	15	3.69	336	15	3.58	346	15	3.49	355	15	3.55	349	11	2.67	464	18	3.24	383	18	3.15	394	16	2.03	612
	16	3.81	326	16	3.61	343	16	3.55	349	16	3.55	349	12	2.93	424	20	3.27	379	20	3.18	390	17	2.12	584
	17	3.81	326	17	3.64	340	17	3.56	348	17	3.55	349	13	2.95	421	21	3.27	379	21	3.21	387	18	2.19	566
	20	4.19	296	18	3.78	328	18	3.63	342	18	3.57	347	14	2.95	421	22	3.29	376	22	3.21	387	19	2.19	566
	23	4.34	286	19	3.80	327	20	3.86	321	19	3.57	347	15	3.00	414	23	3.31	374	23	3.26	381	20	2.20	564
	24	4.34	286	22	4.15	299	24	4.02	309	20	3.76	330	16	3.00	413	24	3.31	374	24	3.26	381	24	2.26	548
	28	4.58	271	25	4.29	289	26	4.16	298	21	3.76	330	17	3.00	413	25	3.37	368	25	3.27	379	29	2.39	518
	30	4.73	262	26	4.29	289	27	4.18	297	25	4.10	302	18	3.07	404	26	3.37	368	26	3.29	377	30	2.40	518
	35	5.03	247	30	4.48	277	28	4.21	295	27	4.24	292	19	3.12	398	27	3.43	362	27	3.29	377	31	2.46	504
	36	5.24	237	31	4.59	270	30	4.24	292	28	4.24	292	20	3.16	392	28	3.43	362	28	3.34	371	32	2.46	504

Table S8: Vertical excitations and oscillator strengths calculated with CAM-B3LYP/def2-TZVP in the gas phase.

	ZnP				ZnDPP				ZnDPP(C_2H)$_2$				ZnTPP				1-Zn				2-ZnDPP				2-ZnDPP(C_2H)$_2$				2-Zn			
	Nr	eV	nm	OSC	Nr	eV	nm	OSC	Nr	eV	nm	OSC	Nr	eV	nm	OSC	Nr	eV	nm	OSC	Nr	eV	nm	OSC	Nr	eV	nm	OSC	Nr	eV	nm	OSC
Singlets	6	1.64	756	0.0080	6	1.61	772	0.0090	6	1.55	799	0.0030	6	1.59	781	0	5	1.57	790	0.0030	8	1.60	776	0	10	1.56	796	0	8	1.58	783	0.0010
	7	2.15	577	0.0220	7	2.14	581	0.0070	7	2.08	597	0.0080	7	2.12	585	0.0020	10	2.06	601	0.2620	9	1.60	775	0.0020	11	1.56	795	0.0060	9	1.59	782	0.0050
	15	3.21	386	1.0330	17	3.10	400	1.3560	17	2.94	421	1.7310	19	3.03	410	1.7450	31	2.59	479	0.2840	12	2.13	581	0.1800	12	2.03	612	0.2900	14	2.02	615	0.7550
	16	3.28	378	1.1340	18	3.15	394	1.5730	18	3.10	400	1.6260	20	3.09	402	1.8520	32	2.60	478	0.0020	15	2.21	561	0	13	2.11	589	0	15	2.11	587	0
	23	3.63	342	0.0230	28	3.57	347	0.0170	29	3.49	356	0.0200	33	3.58	346	0.0190	36	2.66	466	0	31	2.75	451	3.2350	31	2.74	453	3.5780	34	2.64	470	0.7270
	24	3.67	338	0.0010	30	3.63	341	0.0020	30	3.55	349	0	35	3.60	345	0.0010	37	2.66	466	0	34	2.99	414	0	34	2.99	414	0	35	2.65	469	0
	25	3.78	328	0.2460	31	3.71	334	0.1910	33	3.61	344	0.2120	37	3.67	338	0.2750	40	2.86	434	4.5970	36	3.05	406	3.5960	36	3.05	406	3.6690	40	2.67	465	0
	26	3.92	317	0.0010	32	3.76	329	0.0090	34	3.70	335	0.0020	38	3.71	334	0.0150	48	3.07	404	1.3800	37	3.08	403	0	37	3.09	401	0	41	2.67	465	0
	29	4.04	307	0.0060	33	3.88	320	0	36	3.82	325	0.1840	39	3.87	320	0.1330	52	3.15	394	0.0350	50	3.39	366	0.0020	43	3.35	371	0.0020	48	2.74	452	5.4860
	30	4.04	307	0.2230	35	3.89	319	0.0010	38	3.84	323	0.2570	40	3.88	319	0	59	3.28	378	0	56	3.54	350	0	57	3.49	355	0	61	3.09	402	0.0010
	32	4.06	306	0.3160	37	3.89	319	0.0010	40	3.88	319	0.0050	42	3.88	319	0.0010	60	3.28	378	0.0010	58	3.55	350	0.0440	59	3.50	354	0.4840	62	3.09	401	0.0080
	33	4.11	302	0.0600	39	3.92	316	0.2490	42	3.89	319	0	44	3.89	319	0.0010	67	3.34	371	0.0100	61	3.63	342	0.0210	60	3.50	354	0.1450	68	3.15	394	3.1380
	34	4.14	300	0	42	3.98	312	0.1730	43	3.89	319	0.0020	45	3.89	319	0.0060	68	3.35	371	0.0010	62	3.64	341	0	61	3.58	347	0	69	3.18	390	1.0210
	38	4.47	278	0.1460	43	4.02	308	0.0840	45	3.92	316	0.0460	46	3.89	318	0	70	3.35	370	0.0540	63	3.66	339	0.4540	62	3.61	343	0	76	3.27	380	0
	41	4.55	273	0.0220	44	4.08	304	0.0010	49	4.03	308	0.0010	49	3.93	316	0.1030	72	3.37	368	0	64	3.70	335	0	64	3.62	343	0.4670	77	3.31	375	0
	46	4.66	266	0.0020	45	4.10	303	0.0010	50	4.04	307	0.0050	53	3.97	312	0.2110	74	3.37	368	0.0010	65	3.71	335	0.6900	65	3.62	342	0.0630	78	3.31	375	0.0030
	49	4.72	263	0.0240	47	4.17	297	0.0710	51	4.12	301	0.0650	54	4.04	307	0.0270	81	3.39	366	0.0050	70	3.84	323	0	70	3.76	330	0	84	3.36	369	0
	50	4.72	263	0	50	4.28	290	0.0640	54	4.17	297	0.0480	55	4.04	307	0.0280	88	3.51	354	0	71	3.85	322	0.0230	73	3.81	325	0	85	3.36	369	0
	51	4.75	261	0	54	4.45	278	0.0390	55	4.18	297	0	56	4.09	303	0.0690	89	3.53	351	0.0010	72	3.88	319	0	74	3.83	324	0	98	3.42	362	0
	52	4.76	261	0.1680	55	4.49	276	0.0130	60	4.40	282	0.0760	57	4.09	303	0.0010	90	3.55	350	0.0700	74	3.89	319	0.0370	75	3.83	324	0.0030	101	3.43	361	0

	ZnP			ZnDPP			ZnDPP(C_2H)$_2$			ZnTPP			1-Zn			2-ZnDPP			2-ZnDPP(C_2H)$_2$			2-Zn		
	Nr	eV	nm	Nr	eV	nm	Nr	eV	nm	Nr	eV	nm	Nr	eV	nm	Nr	eV	nm	Nr	eV	nm	Nr	eV	nm
Triplets	2	1.00	1245	2	0.97	1276	2	0.91	1361	2	0.96	1288	2	1.03	1203	2	0.99	1251	2	0.94	1322	2	0.94	1322
	3	1.37	905	3	1.34	927	3	1.28	968	3	1.32	942	3	1.03	1202	3	0.99	1251	3	0.94	1321	3	0.94	1321
	4	1.40	884	4	1.39	893	4	1.33	936	4	1.35	920	4	1.38	896	4	1.35	921	4	1.33	935	4	1.33	935
	5	1.60	776	5	1.57	789	5	1.51	822	5	1.58	783	5	1.39	895	5	1.35	918	5	1.33	935	5	1.33	935
	8	2.18	569	8	2.15	576	8	2.15	578	8	2.18	568	6	1.42	872	6	1.35	918	6	1.33	931	6	1.33	931
	9	2.19	566	9	2.16	574	9	2.15	576	9	2.19	566	7	1.45	857	7	1.37	909	7	1.36	915	7	1.36	915
	10	2.20	564	10	2.16	573	10	2.17	572	10	2.20	564	10	1.63	760	10	1.60	773	8	1.52	817	8	1.52	817
	11	2.53	491	11	2.20	565	11	2.18	570	11	2.21	562	11	1.66	749	11	1.65	751	9	1.55	802	9	1.55	802
	12	2.54	488	12	2.24	554	12	2.23	556	12	2.21	561	12	1.81	686	13	2.18	569	14	2.19	566	14	2.19	566
	13	2.65	468	13	2.48	501	13	2.36	526	13	2.21	561	13	1.81	686	14	2.18	569	15	2.19	566	15	2.19	566
	14	2.88	430	14	2.49	497	14	2.42	513	14	2.23	557	16	2.23	556	16	2.23	556	16	2.20	563	16	2.20	563
	17	3.30	375	15	2.61	475	15	2.54	488	15	2.46	503	17	2.24	554	17	2.24	555	17	2.21	561	17	2.21	561
	18	3.41	364	16	2.90	428	16	2.82	440	16	2.48	500	18	2.27	546	18	2.28	545	18	2.26	548	18	2.26	548
	19	3.42	363	19	3.22	386	19	3.11	398	17	2.68	462	19	2.27	546	19	2.28	545	19	2.28	544	19	2.28	544
	20	3.44	360	20	3.32	374	20	3.18	390	18	2.85	434	20	2.32	534	20	2.28	545	20	2.28	543	20	2.28	543
	21	3.62	343	21	3.32	373	21	3.30	376	21	3.20	387	21	2.32	534	21	2.28	544	21	2.28	543	21	2.28	543
	22	3.62	342	22	3.32	373	22	3.32	373	22	3.33	373	22	2.35	529	22	2.34	531	22	2.28	543	22	2.28	543
	27	3.97	313	23	3.33	372	23	3.33	373	23	3.33	372	23	2.38	522	23	2.34	531	23	2.33	531	23	2.33	531
	28	3.99	311	24	3.39	366	24	3.33	372	24	3.33	372	24	2.47	501	24	2.36	525	24	2.33	531	24	2.33	531
	31	4.05	306	25	3.40	365	25	3.34	371	25	3.34	372	25	2.51	493	25	2.46	504	25	2.40	517	25	2.40	517

Table S9: Vertical excitations and oscillator strengths calculated with AM1 in the gas phase.

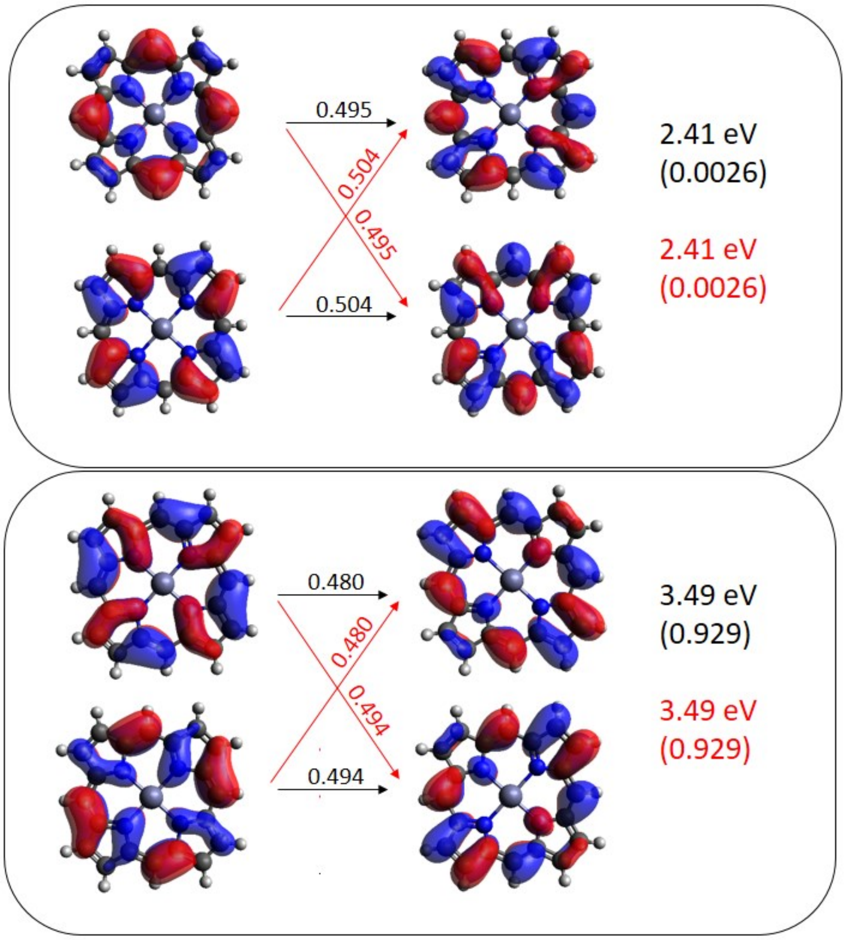

Figure S47: Natural transition orbitals for the Q- and Soret band transition of **ZnP**, predicted with B3LYP/TZVP//B3LYP/TZVP in gas phase. The eigenvalue associated with each NTO hole/electron pair (left/right, 0.02 isovalue) is presented on the arrow, vertical energy and oscillator strength (in brackets) on the right.

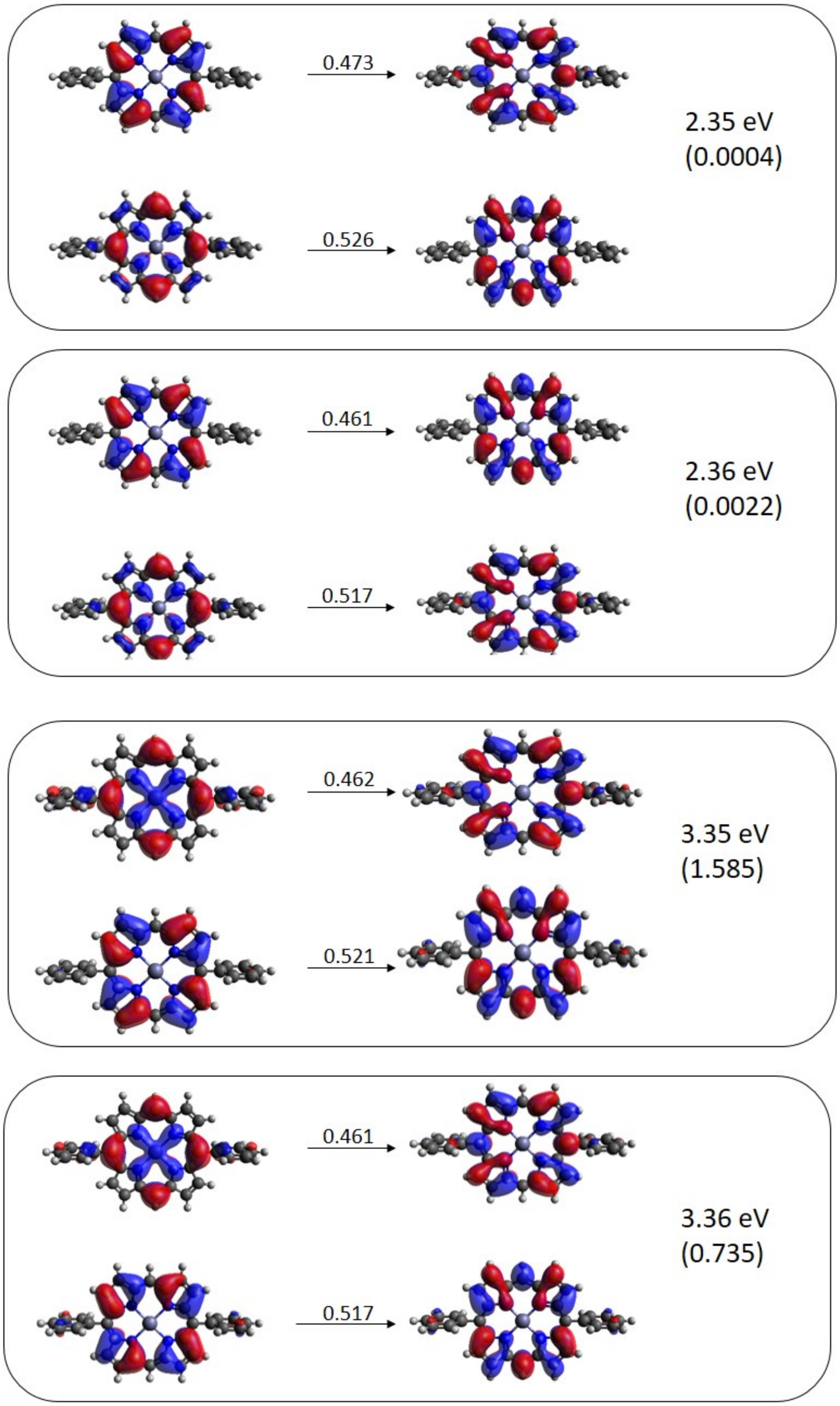

Figure S48: Natural transition orbitals for the Q- and Soret band transition of ZnDPP, predicted with B3LYP/TZVP//B3LYP/TZVP in gas phase. The eigenvalue associated with each NTO hole/electron pair left/right, 0.02 isovalue) is presented on the arrow, vertical energy and oscillator strength (in brackets) on the right.

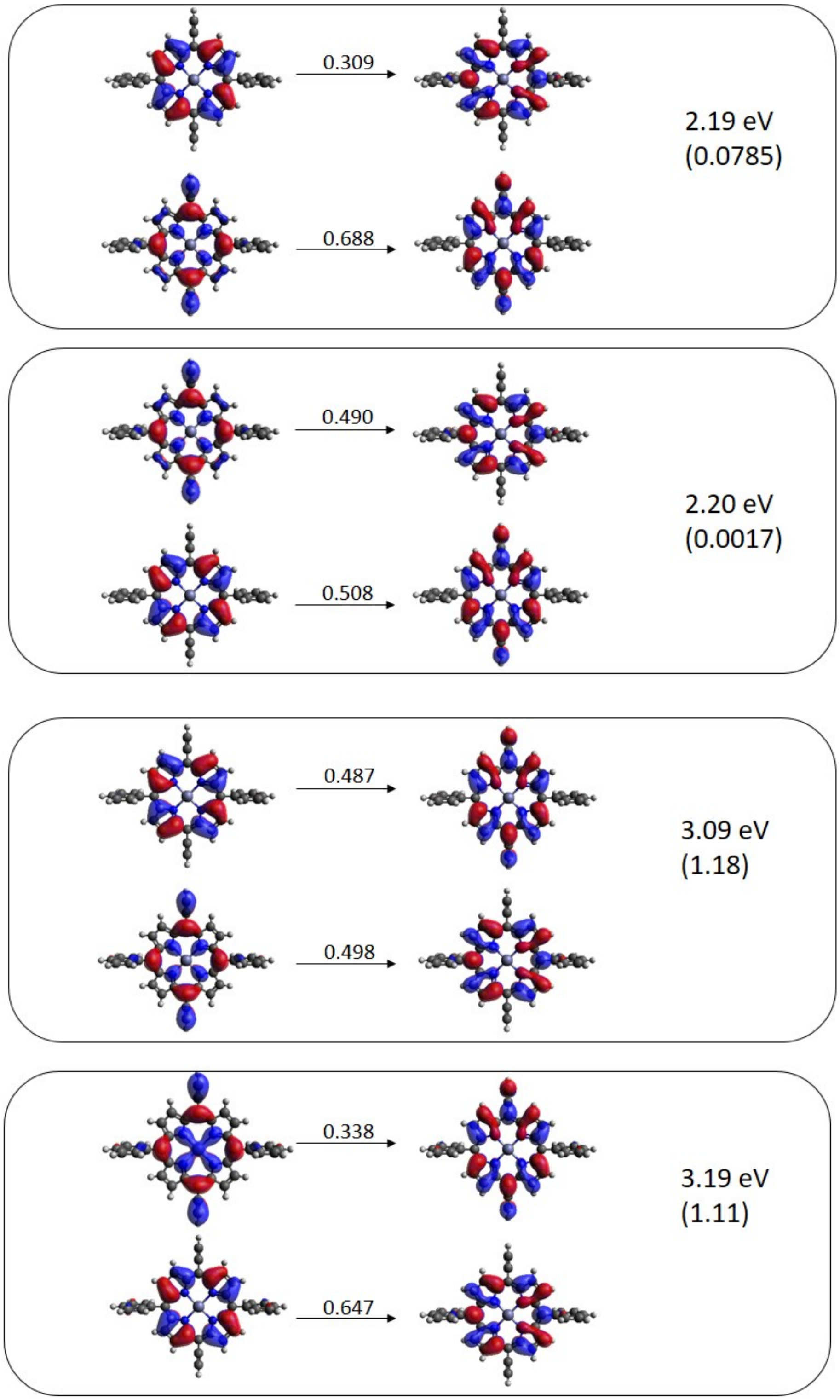

Figure S49: Natural transition orbitals for the Q- and Soret band transition of ZnDPP(C_2H)$_2$, predicted with B3LYP/TZVP//B3LYP/TZVP in gas phase. The eigenvalue associated with each NTO hole/electron pair (left/right, 0.02 isovalue) is presented on the arrow, vertical energy and oscillator strength (in brackets) on the right.

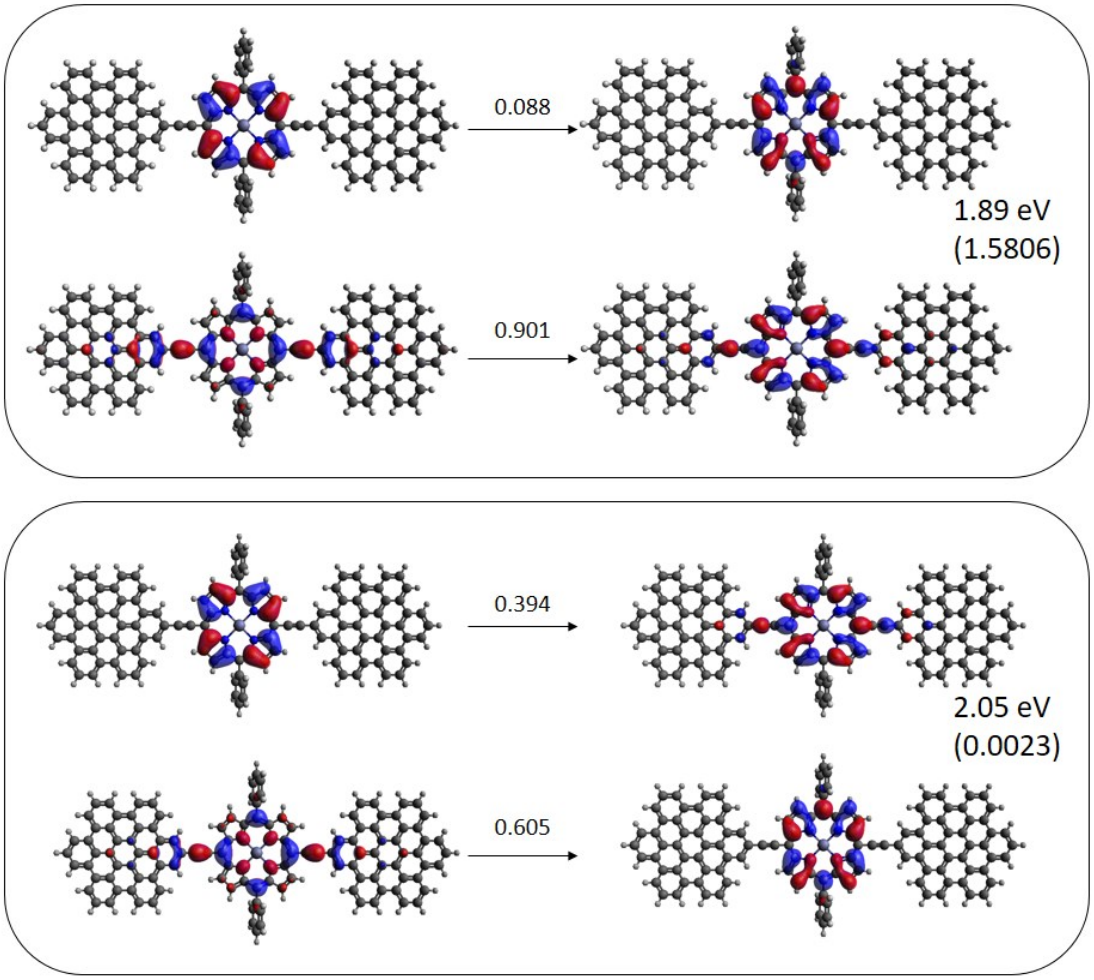

Figure S50: Natural transition orbitals for the Q- band transition of 1-Zn, predicted with B3LYP/TZVP//B3LYP/TZVP in gas phase. The eigenvalue associated with each NTO hole/electron pair (left/right, 0.02 isovalue) is presented on the arrow, vertical energy and oscillator strength (in brackets) on the right.

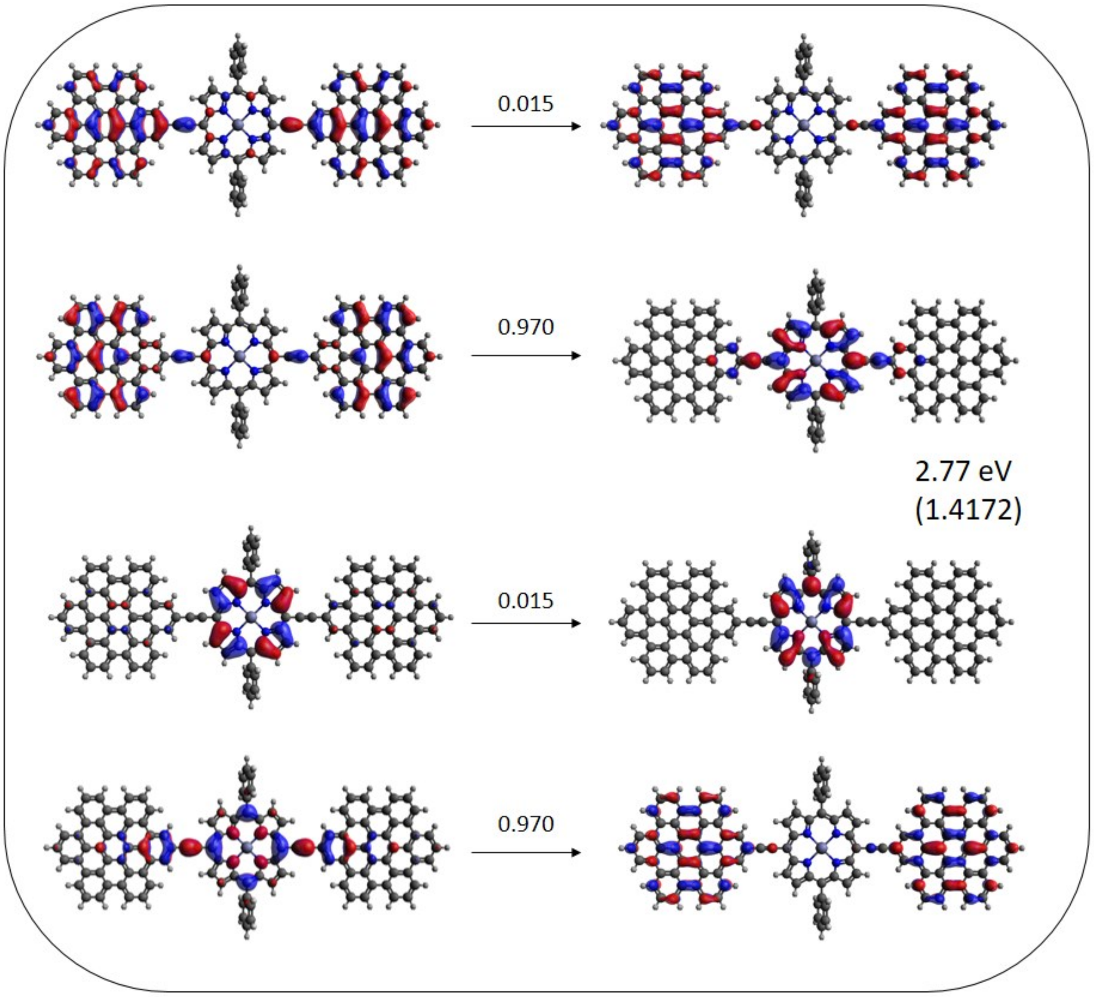
0.015
0.970
2.77 eV
(1.4172)
0.015
0.970

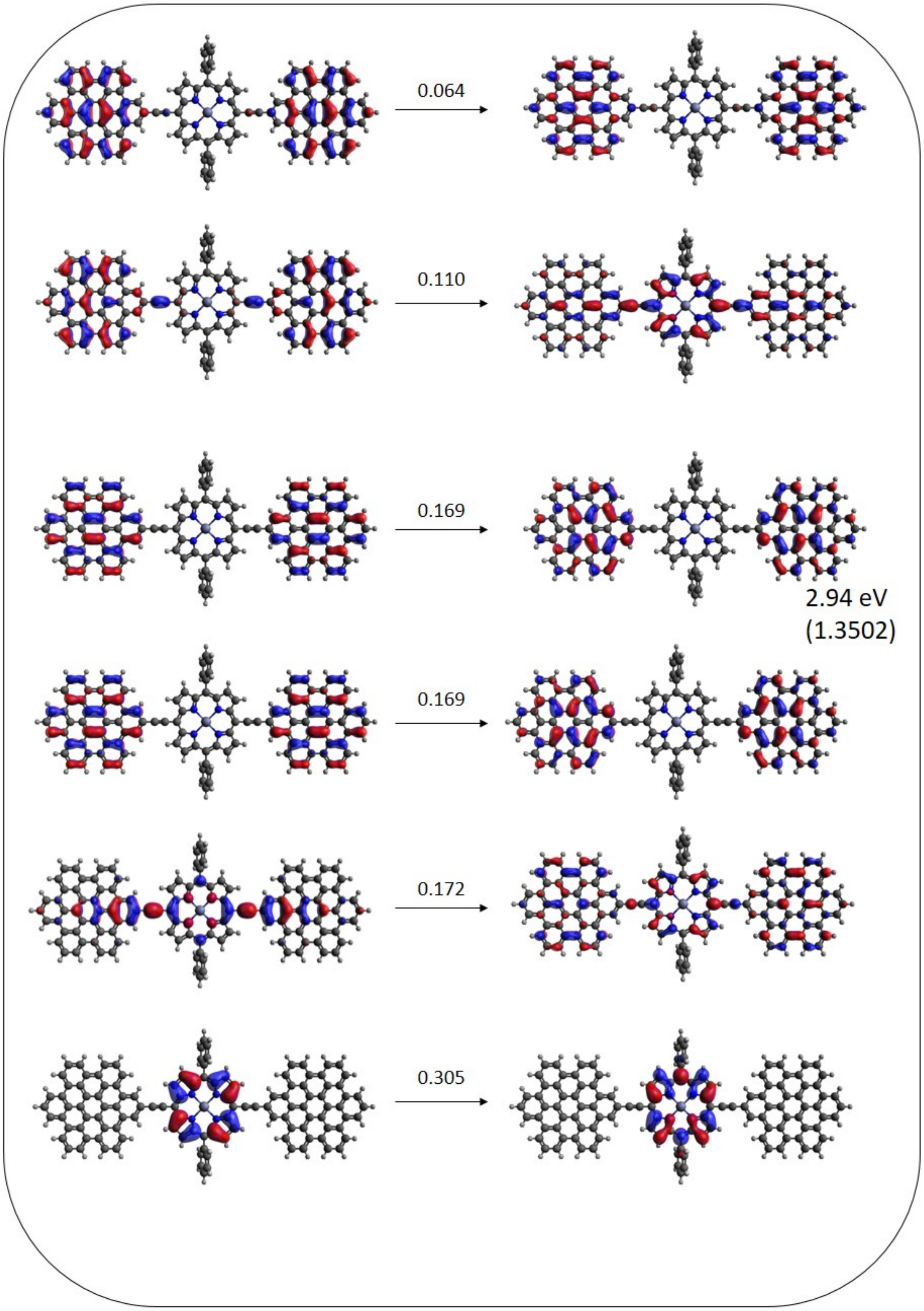

Figure S51: Natural transition orbitals for the Soret- band transition of 1-Zn, predicted with B3LYP/TZVP//B3LYP/TZVP in gas phase. The eigenvalue associated with each NTO hole/electron pair (left/right, 0.02 isovalue) is presented on the arrow, vertical energy and oscillator strength (in brackets) on the right.

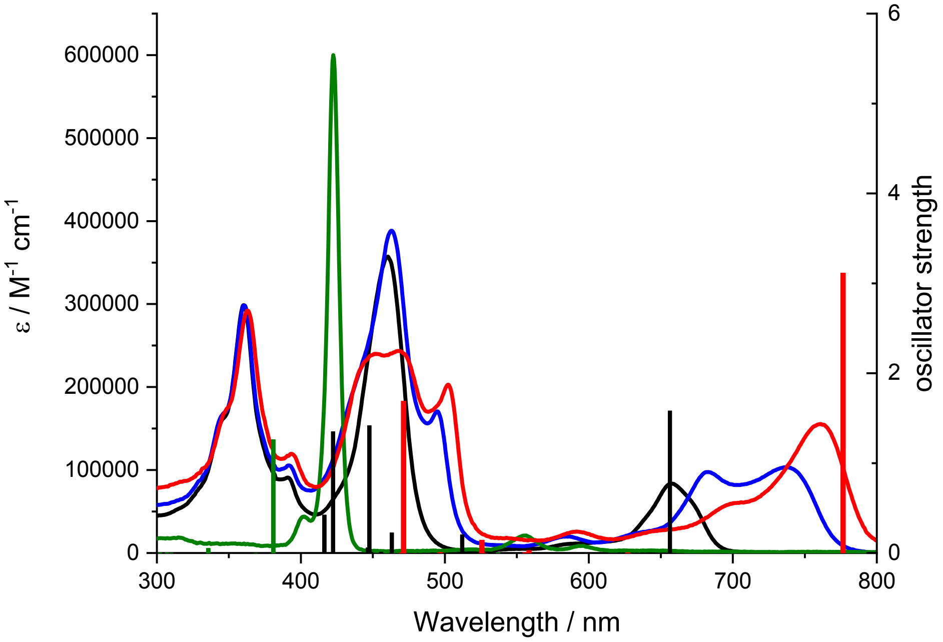

Figure S52: Vertical energies predicted with B3LYP/TZVP//B3LYP/TZVP of **1-Zn**, **2-Zn**, and **ZnTPP**. **1-Zn** (black bars), **2-Zn** (red bars) and **ZnTPP** (green bars) in gas phase. Measured spectra of **ZnTPP** (green), **1-Zn** (black), and **2-Zn** (blue). In addition the planarized **2-Zn** hybrid with **1,10-DA** is shown in red.

4. Spectral Appe

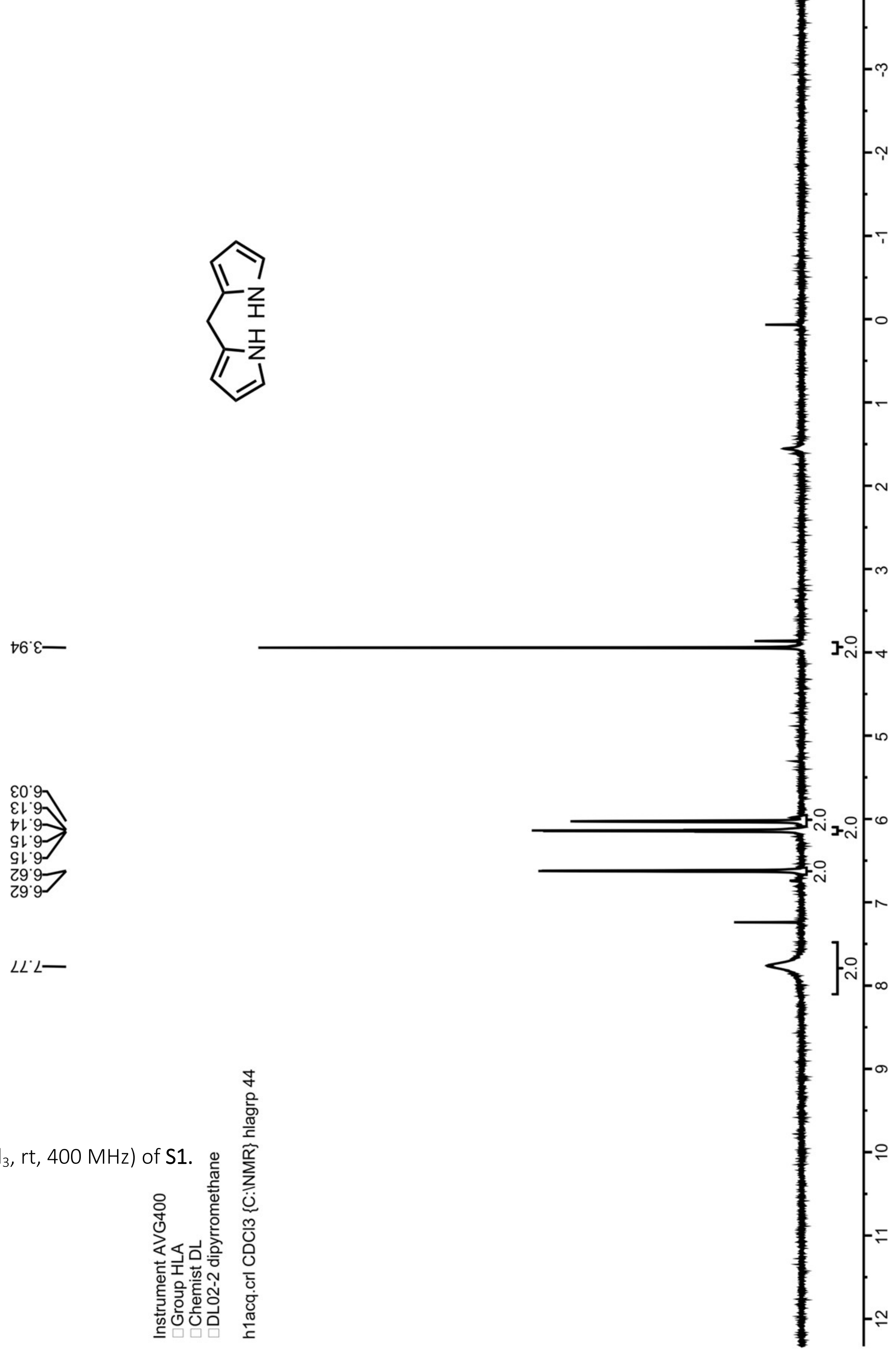

Figure S53: ^{1}H NMR ($CDCl_3$, rt, 400 MHz) of **S1**.

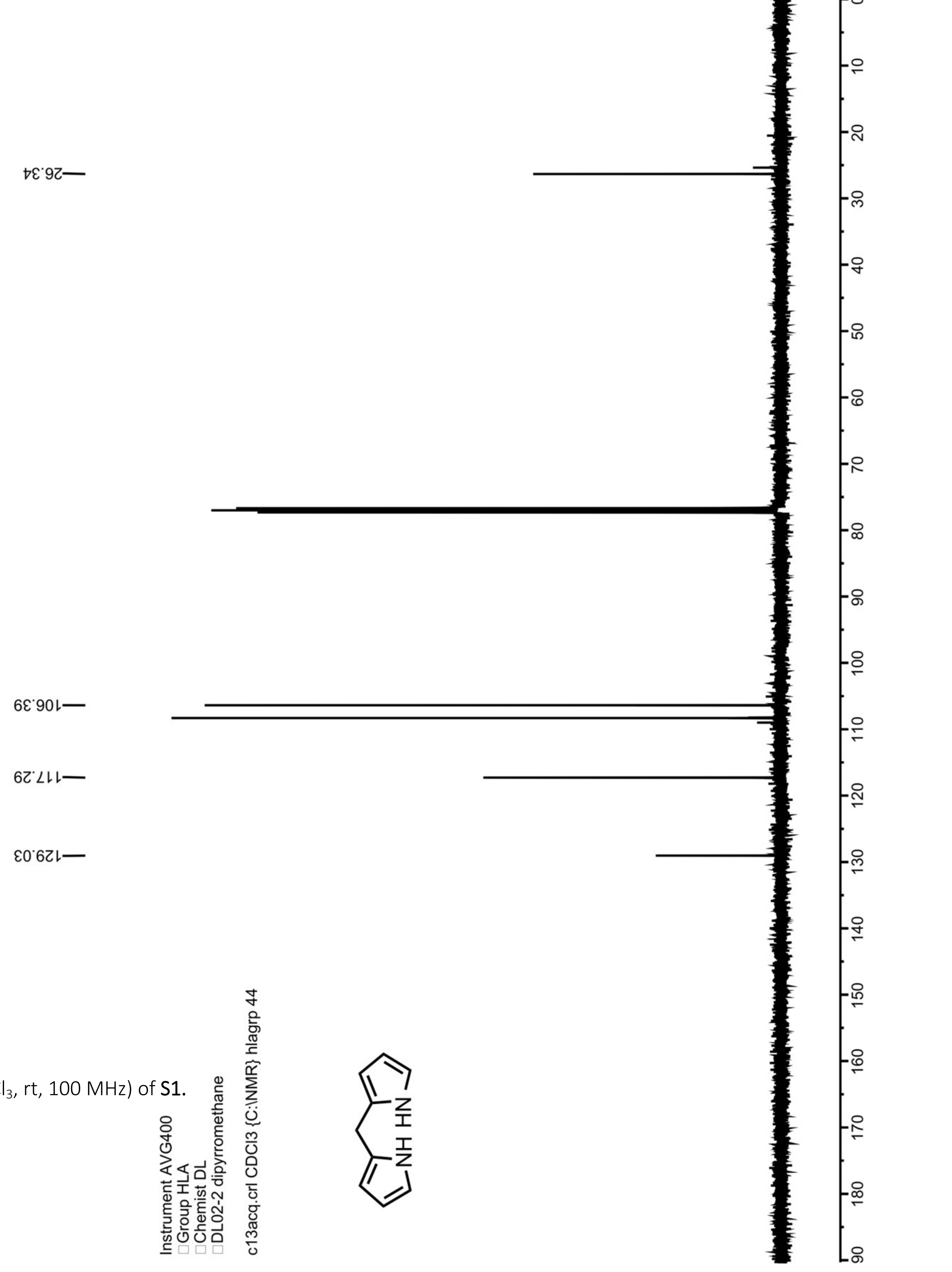

Figure S54: ^{13}C NMR ($CDCl_3$, rt, 100 MHz) of **S1**.

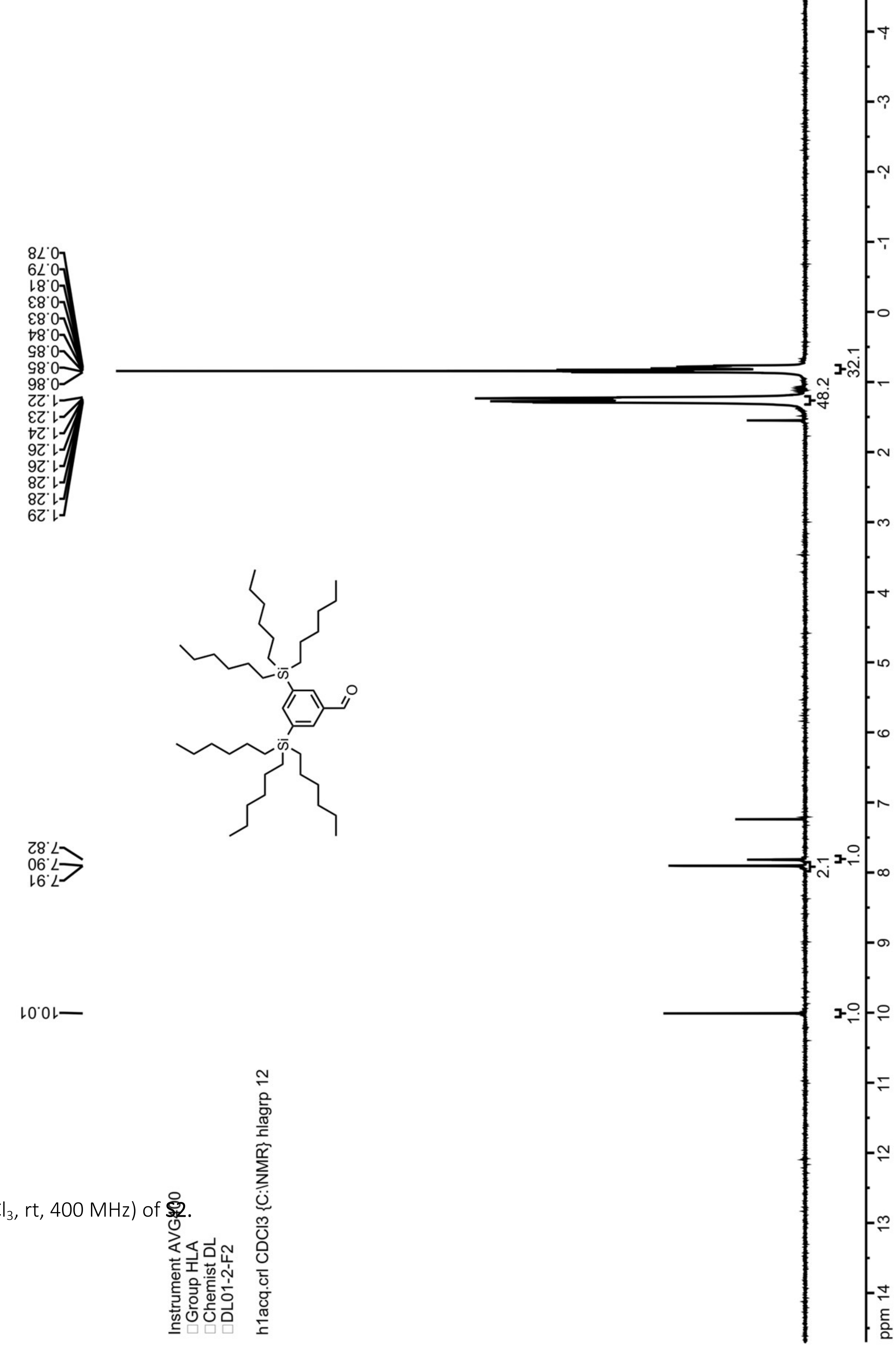

Figure S55: ^{1}H NMR ($CDCl_3$, rt, 400 MHz) of **S2**.

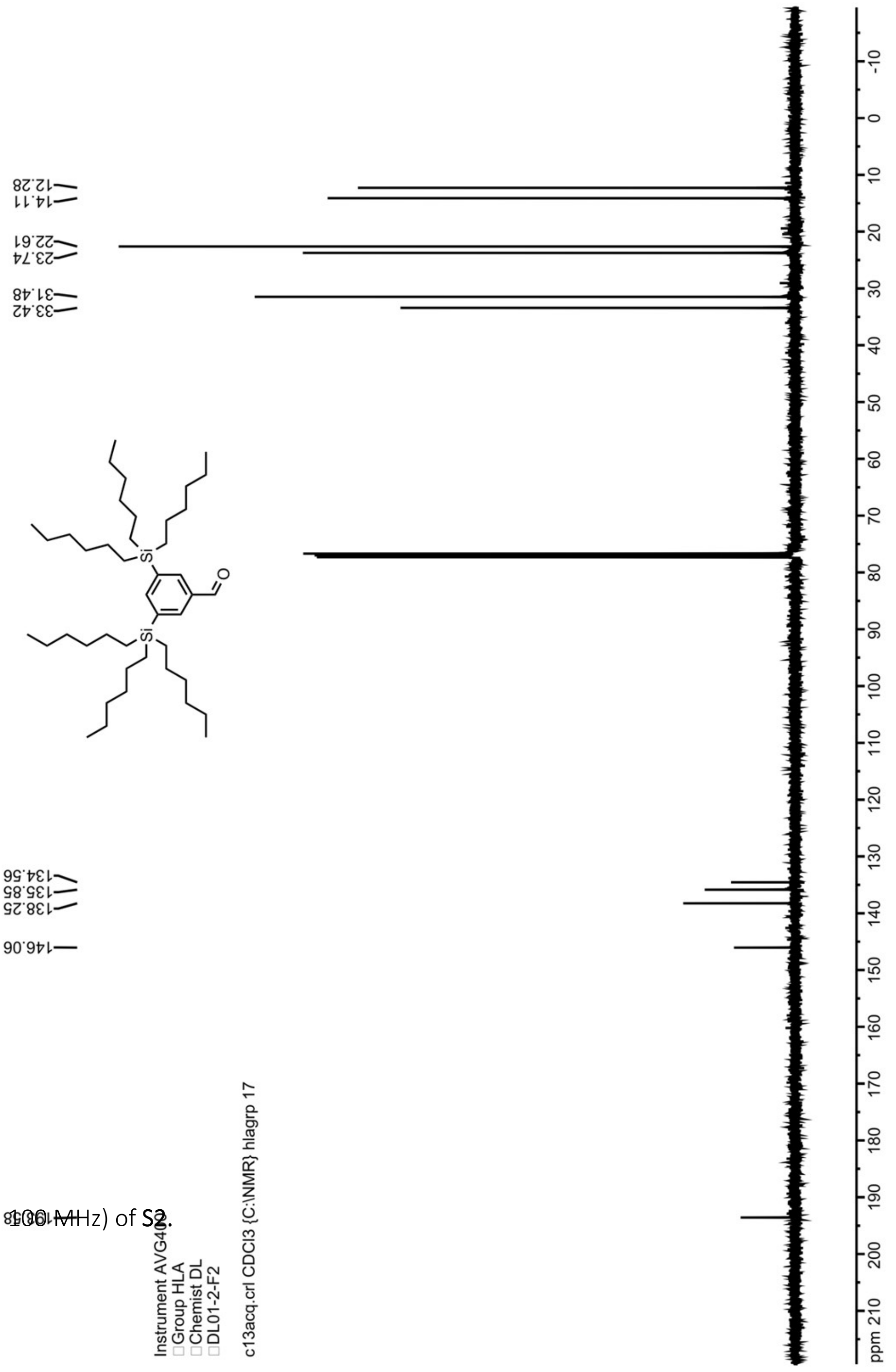

Figure S56: ^{13}C NMR ($CDCl_3$, rt, 100 MHz) of **S2**.

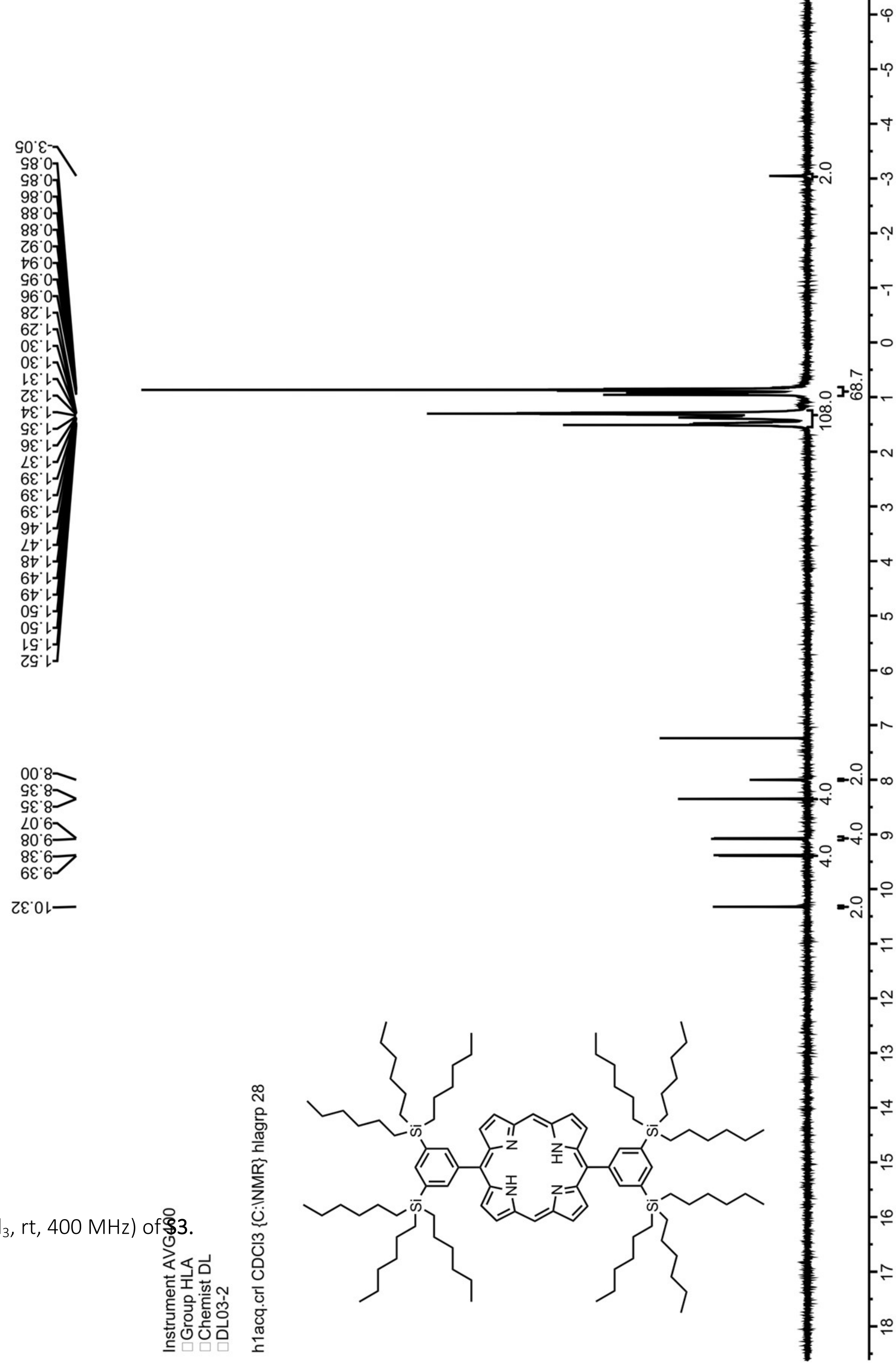

Figure S57: ^{1}H NMR ($CDCl_3$, rt, 400 MHz) of **33.**

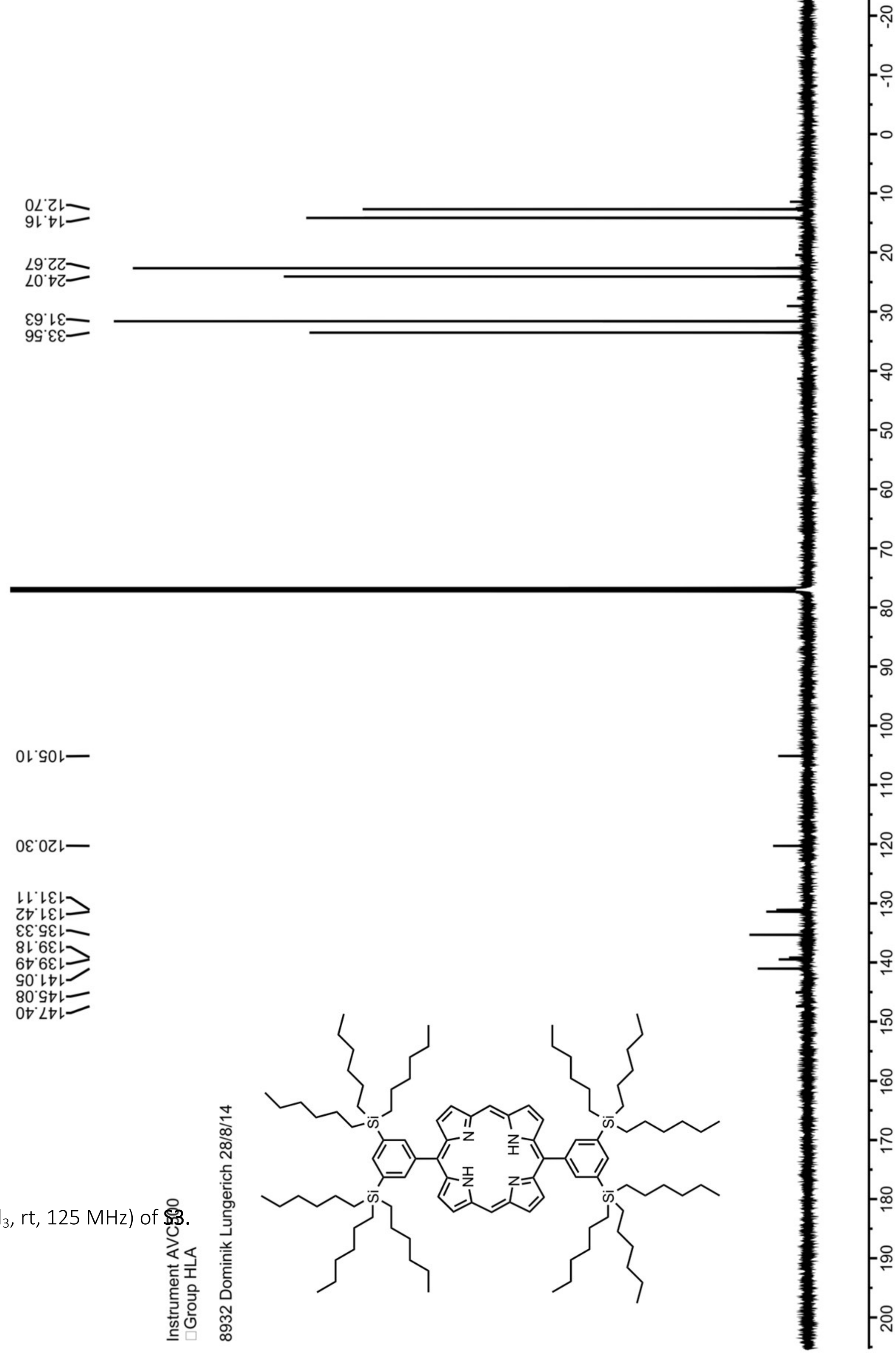

Figure S58: ^{13}C NMR ($CDCl_3$, rt, 125 MHz) of S3.

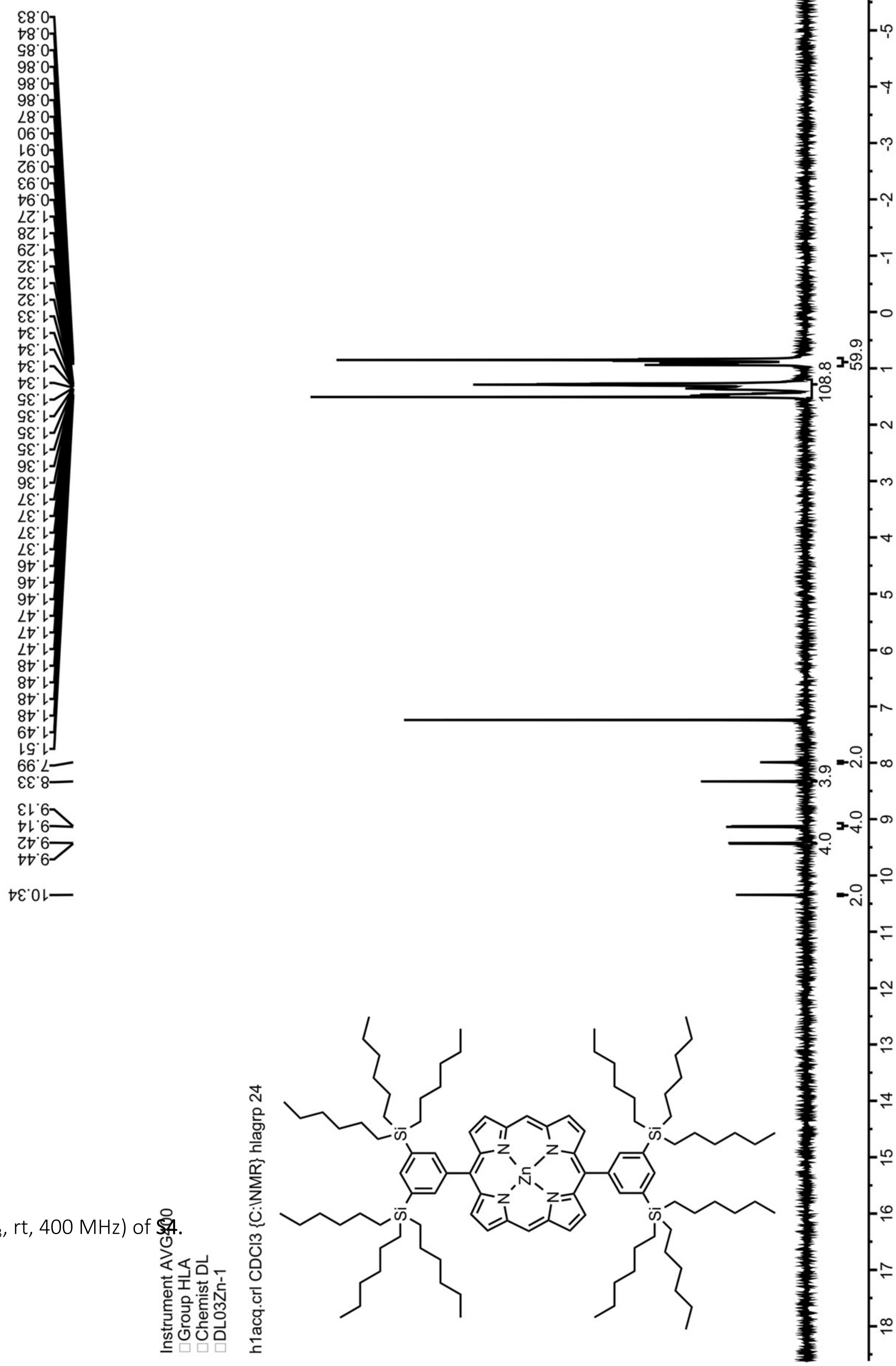

Figure S59: ^{1}H NMR ($CDCl_3$, rt, 400 MHz) of S4.

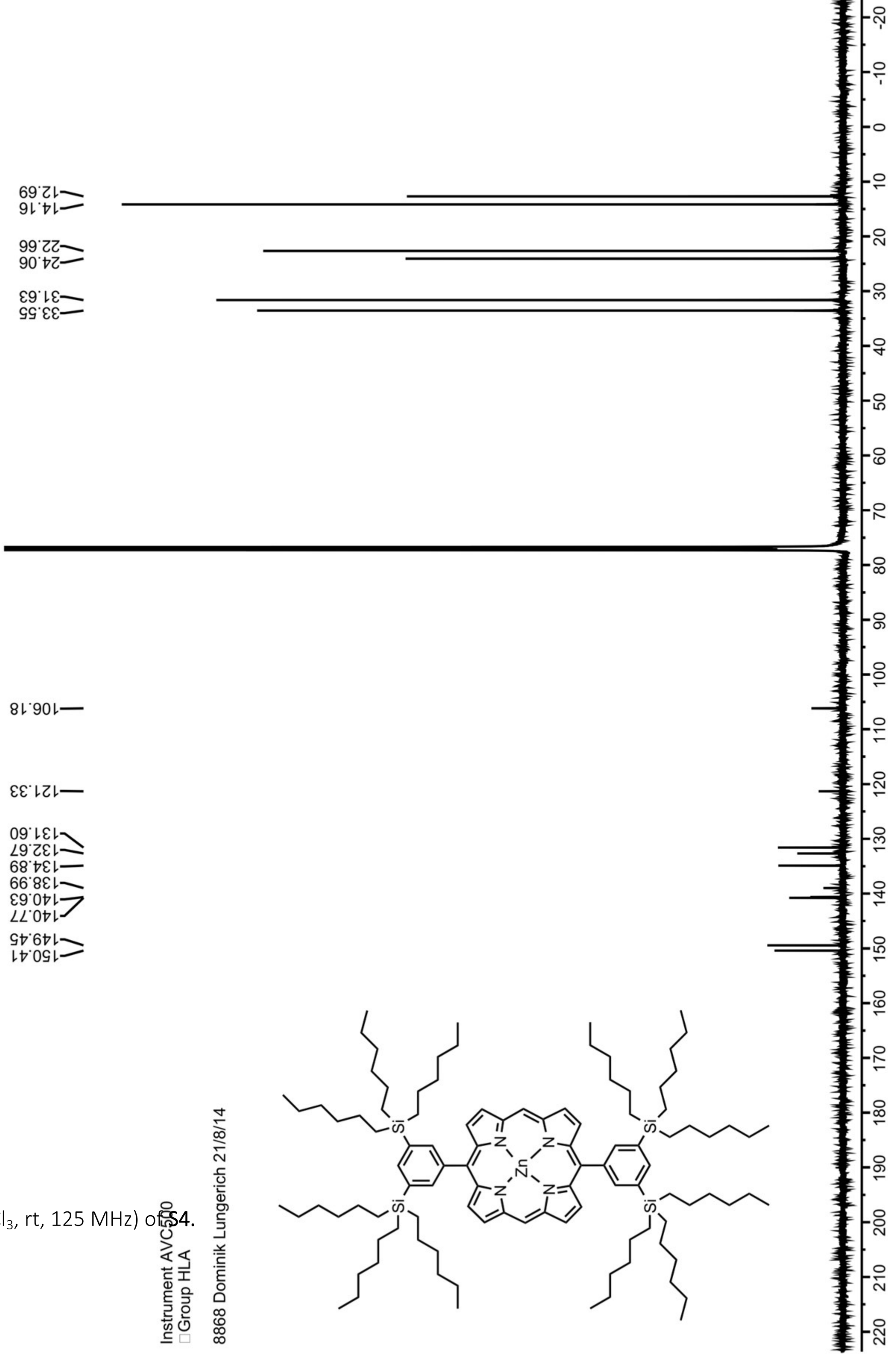

Figure S60: ^{13}C NMR ($CDCl_3$, rt, 125 MHz) of **S4**.

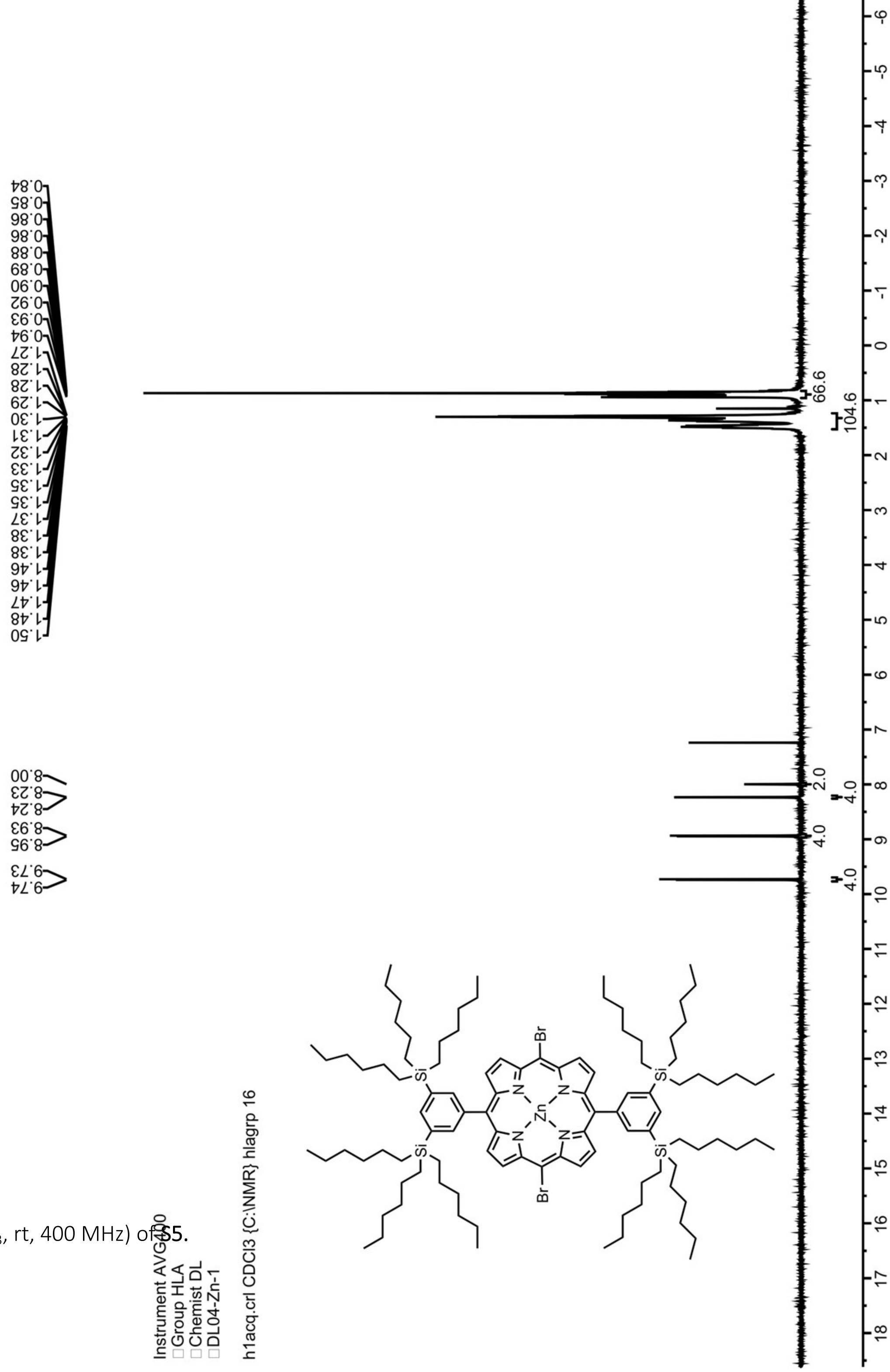

Figure S61: ^{1}H NMR ($CDCl_3$, rt, 400 MHz) of **S5**.

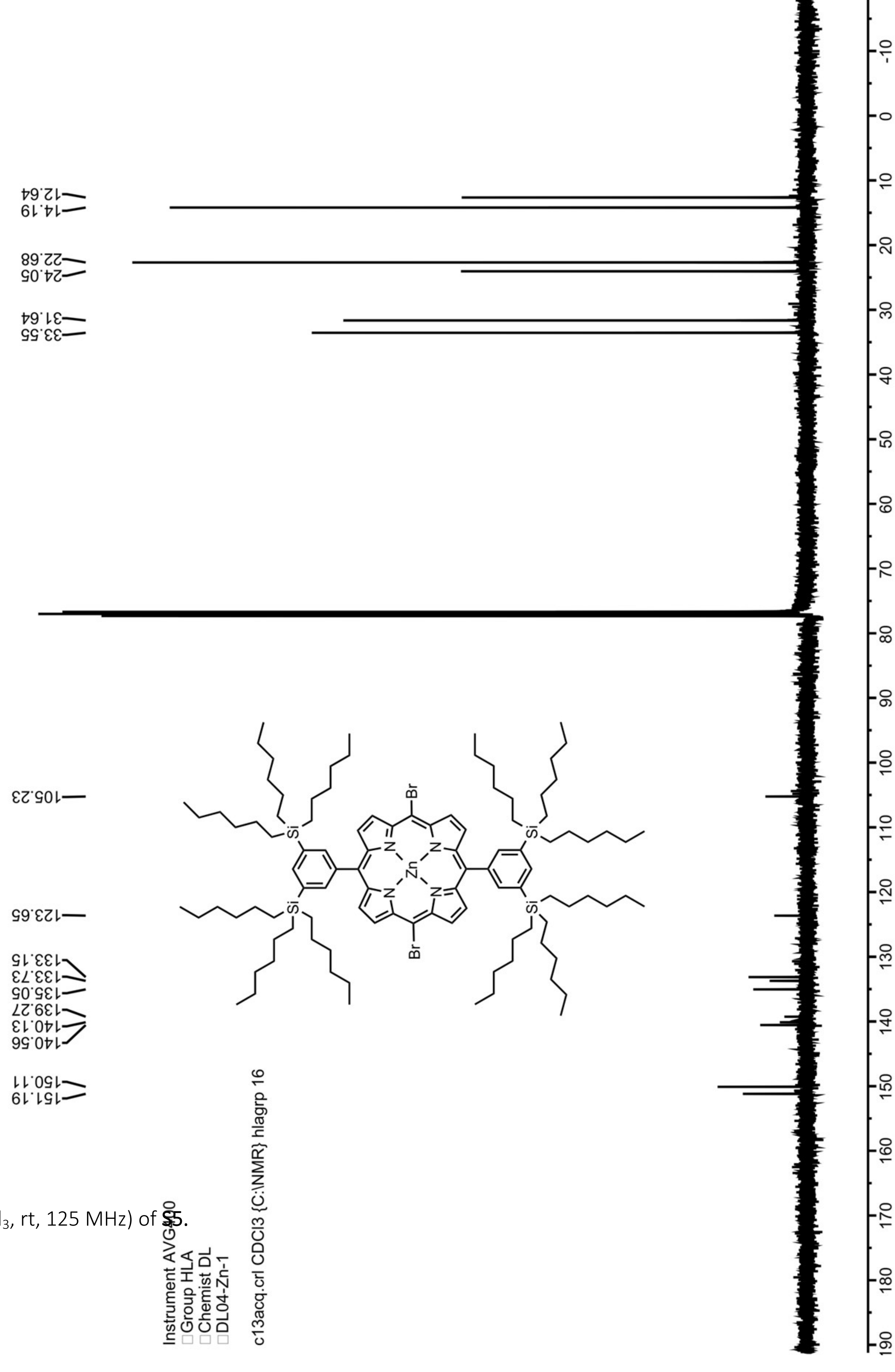

Figure S62: ^{13}C NMR ($CDCl_3$, rt, 125 MHz) of S5.

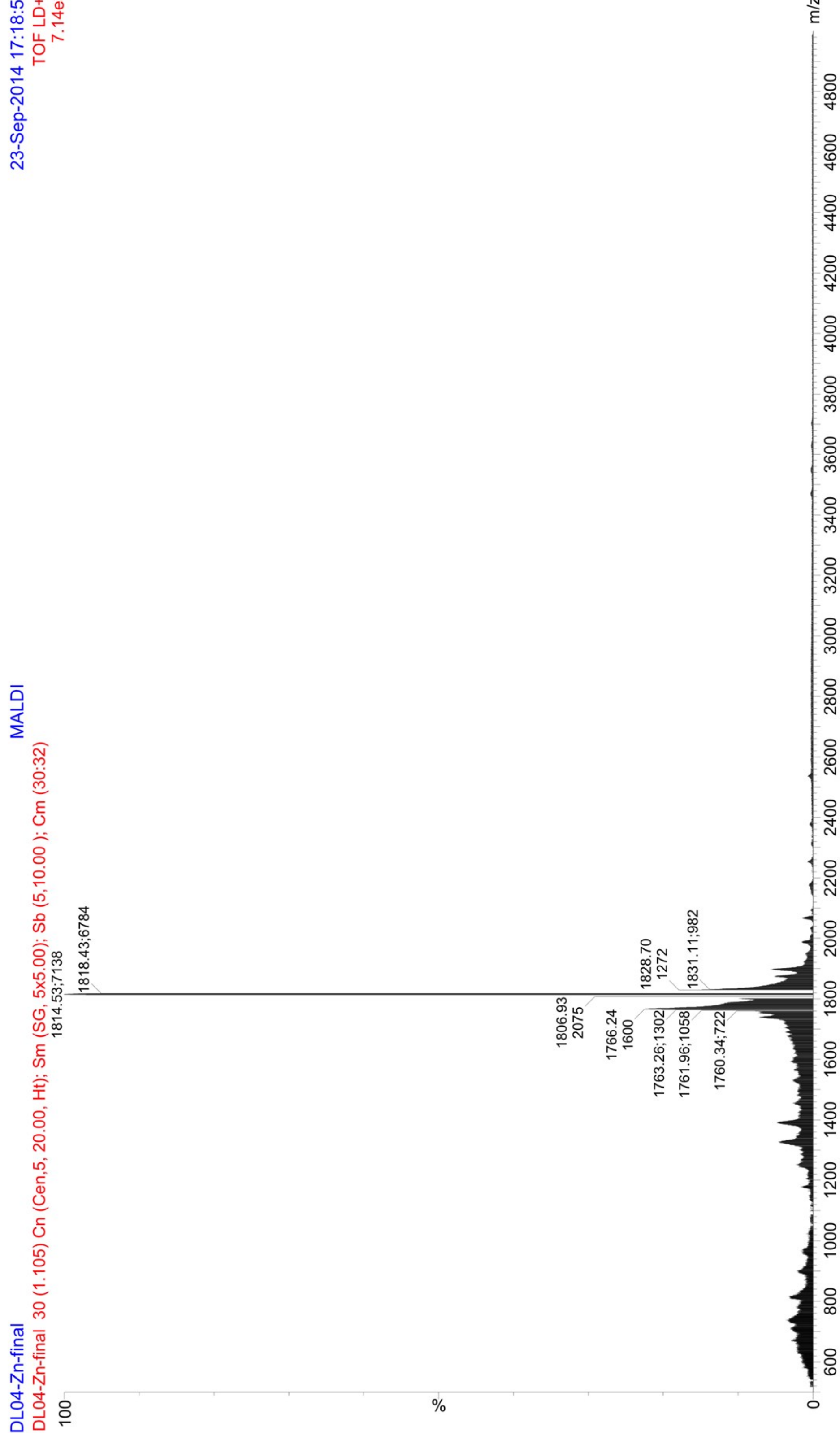

Figure S63: MS (LDI) of **S2**.

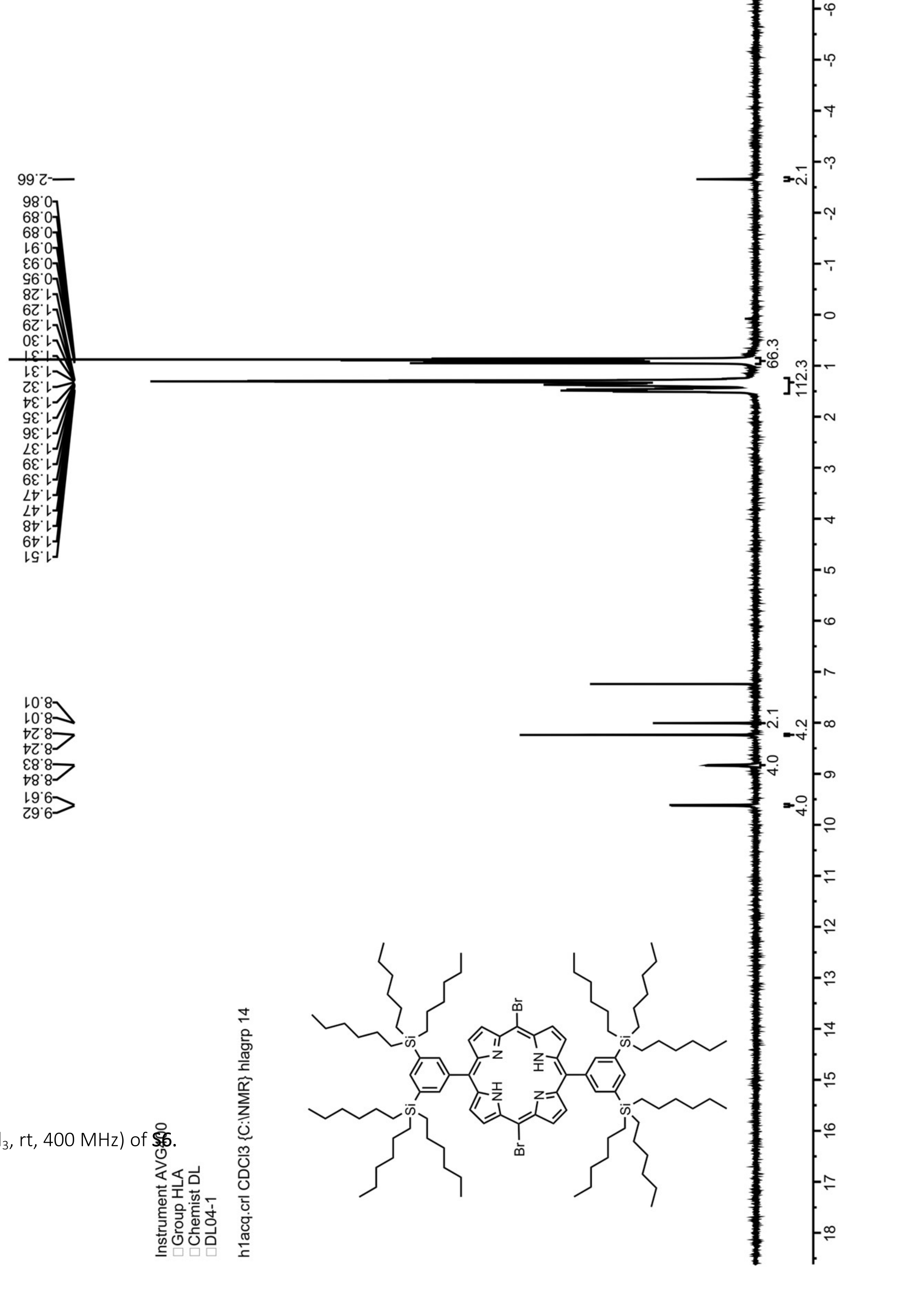

Figure S64: ^{1}H NMR ($CDCl_3$, rt, 400 MHz) of **S6**.

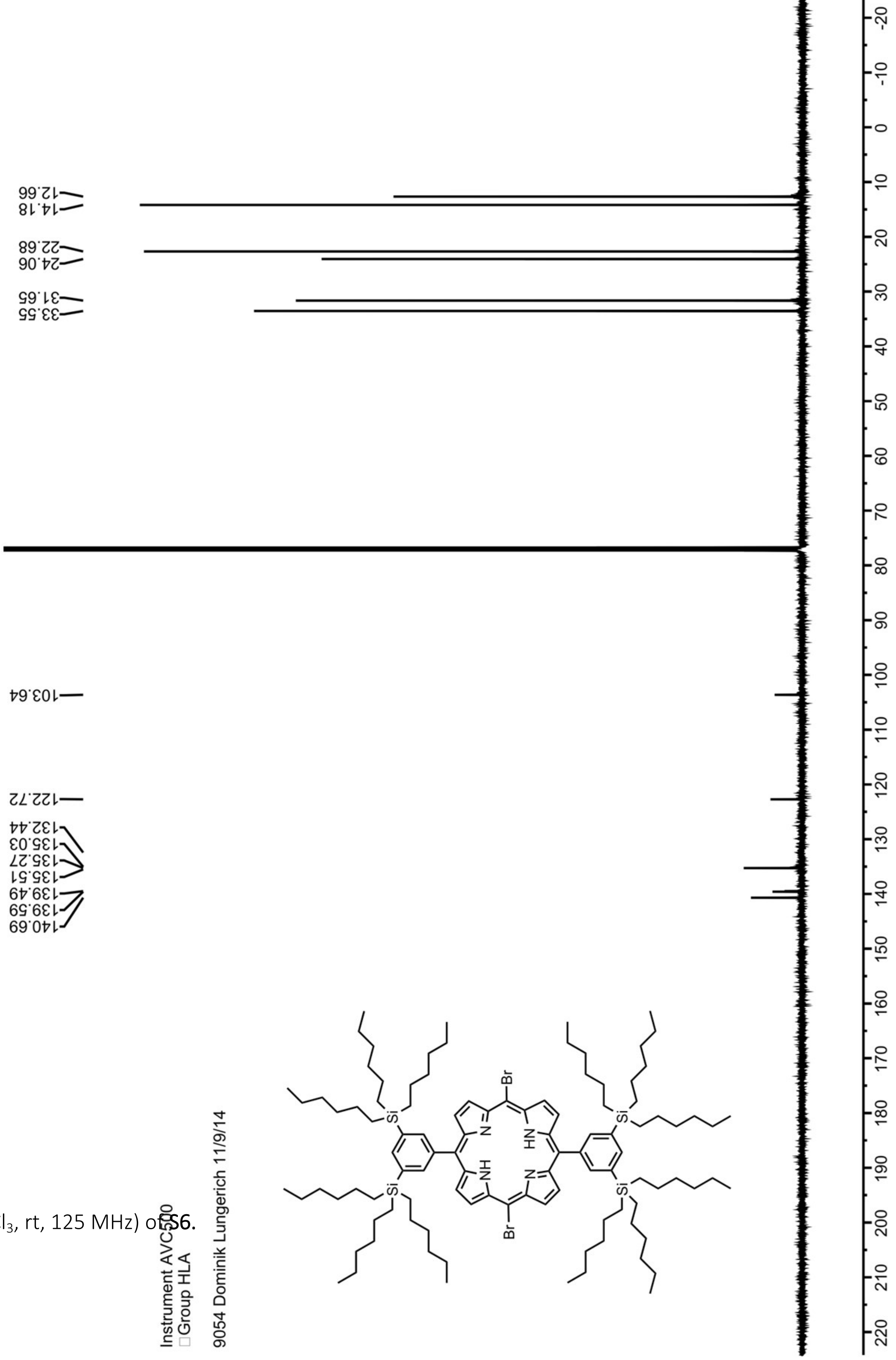

Figure S65: ^{13}C NMR ($CDCl_3$, rt, 125 MHz) of **S6.**

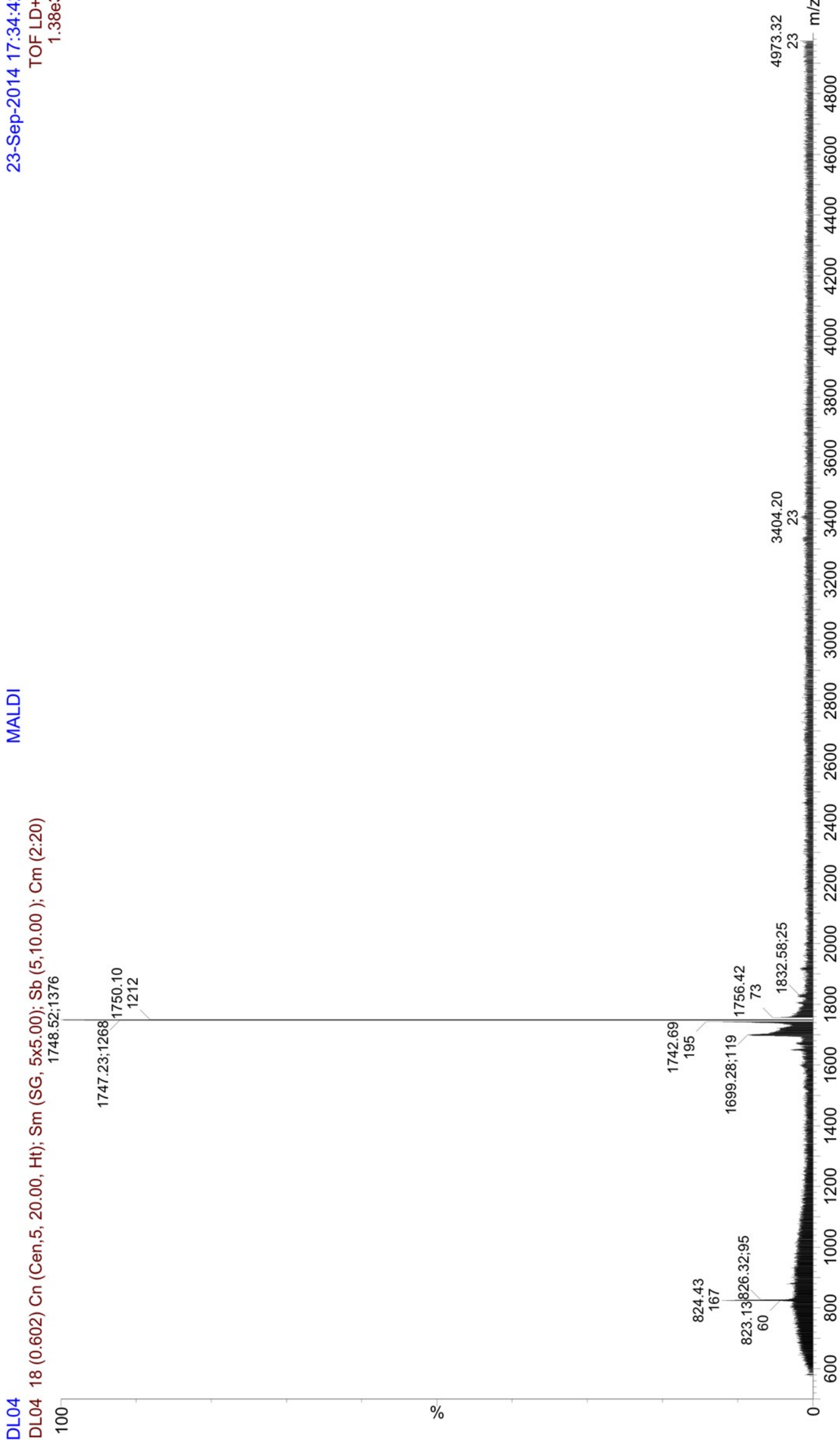

Figure S66: MS (LDI) of **S6.**

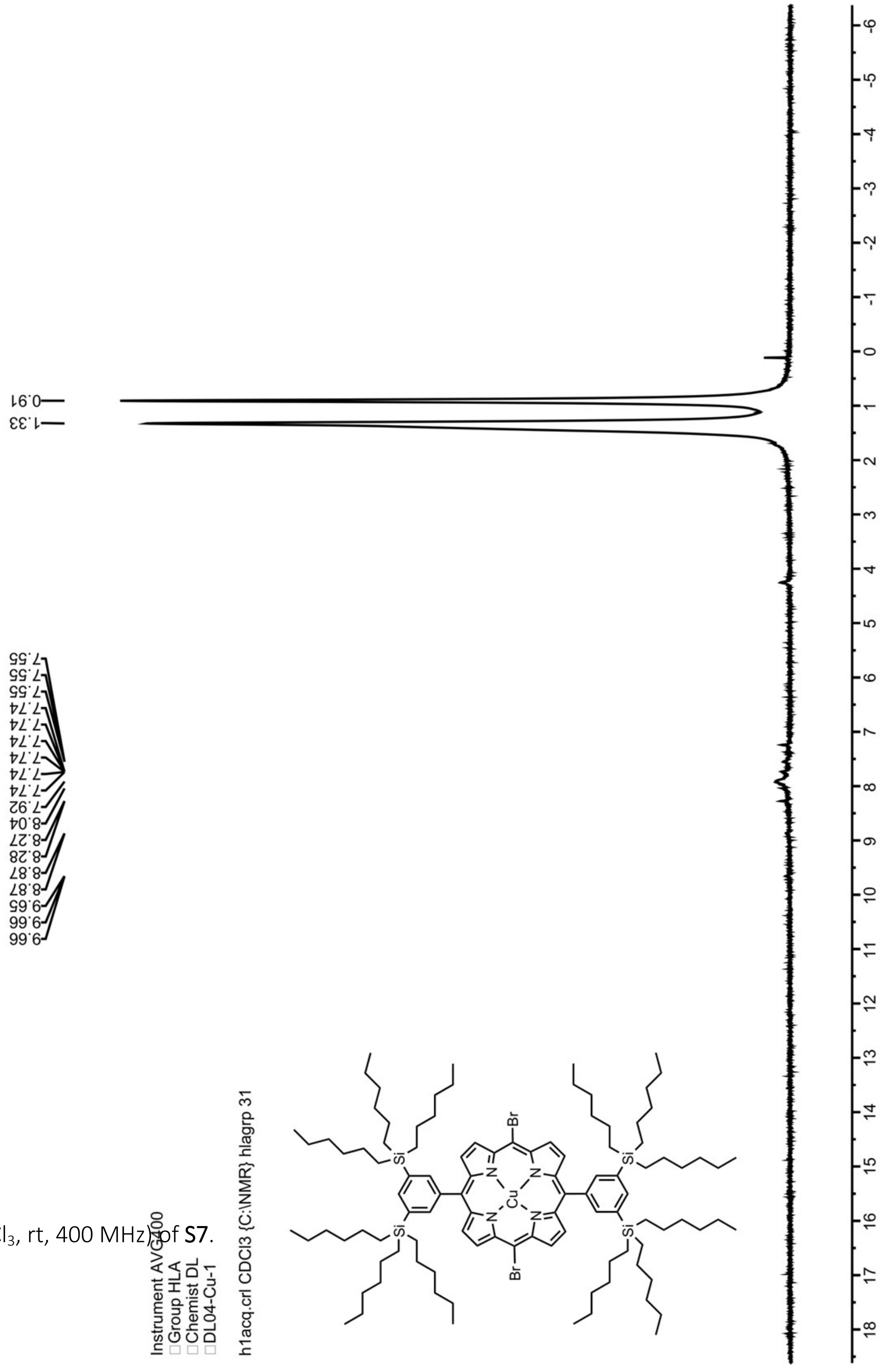

Figure S67:^{1}H NMR ($CDCl_3$, rt, 400 MHz) of **S7**.

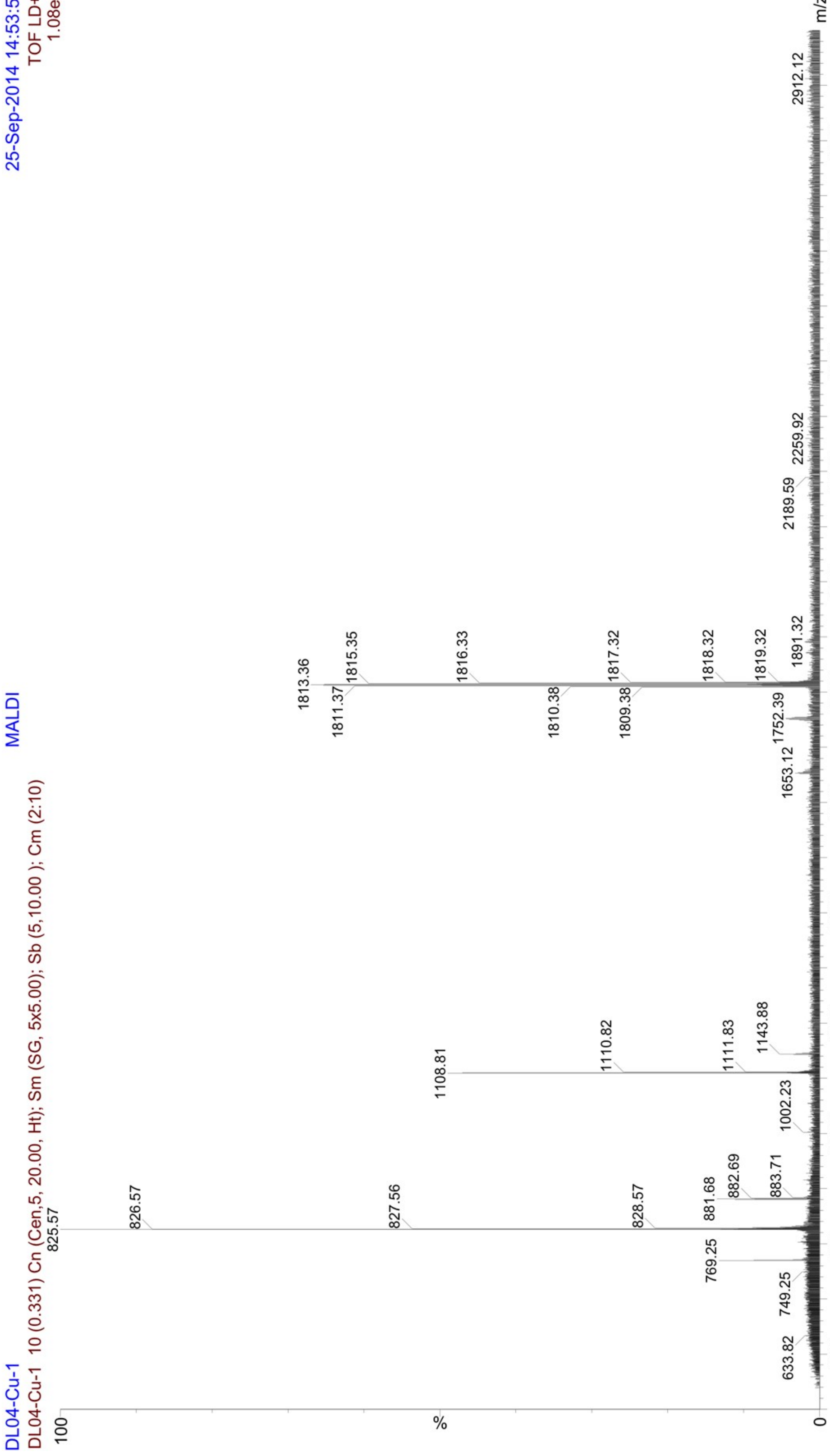

Figure S68: MS(LDI) of **S7**.

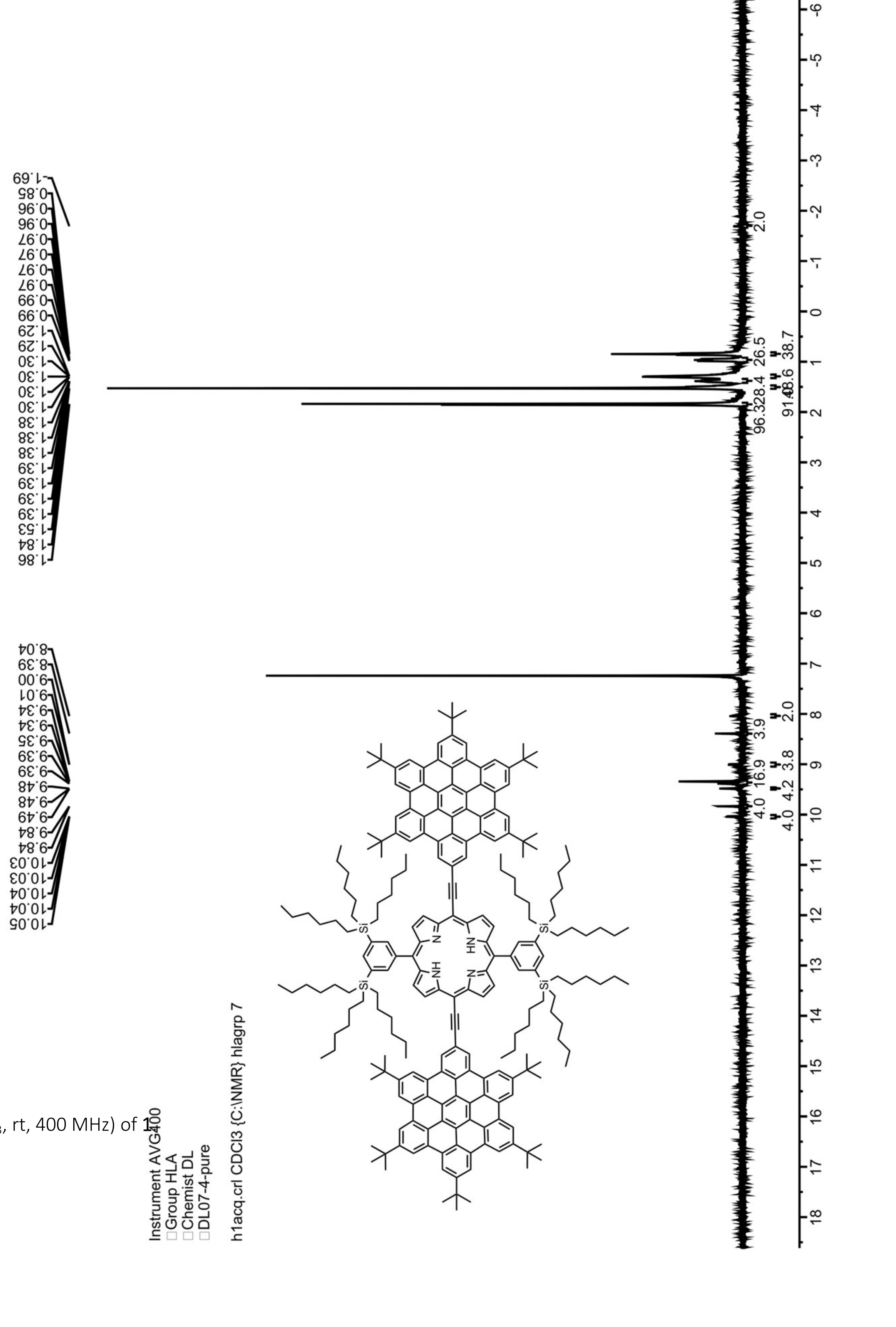

Figure S69: ^{1}H NMR ($CDCl_3$, rt, 400 MHz) of **1**

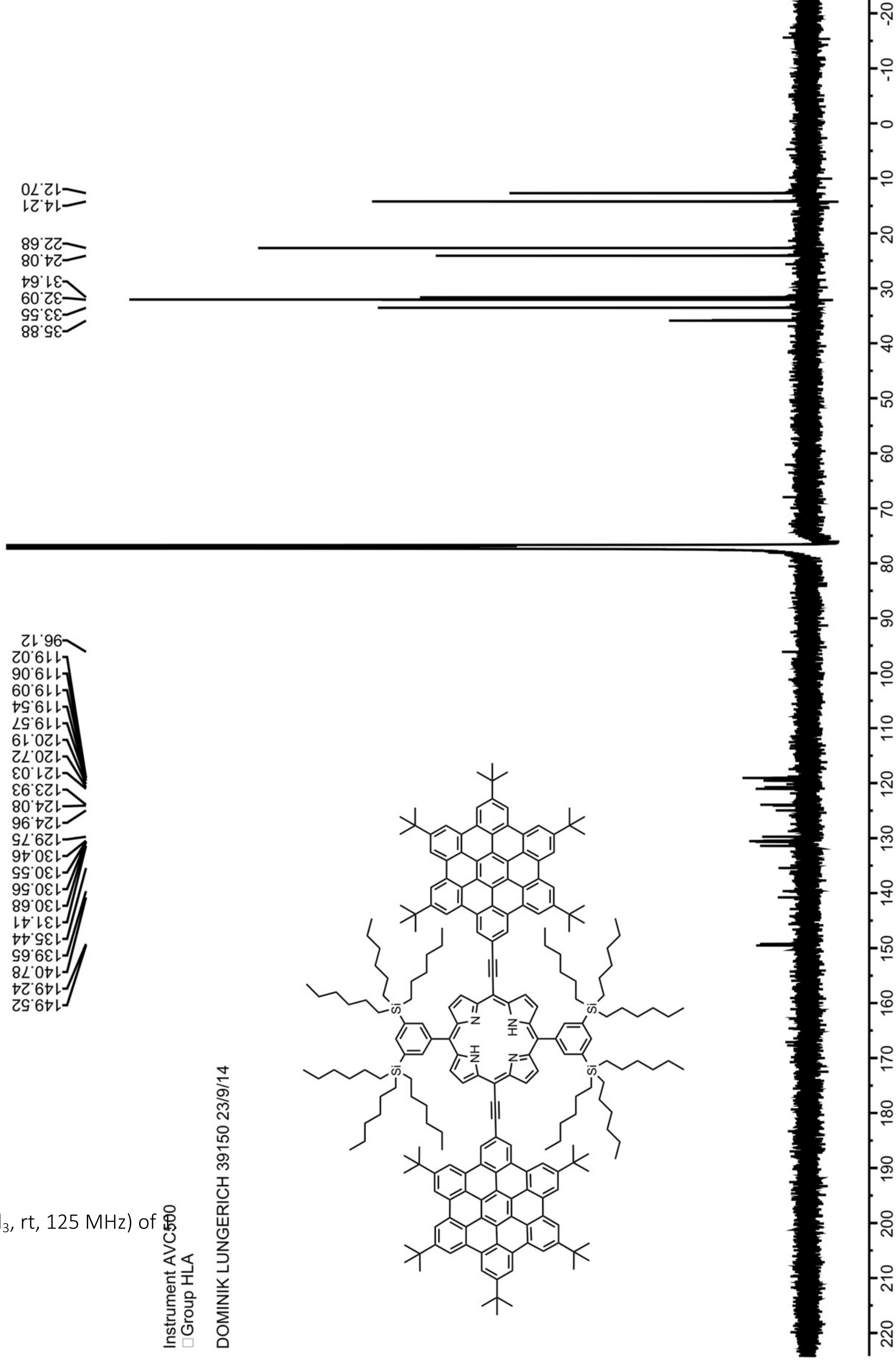

Figure S70: ^{13}C NMR ($CDCl_3$, rt, 125 MHz) of

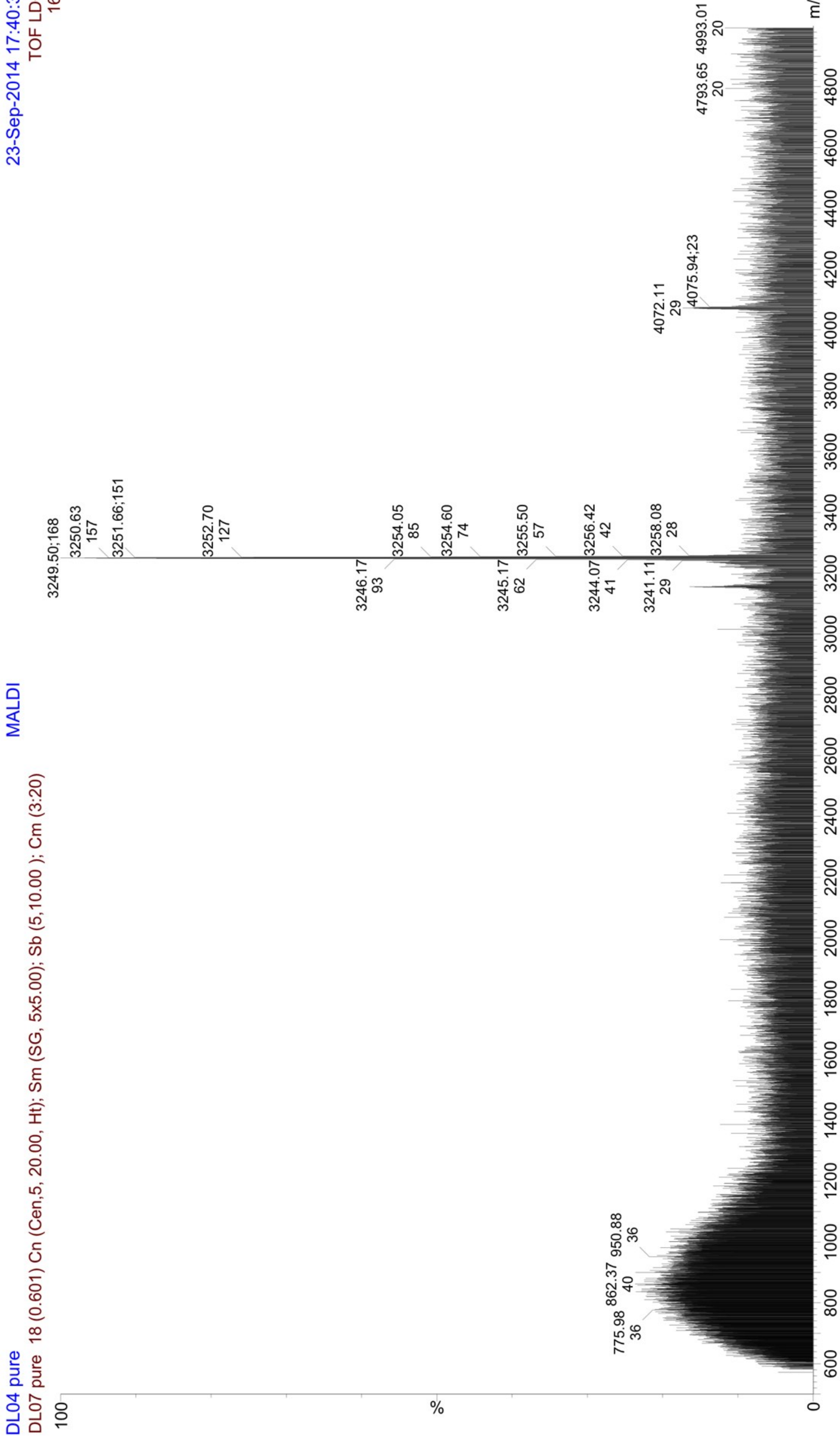

Figure S71: MS (LDI) of **1**.

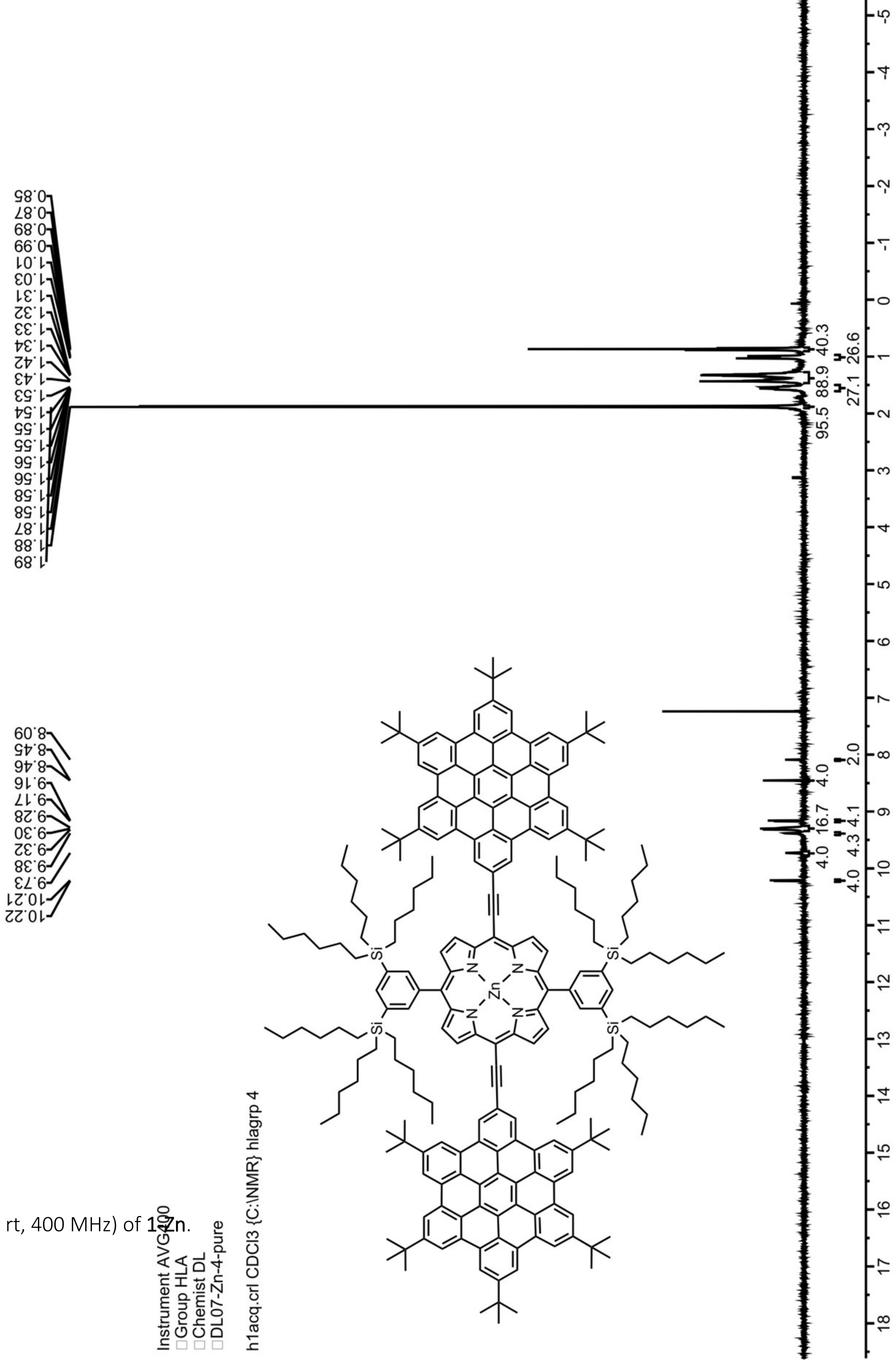

Figure S72: ^{1}H NMR ($CDCl_3$, rt, 400 MHz) of **1-Zn**.

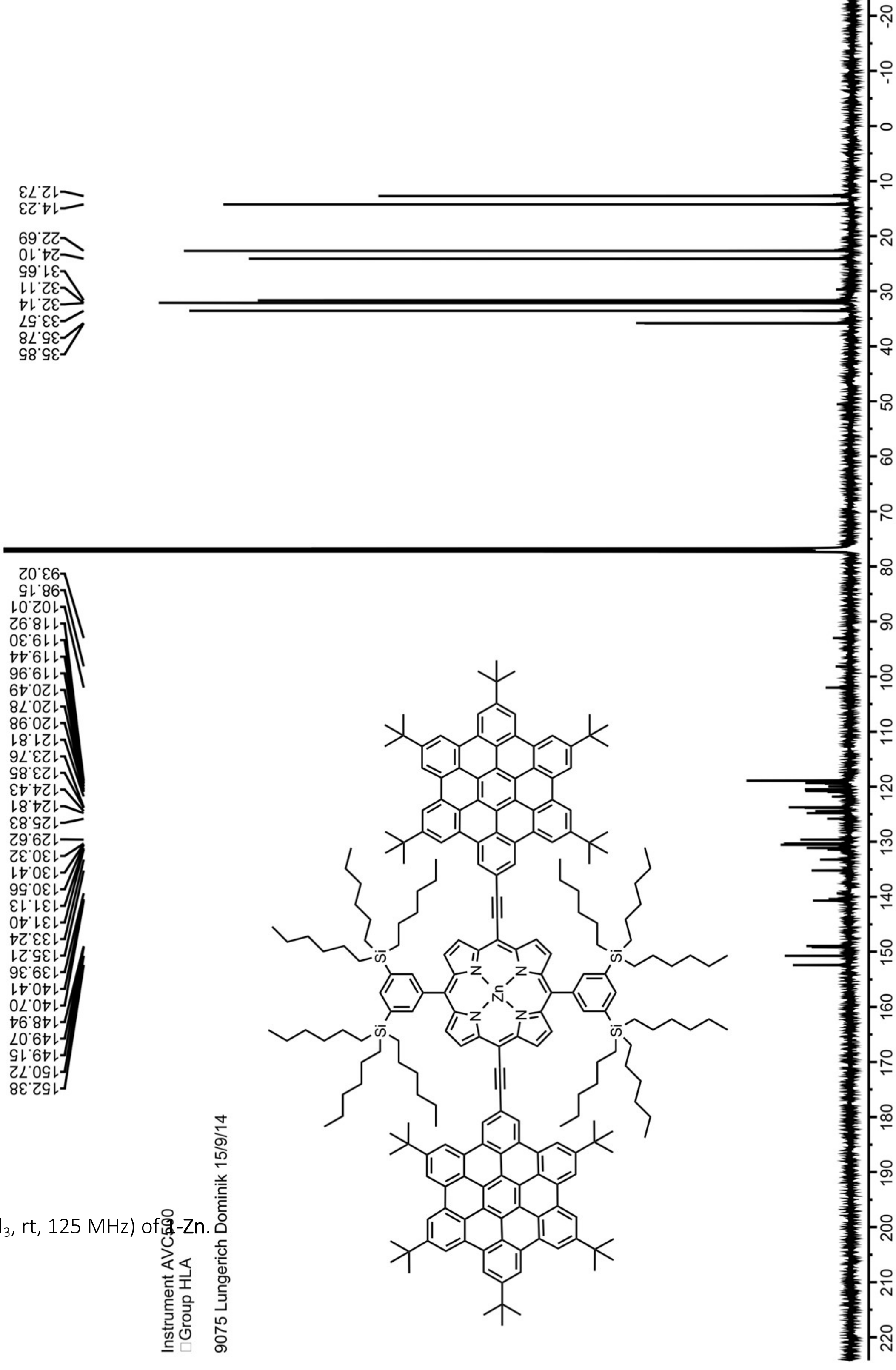

Figure S73: ^{13}C NMR ($CDCl_3$, rt, 125 MHz) of **1-Zn**.

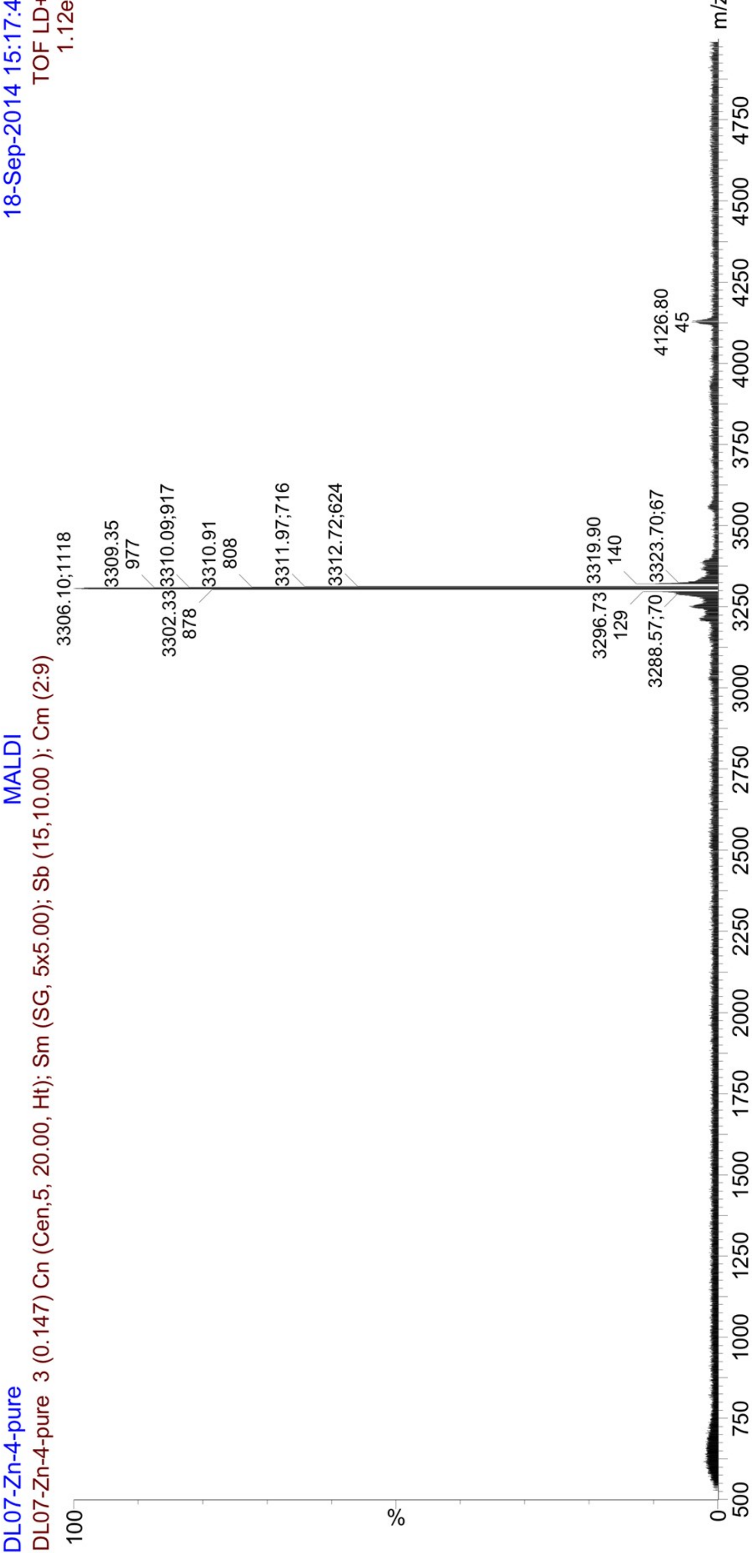

Figure S74: MS (LDI) of **1-Zn**.

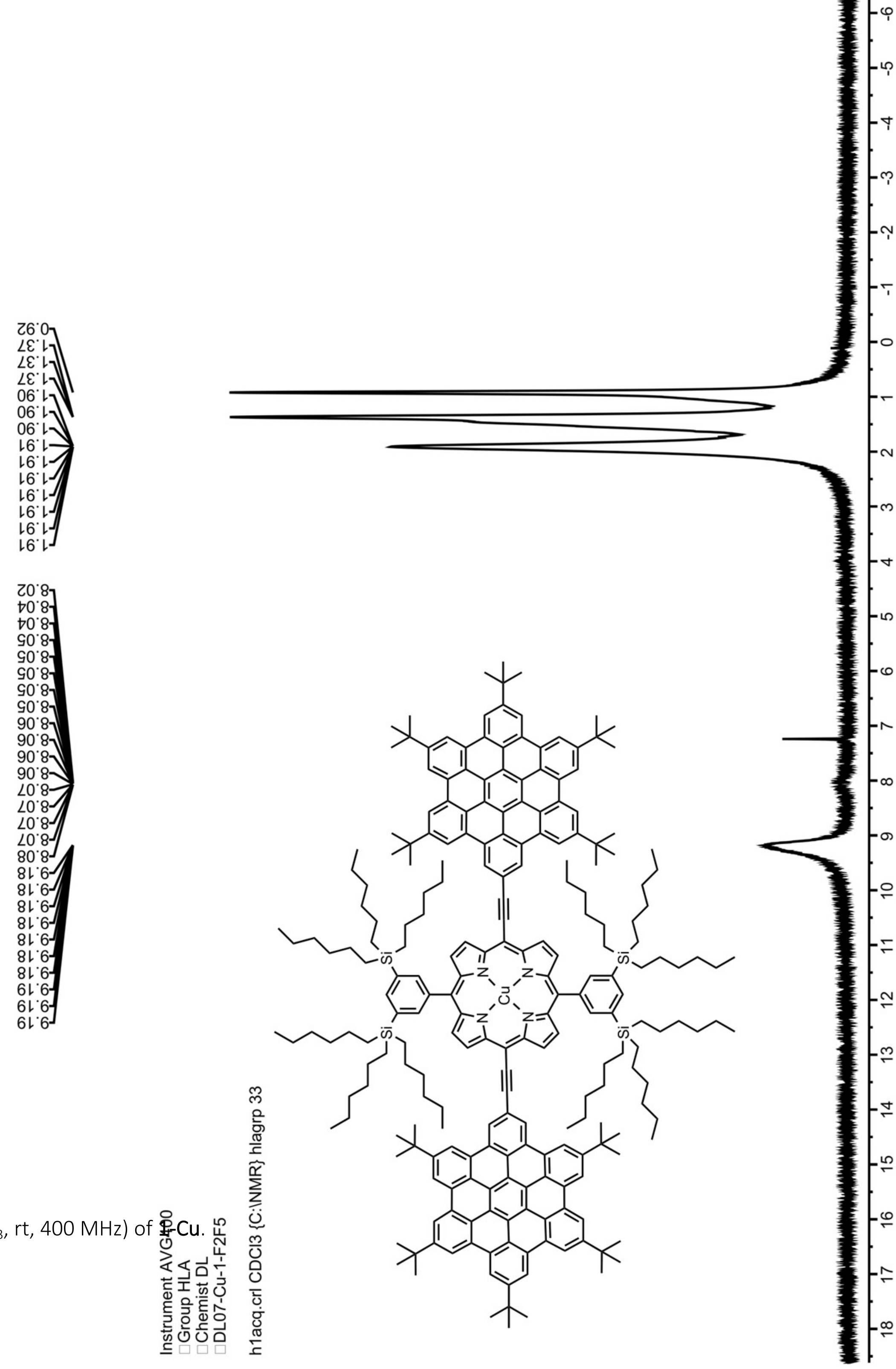

Figure S75: ^{1}H NMR ($CDCl_3$, rt, 400 MHz) of **1-Cu**.

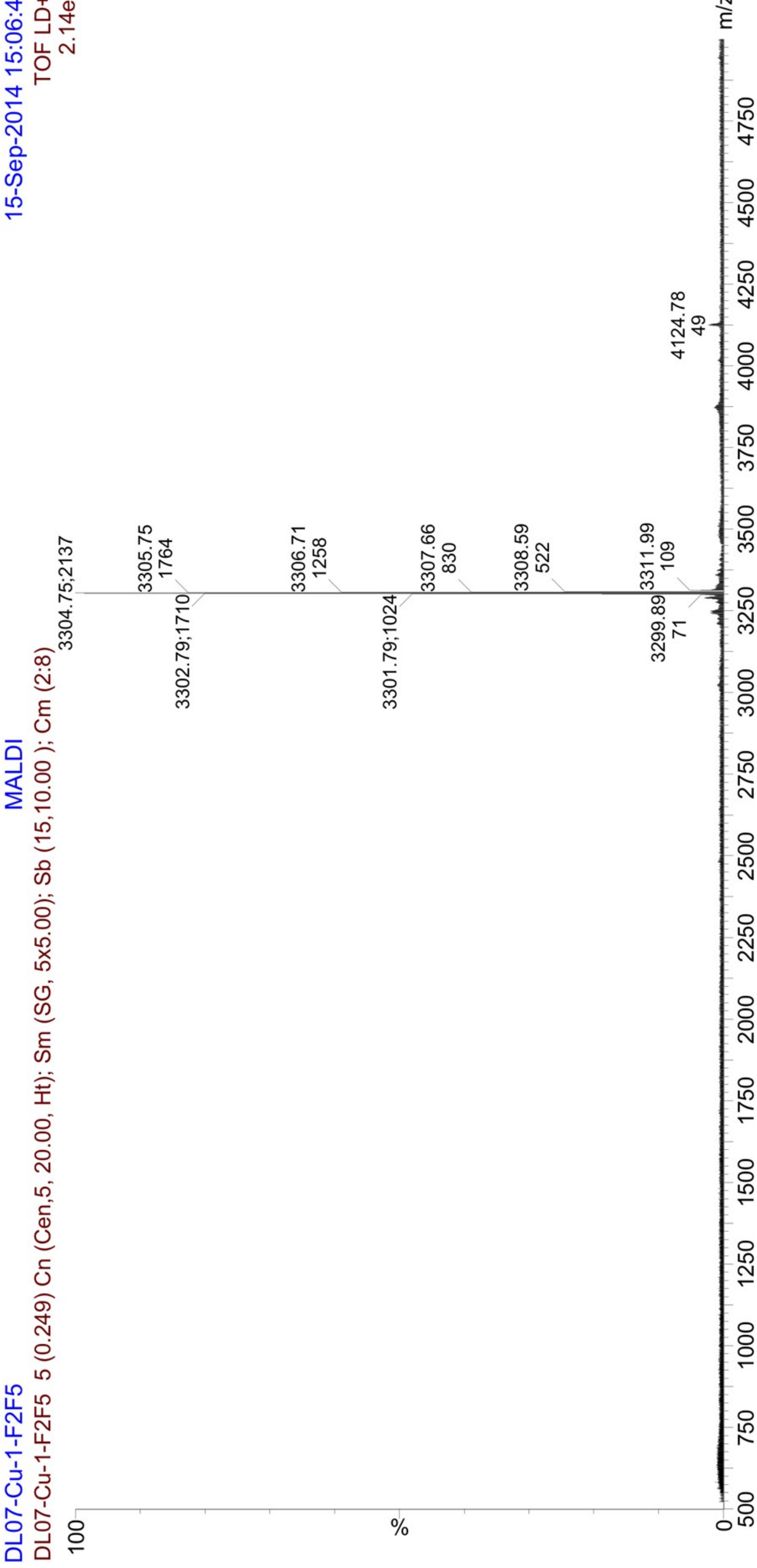

Figure S76: MS (LDI) of **1-Cu**.

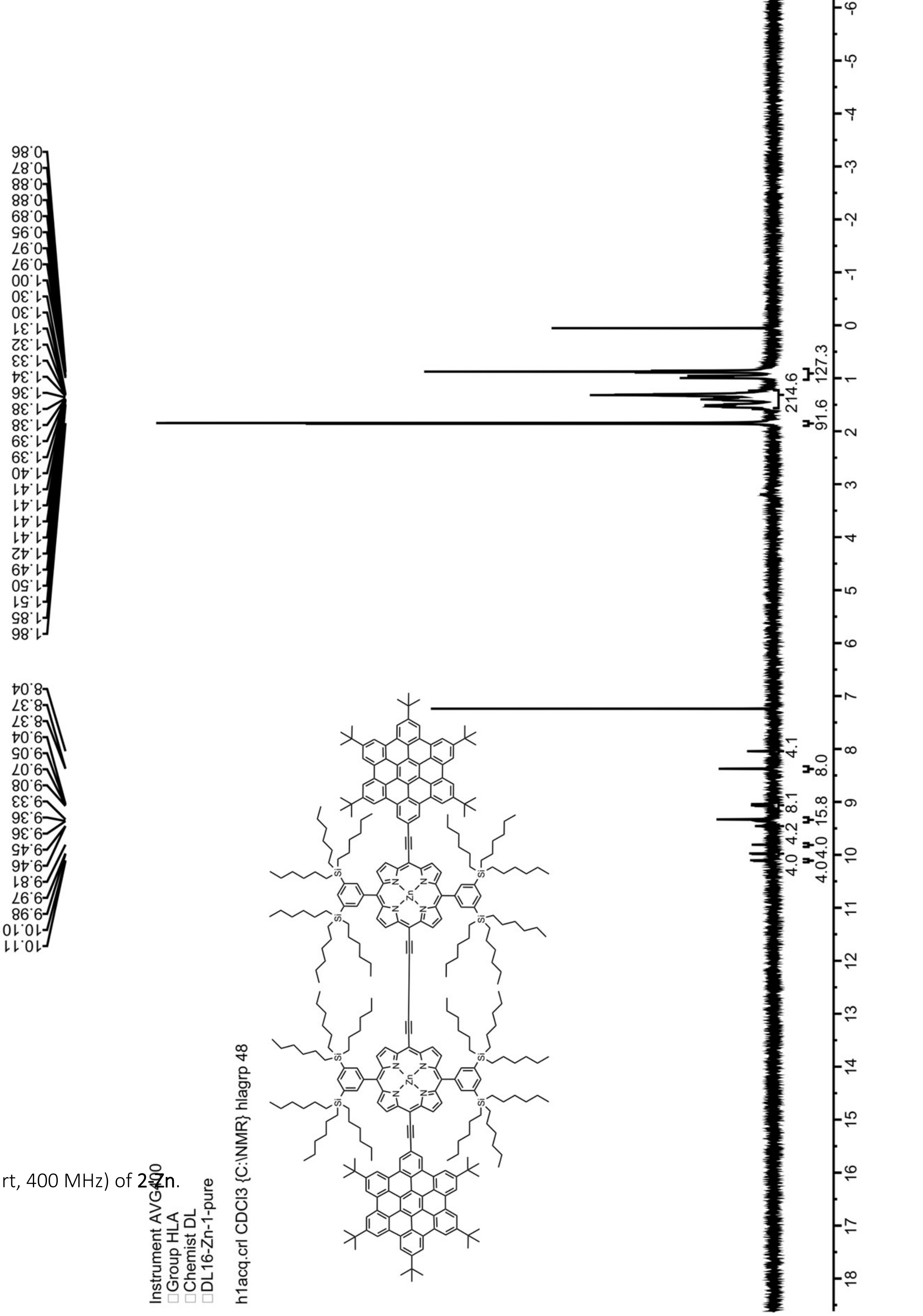

Figure S77: ^{1}H NMR ($CDCl_3$, rt, 400 MHz) of **2-Zn**.

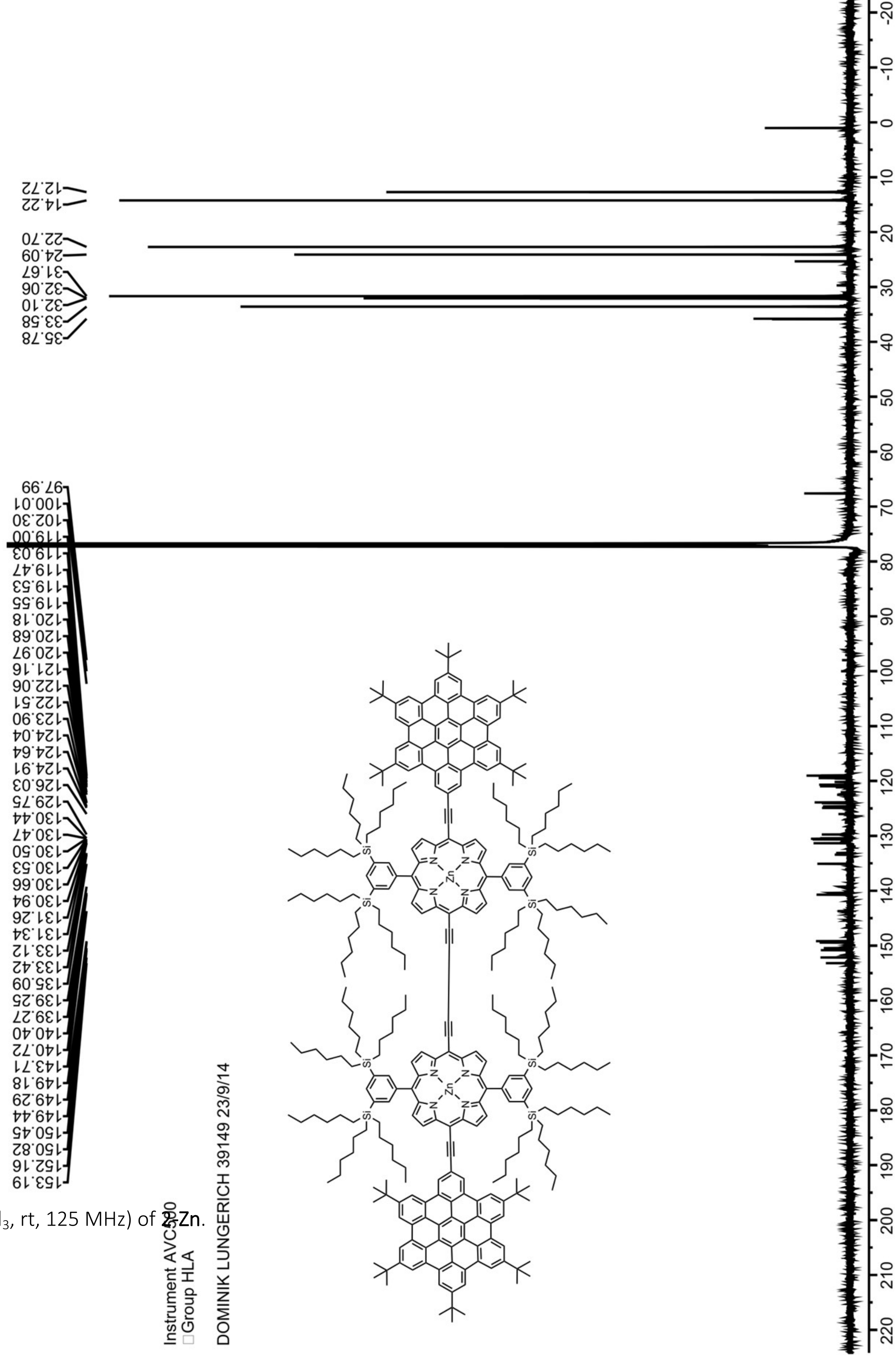

Figure S78: ^{13}C NMR ($CDCl_3$, rt, 125 MHz) of **2-Zn**.

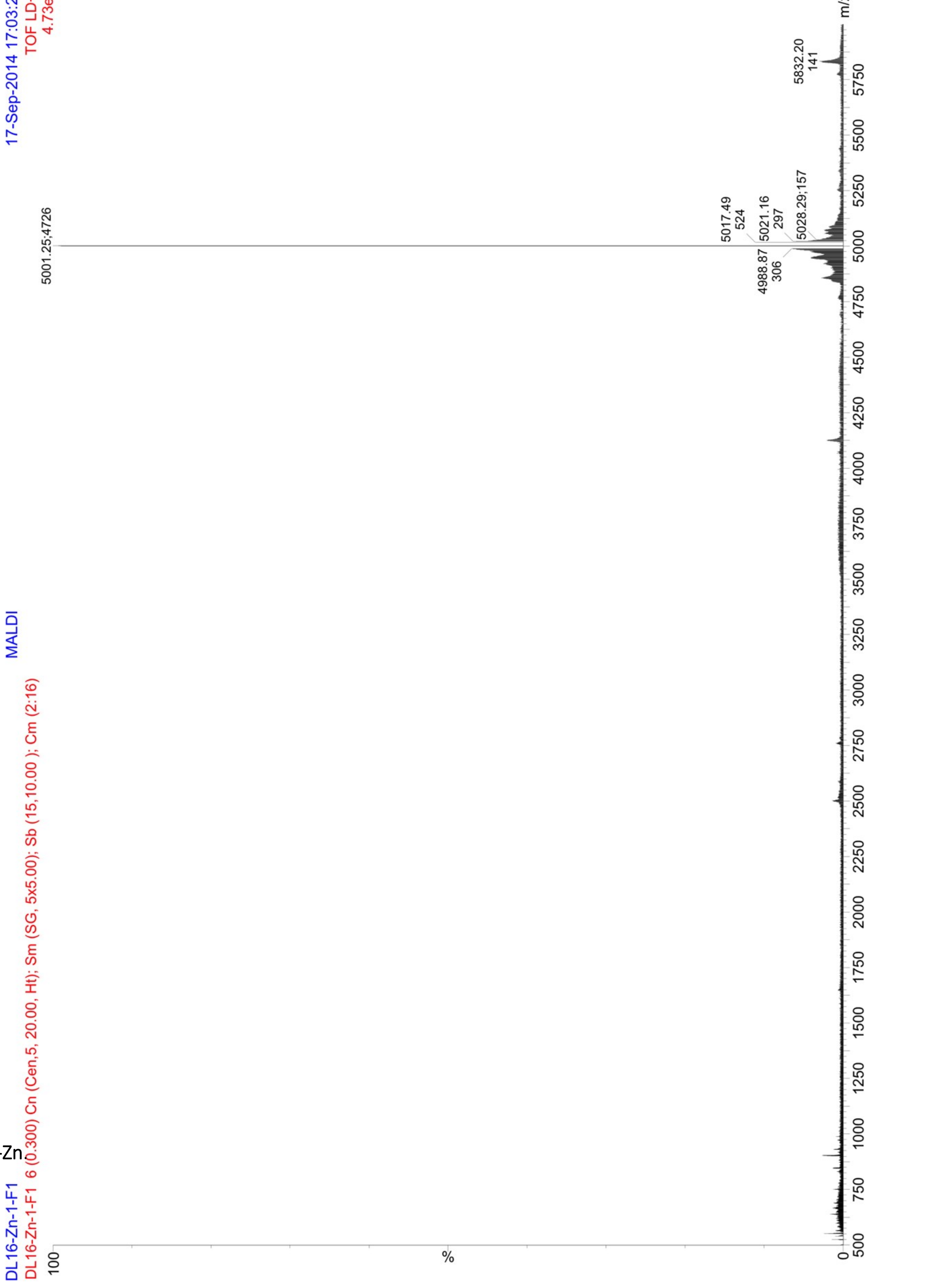

Figure S79: MS (LDI) of **2-Zn**

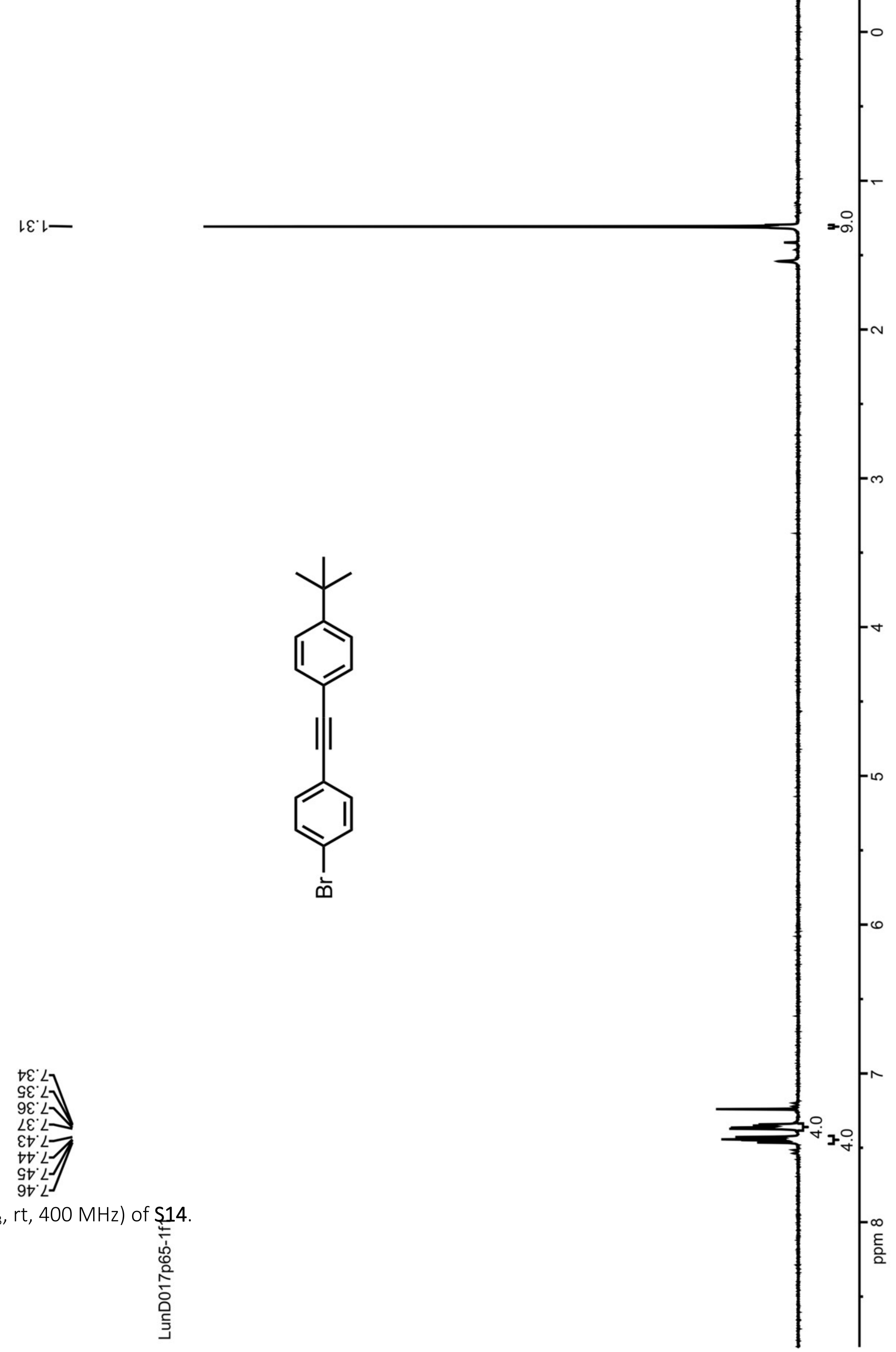

Figure S80: ^{1}H NMR ($CDCl_3$, rt, 400 MHz) of **S14**.

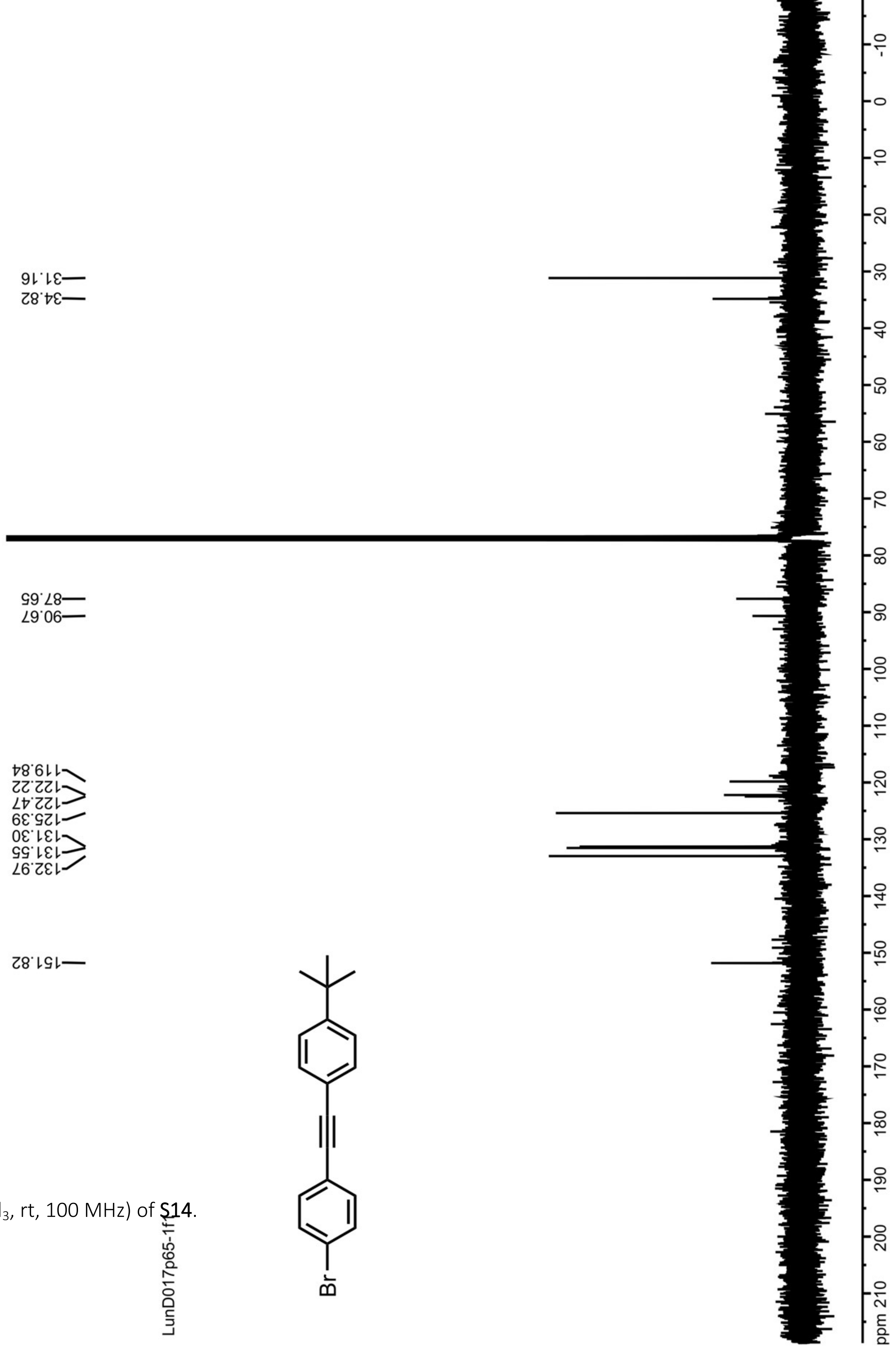

Figure S81: ^{13}C NMR ($CDCl_3$, rt, 100 MHz) of S14.

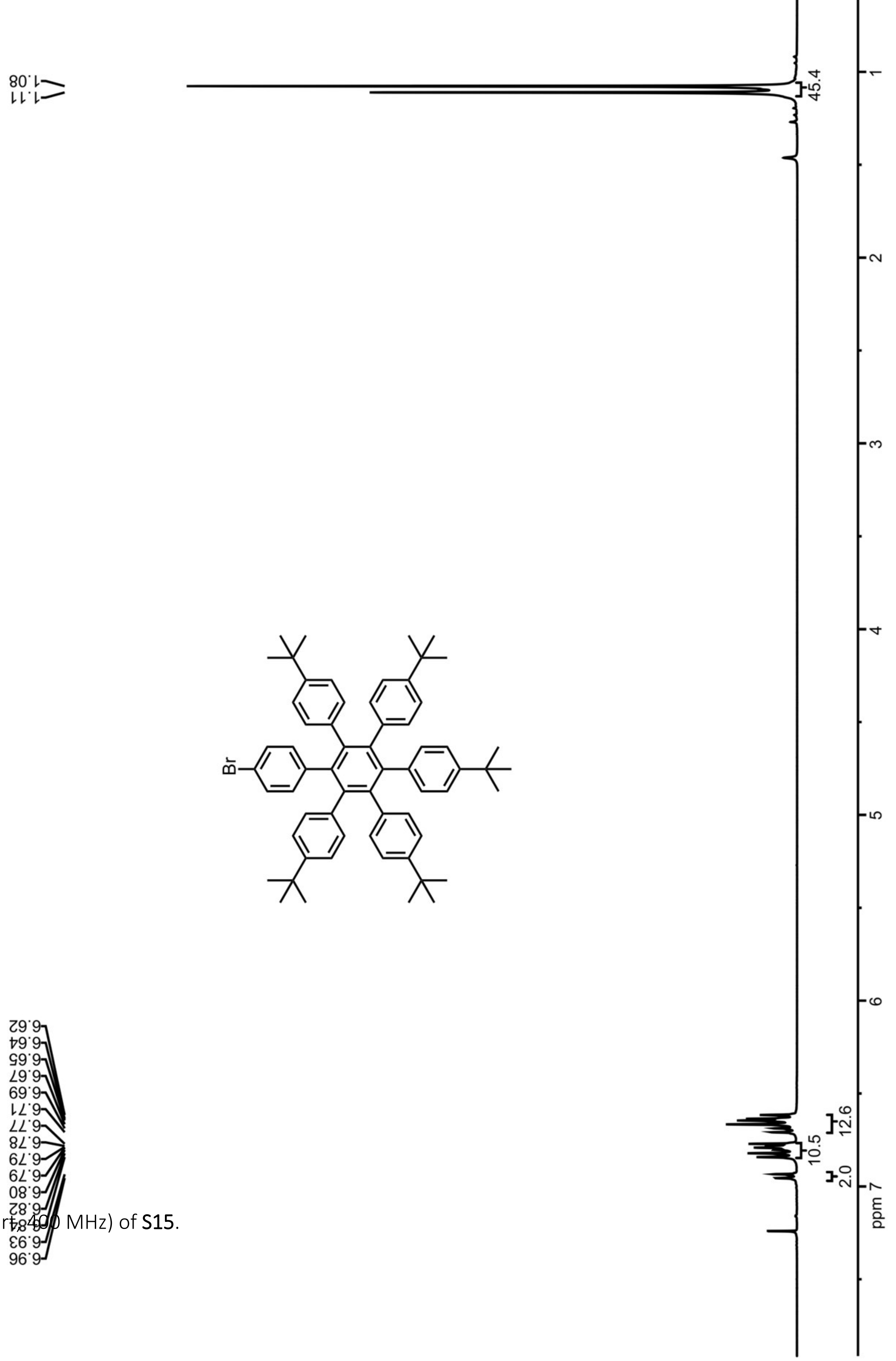

Figure S82: ^{1}H NMR ($CDCl_3$, rt, 400 MHz) of **S15**.

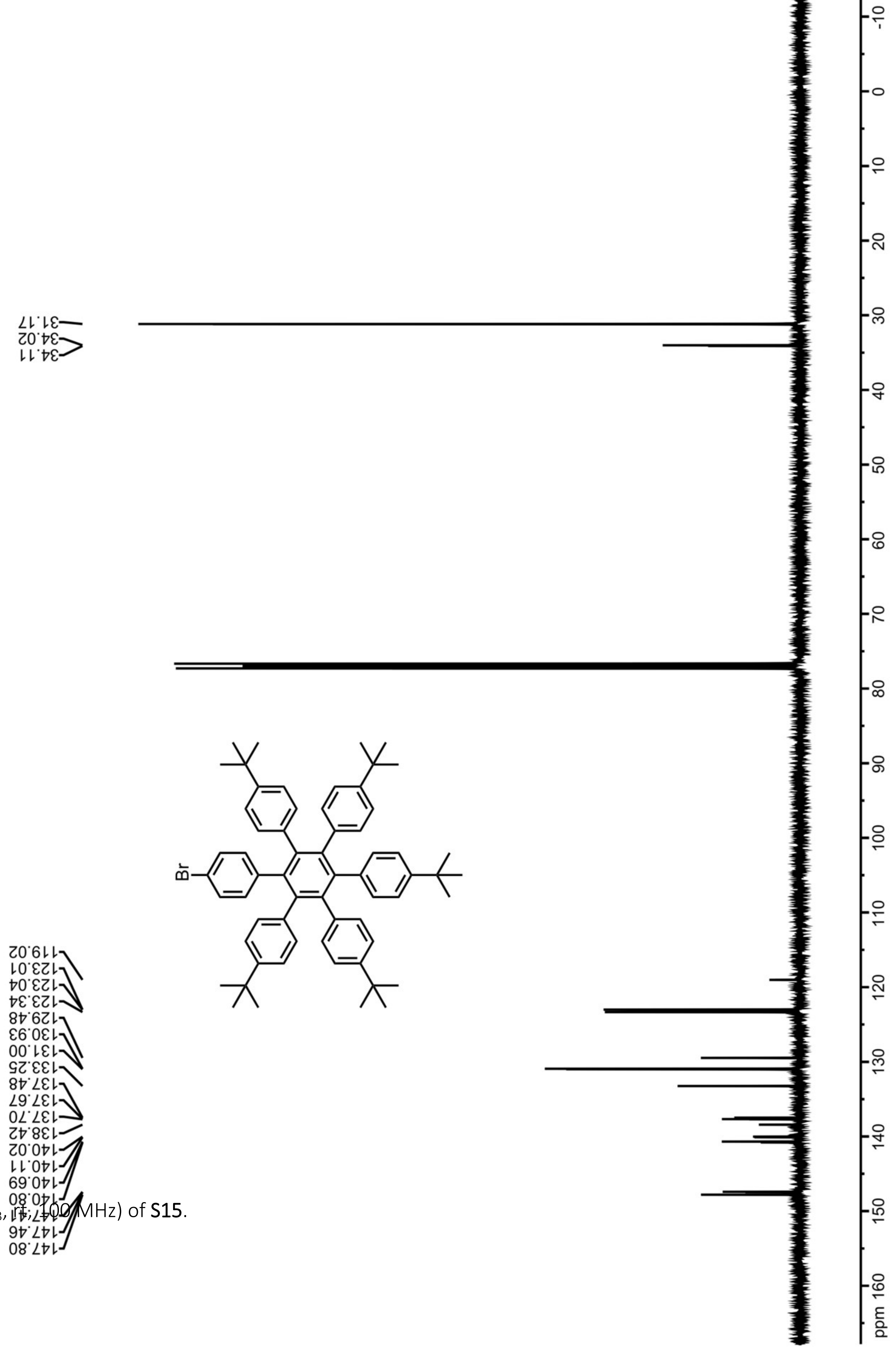

Figure S83: ^{13}C NMR ($CDCl_3$, rt, 100 MHz) of **S15**.

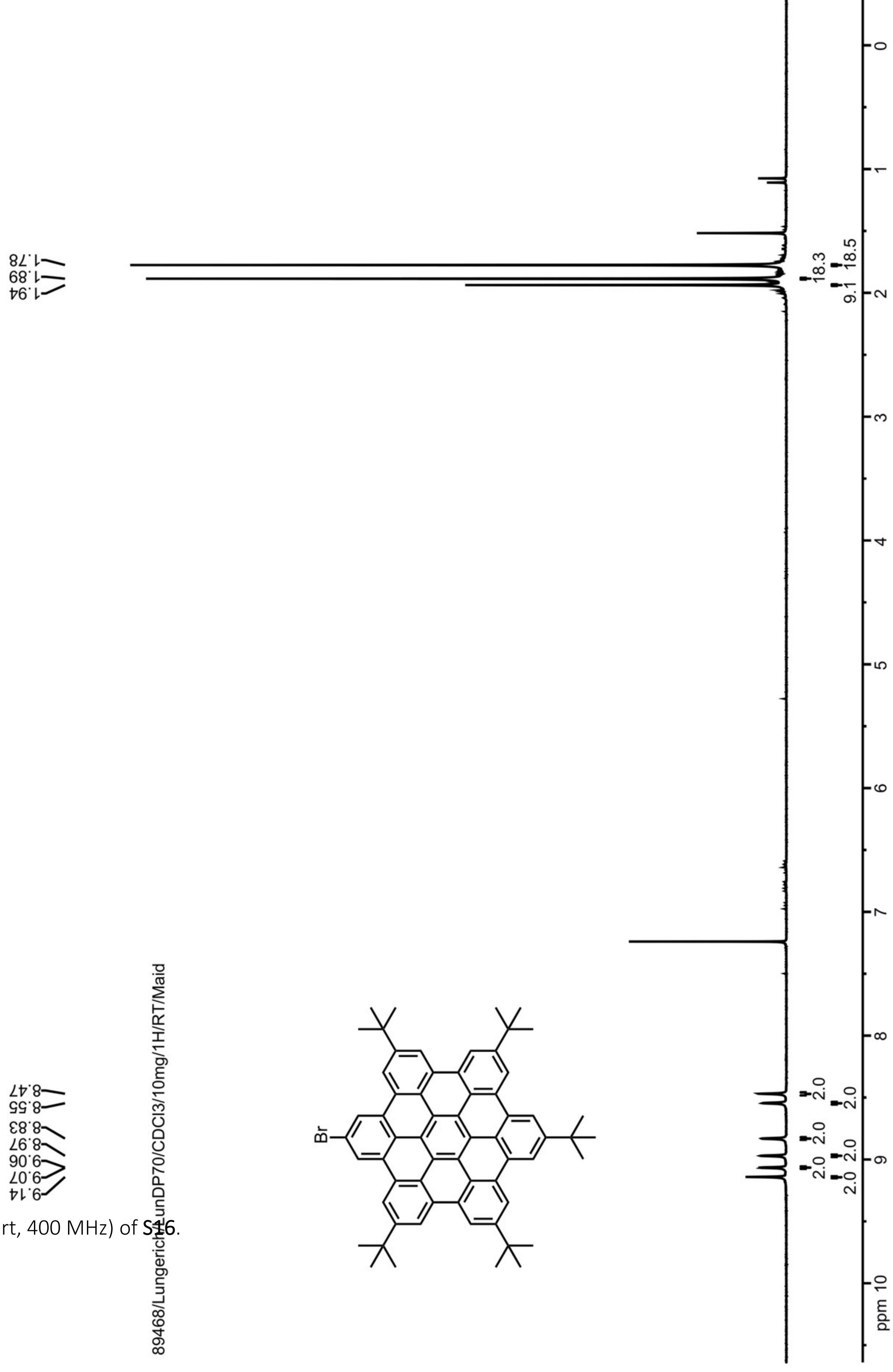

Figure S84: ^{1}H NMR ($CDCl_3$, rt, 400 MHz) of S16.

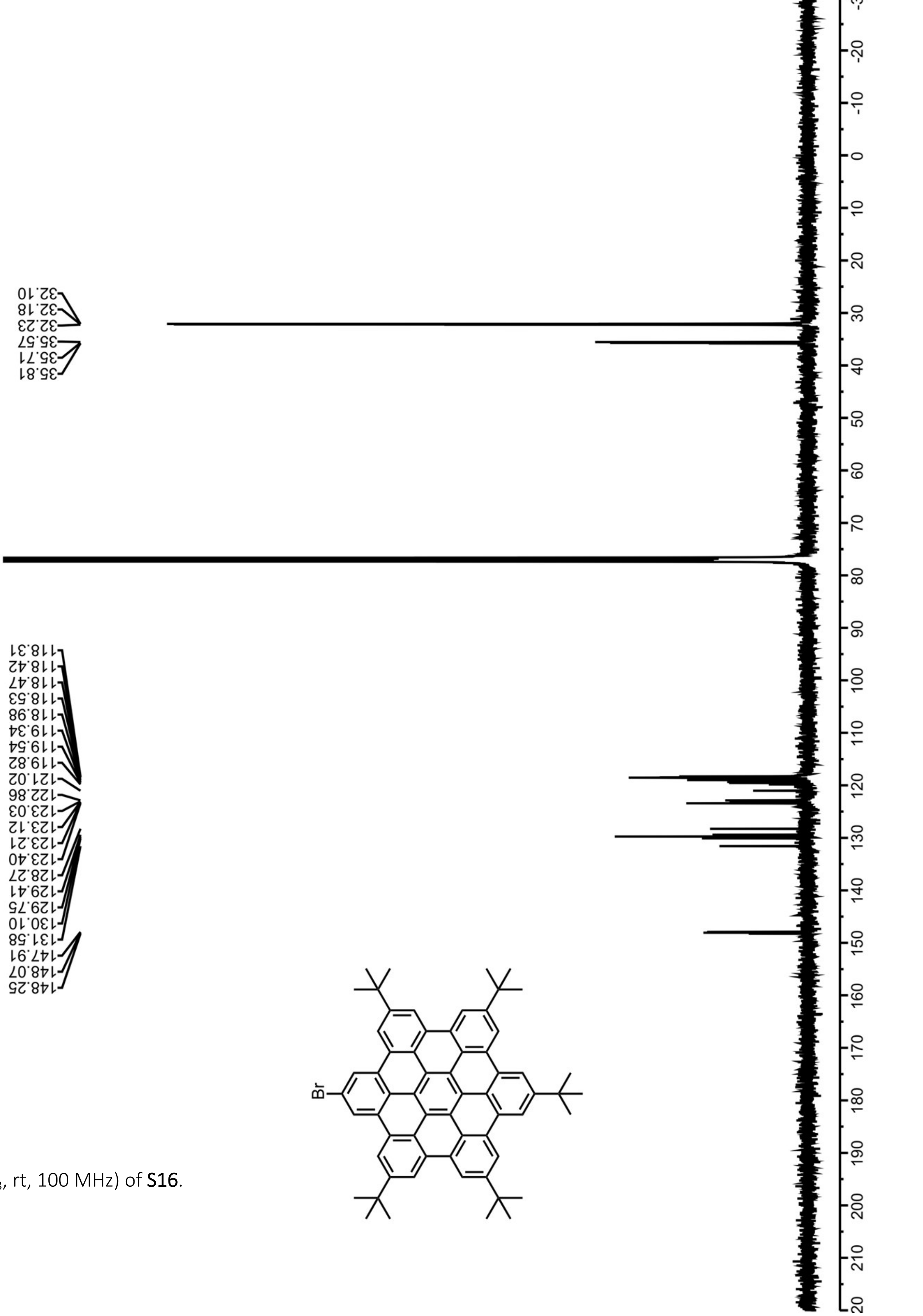

Figure S85: ^{13}C NMR ($CDCl_3$, rt, 100 MHz) of **S16**.

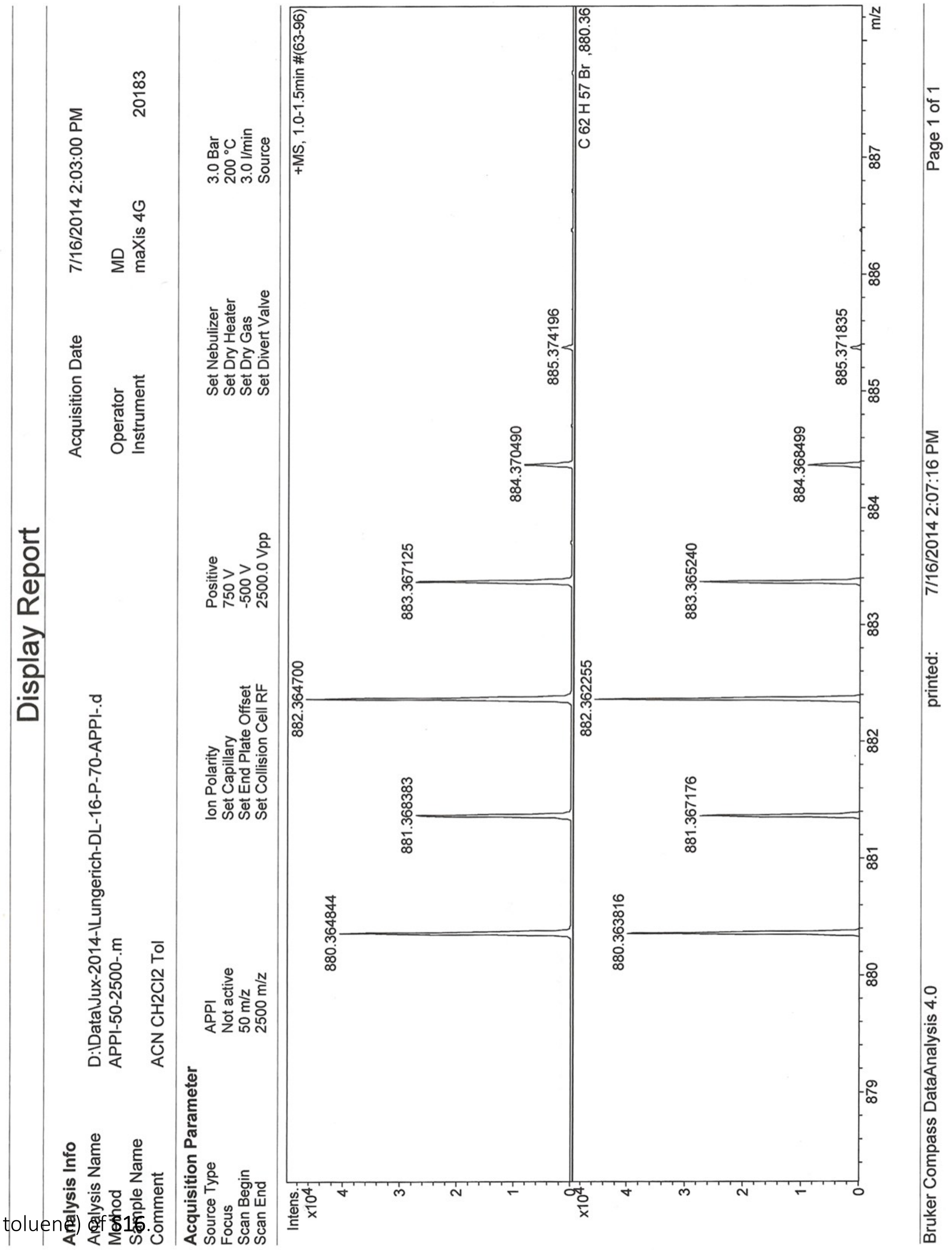

Figure S86: HRMS (APPI, toluene) of **116**.

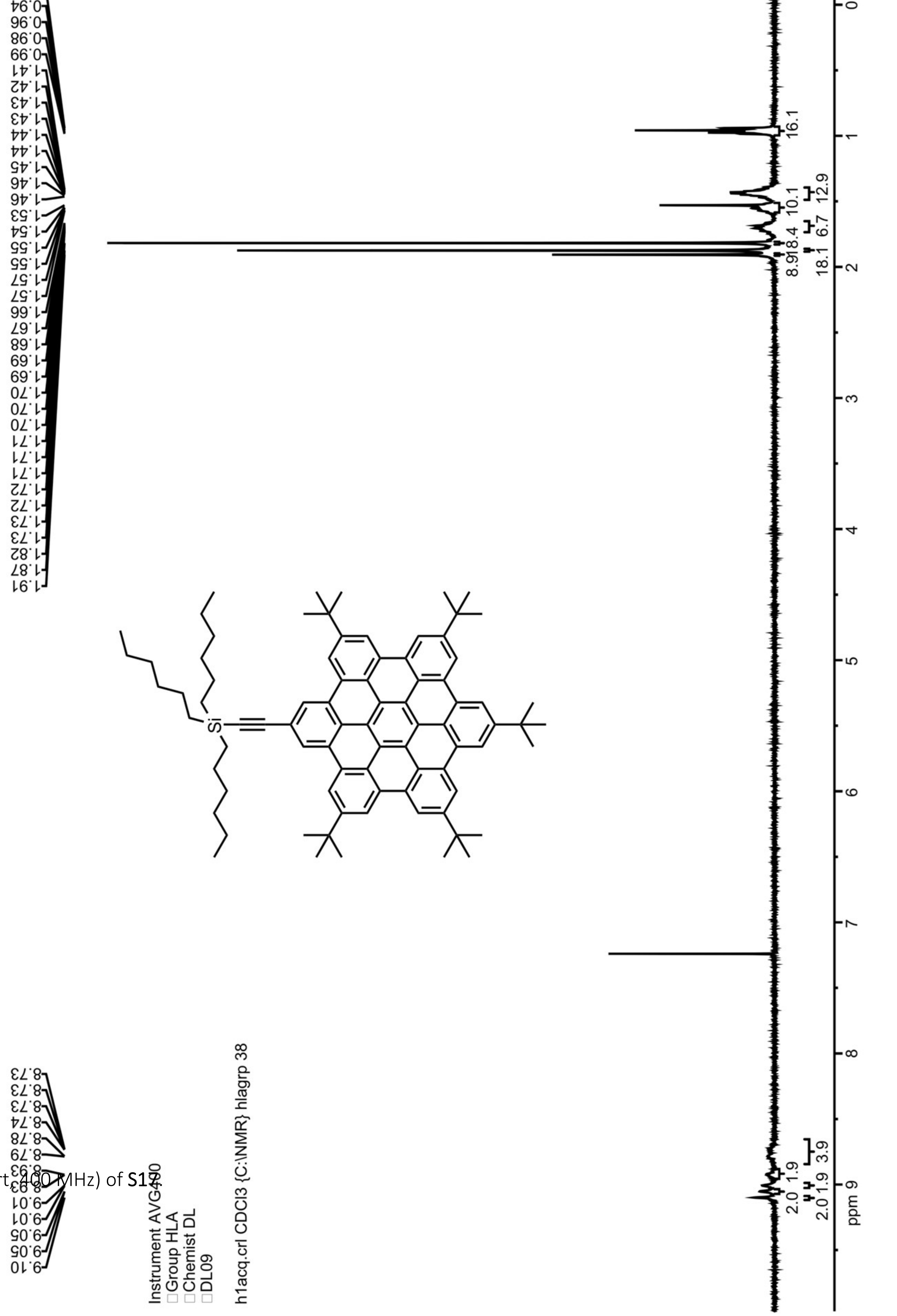

Figure S87: ^{1}H NMR ($CDCl_3$, rt, 400 MHz) of **S170**

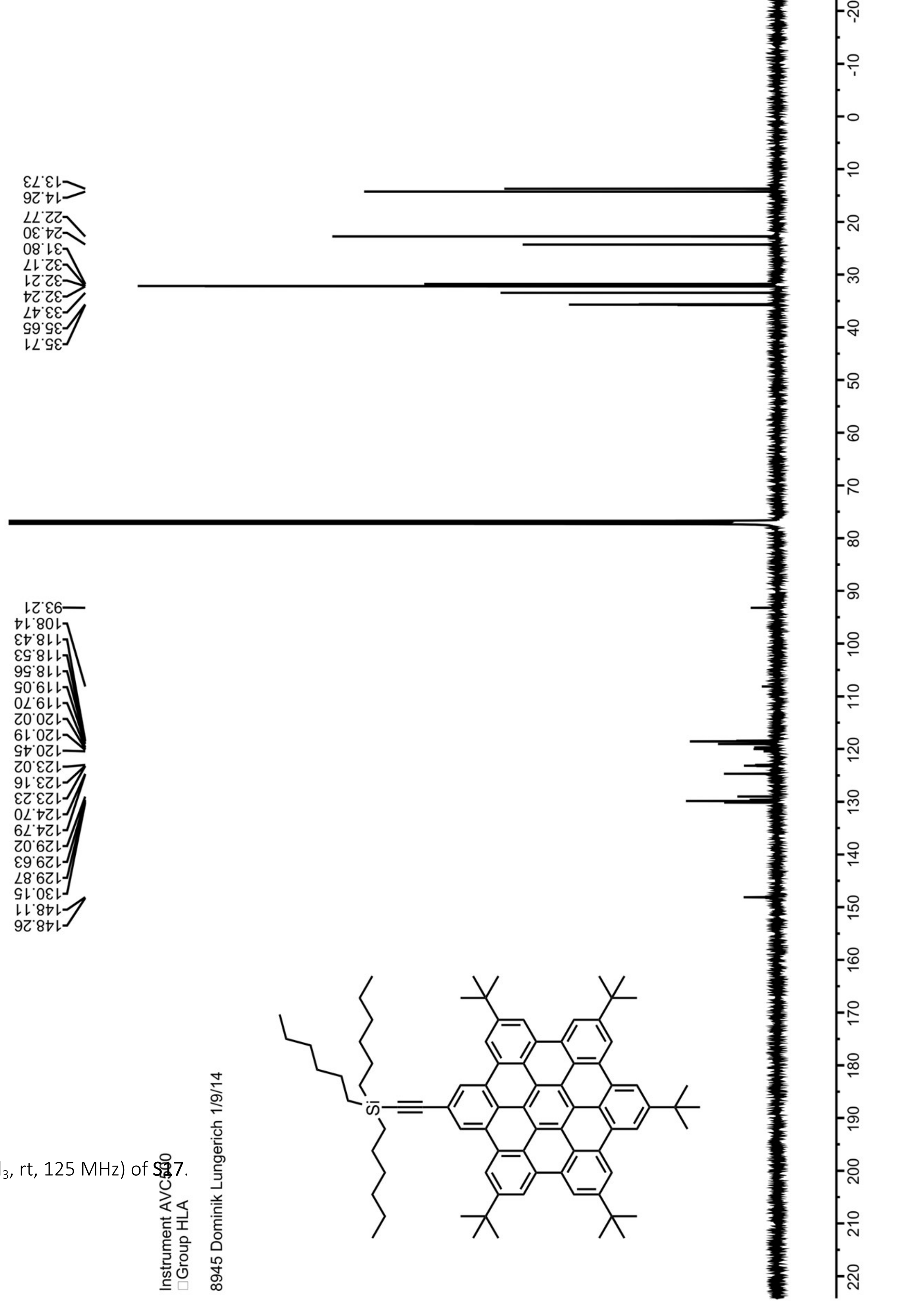

Figure S88: ^{13}C NMR ($CDCl_3$, rt, 125 MHz) of **S17**.

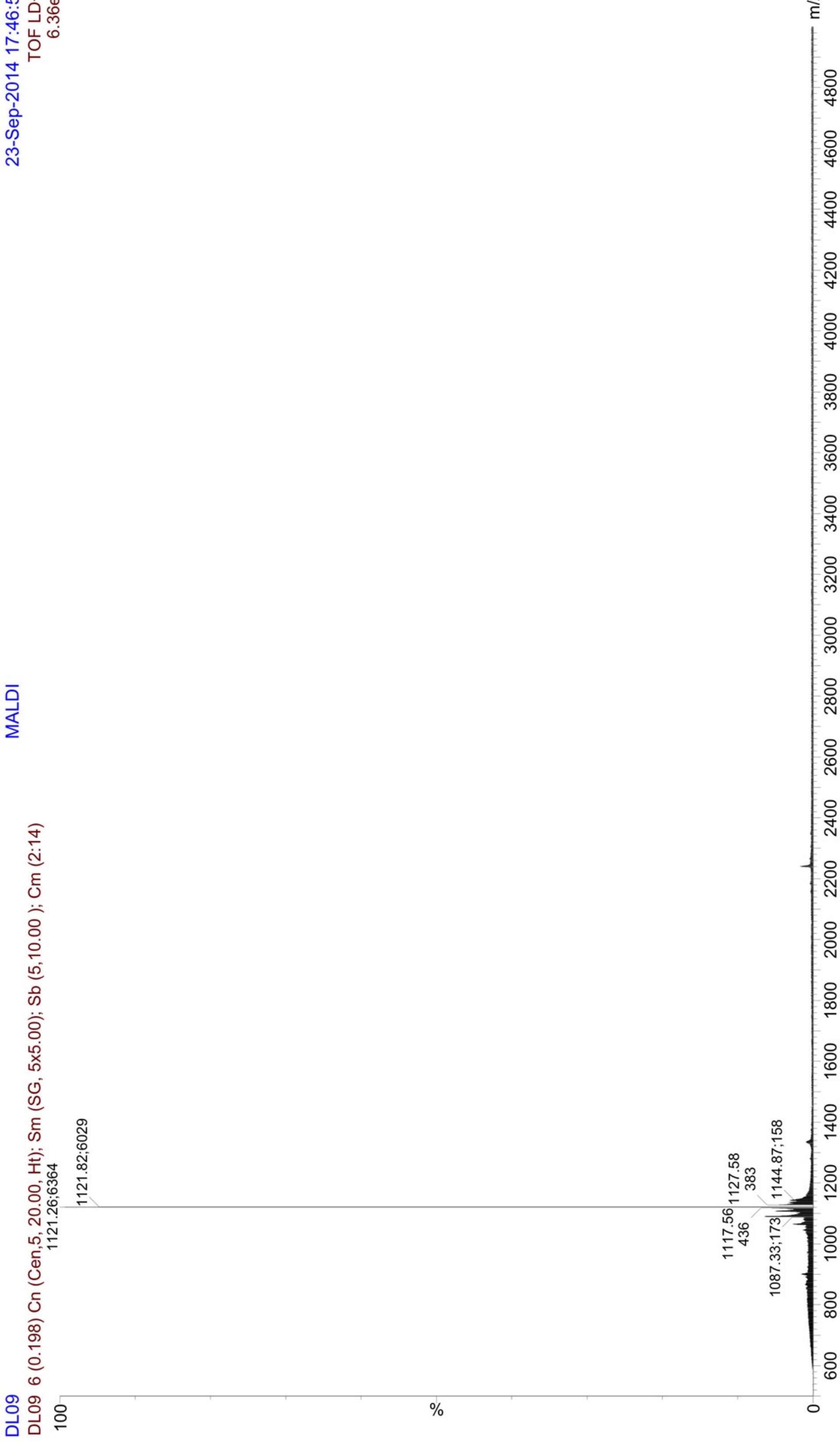

Figure S89: MS (LDI) of **S17**.

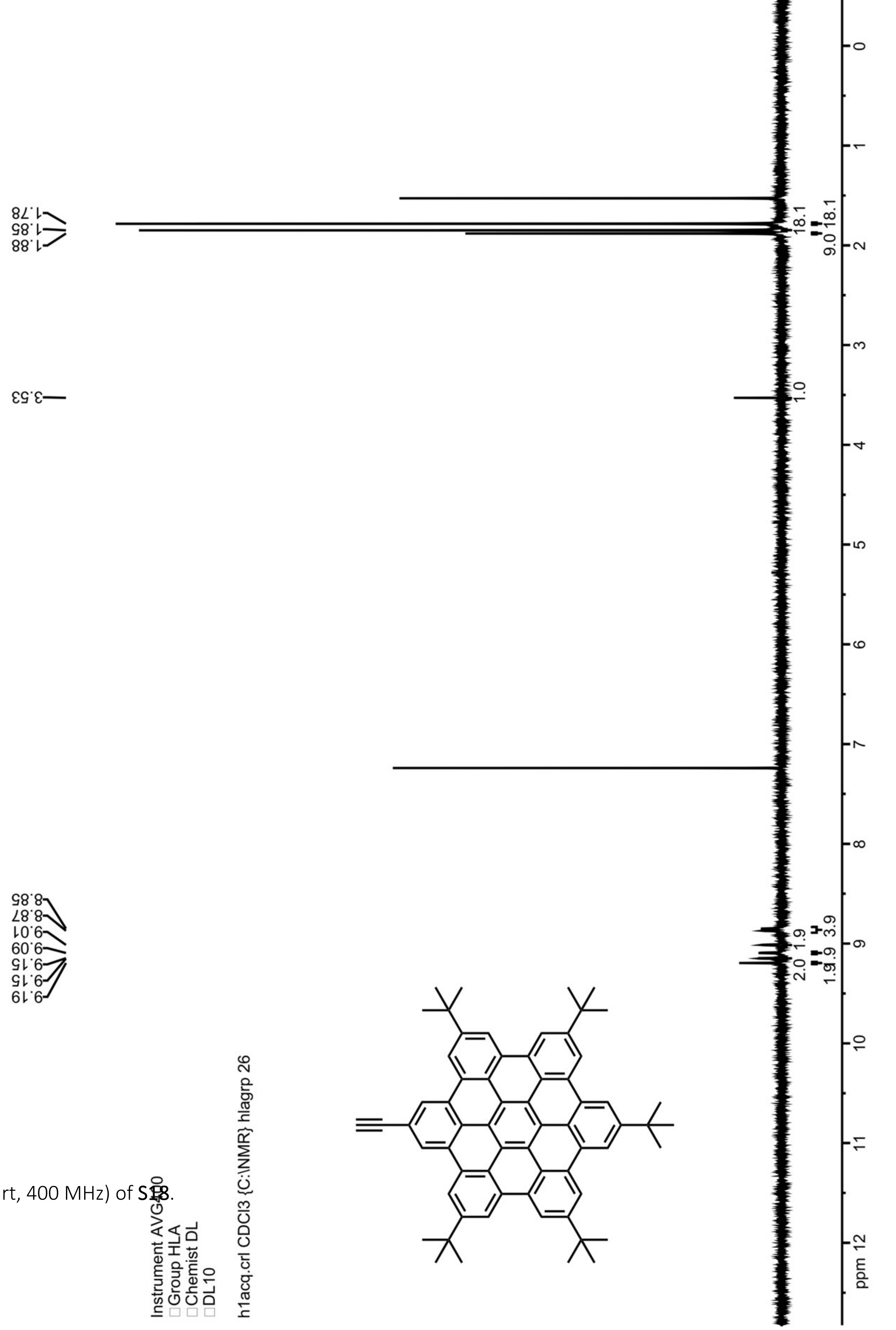

Figure S90: ^{1}H NMR ($CDCl_3$, rt, 400 MHz) of **S18**.

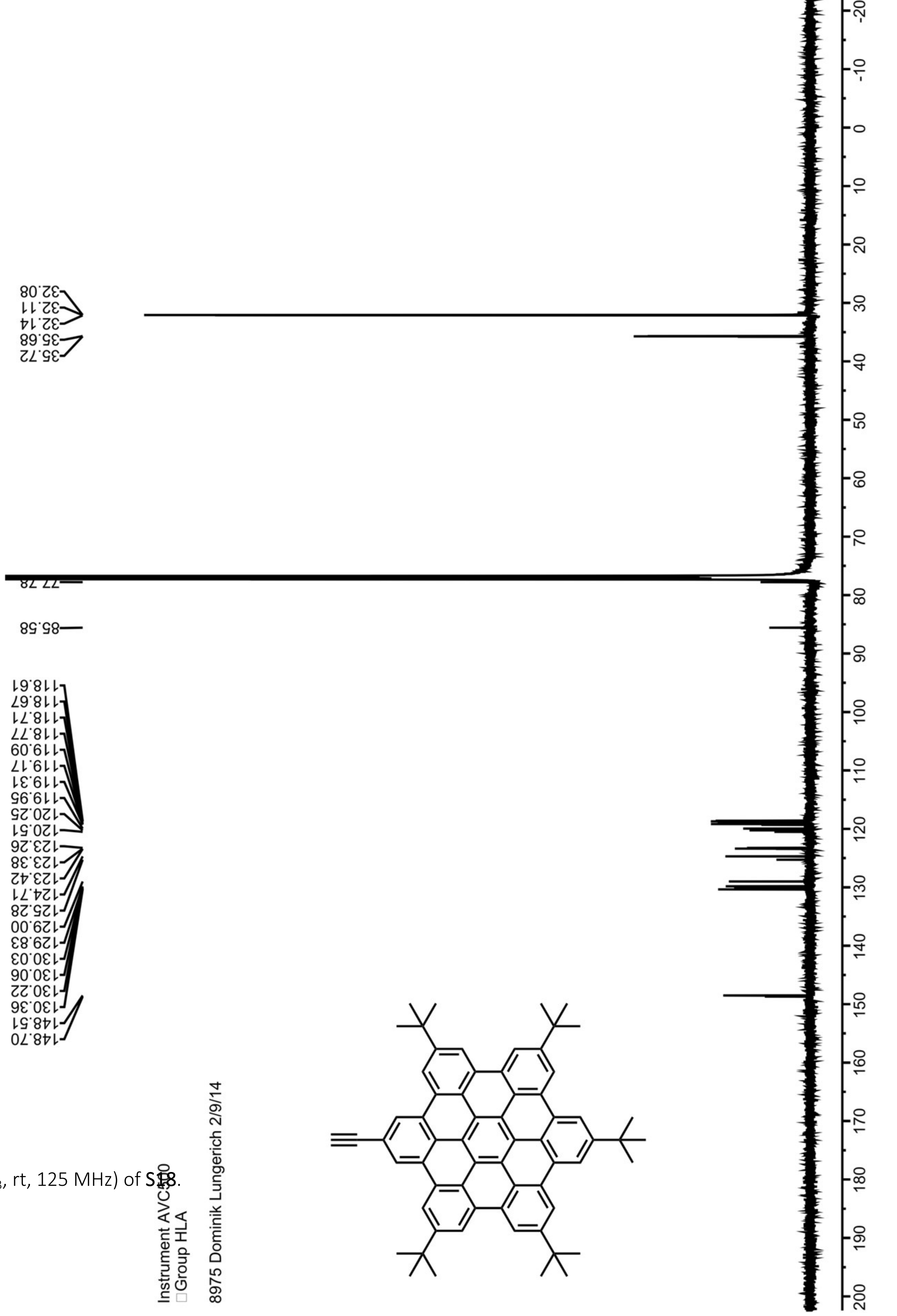

Figure S91: ^{13}C NMR ($CDCl_3$, rt, 125 MHz) of **S18**.

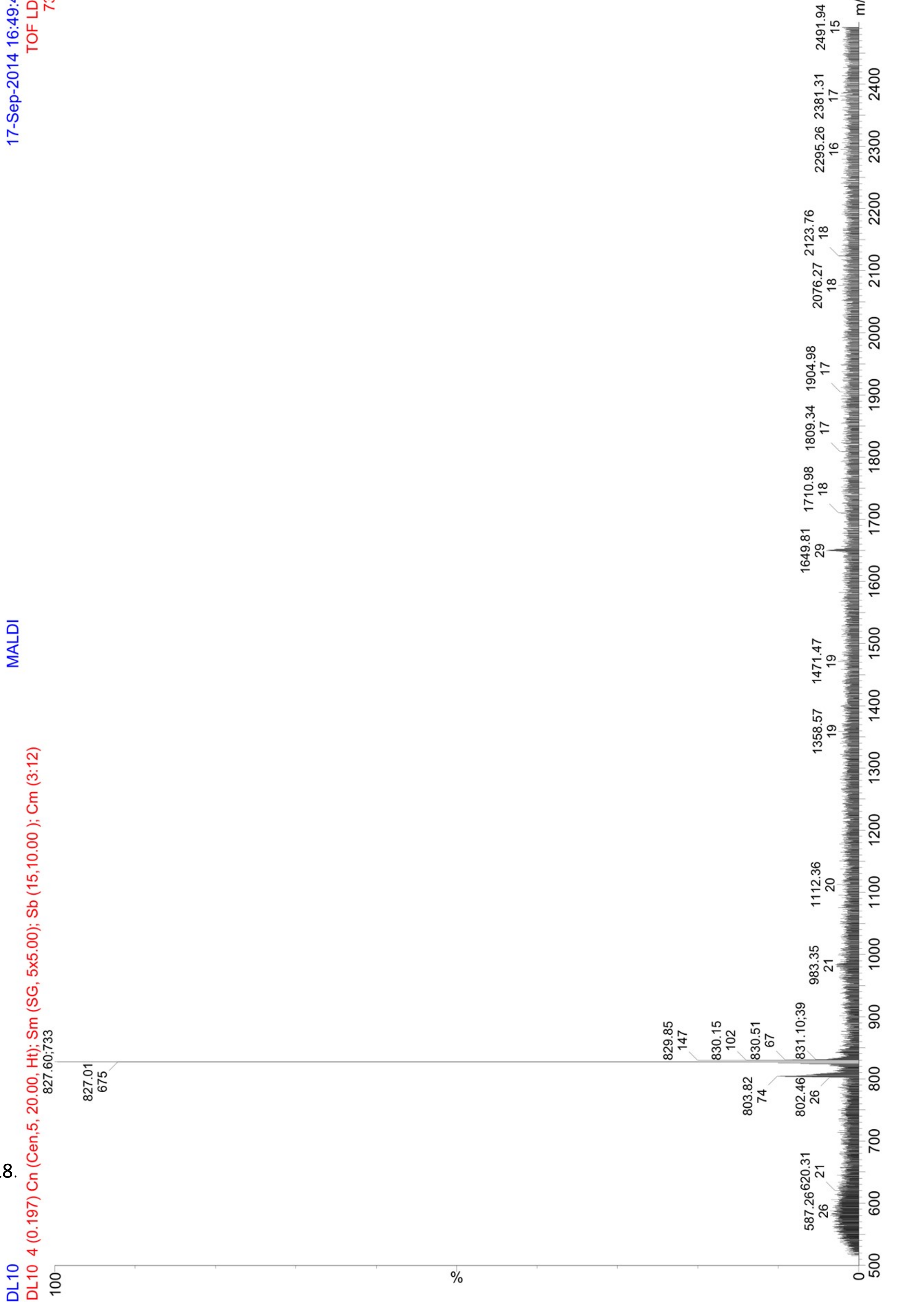

Figure S92: MS (LDI) of **S18**.

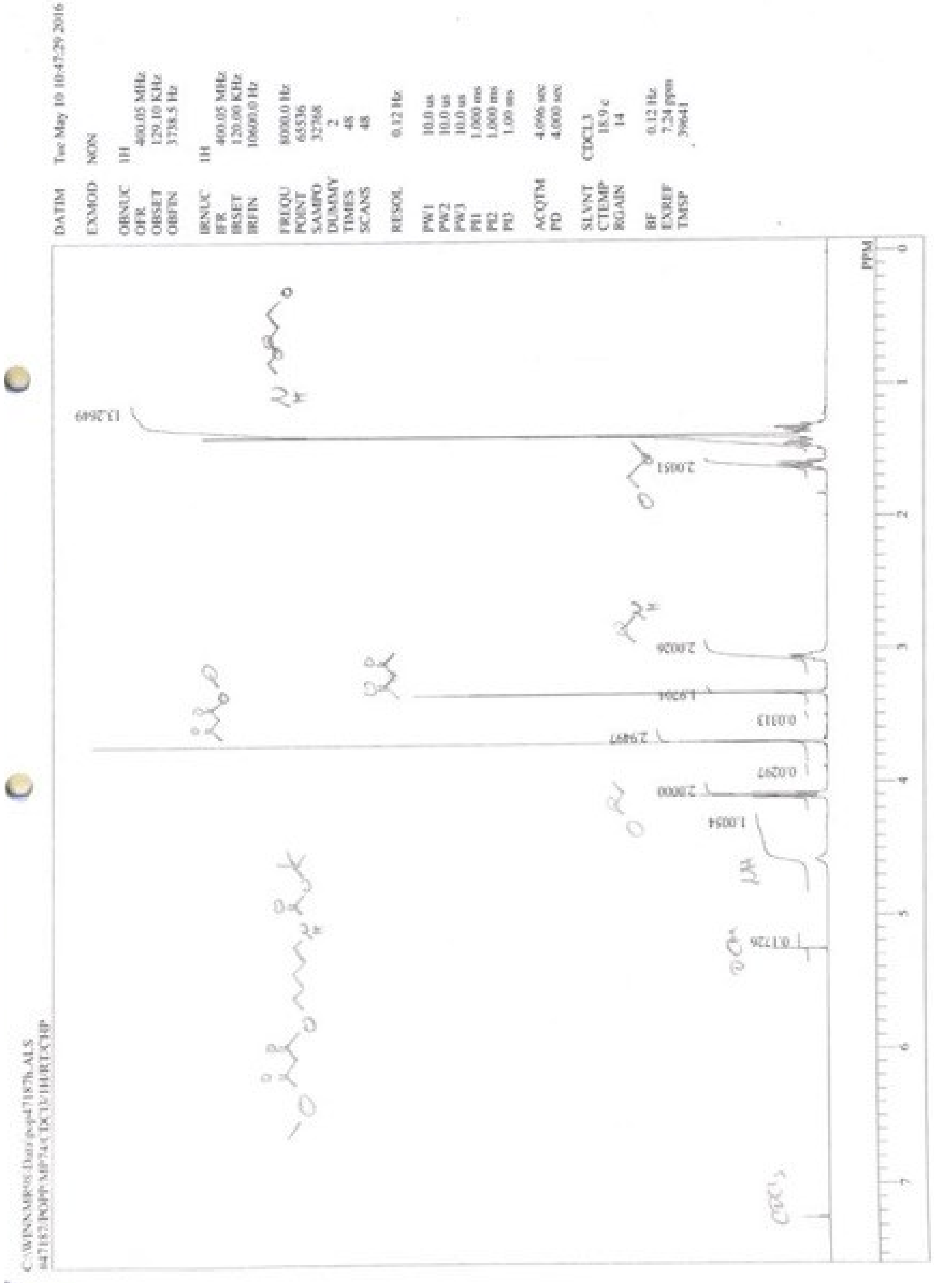

Figure S93: ^{1}H NMR ($CDCl_3$, rt, 400 MHz) of **S19**.

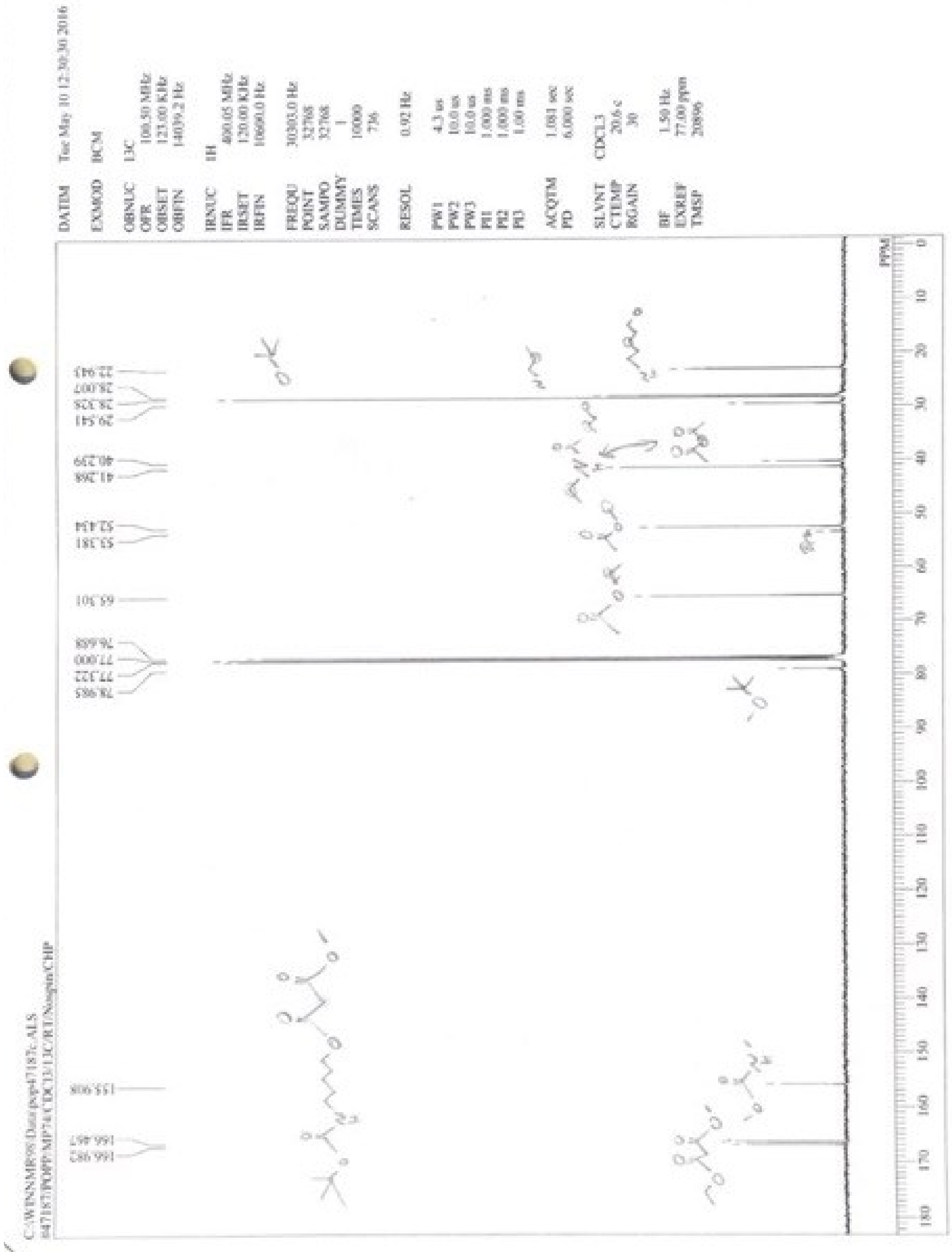

Figure S94: ^{13}C NMR ($CDCl_3$, rt, 100 MHz) of **S19**.

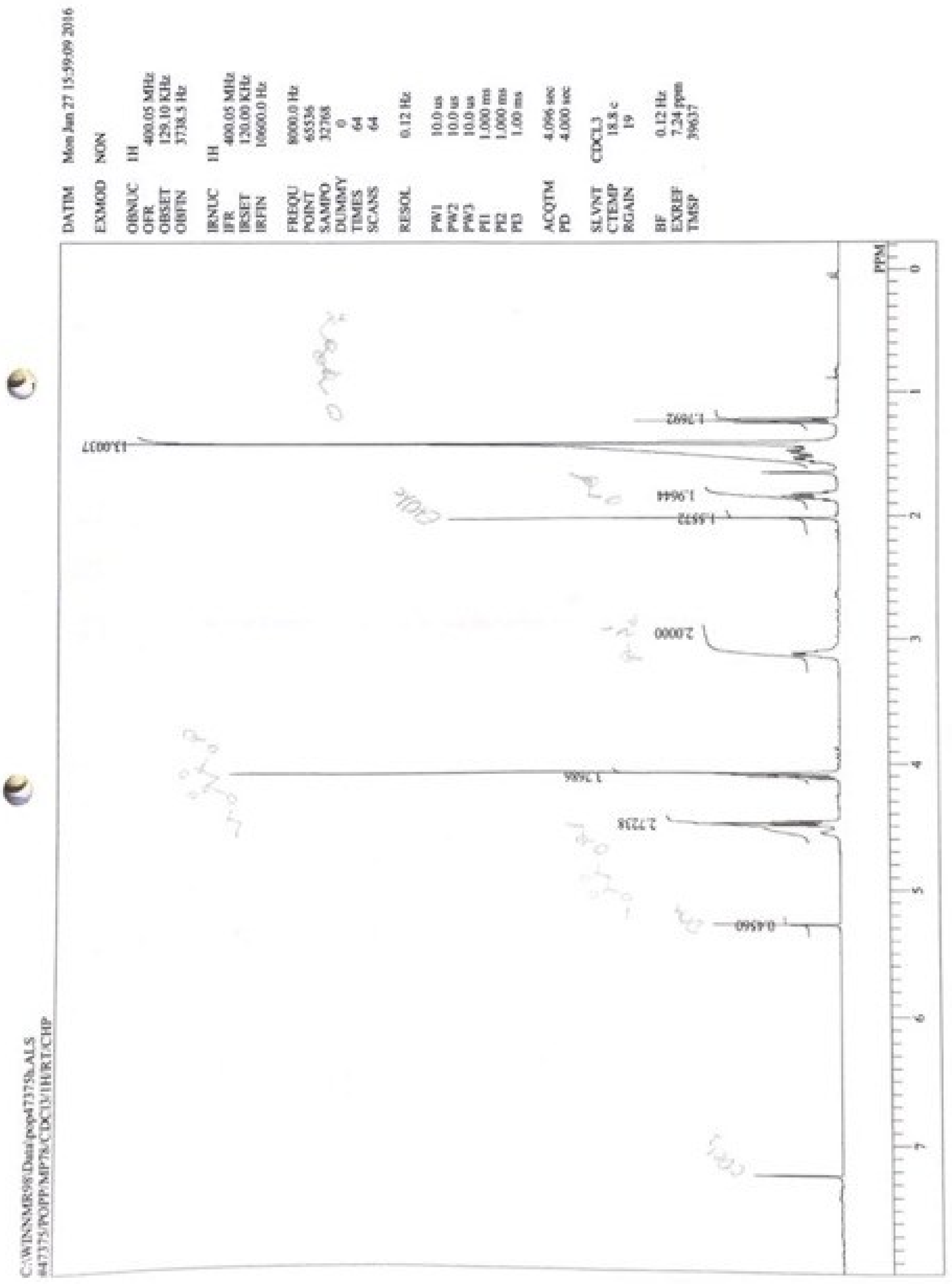

Figure S95: ^{1}H NMR ($CDCl_3$, rt, 400 MHz) of S20.

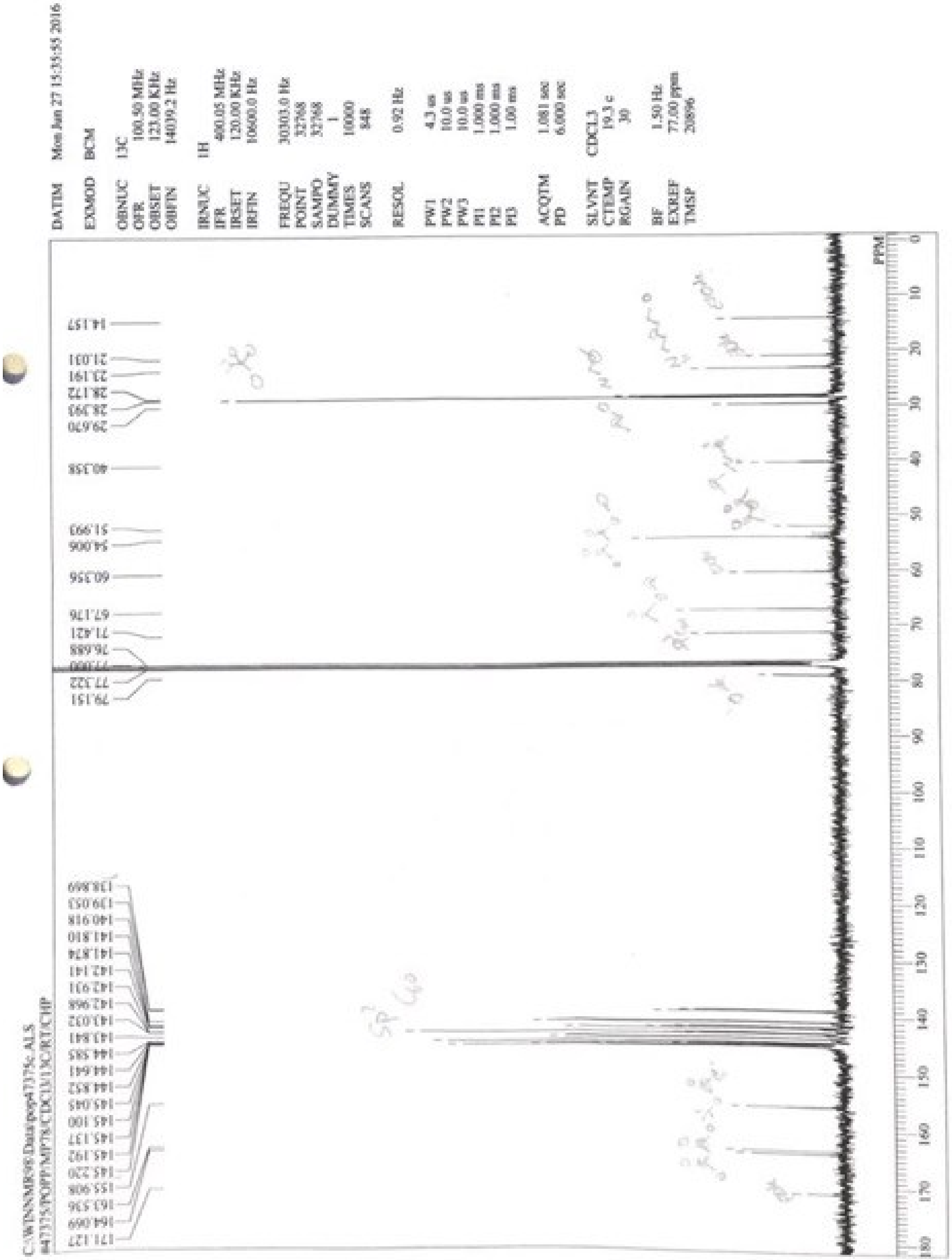

Figure S96: ^{13}C NMR ($CDCl_3$, rt, 100 MHz) of **S20**.

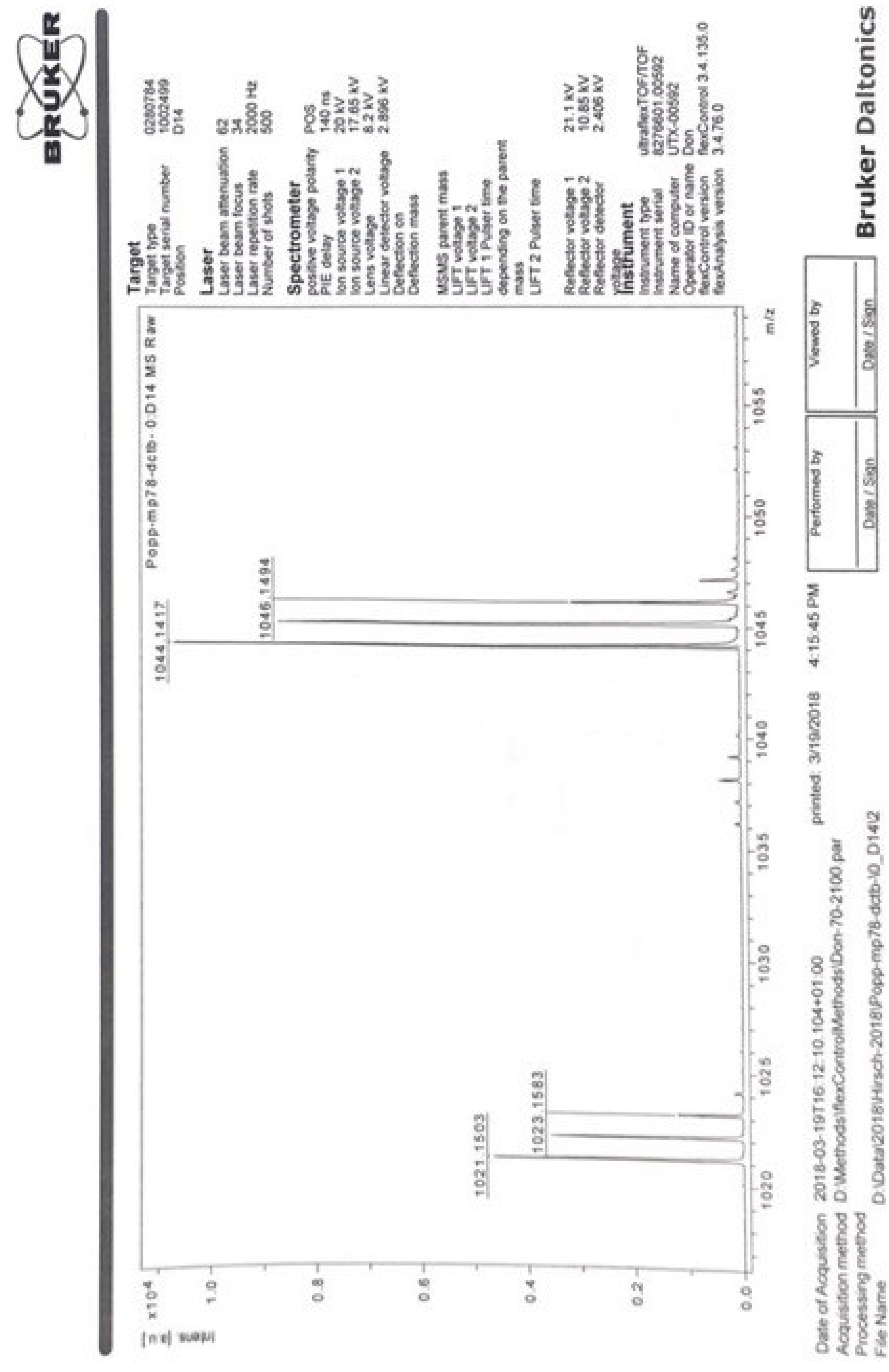

Figure S97: HRMS (MALDI, DCTB) of **S20**.

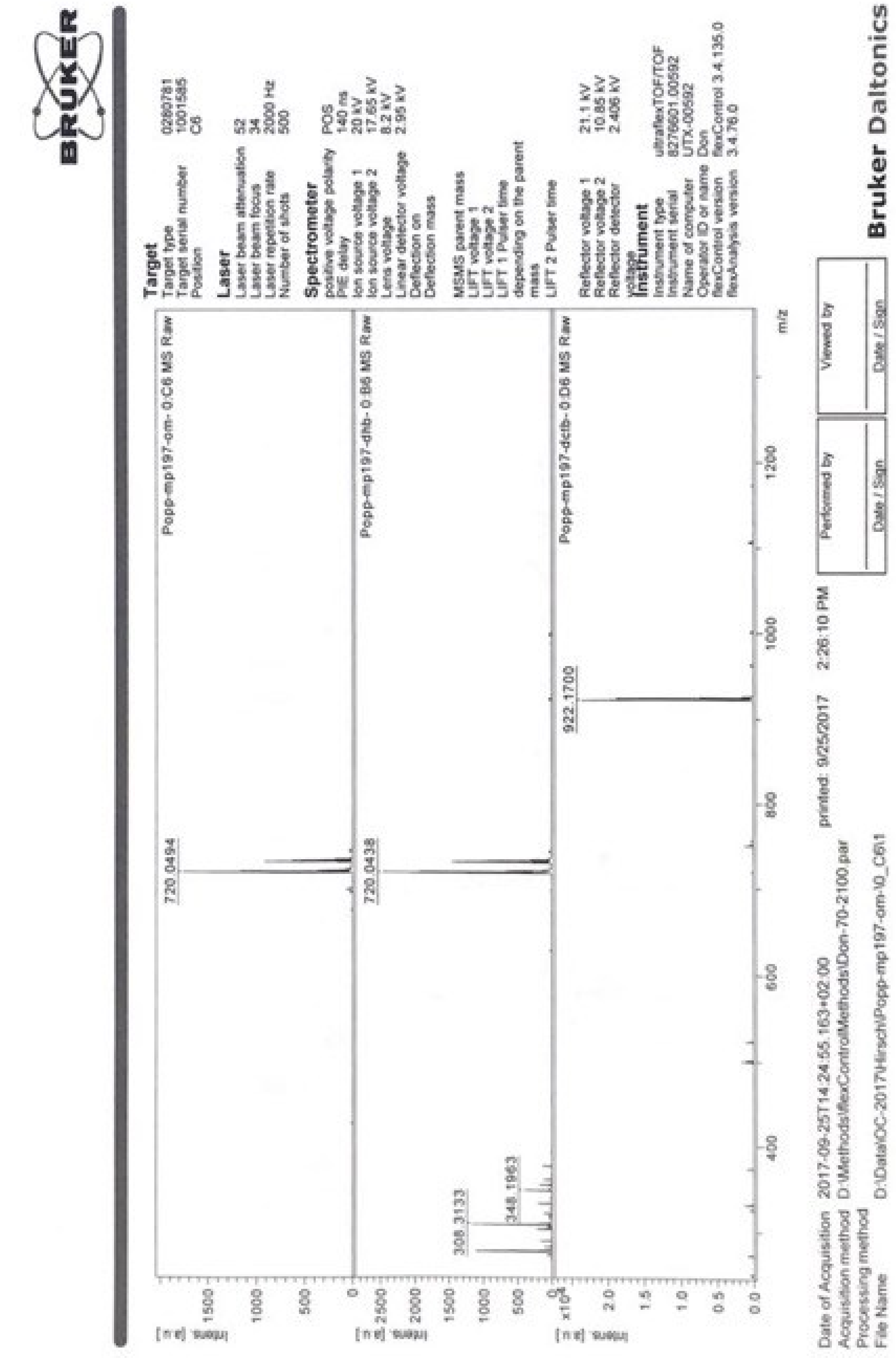

Figure S98: HRMS (MALDI, DCTB) of **S21**.

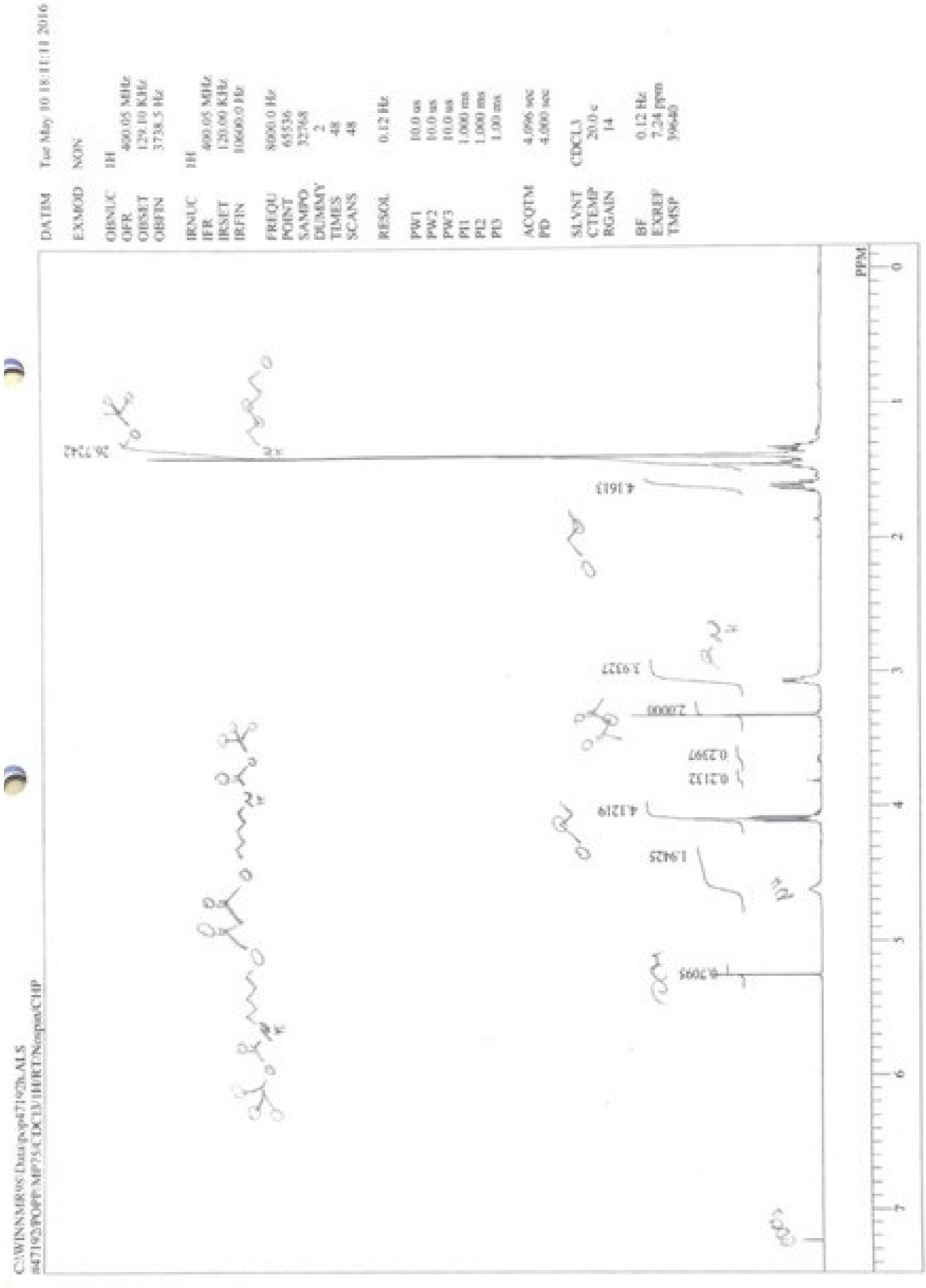

Figure S99: ^{1}H NMR ($CDCl_3$, rt, 400 MHz) of **S22**.

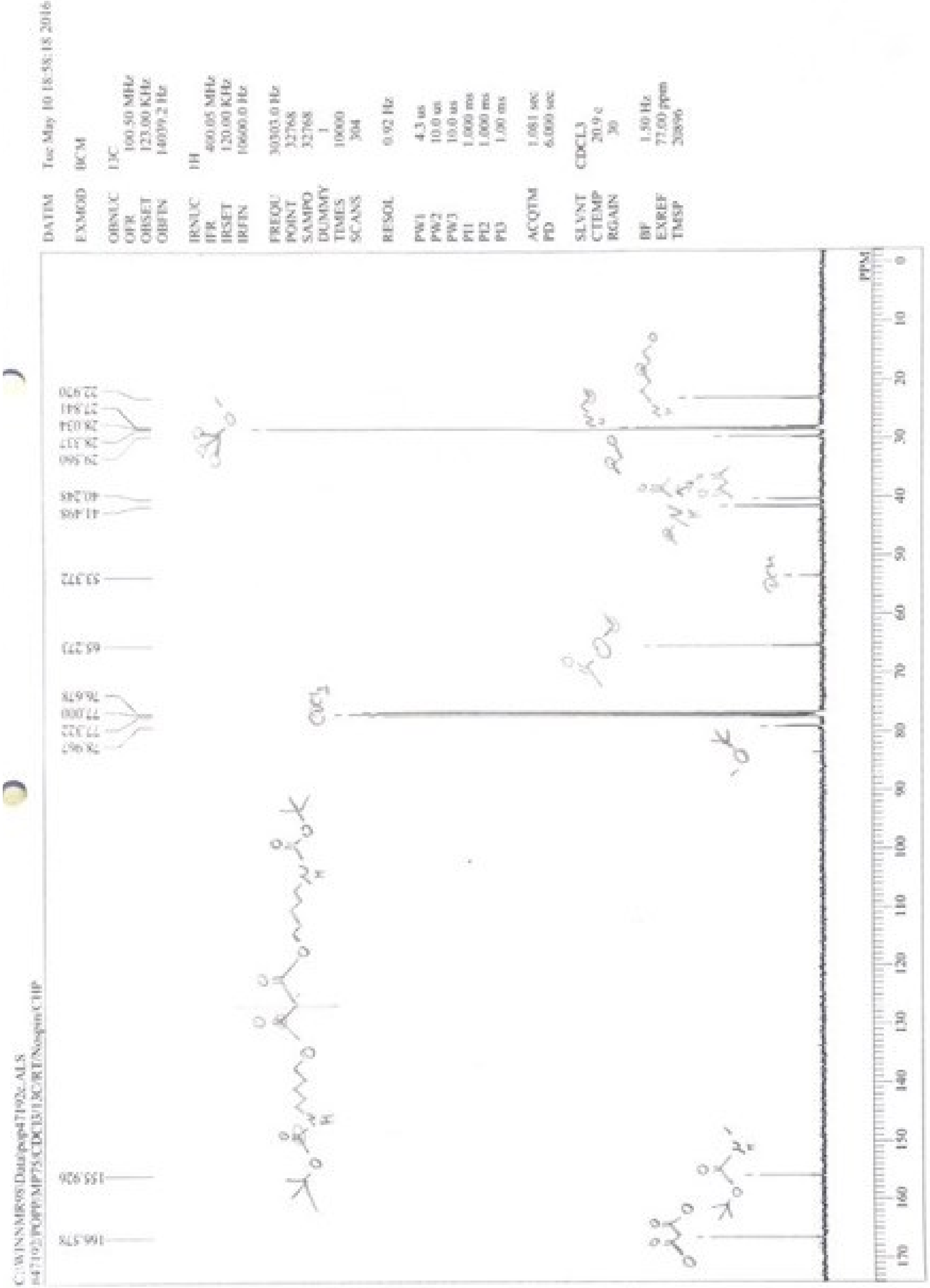

Figure S100: ^{13}C NMR ($CDCl_3$, rt, 100 MHz) of **S22**.

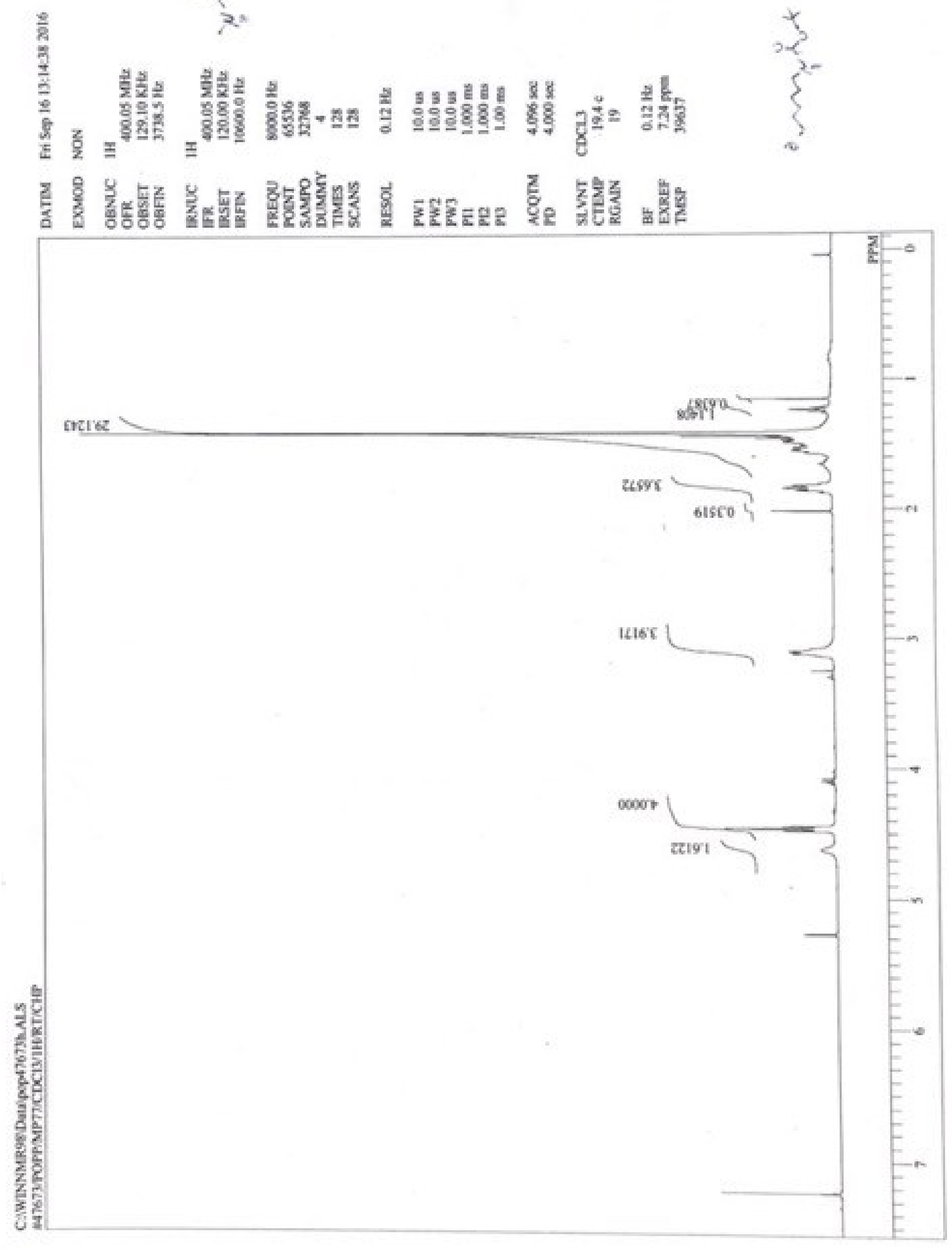

Figure S101: ^{1}H NMR ($CDCl_3$, rt, 400 MHz) of **S23**.

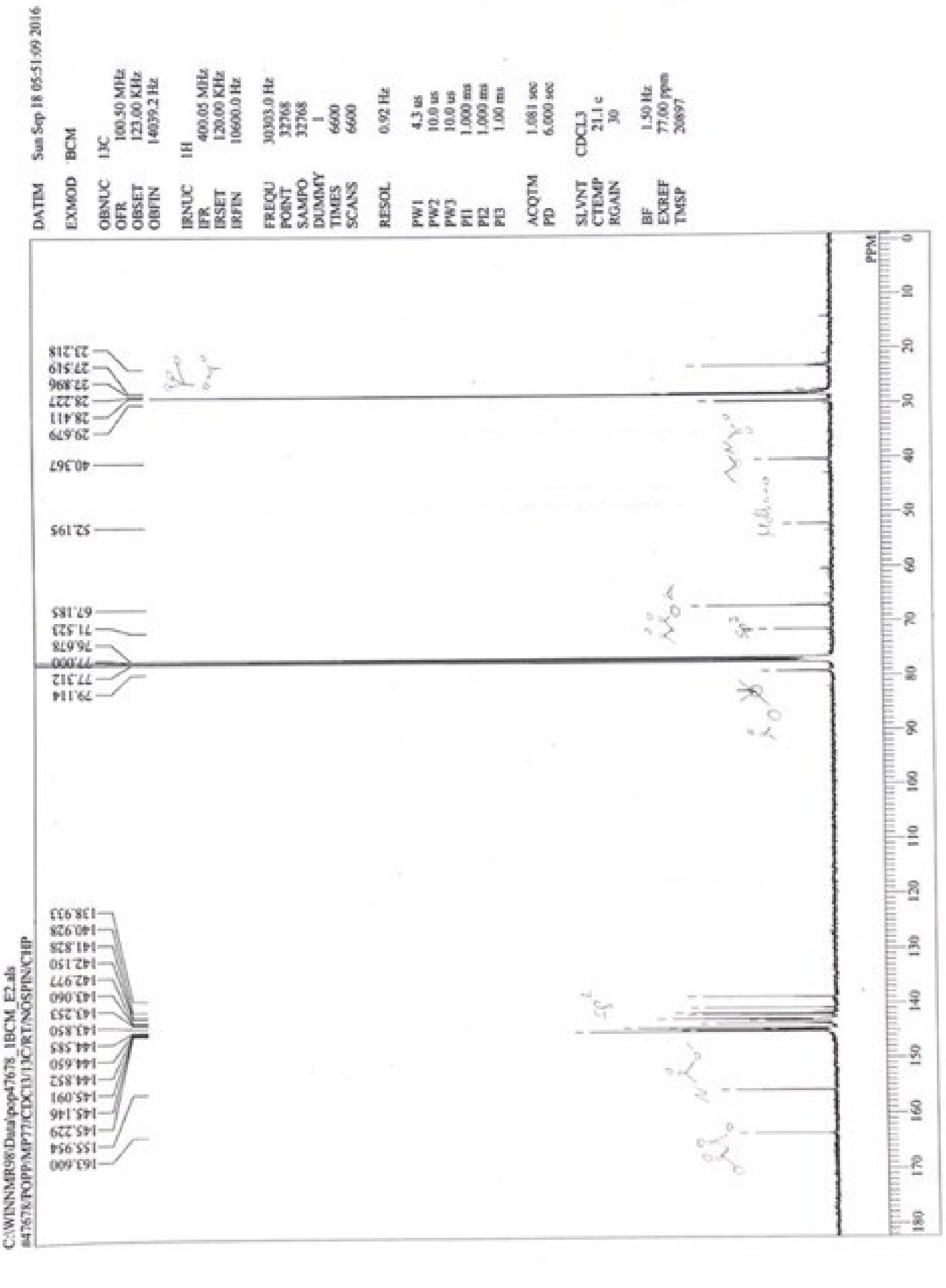

Figure S102: ^{13}C NMR ($CDCl_3$, rt, 100 MHz) of **S23**.

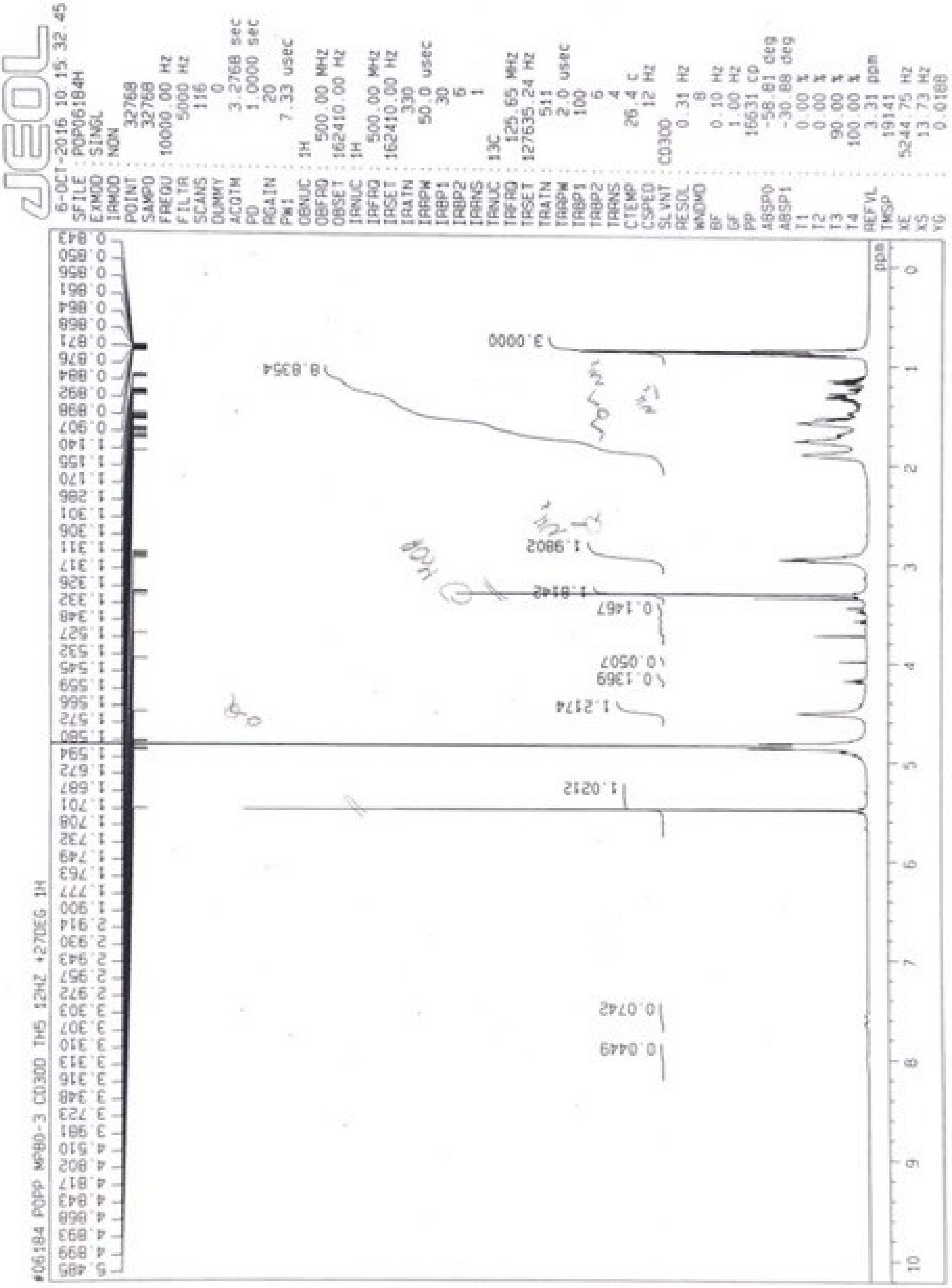

Figure S103: ^{1}H NMR ($CDCl_3$, rt, 400 MHz) of **3**.

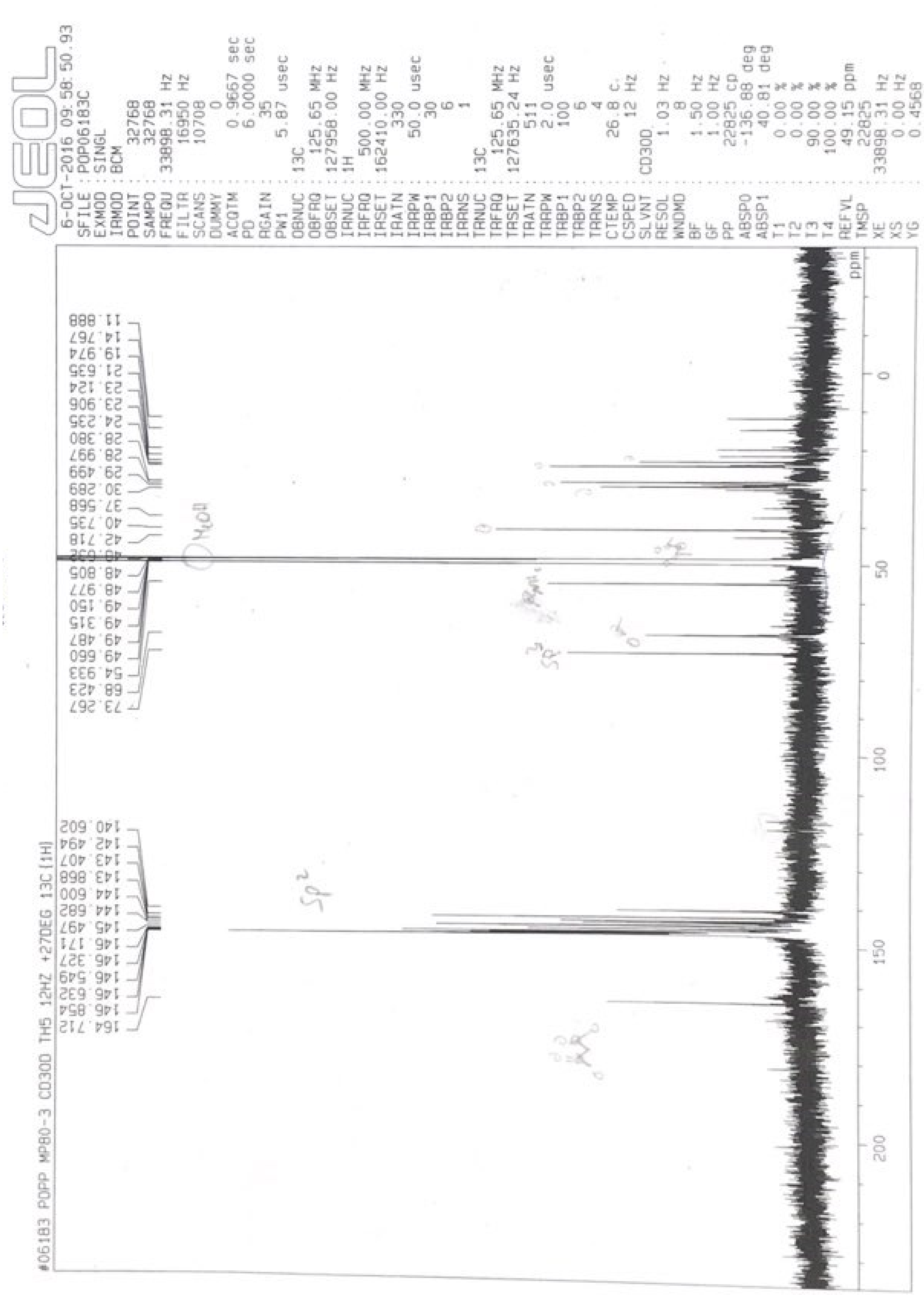

Figure S104: ^{13}C NMR ($CDCl_3$, rt, 100 MHz) of **3**.

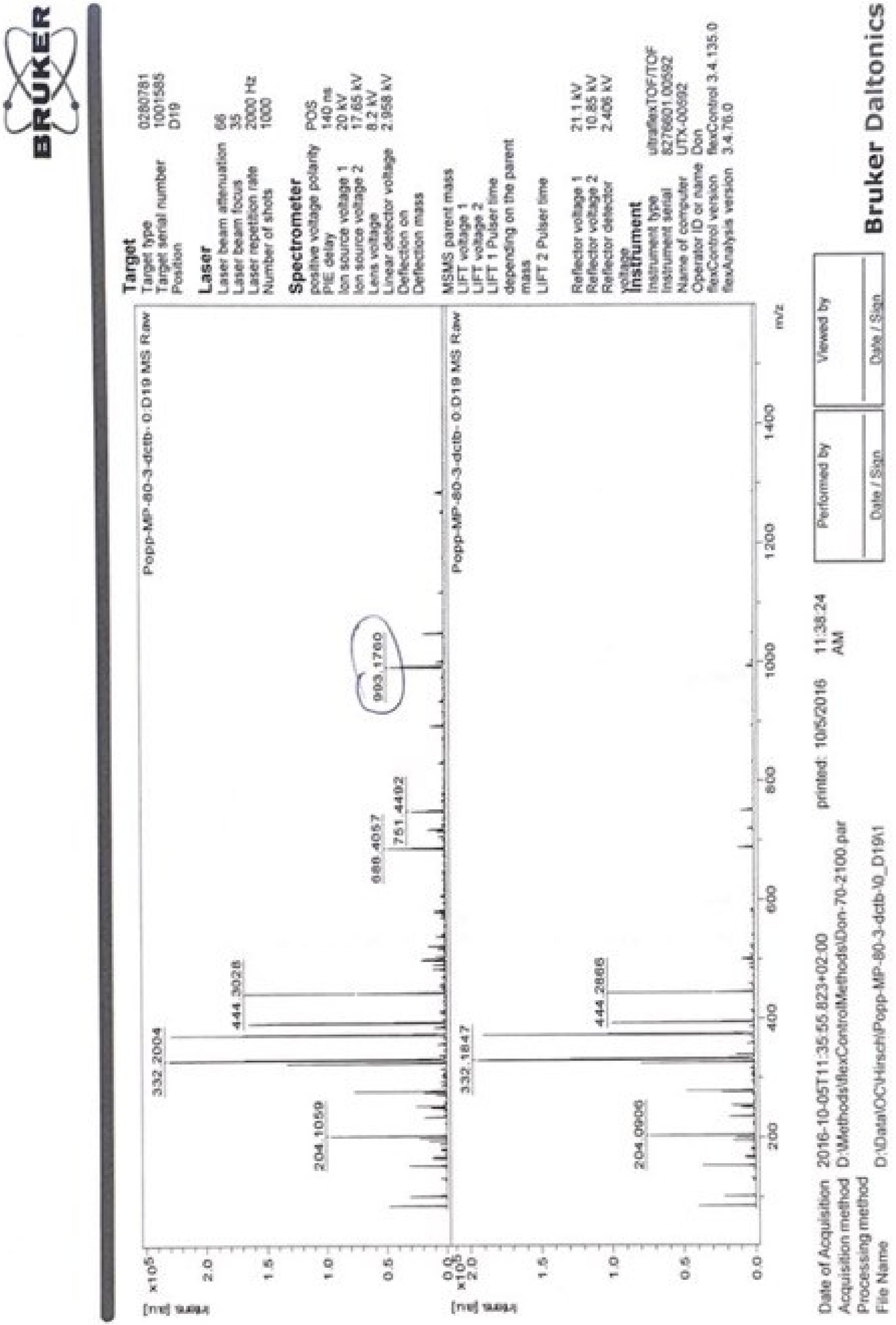

Figure S105: MS (MALDI, DCTB) of **3**.
